# Hydrogen‐Borrowing Alkylation of 1,2‐Amino Alcohols in the Synthesis of Enantioenriched γ‐Aminobutyric Acids

**DOI:** 10.1002/anie.202100922

**Published:** 2021-02-24

**Authors:** Christopher J. J. Hall, William R. F. Goundry, Timothy J. Donohoe

**Affiliations:** ^1^ Department of Chemistry University of Oxford Chemistry Research Laboratory Mansfield Road Oxford OX1 3TA UK; ^2^ Early Chemical Development Pharmaceutical Sciences, R&D AstraZeneca Macclesfield SK10 2NA UK

**Keywords:** amino acids, amino alcohols, catalysis, hydrogen borrowing, iridium

## Abstract

For the first time we have been able to employ enantiopure 1,2‐amino alcohols derived from abundant amino acids in C−C bond‐forming hydrogen‐borrowing alkylation reactions. These reactions are facilitated by the use of the aryl ketone Ph*COMe. Racemisation of the amine stereocentre during alkylation can be prevented by the use of sub‐stoichiometric base and protection of the nitrogen with a sterically hindered triphenylmethane (trityl) or benzyl group. The Ph* and trityl groups are readily cleaved in one pot to give γ‐aminobutyric acid (GABA) products as their HCl salts without further purification. Both steps may be performed in sequence without isolation of the hydrogen‐borrowing intermediate, removing the need for column chromatography.

## Graphical abstract

The scope of carbon–carbon bond‐forming hydrogen‐borrowing reactions has been expanded to include 1,2‐amino alcohols derived from both natural and unnatural amino acids. The vulnerable amine stereocentre is preserved in the reaction by the use of a bulky nitrogen protecting group (trityl or benzyl), with the products being readily cleaved under acidic conditions to the corresponding γ‐aminobutyric acids without further purification.

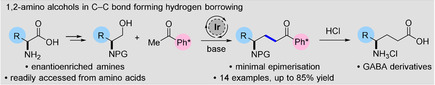

The formation of carbon–carbon bonds adjacent to carbonyl groups often relies on the formation of an enolate, followed by trapping with reactive electrophiles such as alkyl halides or tosylates and mesylates.^[1]^ Such an approach, whilst effective, is not without issues, such as the cryogenic temperatures required, the toxic nature of many alkyl halides, and competing enolate *O*‐alkylation all proving problematic.^[2]^

An attractive alternative employs hydrogen‐borrowing catalysis, which enables alcohols to be used as electrophiles in alkylation reactions with enolates.^[3]^ This avoids many of the problems associated with traditional electrophiles, with the additional advantage of water being the only stoichiometric waste product. In recent years, we have reported the use of catalytic iridium to enable the alkylation reactions of pentamethylphenyl (Ph*) methyl ketone with a range of primary and secondary alcohols, forming branched products, as well as with diols to form cyclohexanes.^[4]^ In the case of secondary alcohols and diols, the use of an iridium(I) catalyst with a chiral phosphine ligand delivered enantioenriched products (Scheme 1 A).^[5]^ Crucial to these results was the use of the sterically hindered Ph* methyl ketone—the bulky *ortho*‐substituted aromatic ring is twisted out of conjugation and protects the carbonyl from reduction and dimerization reactions during the alkylation. Furthermore, the Ph* motif is easily removed by either *ipso*‐protonation or bromination to give a range of acyl functional groups in one pot.

**Scheme 1:**
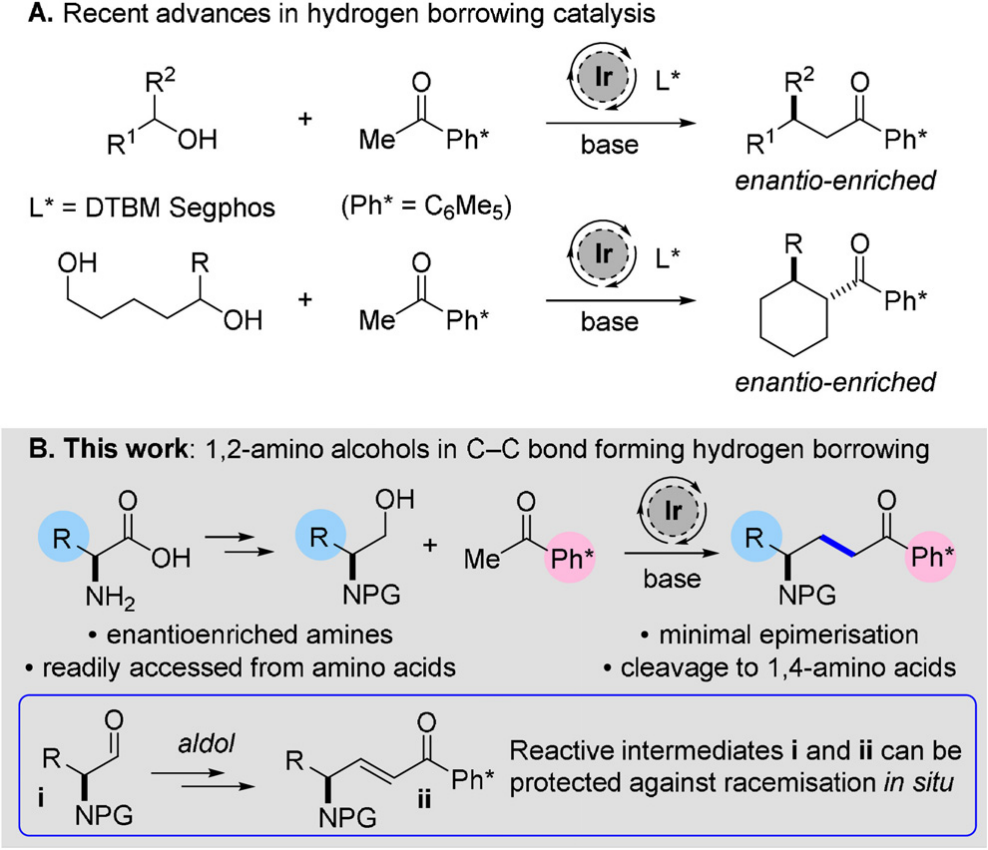
A) Previous work, B) proposed hydrogen‐borrowing reaction of 1,2‐amino alcohols with Ph* methyl ketone.

The scope of hydrogen‐borrowing alkylation in the literature has mainly comprised simple aliphatic and aromatic alcohols, while substrate alcohols containing amine functionality are under‐represented.^[6]^ Given the ubiquity of nitrogen‐containing compounds,^[7]^ we envisaged expanding the scope of C−C bond‐forming hydrogen borrowing to 1,2‐amino alcohols. Our decision to use 1,2‐amino alcohols was in part driven by the fact that the classical equivalent of such alcohols in alkylation reactions are highly toxic 1,2‐amino halides.^[8]^ Another attractive facet of 1,2‐amino alcohols was that they may be readily synthesized in enantiopure form from amino acids, allowing us to examine the functional group and stereochemical compatibility of a variety of side chains. The proposed transformation was to first synthesize protected 1,2‐amino alcohols from amino acids, optimize a hydrogen borrowing alkylation reaction with Ph* methyl ketone, and remove the Ph* to give 1,4‐amino acids, hopefully in enantioenriched form (Scheme 1 B).

The desired products are of significant medicinal interest as structural analogues of γ‐aminobutyric acid (GABA), a key inhibitory neurotransmitter.^[9]^ Moreover, we envisaged a study of the stereochemical integrity of chiral 1,2‐amino alcohols in hydrogen‐borrowing alkylations.^[10]^ Any retention of absolute stereochemistry from the starting amino alcohol would provide a new route to enantioenriched products. Our initial concern lay in the vulnerability of reactive intermediates **i** and **ii** (Scheme 1 B), formed during alkylation, to the basic reaction conditions: this was especially pertinent given our previous work which had clearly showed racemization adjacent to the alcohol with all carbon substituents.^[4c]^

We began with the hydrogen‐borrowing reaction between Ph* methyl ketone **2** and commercially available (*S*)‐N‐benzyl‐*L*‐prolinol **1 a**, using conditions developed within our group for reactions of Ph* ketones: [(cod)IrCl]_2_ **A** (2 mol %), dppBz (4 mol %) and KOH (2.0 equiv.) in PhMe (1.0 M) at 85 °C for 16 hours (Table 1, entry 1).^[4]^ Whilst we were delighted to see the formation of the desired product **3 a**, both the yield (20 %) and e.r. (77:23) were unsatisfactory.

**Table 1:** Optimization of a 1,2‐amino alcohol hydrogen‐borrowing reaction using (*S*)‐*N*‐benzyl‐*L*‐prolinol **1 a** as a model substrate. 
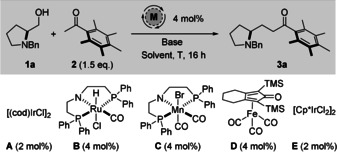

Entry	[M]	Base (equiv)	*T* [°C]	Solvent (M)	Yield^[b]^	e.r. **3 a** ^[c]^
1	**A** ^[d]^	KOH (2.0)	85	PhMe (1.0 M)	20	77:23
2	**B**	KOH (2.0)	85	PhMe (1.0 M)	41	81:19
3	**C**	KOH (2.0)	85	PhMe (1.0 M)	–^[e]^	–^[e]^
4	**D** ^[f]^	KOH (2.0)	85	PhMe (1.0 M)	–^[g]^	–^[g]^
5	**E**	KOH (2.0)	85	PhMe (1.0 M)	51	82:18
6	**E**	KOH (2.0)	85	no solvent	63	82:18
7	**E**	KOH (2.0)	110	no solvent	62	65:35
8	**E**	KOH (2.0)	65	no solvent	49	88:12
9	**E**	KO*t*Bu (2.0)	85	no solvent	18	90:10
10	**E**	NaO*t*Bu (2.0)	85	no solvent	10	96:4
11	**E**	NaO*t*Bu (1.0)	85	no solvent	42	95:5
12	**E**	NaO*t*Bu (0.5)	85	no solvent	63	92:8
13	**E**	NaO*t*Bu (0.5)	85	*t*BuOH (0.5 M)	50	92:8
14	**E**	NaO*t*Bu (0.5)	85	*t*BuOH (1.0 M)	46	95:5
**15**	**E**	**NaO*t*Bu (0.5)**	**85**	***t*** **BuOH (2.5M)**	**72**	**94:6**

[a] All reactions were performed on a 0.4 mmol scale. [b] Isolated yield. [c] Determined by normal phase HPLC analysis using a chiral stationary phase. [d] With 4 mol % dppBz. [e] Complex mixture formed. [f] With 8 mol % Me_3_NO. [g] No reaction. cod=1,5‐cyclooctadiene; dppBz=1,2‐bis(diphenylphosphino)benzene; TMS=trimethylsilane; Cp*=pentamethylcyclopentadienyl.

Inspired by recent developments in hydrogen borrowing utilizing earth‐abundant catalysts,^[11]^ we screened a range of transition metal complexes. Whilst Ru‐MACHO catalyst **B** led to an improvement in both yield (41 %) and e.r. (81:19), its manganese analogue **C** resulted in only decomposition of the starting alcohol (Table 1, entries 2 and 3). The use of a mixture of iron complex **D** and Me_3_NO (8 mol %) resulted in no reaction, with prolinol **1 a** and Ph* methyl ketone both appearing unchanged in the crude ^1^H NMR spectrum (Table 1, entry 4). Upon switching to [Cp*IrCl_2_]_2_ **E** we observed an increase in both yield (51 %) and e.r. (82:18), which was then used as a basis for further optimization (Table 1, entry 5). Next, the reaction was conducted in the absence of toluene solvent; this resulted in a further increase in yield to 63 %, with no change in e.r. (Table 1, entry 6).

Upon increasing the reaction temperature to 110 °C, we observed product formation in a similar 62 % yield accompanied by a large drop in e.r. to 65:35 (Table 1, entry 7). As a corollary, lowering the temperature to 65 °C did result in an increase in e.r. to 88:12, but at the expense of conversion with the yield dropping to 49 % (Table 1, entry 8). In an attempt to prevent deprotonation of the acidic α‐protons, and preserve enantioenrichment, we screened the more hindered alkoxide bases KO*t*Bu and NaO*t*Bu. Although this modification resulted in an increase in product e.r. to 90:10 and 96:4, respectively, it was accompanied by marked decreases in yield to 18 % and 10 % (Table 1, entries 9 and 10). Despite the low yield, the high e.r. encouraged us to persist with NaO*t*Bu and we discovered that decreasing the equivalents of base first to equimolar, and then to sub‐stoichiometric amounts resulted in a dramatic increase in yield to 63 %—with only a slight reduction in e.r. to 92:8 (Table 1, entries 11 and 12). At this point it was observed that, in the absence of a solvent, the change in base from KOH to NaO*t*Bu was preventing full mixing of reactants. A range of concentrations of *t*BuOH as solvent were therefore screened, with a 2.5 M solution giving the final optimized yield and e.r. of 72 % and 94:6, respectively (Table 1, entries 13, 14 and 15).

With optimized conditions in hand, we set out to evaluate the reactivity of other amino acid‐derived alcohols. We were therefore surprised to find that although (*S*)‐*N*,*N*‐dibenzyl‐*L*‐alaninol **4 b** gave the corresponding *N*,*N*‐dibenzyl ketone **6 b** in 70 % yield, the e.r. of the product was only 73:27. To probe the effect of sterics on the reaction, we examined smaller and larger nitrogen protecting groups for both alaninols **4 a**–**c** and d_2_‐glycinols **5 a**–**c** (Table 2).

**Table 2:** Experiments to probe the effect of the *N*‐protecting group on unwanted deprotonation in 1,2‐amino alcohols during hydrogen‐borrowing alkylation.^[a]^

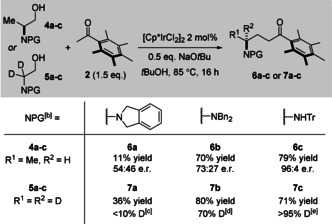

[a] Reaction conditions: amino alcohol **4** or **5** (0.4 mmol, 1 equiv.), **2** (0.6 mmol, 1.5 equiv.), [Cp*IrCl_2_]_2_ (0.008 mmol, 2 mol %), NaO*t*Bu (0.2 mmol, 0.5 equiv.), *t*BuOH (0.16 mL, 2.5 M), 85 °C, 16 hours. [b] NPG=**a** N(CH_2_)(CH_2_)C_6_H_4_; **b** NBn_2_; **c** NHTr. [c] Starting from 90 % D in alcohol **5 a**. [d] Starting from >95 % D in alcohol **5 b**. [e] Starting from >95 % D in alcohol **5 c**.

In both cases, we saw a clear trend between increasing steric bulk of the nitrogen protecting group and a decreasing degree of intermediate deprotonation—indicated either by racemization in products **6 a**–**c**, or by loss of deuterium incorporation in d_2_‐glycine derivatives **7 a**–**c**. The use of a triphenylmethane (trityl, Tr) protecting group yielded the best results—with alanine and d_2_‐glycine‐derived products **6 c** and **7 c** being formed in 79 % yield, 96:4 e.r. and 71 % yield, >95 % D, respectively. There are several advantages that accompany the use of a Tr protecting group: i) prevention of epimerization; ii) low cost of the TrCl precursor; iii) ease of removal with either mild acid or hydrogenolysis.^[12]^ With this modification of the protecting group, we then embarked on an evaluation of other amino acid‐derived alcohols under our optimized reaction conditions (Scheme 2).

**Scheme 2:**
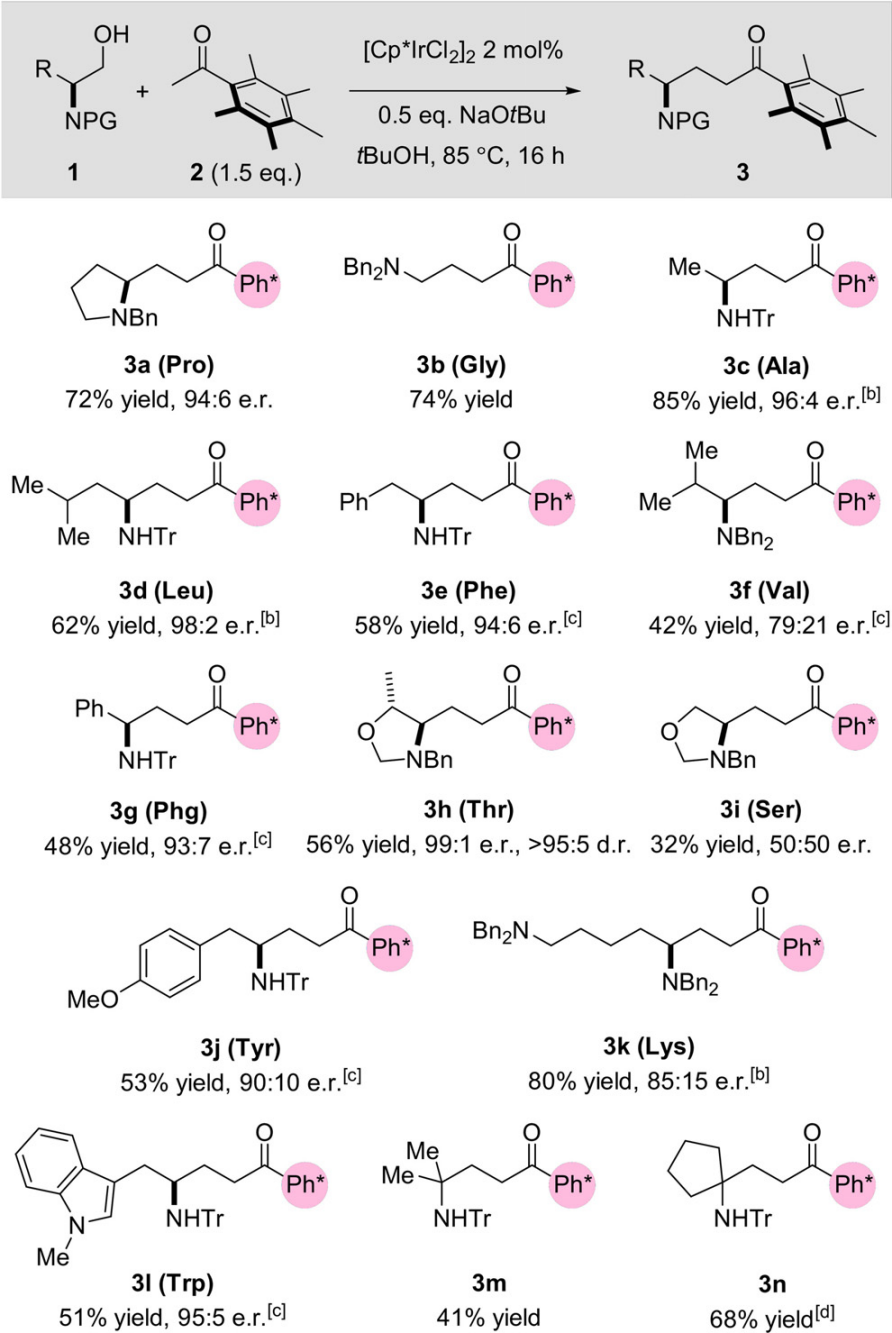
Substrate scope of the hydrogen‐borrowing reaction between 1,2‐amino alcohols and Ph* methyl ketone.^[a]^ [a] Reaction conditions: amino alcohol **1** (1.0 equiv.), **2** (1.5 equiv.), [Cp*IrCl_2_]_2_ (2 mol %), NaO*t*Bu (0.5 equiv.), *t*BuOH (2.5 M), 85 °C, 16 hours. [b] Reaction conducted on a gram scale. [c] Reaction conducted at 110 °C. [d] Reaction conducted with amino alcohol **1 n** (2.0 equiv.) and **2** (1.0 equiv.) at 110 °C.

The alcohols derived from the hydrophobic amino acids glycine, alanine, leucine, and phenylalanine were well tolerated—with good to excellent yields and high product e.r. (**3 b**, **3 c**, **3 d** and **3 e**). The reaction proved consistent upon scale‐up and pleasingly, the formation of **3 c**, **3 d** and **3 k** could be carried out on a gram scale. In contrast, valine‐derived dibenzylated product **3 f** was obtained in a lower yield of 41 % with a moderate e.r. of 79:21. We suggest that this was a result of the sterically bulky isopropyl sidechain impeding the key aldol condensation (the use of *N*‐trityl valinol succeeded only in completely shutting down reactivity). To our delight, use of an unnatural phenylglycine derivative resulted in the desired product **3 g** being obtained in moderate yield, with an admirable e.r of 93:7—this is surprising given that arylglycine derivatives have long been known to be more susceptible to racemization as a result of acidification of the α‐proton.^[13]^ Inspired by the successful reactivity of prolinol **3 a**, we decided to protect the secondary alcohol and amine in threonine as a five‐membered hemiaminal ether. Pleasingly, this manoeuver resulted in the formation of the desired product **3 h** in 56 % yield, 99:1 e.r., and as a single diastereomer. Intriguingly, when serine was protected in the same manner the isolated product **3 i** was racemic. Further investigations showed the starting cyclic alcohol derived from serine to be fluxional, racemizing with a timescale on the order of minutes (for full details, see Supporting Information).

The electron‐rich aromatic side chains of tyrosine and tryptophan were also tolerated by the reaction conditions—affording alkylated products **3 j** and **3 l** with good e.r. and acceptable yields, despite the potential for competing side reactions from the indole moiety in **3 l**. Perbenzylated lysine derivative **3 k** was isolated in excellent yield, albeit with a slightly lower e.r. of 85:15. A key advantage of hydrogen borrowing catalysis is its ability to form C−C bonds that would be difficult to achieve using conventional alkylation reactions, as demonstrated by the formation of unnatural dimethyl and cyclopentane amino acid derivatives **3 m** and **3 n** which were obtained in 41 % and 68 % yield, respectively. A traditional alkylation approach in this case would necessitate the use of sterically hindered neopentyl amino‐substituted halides.

Not all amino acid‐derived alcohols screened were reactive under our optimized conditions (Figure 1). The alcohols **8 a** and **8 b**, corresponding to the sulfur containing amino acid residues cysteine and methionine, both showed no reaction with only starting material being recovered in each case. In an attempt to probe whether coordination of the alcohol to the iridium catalyst might be responsible for the lack of reactivity, we subjected methionine derived sulfone **8 c** to the reaction conditions, again with only unreacted starting material being recovered. Glutamine derived dimethyl amide **8 d** was also unreactive, as was alcohol **8 e** derived from histidine. Finally, the free carboxylic acid **8 f** synthesized from glutamic acid also showed no reactivity, despite an extra equivalent of base being used to form the carboxylate salt in situ.

**Figure 1:**
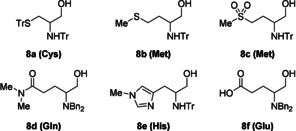
Unreactive amino alcohols.

With the substrate scope completed, our attention turned to removal of the Ph* group from the reaction products. This is typically achieved by using an electrophile such as molecular bromine to react at the *ipso*‐position of the aromatic ring, followed by retro Friedel–Crafts acylation.^[4]^ Unfortunately, initial investigations found the use of Br_2_ to be incompatible with nitrogen‐containing substrates—with oxidation of the amine functionality observed by ^1^H NMR spectroscopy of the crude reaction mixtures. Pleasingly, our recently disclosed conditions for the conversion of Ph* ketones to carboxylic acids (2 M HCl in HFIP at 65 °C) proved effective in cleaving the amino acid‐derived hydrogen‐borrowing products to give γ‐aminobutyric acids directly (Scheme 3).^[14]^

**Scheme 3:**
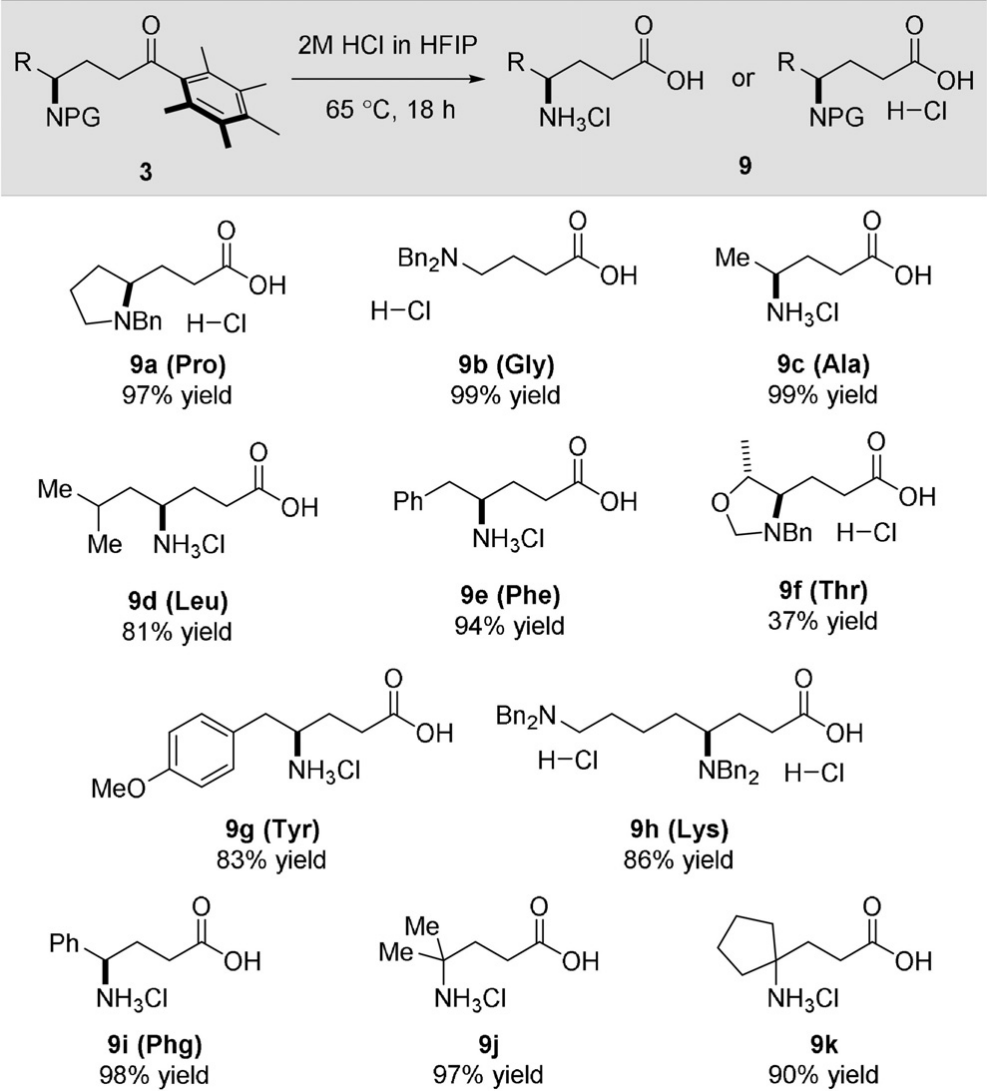
Acid‐mediated cleavage of the Ph* group to give γ‐aminobutyric acids.^[a]^ [a] Reaction conditions: hydrogen‐borrowing product **3** (1 equiv.), (aq.) HCl (conc) in HFIP (2.0 M), 65 °C, 18 hours.

In almost all cases, the acid‐mediated cleavage proceeded to give the desired products in high yields—the low yield of threonine‐derived product **9 f** being attributed to potential decomposition of the hemiaminal ether. Unfortunately, exposure of the tryptophan‐derived hydrogen borrowing product **3 l** to the cleavage conditions resulted in decomposition. Of particular interest were leucine and phenylglycine‐derived products **9 d** and **9 i**, being close positional isomers of the gabapentinoid drugs Pregabalin and Phenibut, respectively.^[15]^ A notable advantage of these cleavage conditions was that extensive purification of the resulting amino acid salts was not necessary—with only addition of water to the reaction, washing with diethyl ether to remove the liberated triphenylmethanol and pentamethylbenzene, and a final concentration of the resulting aqueous solution in vacuo needed to obtain the desired products as pure, crystalline solids.

With the scope of Ph* cleavage completed, our next concern lay in ensuring that epimerization of the amine stereocentre had not occurred under the acidic conditions used. As the amino acid hydrochloride salts were unsuitable for HPLC analysis, we chose to convert phenylglycine‐derived product **9 i** into its corresponding perbenzylated derivative. We were therefore pleased to isolate the desired product **10** in 80 % yield and with no change in e.r. of 93:7 from **3 g** (Scheme 4 A).

**Scheme 4:**
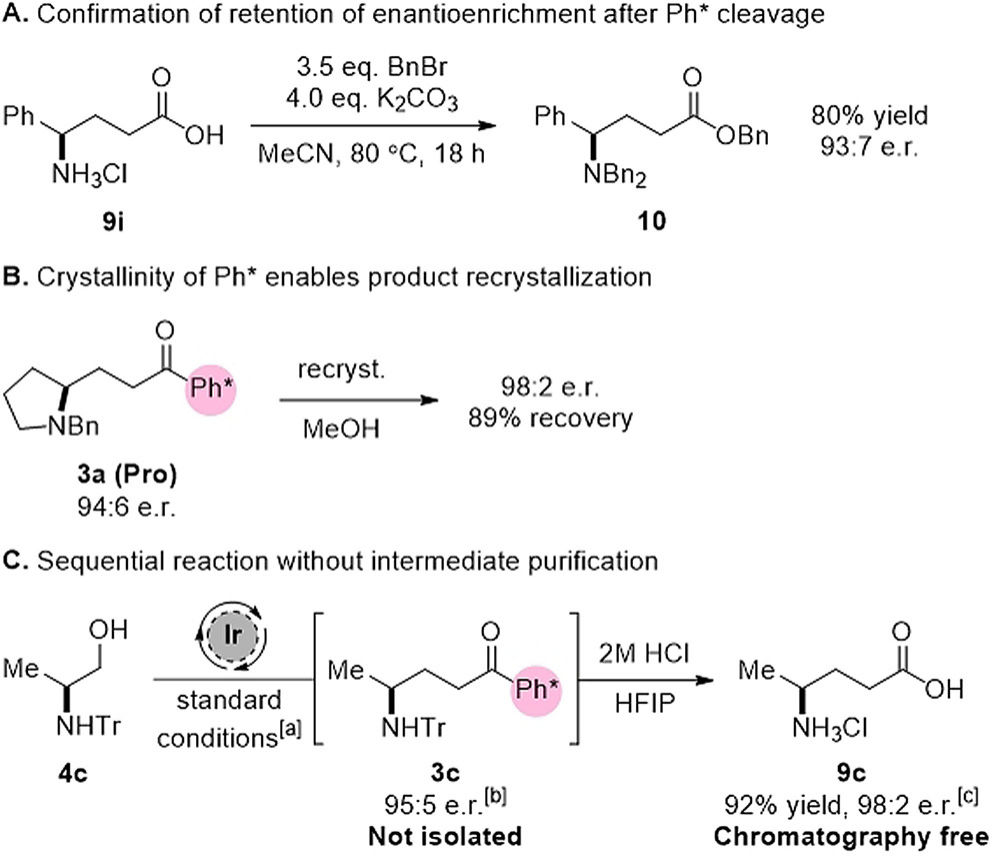
A) Determination of amino acid enantioenrichment by derivatization and HPLC analysis, B) enhancement of product enantioenrichment via recrystallization, C) chromatography‐free hydrogen‐borrowing and cleavage. [a] Reaction conditions: 4.0 mmol amino alcohol **4 c** (1.0 equiv.), **2** (1.5 equiv.), [Cp*IrCl_2_]_2_ (2 mol %), NaO*t*Bu (0.5 equiv.), *t*BuOH (2.5 M), 85 °C, 16 hours. [b] Measured by HPLC analysis of a reaction aliquot. [c] Measured by HPLC analysis of the corresponding perbenzylated derivative.

In addition to enabling the key hydrogen‐borrowing alkylation by protection of the carbonyl from competing side reactions, it has been observed that the presence of the Ph* moiety often results in the formation of crystalline products and so allows for improvement of product e.r. via recrystallization.^[5]^ We were therefore pleased to observe that our proline‐derived model substrate **3 a** could be readily recrystallized from MeOH in 98:2 e.r. and with 89 % recovery of material (Scheme 4 B).

Given that the final γ‐aminobutyric acids could be readily separated from cleavage byproducts using an aqueous extraction, we hypothesized that it might be possible to perform the two key steps in tandem and thereby avoid purification of the intermediate hydrogen‐borrowing product. Using trityl‐protected alaninol **4 c** on a 4.0 mmol scale, we were therefore delighted to isolate the desired amino acid **9 c** without the use of column chromatography in an excellent 92 % yield (571 mg) after recrystallization, with subsequent benzylation and HPLC analysis establishing a product e.r. of 98:2 (Scheme 4 C).

In conclusion, we have expanded the scope of carbon–carbon bond‐forming hydrogen‐borrowing reactions to include enantioenriched 1,2‐amino alcohols derived from cheap and abundant amino acids. This represents a substantial expansion in functional group tolerance of a class of reactions that has previously been broadly limited to simple alcohols. Furthermore, facile cleavage of the reaction products provides access to a range of enantioenriched 1,4‐amino acids in excellent yields. The use of column chromatography can be avoided altogether by performing the two key steps in sequence, with no loss in yield or enantioretention. We anticipate that these results will encourage the continued expansion of hydrogen‐borrowing catalysis to encompass a wider range of substrate functionality, as well as the use of potentially epimerizable stereogenic centres.

## Conflict of interest

The authors declare no conflict of interest.

## Supporting information

As a service to our authors and readers, this journal provides supporting information supplied by the authors. Such materials are peer reviewed and may be re‐organized for online delivery, but are not copy‐edited or typeset. Technical support issues arising from supporting information (other than missing files) should be addressed to the authors.

Supplementary
