# Supplementary material for: Hydrogen‐Borrowing Alkylation of 1,2‐Amino Alcohols in the Synthesis of Enantioenriched γ‐Aminobutyric Acids
Source: Angew Chem Int Ed Engl. 2021 Feb 24;60(13):6981–5. doi: 10.1002/anie.202100922 (PMC8048514; doi:10.1002/anie.202100922)
Supplement: Supplementary file 1 — Supplementary [file ANIE-60-6981-s001.pdf]

Angewandte Chemie
Eine Zeitschrift der Gesellschaft Deutscher Chemiker

Supporting Information

Hydrogen-Borrowing Alkylation of 1,2-Amino Alcohols in the Synthesis of Enantioenriched γ-Aminobutyric Acids

*Christopher J. J. Hall, William R. F. Goundry, and Timothy J. Donohoe**

anie_202100922_sm_miscellaneous_information.pdf

Supporting Information

Contents

**1.**	**General Information**	**S2**
**2.**	**General Procedures**	**S4**
**3.**	**Extended Optimization Table**	**S6**
**4.**	**Experimental Procedures**	**S8**
4.1.	*Synthesis of Pentamethylacetophenone* ***2***	**S8**
4.2.	*Synthesis of 1,2-Amino Alcohols*	**S9**
4.3.	*Hydrogen Borrowing Alkylations of 1,2-Amino Alcohols*	**S50**
4.4.	*Acid-Mediated Cleavage*	**S68**
4.5.	*Experiments from Scheme 4*	**S75**
**5.**	**References**	**S79**
**6.**	**NMR Spectra**	**S80**
**7.**	**HRMS Data for Deuterated Compounds**	**S170**

1. General Information

Reactions were carried out in standard glassware under an atmosphere of air unless stated otherwise. Room temperature (RT) refers to 20-25 °C. Temperatures of 0 °C were obtained using an ice/water bath. High temperatures were obtained using an oil bath equipped with a contact thermometer.

Diethyl ether, CH_2Cl_2, DMF, and tetrahydrofuran were purified by filtration through activated alumina columns employing the method of Grubbs *et al.*[1] All other solvents and reagents were used as supplied without prior purification. All other reagents were used directly as supplied by major chemical suppliers, or following purification procedures described by Perrin and Armarego.[2]

Thin layer chromatography was performed on Merck Kieselgel 60 F_{254} 0.25 mm pre-coated aluminium plates. Product spots were visualized under UV light (λ = 254 nm) and/or by staining with potassium permanganate solution. Flash chromatography was performed using VWR silica gel 60 (40-63 µm particle size) using head pressure by means of a nitrogen line.

NMR spectroscopy was carried out using Bruker 400 MHz or 500 MHz spectrometers in the deuterated solvent stated, using the residual non-deuterated solvent signal as an internal reference. Chemical shifts are quoted in ppm with signal splittings recorded as singlet (s), doublet (d), triplet (t), quartet (q), quintet (qn), sextet (sext), septet (sept), octet (oct), nonet (non) and multiplet (m). The abbreviation br denotes broad. Coupling constants, *J*, are measured to the nearest 0.1 Hz and are presented as observed.

Infrared spectra were recorded neat on a Bruker Tensor 27 spectrometer equipped with an attenuated total reflectance attachment with internal calibration. Absorption maxima (λ_{max}) are quoted in wavenumbers (cm^{-1}). The abbreviation br denotes broad.

Electrospray ionisation (ESI) HRMS were recorded on a Thermo Exactive orbitrap spectrometer equipped with a Waters Equity LC system, with a flow rate of 0.2 mL/min using water:methanol:formic acid (10:89.9:0.1) as eluent. The system uses a heated electrospray ionisation (HESI-II) probe for ESI^+ and has a resolution of 50,000 FWHM under conditions for

maximum sensitivity, with an accuracy of better than 5 ppm for 24 h following external calibration on the day of analysis. The mass reported is that containing the most abundant isotopes, with each value rounded to 4 decimal places and within 5 ppm of the calculated mass. Electron impact ionisation (EI) HRMS were performed on an Agilent 7200 quadrupole time of flight (Q-ToF) instrument equipped with a direct insertion probe supplied by Scientific Instrument Manufacturer (SIM) GmbH. Instrument control and data processing were performed using Agilent MassHunter software. The mass reported is that containing the most abundant isotopes, with each value to 4 decimal places and within 5 ppm of the calculated mass.

Optical rotations were recorded on a Schmidt Haensch Unipol L2000 polarimeter in a cell with a path length of 1 dm (using the sodium D line, 589 nm). Concentrations are reported in g/100 mL. Temperatures are reported in °C.

Chiral normal phase HPLC was performed on an Agilent 1260 Series HPLC unit equipped with UV-vis diode-array detector, fitted with the appropriate Daicel Chiralpak column (dimensions: 0.46 cm ø × 25 cm) along with the corresponding guard column (0.4 cm ø × 1 cm). Wavelengths (λ) are reported in nm, retention times (t_R) are reported in minutes and solvent flow rates are reported in mL min^{-1}.

In all cases, racemic samples for HPLC analysis were synthesised from the corresponding racemic amino acids using an identical synthetic route to that used to prepare enantioenriched material.

2. General Procedures

General Procedure A: Hydrogen Borrowing Alkylation of 1,2-Amino Alcohols

Under an air atmosphere, a 2–5 mL Biotage® microwave vial equipped with a stirrer bar was sequentially charged with the appropriate amino alcohol (1.0 eq.), pentamethylacetophenone **2** (1.5 eq.), $[Cp^*IrCl_2]_2$ (2 mol%), NaO^tBu (0.5 eq.), and *tert*-butanol (0.4 mL/mmol amino alcohol). The reaction vessel was sealed with a microwave vial cap (containing a Reseal™ septum), pierced with a needle attached to a nitrogen balloon, and heated to 85 or 110 ^oC in a preheated oil bath for 16 h. The mixture was cooled to RT and filtered through a SiO_2 plug (eluting with Et_2O). The resulting solution was then concentrated and purified by column chromatography (see experimental methods section for details).

General Procedure B: Acid Mediated Cleavage of Hydrogen Borrowing Products

A 2–5 mL Biotage® microwave vial equipped with a stirrer bar was sequentially charged with the appropriate hydrogen borrowing product (1.0 eq.), HFIP (8.8 mL/mmol) and conc. HCl (1.3 mL/mmol). The reaction vessel was sealed with a microwave vial cap (containing a Reseal™ septum) and heated at 65 °C for 16 h. The reaction was then cooled to RT and diluted by the addition of water. The mixture was washed with Et_2O and concentrated *in vacuo* to give the pure desired product as a hydrochloride salt.

General Procedure C: Formation of Amino Acid Methyl Ester Hydrochlorides

To a stirred solution of amino acid (1.0 eq.) in MeOH (10 mL/g amino acid) at 0 °C was added $SOCl_2$ (1.5 eq.) dropwise. The reaction was then warmed to RT and left to stir overnight. The solvent was then removed *in vacuo* to give a crude solid that was redissolved in MeOH (5 mL/g amino acid) before being again concentrated *in vacuo*. This process was repeated twice before a final concentration *in vacuo* to give the pure desired product.

General Procedure D: Tritylation of Amino Acid Esters

To a stirred solution of amino acid ester hydrochloride (1.0 eq.) and trityl chloride (1.0 eq.) in CH_2Cl_2 (3 mL/mmol amino acid) at RT was added Et_3N (2.0 eq.) dropwise and the reaction left to stir for 16

h. The reaction was then washed with water (3 mL/mmol amino acid), dried over Na_2SO_4 and concentrated *in vacuo* to give the crude product.

General Procedure E: $LiAlH_4$ Reduction of Amino Acids and Esters

To a stirred solution of $LiAlH_4$ (2.0 eq.) in THF (10 mL/mmol $LiAlH_4$) at 0 °C was added a solution of the amino acid ester (1.0 eq.) in THF (1 mL/mmol $LiAlH_4$) dropwise, before being warmed to RT for esters or reflux for carboxylic acids. Reaction progress was monitored by TLC analysis and upon completion (typically 15 min for methyl esters, 3 h for benzyl esters, and 16 h for carboxylic acids), the reaction was cooled to 0 °C and diluted with Et_2O (5 mL/mmol $LiAlH_4$) before being quenched by the sequential addition of water (1 mL/g $LiAlH_4$), 15% aqueous NaOH (1 mL/g $LiAlH_4$), water (3 mL/g $LiAlH_4$) and $MgSO_4$. The thick emulsion was then filtered under reduced pressure (washing the filter cake with Et_2O), and concentrated *in vacuo* to give the crude product.

3. Extended Optimization Table

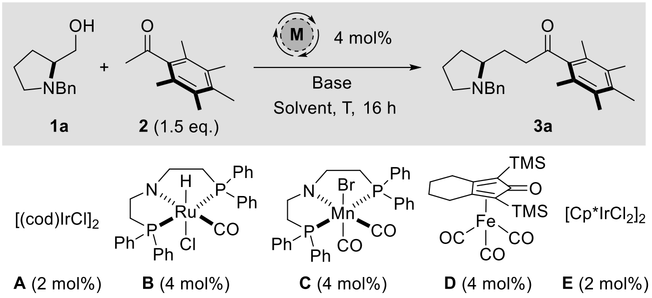

Under an air atmosphere, a 2–5 mL Biotage® microwave vial equipped with a stirrer bar, was sequentially charged with (*S*)-*N*-benzyl-*L*-prolinol **1a** (76 mg, 0.40 mmol), pentamethylacetophenone **2** (114 mg, 0.60 mmol), metal catalyst, base, and solvent. The reaction vessel was sealed with a microwave vial cap (containing a Reseal™ septum) and the vial was heated to *the indicated temperature* in a preheated oil bath for 16 h. The mixture was cooled to RT and filtered through a SiO_2 plug (eluting with Et_2O) and the filtrate was concentrated under reduced pressure. Purification by column chromatography (SiO_2, CH_2Cl_2:MeOH, 95:5) afforded the title compound **3a** as a tan solid and the enantioselectivity was determined by normal phase HPLC analysis (Chiralpak IA column with guard, 1 % IPA, 99 % hexane, 1.0 mL/min, 25 °C, λ = 254 nm, 10 µL injection).

Entry	[M]	Base (equiv.)	T/ °C	Solvent	Yield[a] 3a	e.r.[b]
1	**A**[c]	KOH (2.0)	85	PhMe (1.0 M)	20	77:23
2	**B**	KOH (2.0)	85	PhMe (1.0 M)	41	81:19
3	**C**	KOH (2.0)	85	PhMe (1.0 M)	–[d]	–[d]
4	**D**[e]	KOH (2.0)	85	PhMe (1.0 M)	NR	NR
5	**E**	KOH (2.0)	85	PhMe (1.0 M)	51	82:18
6	**E**	KOH (2.0)	85	No solvent	63	82:18
7	**E**	KOH (2.0)	110	No solvent	62	65:35
8	**E**	KOH (2.0)	65	No solvent	49	88:12
9	**E**	KO*t*Bu (2.0)	85	No solvent	18	90:10
10	**E**	NaO*t*Bu (2.0)	85	No solvent	10	96:4
11	**E**	NaO*t*Bu (1.0)	85	No solvent	42	95:5

12	**E**	NaO*t*Bu (0.5)	85	No solvent	63	92:8
13	**E**	NaO*t*Bu (0.5)	85	*t*BuOH (0.5 M)	50	92:8
14	**E**	NaO*t*Bu (0.5)	85	*t*BuOH (1.0 M)	46	95:5
**15**	**E**	**NaO*t*Bu (0.5)**	**85**	***t*BuOH (2.5M)**	**72**	**94:6**
16	**E**	NaOH (2.0)	85	No solvent	55	95:5
17	**E**	NaO*t*Bu (0.2)	85	No solvent	60	89:11
18	**E**	NaO*t*Bu (1.0)	85	*t*BuOH (0.5 M)	39	94:6
19	**E**	NaO*t*Bu (2.0)	85	PhMe (0.5 M)	25	98:2
20	**E**	NaOH (0.5)	85	No solvent	63	90:10
21	**E**	LiOH (0.5)	85	No solvent	NR	NR
22	**E**	Cs_2CO_3 (0.5)	85	No solvent	NR	NR
23	**E**	NaOH (0.5)	85	*t*BuOH (0.5 M)	38	87:13

[a] Isolated yield. [b] Determined by normal phase HPLC analysis using a chiral stationary phase. [c] With 4 mol% dppBz. [d] Complex mixture formed. [e] With 8 mol% Me_3NO.

cod = 1,5-cyclooctadiene; dppBz = 1,2-Bis(diphenylphosphino)benzene; TMS = trimethylsilyl; Cp* = pentamethylcyclopentadienyl; NR = No reaction

4. *Experimental Procedures*

4.1 *Synthesis of Pentamethylacetophenone 2*

1-(2,3,4,5,6-Pentamethylphenyl)ethan-1-one, 2

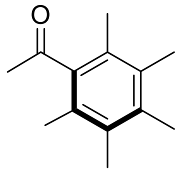

To a stirred solution of pentamethylbenzene (10.0 g, 67.6 mmol, 1.0 eq.) and acetyl bromide (5.48 mL, 74.3 mmol, 1.1 eq.) in CH_2Cl_2 (675 mL) at 0 °C was added $AlCl_3$ (11.25 g, 84.5 mmol, 1.25 eq.) portionwise. After stirring for 30 min, the reaction was poured into water (cooled with a water-ice bath) and the layers were separated. The aqueous layer was extracted with CH_2Cl_2 and the combined organic layers washed with sat. $NaHCO_3$, dried over $MgSO_4$ and concentrated *in vacuo*. Purification by column chromatography (SiO_2, dry load, pentane:Et_2O, 90:10) gave the desired compound **2** as a white solid (12.0 g, 93%) that may be recrystallised from hexane.

The spectral data matched that previously reported in the literature.[3]

^{1}H NMR ($CDCl_3$, 400 MHz) δ = 2.46 (s, 3H), 2.24 (s, 3H), 2.19 (s, 6H), 2.14 (s, 6H).

^{13}C NMR ($CDCl_3$, 101 MHz) δ = 210.2, 141.1, 135.5, 133.2 (2C), 127.1 (2C), 33.3, 17.2 (2C), 16.8, 16.1 (2C).

4.2 *Synthesis of 1,2-Amino Alcohols*

Alcohol **1a** was purchased from Sigma Aldrich and used as received. The majority of other amino alcohols were synthesised by protection of either the free amino acid or amino acid methyl ester hydrochloride, followed by reduction with $LiAlH_4$ to give the desired product. Experimental details and full characterization are provided below.

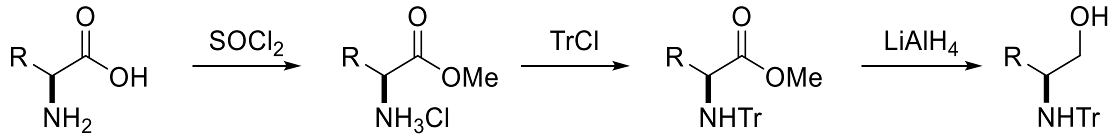

(*S*)-2-(Isoindolin-2-yl)propan-1-ol, 4a

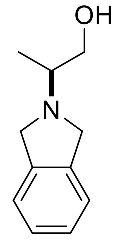

To a stirred solution of *L*-alaninol (0.75 mg, 10 mmol, 1.0 eq.) and α,α'-dibromo-*o*-xylene (2.64 g, 10 mmol, 1.0 eq.) in dioxane (25 mL) at RT was added NaOH (0.96 g, 24 mmol, 2.4 eq.) and the reaction left to stir for 1 h. The reaction mixture was then filtered through a pad of celite (eluting with Et_2O) and concentrated *in vacuo*. Purification by column chromatography (SiO_2, eluent load, CH_2Cl_2:MeOH, 85:15) gave the desired compound **4a** (319 mg, 18%, >99:1 e.r.) as a fluffy, pale-yellow solid.

m.p. = 123–125 °C.

IR (film) ν_{max}/cm^{-1} = 2979, 2804, 1462, 1378, 1069, 1048, 977, 755.

^{1}H NMR ($CDCl_3$, 400 MHz) δ = 7.22 (s, 4H), 4.09 – 3.95 (m, 4H), 3.69 (dd, *J* = 10.8, 4.3 Hz, 1H), 3.48 (dd, *J* = 10.8, 6.2 Hz, 1H), 2.97 (pd, *J* = 6.5, 4.3 Hz, 1H), 1.15 (d, *J* = 6.6 Hz, 3H).

^{13}C NMR ($CDCl_3$, 101 MHz) δ = 139.7 (2C), 126.9 (2C), 122.5 (2C), 64.7, 58.4, 55.1 (2C), 13.3.

HRMS (ESI$^+$) Found $[M+H]^+$ = 178.1227; $C_{11}H_{16}ON$ requires 178.1226, Δ 0.14 ppm.

$[\alpha]_D^{25}$ = +51.0 (c = 1.00, $CHCl_3$).

Chiral HPLC (Chiralpak IB-N5 with guard, 0.9 % IPA, 99.1 % hexane, 1.0 mL/min, 25 °C, λ = 210 nm, 5 μL injection).

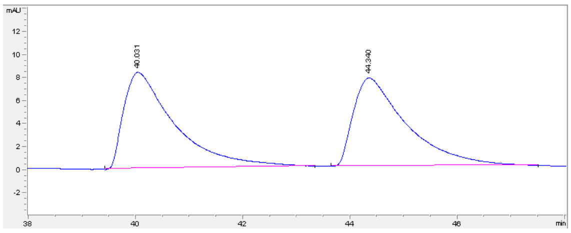

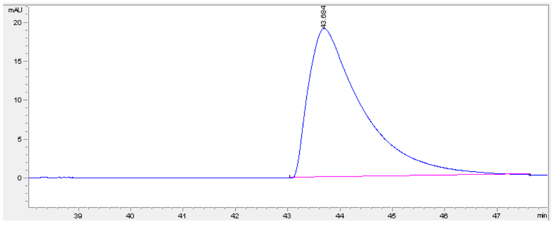

#	Time	Type	Area	Height	Width	Area%	Symmetry
1	40.031	BB	563.7	8.4	0.9028	51.476	0.348
2	44.34	BB	531.4	7.7	0.9236	48.524	0.383

#	Time	Type	Area	Height	Width	Area%	Symmetry
1	43.684	BB	1396.5	19.3	1.0026	100.000	0.365

Benzyl dibenzyl-*L*-alaninate, S1

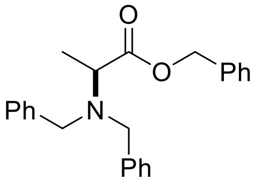

To a stirred suspension of *L*-alanine (0.89 g, 10 mmol, 1.0 eq.) and K_2CO_3 (4.14 g, 30 mmol, 3.0 eq.) in MeCN (30 mL) at RT was added benzyl bromide (4.16 mL, 35 mmol, 3.0 eq.) dropwise. The resulting solution was then warmed to 80 °C and left to stir for 16 h. The reaction mixture was then cooled to RT and water (50 mL) was added. The resulting mixture was then extracted with EtOAc (3 × 50 mL), and the organic extracts washed with brine (50 mL), dried over Na_2SO_4, and concentrated *in vacuo*. Purification by column chromatography (SiO_2, eluent load, pentane:Et_2O, 96:4) gave the desired compound **S1** (585 mg, 16%) as a colourless oil.

The spectral data matched that previously reported in the literature.[4]

^{1}H NMR ($CDCl_3$, 400 MHz) δ = 7.43 – 7.20 (m, 15H), 5.28 – 5.12 (m, 2H), 3.88 – 3.79 (m, 2H), 3.68 – 3.60 (m, 2H), 3.56 (qd, *J* = 5.9, 2.4 Hz, 1H), 1.39 – 1.33 (m, 3H).

^{13}C NMR ($CDCl_3$, 101 MHz) δ = 173.7, 140.0 (2C), 136.3, 128.8 (4C), 128.7 (2C), 128.44 (2C), 128.38, 128.35 (4C), 127.1 (2C), 66.2, 56.3, 54.5 (2C), 15.1.

(*S*)-2-(Dibenzylamino)propan-1-ol, 4b

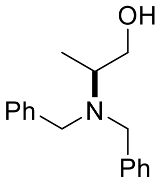

Perbenzylated alanine **S1** (528 mg, 1.5 mmol, 1.0 eq.) was subjected to **General Procedure E**. Purification by column chromatography (SiO_2, eluent load, pentane:Et_2O, 80:20 to 70:30) gave the desired alcohol **4b** (285 mg, 75%, >99:1 e.r.) as a colourless oil.

The spectral data matched that previously reported in the literature.[5]

^{1}H NMR ($CDCl_3$, 400 MHz) δ = 7.39 – 7.22 (m, 10H), 3.83 (d, *J* = 13.3 Hz, 2H), 3.45 (q, *J* = 13.6 Hz, 1H), 3.40 – 3.30 (m, 3H), 3.07 – 2.93 (m, 1H), 0.99 (d, *J* = 6.6 Hz, 3H).

^{13}C NMR ($CDCl_3$, 101 MHz) δ = 139.4 (2C), 129.1 (4C), 128.6 (4C), 127.3 (2C), 62.9, 54.3, 53.1 (2C), 8.8.

$[\alpha]_D^{25}$ = +51.0 (c = 1.00, $CHCl_3$).

Chiral HPLC (Chiralpak IB-N5 with guard, 5.0 % IPA, 95.0 % hexane, 1.0 mL/min, 25 °C, λ = 254 nm, 10 µL injection).

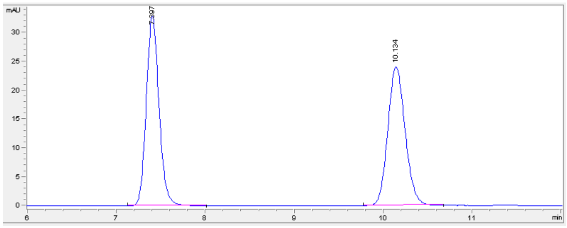

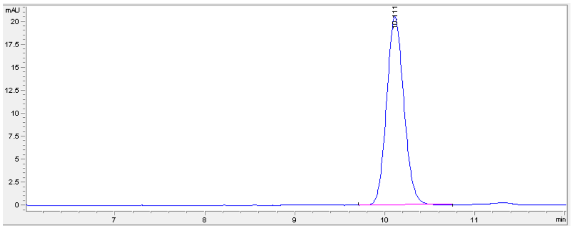

#	Time	Type	Area	Height	Width	Area%	Symmetry
1	7.397	BB	325.2	32.8	0.1535	49.960	0.852
2	10.134	BB	325.7	24	0.2101	50.040	0.861

#	Time	Type	Area	Height	Width	Area%	Symmetry
1	10.111	BB	275.5	20.5	0.2065	100.000	0.873

(*S*)-2-(Tritylamino)propan-1-ol, 4c

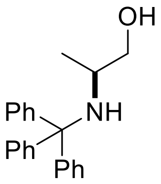

L-Alanine (3.56 g, 40 mmol, 1.0 eq.) was subjected to **General Procedure C** to give the desired amino

acid methyl ester hydrochloride (5.24 g, 94%). This ester (4.86 g, 35 mmol, 1.0 eq.) was then subjected to **General Procedure D** to give the crude tritylated alanine methyl ester which was immediately subjected to **General Procedure E**. Purification by column chromatography (SiO_2, eluent load, pentane:Et_2O, 70:30 to 50:50) gave the desired alcohol **4c** as a colourless solid (7.26 g, 65% over two steps, >99:1 e.r.).

m.p. = 82–84 °C.

IR (film) ν_{max}/cm^{-1} = 2968, 1448, 1032, 950, 745, 706.

^{1}H NMR ($CDCl_3$, 400 MHz) δ = 7.61 – 7.52 (m, 6H), 7.33 – 7.25 (m, 6H), 7.24 – 7.18 (m, 3H), 3.18 (dd, *J* = 10.5, 4.3 Hz, 1H), 3.06 (dd, *J* = 10.5, 4.8 Hz, 1H), 2.78 (qt, *J* = 6.5, 4.5 Hz, 1H), 0.68 (d, *J* = 6.5 Hz, 3H).

^{13}C NMR ($CDCl_3$, 101 MHz) δ = 146.9 (3C), 128.9 (6C), 128.0 (6C), 126.6 (3C), 71.3, 67.1, 49.6, 19.9.

HRMS (ESI$^+$) Found $[M+Na]^+$ = 340.1671; $C_{22}H_{23}ONNa$ requires 340.1672, Δ –0.31 ppm.

$[\alpha]_D^{25}$ = +10.9 (c = 1.00, $CHCl_3$).

Chiral HPLC (Chiralpak IA with guard, 5.0 % IPA, 95.0 % hexane, 1.0 mL/min, 25 °C, λ = 254 nm, 10 µL injection).

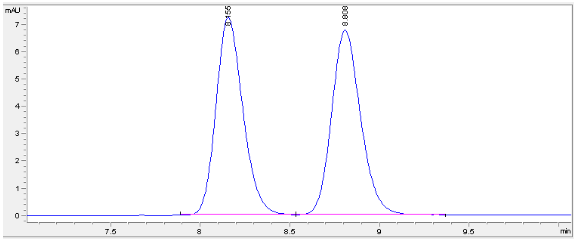

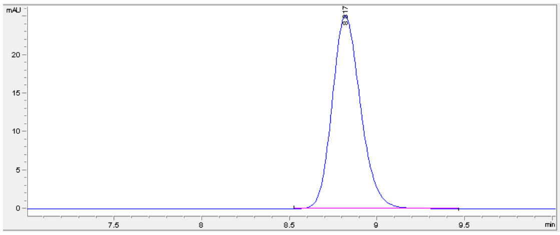

#	Time	Type	Area	Height	Width	Area%	Symmetry
1	8.155	BB	76	7.3	0.1602	49.912	0.802
2	8.808	BB	76.2	6.8	0.1734	50.088	0.813

#	Time	Type	Area	Height	Width	Area%	Symmetry
1	8.817	BB	282.3	25	0.1716	100.000	0.809

Methyl glycinate-2,2-*d*$_2$ hydrochloride, S2

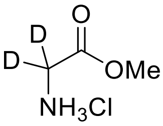

Glycine-*d*$_5$ (2.0 g, 25 mmol, 1.0 eq.) was subjected to **General Procedure C** to give the desired

compound **S2** (3.0 g, 95%) as a colourless solid with >95% D incorporation, as determined by ^{1}H NMR analysis.

m.p. = 182–184 °C.

IR (film) ν_{max}/cm^{-1} = 2907, 1743, 1499, 1329, 1209, 1150, 910, 792.

^{1}H NMR (D_2O, 400 MHz) δ = 3.86 (s, 3H).

^{13}C NMR (D_2O, 101 MHz) δ = 168.7, 53.4, 39.6.

^{2}H NMR (H_2O, 77 MHz) δ = 3.80.

HRMS (ESI^{+}) Found [M+H]$^{+}$ = 92.0679; $C_3H_6D_2O_2N$ requires 92.0675, Δ 4.19 ppm.

Methyl 2-(isoindolin-2-yl)acetate-*d*$_2$, S3

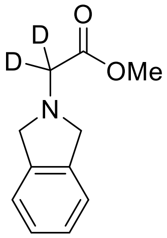

A stirred solution of **S2** (635 mg, 5 mmol, 1.0 eq.), α,α'-dibromo-*o*-xylene (1.32 g, 5 mmol, 1.0 eq.) and K_2CO_3 (2.07 g, 15 mmol, 3.0 eq.) in MeCN (100 mL) was warmed to 80 °C and the reaction left to stir for 24 h. The reaction was then quenched by the addition of water (100 mL) and the mixture extracted with EtOAc (3 × 75 mL). The combined organic extracts were then washed with brine (100 mL), dried over Na_2SO_4, and concentrated *in vacuo* to give the crude product. Purification by column chromatography (SiO_2, eluent load, pentane:Et_2O, 50:50) gave the desired compound **S3** (568 mg, 59%) as a brown oil with 94% D incorporation, as determined by ^{1}H NMR analysis.

IR (film) ν_{max}/cm^{-1} = 1741, 1466, 1434, 1285, 1265, 1202, 1168, 745.

^{1}H NMR ($CDCl_3$, 400 MHz) δ = 7.19 (s, 4H), 4.12 (s, 4H), 3.75 (s, 3H), 3.60 (t, *J* = 2.4 Hz, 0.12H).

^{13}C NMR ($CDCl_3$, 101 MHz) δ = 171.3, 139.8 (2C), 126.9 (2C), 122.4 (2C), 58.8 (2C), 51.8. [*N.B.* The signal for the CD_2 carbon is a very weak multiplet at ~55.8 ppm].

^{2}H NMR ($CHCl_3$, 77 MHz) δ = 3.60.

HRMS (ESI$^+$) Found $[M+H]^+$ = 194.1146; $C_{11}H_{12}D_2O_2N$ requires 194.1145, Δ 0.78 ppm.

2-(Isoindolin-2-yl)ethan-2,2-*d*$_2$-1-ol, 5a

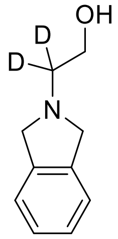

Methyl ester **S3** (483 mg, 2.5 mmol, 1.0 eq.) was subjected to **General Procedure E**. Purification by column chromatography (SiO_2, eluent load, CH_2Cl_2:MeOH, 92:8) gave the desired alcohol **5a** (342 mg, 83%) as a brown oil with 90% D incorporation, as determined by ^{1}H NMR analysis.

IR (film) ν_{max}/cm^{-1} = 3289 (br.), 2778, 1464, 1056, 1028, 741.

^{1}H NMR ($CDCl_3$, 400 MHz) δ = 7.21 (s, 4H), 4.00 (s, 4H), 3.70 (s, 2H), 2.95 – 2.87 (m, 0.2H).

^{13}C NMR ($CDCl_3$, 101 MHz) δ = 139.8 (2C), 127.0 (2C), 122.4 (2C), 59.8, 59.0 (2C), 57.0.

^{2}H NMR ($CHCl_3$, 77 MHz) δ = 2.91.

HRMS (ESI$^+$) Found $[M+H]^+$ = 166.1194; $C_{10}H_{12}D_2ON$ requires 166.1195, Δ –0.70 ppm.

Methyl *N*,*N*-dibenzylglycinate-*d*$_2$, S4

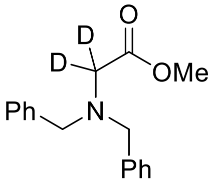

A stirred solution of **S2** (635 mg, 5 mmol, 1.0 eq.) and K_2CO_3 (1.73 g, 12.5 mmol, 2.5 eq.) in MeCN (100 mL) was added benzyl bromide (1.19 mL, 10 mmol, 2.0 eq.) dropwise and the reaction left to stir for 18 h. The reaction was filtered through a pad of celite (eluting with Et_2O) and concentrated *in vacuo* to give the desired pure product **S4** (1.35 g, 99%) as a colourless solid with >95% D incorporation, as determined by ^{1}H NMR analysis.

m.p. = 31–33 °C.

IR (film) ν_{max}/cm^{-1} = 1743, 1453, 1275, 1243, 1156, 1143, 1064, 746, 735, 696.

^{1}H NMR ($CDCl_3$, 400 MHz) δ = 7.42 – 7.36 (m, 4H), 7.35 – 7.29 (m, 4H), 7.28 – 7.23 (m, 2H), 3.82 (s, 4H), 3.69 (s, 3H).

^{13}C NMR ($CDCl_3$, 101 MHz) δ = 172.0, 139.1 (2C), 129.0 (4C), 128.4 (4C), 127.3 (2C), 57.8, 51.4. [*N.B.* The signal for the CD_2 carbon is too weak to observe].

^{2}H NMR ($CHCl_3$, 77 MHz) δ = 2.91.

HRMS (ESI^+) Found $[M+H]^+$ = 272.1614; $C_{17}H_{18}D_2O_2N$ requires 272.1614, Δ –0.20 ppm.

2-(Dibenzylamino)ethan-2,2-*d*$_2$-1-ol, 5b

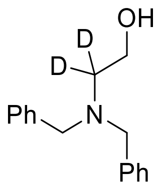

Methyl ester **S4** (813 mg, 3.0 mmol, 1.0 eq.) was subjected to **General Procedure E**. Purification by column chromatography (SiO_2, eluent load, pentane:Et_2O, 60:40) gave the desired alcohol **5b** (631 mg, 87%) as a colourless solid with >95% D incorporation, as determined by ^{1}H NMR analysis.

m.p. = 39–41 °C.

IR (film) ν_{max}/cm^{-1} = 3300, 2796, 1494, 1453 1369, 1058, 1025, 744, 731, 697.

^{1}H NMR ($CDCl_3$, 400 MHz) δ = 7.42 – 7.22 (m, 10H), 3.64 (s, 4H), 3.58 (s, 2H).

^{13}C NMR ($CDCl_3$, 101 MHz) δ = 138.9 (2C), 129.1 (4C), 128.6 (4C), 127.4 (2C), 58.5, 58.3, 54.1.

^{2}H NMR ($CHCl_3$, 77 MHz) δ = 2.65.

HRMS (ESI^+) Found $[M+H]^+$ = 244.1666; $C_{16}H_{18}D_2ON$ requires 244.1665, Δ 0.23 ppm.

Methyl tritylglycinate-2,2-d_2, S5

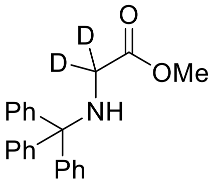

To a stirred solution of methyl ester hydrochloride **S2** (635 mg, 5 mmol, 1.0 eq.) and trityl chloride (1.45 g, 5.2 mmol, 1.04 eq.) in DMF (10 mL) at 0 °C was added Et_3N (1.73 mL, 12.5 mmol, 2.5 eq.) dropwise before being warmed to RT and left to stir for 16 h. The reaction was quenched by the addition of water (20 mL) and the mixture extracted with EtOAc:Hexane (2:1, 3 × 25 mL). The combined organic extracts were then washed with brine (3 × 25 mL), dried over Na_2SO_4, and concentrated *in vacuo*. Purification by column chromatography (SiO_2, eluent load, pentane:Et_2O, 90:20) gave the desired pure product **S5** (1.63 g, 98%) as a colourless solid with >95% D incorporation, as determined by ^{1}H NMR analysis.

m.p. = 99–101 °C.

IR (film) ν_{max}/cm^{-1} = 1739, 1695, 1489, 1447, 1286, 1154, 770, 746, 698, 624.

^{1}H NMR ($CDCl_3$, 400 MHz) δ = 7.52 – 7.42 (m, 6H), 7.30 – 7.24 (m, 6H), 7.21 – 7.16 (m, 3H), 3.60 (s, 3H).

^{13}C NMR ($CDCl_3$, 101 MHz) δ = 172.9, 145.5 (3C), 128.8 (6C), 128.1 (6C), 126.7 (3C), 70.8, 51.9. [*N.B.* The signal for the CD_2 carbon is too weak to observe].

^{2}H NMR ($CHCl_3$, 77 MHz) δ = 3.14.

HRMS (ESI$^+$) Found [M+Na]$^+$ = 356.1589; $C_{22}H_{19}D_2O_2NNa$ requires 356.1590, Δ –0.40 ppm.

2-(Tritylamino)ethan-2,2-*d*$_2$-1-ol, 5c

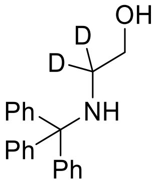

Methyl ester **S5** (999 mg, 3.0 mmol, 1.0 eq.) was subjected to **General Procedure E**. Purification by column chromatography (SiO_2, eluent load, pentane:Et_2O, 60:40) gave the desired alcohol **5c** (804 mg, 88%) as a colourless solid with >95% D incorporation, as determined by ^{1}H NMR analysis.

m.p. = 74–76 °C.

IR (film) ν_{max}/cm^{-1} = 3272 (br.), 1487, 1447, 1047, 1027, 760, 748, 700, 639.

^{1}H NMR ($CDCl_3$, 400 MHz) δ = 7.52 – 7.44 (m, 6H), 7.34 – 7.26 (m, 6H), 7.23 – 7.17 (m, 3H), 3.68 (s, 2H).

^{13}C NMR ($CDCl_3$ 101 MHz) δ = 146.0 (3C), 128.8 (6C), 128.0 (6C), 126.5 (3C), 70.7, 62.7, 45.1.

^{2}H NMR ($CHCl_3$, 77 MHz) δ = 2.34.

HRMS (ESI$^+$) Found $[M+Na]^+$ = 328.1641; $C_{21}H_{19}D_2ONNa$ requires 328.1641, Δ –0.08 ppm.

2-(Dibenzylamino)ethan-1-ol, 1b

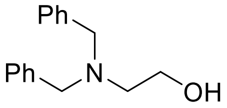

Ethanolamine (1.50 mL, 25 mmol, 1.0 eq.), benzyl bromide (6.30 mL, 53 mmol, 2.1 eq.) and K_2CO_3 (6.9 g, 50 mmol, 2.0 eq.) were added to acetone (50 mL) and stirred for 16 h at RT. The reaction mixture was then filtered, and the solvent removed *in vacuo*. The crude product was then passed through a short plug of SiO_2 (eluting with pentane:Et_2O, 70:30 to 40:60) and the solvent removed *in vacuo* to give an oil which crystallised over 3 d to give pure **1b** (2.29 g, 38%) as a colourless solid.

The spectral data matched that previously reported in the literature.[6]

^{1}H NMR ($CDCl_3$, 400 MHz) δ = 7.39 – 7.22 (m, 10H), 3.63 (s, 4H), 3.58 (t, *J* = 5.8 Hz, 2H), 2.68 (t, *J* = 5.8 Hz, 2H), 2.61 (br. s, 1H, *OH*).

^{13}C NMR ($CDCl_3$, 101 MHz) δ = 138.9 (2C), 129.1 (4C), 128.6 (4C), 127.4 (2C), 58.6, 58.3, 54.9.

(*S*)-4-Methyl-2-(tritylamino)pentan-1-ol, 1d

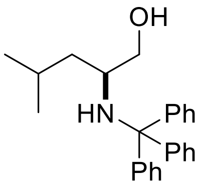

To a stirred solution of commercially available *L*-leucinol (2.60 g, 20 mmol, 1.0 eq.) and trityl chloride (5.58 g, 20 mmol, 1.0 eq.) in CH_2Cl_2 (50 mL) at 0 °C was added Et_3N (2.77 mL, 20 mmol, 1.0 eq.) dropwise over the course of 15 min. The mixture was then warmed to RT and left to stir for 24 h. The reaction was then quenched by the addition of sat. aq. $NaHCO_3$ (50 mL) and the layers were separated. The aqueous layer was extracted with CH_2Cl_2 (3 × 30 mL) and the combined organic extracts washed with brine (30 mL), dried over Na_2SO_4 and concentrated *in vacuo*. Purification by column chromatography (SiO_2, eluent load, pentane:Et_2O, 80:20) gave the desired alcohol **1d** (3.68 g, 51%, >99:1 e.r.) as a thick and colourless oil.

The spectral data matched that previously reported in the literature.[7]

^{1}H NMR ($CDCl_3$, 400 MHz) δ = 7.59 – 7.51 (m, 6H), 7.33 – 7.25 (m, 6H), 7.23 – 7.18 (m, 3H), 3.23 (dd, *J* = 10.9, 2.6 Hz, 1H), 3.06 (ddd, *J* = 10.9, 3.9, 0.8 Hz, 1H), 2.66 (dtd, *J* = 10.2, 3.9, 2.6 Hz, 1H), 1.46 – 1.32 (m, 2H), 0.68 (d, *J* = 6.4 Hz, 3H), 0.66 – 0.60 (m, 1H), 0.58 (d, *J* = 6.4 Hz, 3H).

^{13}C NMR ($CDCl_3$, 101 MHz) δ = 146.7 (3C), 128.9 (6C), 128.0 (6C), 126.6 (3C), 71.5, 62.9, 51.7, 42.8, 24.9, 23.8, 21.6.

$[\alpha]_D^{25}$ = +20.8 (c = 1.00, $CHCl_3$).

Chiral HPLC (Chiralpak IA with guard, 5.0 % IPA, 95.0 % hexane, 1.0 mL/min, 25 °C, λ = 254 nm, 10 µL injection).

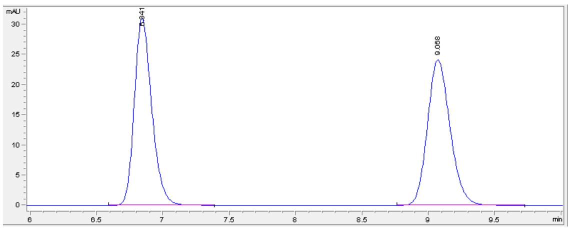

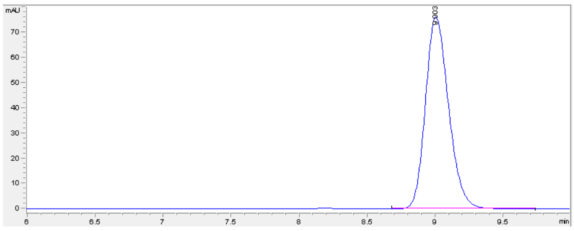

#	Time	Type	Area	Height	Width	Area%	Symmetry
1	6.841	BB	285.4	31.2	0.1387	49.792	0.761
2	9.068	BB	287.8	24.3	0.1824	50.208	0.819

#	Time	Type	Area	Height	Width	Area%	Symmetry
1	9.003	BB	901.9	76.3	0.182	100.000	0.774

(*S*)-3-Phenyl-2-(tritylamino)propan-1-ol, 1e

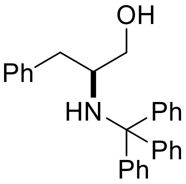

The initial tritylation was performed using the method reported by Zervas and Theodoropoulos.[8] To a stirred solution of *L*-phenylalanine (3.30 g, 20 mmol, 1.0 eq.) in $CHCl_3$:MeCN (30:6 mL) at RT was added Me_3SiCl (2.53 mL, 20 mmol, 1.0 eq.) and the reaction heated to reflux for 2 h before being allowed to cool back to RT. Et_3N (5.53 mL, 40 mmol, 2.0 eq.) was slowly added, followed by trityl chloride (5.58 g, 20 mmol, 1.0 eq.) in $CHCl_3$ (20 mL) and the reaction left to stir for 1 h. The reaction mixture was then quenched by the addition of MeOH (4 mL) and the solvent was removed *in vacuo* to give a residue which was redissolved in Et_2O (50 mL) and water (50 mL). The layers were separated, and the organic layer sequentially washed with a 5% precooled aq. solution of citric acid (2 × 30 mL) and brine (30 mL), dried over Na_2SO_4, and concentrated *in vacuo* to give the crude tritylated product which was used for the next step without further purification. The crude tritylated phenylalanine (8.14 g) was subjected to **General Procedure E**. Purification by column chromatography (SiO_2, eluent load, pentane:Et_2O, 75:25) gave the desired alcohol **1e** (4.59 g, 58%, 99:1 e.r.) as a colourless solid.

The spectral data matched that previously reported in the literature.[7]

^{1}H NMR ($CDCl_3$, 400 MHz) δ = 7.64 – 7.55 (m, 6H), 7.36 – 7.27 (m, 6H), 7.27 – 7.11 (m, 6H), 7.00 – 6.92 (m, 2H), 3.15 (dd, *J* = 10.9, 2.8 Hz, 1H), 2.96 (dd, *J* = 10.9, 4.0 Hz, 1H), 2.85 (dtd, *J* = 9.3, 4.6, 2.8 Hz, 1H), 2.55 (dd, *J* = 13.1, 9.3 Hz, 1H), 2.32 (dd, *J* = 13.1, 4.6 Hz, 1H).

^{13}C NMR ($CDCl_3$, 101 MHz) δ = 146.7 (3C), 139.2, 129.6 (2C), 128.9 (6C), 128.4 (2C), 128.1 (6C), 126.7 (3C), 126.2, 71.5, 62.6, 55.5, 39.3.

$[\alpha]_D^{25}$ = –28.1 (c = 1.00, $CHCl_3$).

Chiral HPLC (Chiralpak IA with guard, 5.0 % IPA, 95.0 % hexane, 1.0 mL/min, 25 °C, λ = 254 nm, 10 µL injection).

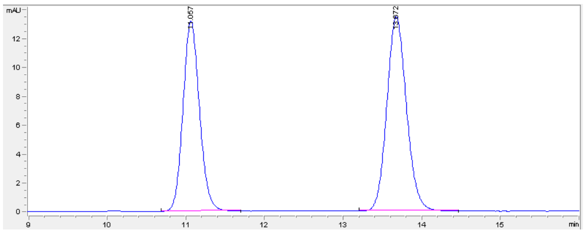

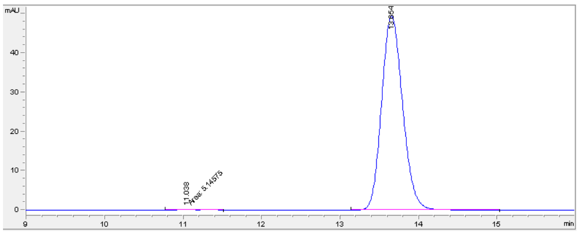

#	Time	Type	Area	Height	Width	Area%	Symmetry
1	11.057	BB	192.2	13.2	0.2242	45.109	0.862
2	13.672	BB	233.9	13.5	0.2689	54.891	0.867

#	Time	Type	Area	Height	Width	Area%	Symmetry
1	11.038	MM	5.1	1.9E-1	0.4585	0.555	0.399
2	13.654	BB	921.6	49.5	0.2913	99.445	0.838

(*S*)-2-(Dibenzylamino)-3-methylbutan-1-ol, 1f

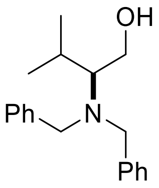

To a stirred suspension of *L*-valine (944 mg, 8.5 mmol, 1.0 eq.) and K_2CO_3 (4.69 g, 34 mmol, 4.0 eq.) in EtOH (10 mL) and water (10 mL) at RT was added benzyl bromide (3.53 mL, 30 mmol, 3.5 eq.) dropwise. The resulting solution was then and left to stir for 16 h before being heated to reflux for a final 2 h. The reaction mixture was then cooled to RT and the EtOH removed *in vacuo*. The resulting mixture was then extracted with CH_2Cl_2 (3 × 10 mL), and the combined organic extracts washed with brine (20 mL), dried over Na_2SO_4, and concentrated *in vacuo* to give the crude product which was used in the next step without further purification. The crude perbenzylated valine (3.29 g) was

subjected to **General Procedure E**. Purification by column chromatography (SiO_2, eluent load, pentane:Et_2O, 75:25 to 70:30) gave the desired alcohol **1f** (1.56 g, 65% over two steps, >99:1 e.r.) as a colourless oil.

The spectral data matched that previously reported in the literature.[9]

^{1}H NMR ($CDCl_3$, 400 MHz) δ = 7.49 – 7.13 (m, 10H), 3.89 (d, *J* = 13.2 Hz, 2H), 3.68 (d, *J* = 13.2 Hz, 2H), 3.61 – 3.52 (m, 1H), 3.44 (t, *J* = 10.2 Hz, 1H), 2.97 (s, 1H), 2.59 – 2.49 (m, 1H), 2.07 (h, *J* = 6.7 Hz, 1H), 1.14 (d, *J* = 6.7 Hz, 3H), 0.89 (d, *J* = 6.7 Hz, 3H).

^{13}C NMR ($CDCl_3$, 101 MHz) δ = 139.9 (2C), 129.4 (4C), 128.6 (4C), 127.3 (2C), 64.9, 59.4, 54.4 (2C), 27.8, 22.9, 20.3.

$[\alpha]_D^{25}$ = +27.4 (c = 1.00, $CHCl_3$).

Chiral HPLC (Chiralpak IA with guard, 5.0 % IPA, 95.0 % hexane, 1.0 mL/min, 25 °C, λ = 254 nm, 10 µL injection).

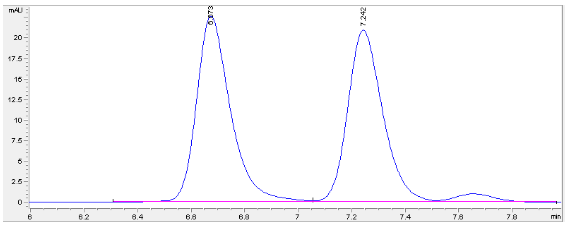

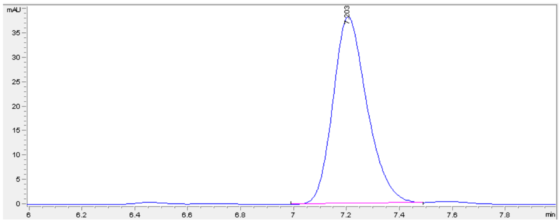

#	Time	Type	Area	Height	Width	Area%	Symmetry
1	6.673	BV	202.1	22.7	0.134	50.102	0.703
2	7.242	VV R	201.2	20.9	0.1385	49.898	0.761

#	Time	Type	Area	Height	Width	Area%	Symmetry
1	7.203	BB	343	38.3	0.1384	100.000	0.759

(*S*)-2-Phenyl-2-(tritylamino)ethan-1-ol, 1g

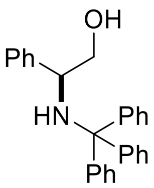

According to a literature procedure,[10] to a stirred solution of *L*-phenylglycine (1.51 g, 10 mmol, 1.0 eq.) in DMF (10 mL) at RT in flame-dried glassware under N_2 was added Me_3SiCl (1.33 mL, 10.5 mmol, 1.05 eq.) and left to stir for 10 min. A solution of trityl chloride (2.93 g, 10 mmol, 1.05 eq.) in

DMF (5 mL) was added dropwise, followed by dropwise addition of Et_3N (2.91 mL, 21 mmol, 2.1 eq.) and the reaction left to stir for 2 h. The reaction mixture was then quenched by the addition of water (20 mL) and acidified to pH 3-4 by the dropwise addition of 3M aq. HCl. The resulting mixture was then extracted with Et_2O (3 × 30 mL) and the combined organic layers washed with brine (50 mL), dried over Na_2SO_4, and concentrated *in vacuo* to give the crude product that was used without further purification. The crude tritylated phenylglycine (3.93 g) was subjected to **General Procedure E**. Purification by column chromatography (SiO_2, eluent load, pentane:Et_2O, 80:20) gave the desired alcohol **1g** (1.94 g, 51% over two steps, 99:1 e.r.) as a colourless solid.

The spectral data matched that previously reported in the literature.[10]

^{1}H NMR ($CDCl_3$, 400 MHz) δ = 7.61 – 7.49 (m, 6H), 7.35 – 7.12 (m, 14H), 3.83 (t, *J* = 4.9 Hz, 1H), 3.24 (dd, *J* = 10.9, 4.2 Hz, 1H), 2.82 (dd, *J* = 10.9, 5.2 Hz, 1H).

^{13}C NMR ($CDCl_3$, 101 MHz) δ = 146.6 (3C), 143.6, 129.0 (6C), 128.4 (2C), 127.9 (6C), 127.2 (2C), 126.7, 126.5 (3C), 71.9, 67.1, 58.6.

$[\alpha]_D^{25}$ = +114.2 (c = 1.00, $CHCl_3$).

Chiral HPLC (Chiralpak IA with guard, 10.0 % IPA, 90.0 % hexane, 1.0 mL/min, 25 °C, λ = 254 nm, 10 µL injection).

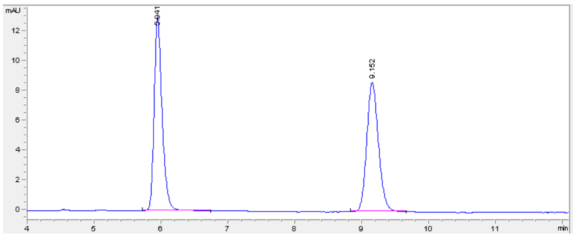

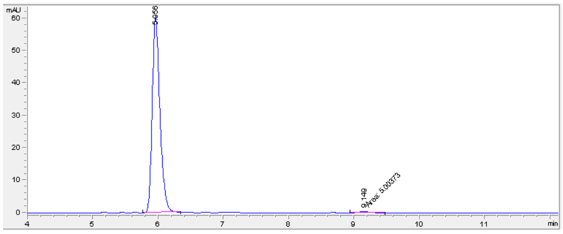

#	Time	Type	Area	Height	Width	Area%	Symmetry
1	5.941	BB	107.1	13	0.1241	50.312	0.699
2	9.152	BB	105.8	8.7	0.1861	49.688	0.826

#	Time	Type	Area	Height	Width	Area%	Symmetry
1	5.956	BB	495.8	60.6	0.1235	99.001	0.707
2	9.149	MM	5	3.8E-1	0.2173	0.999	0.852

Methyl benzyl-*L*-threoninate, S6

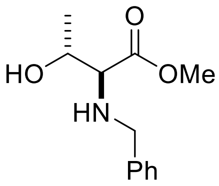

To a stirred solution of methyl *L*-threoninate hydrochloride (2.51 g, 15 mmol, 1.0 eq.) in glacial acetic acid (7.5 mL) and CH_2Cl_2 (12.5 mL) at RT were added benzaldehyde (3.06 mL, 30 mmol, 2.0 eq.) and crushed 4Å molecular sieves (2.5 g), and the reaction left to stir for 1 h. The mixture was then cooled to 0 °C and $NaBH_3CN$ (1.89 g, 30 mmol, 2.0 eq.) was added portionwise with substantial evolution of gas. The reaction was then warmed to RT and left to stir for 4 h before being carefully quenched by the slow addition of sat. aq. $NaHCO_3$ (100 mL) with further gas evolution being observed. The resulting mixture was then extracted with CH_2Cl_2 (3 × 50 mL) and the combined organic extracts were washed with brine (50 mL), dried over Na_2SO_4, and concentrated *in vacuo*. Purification by column chromatography (SiO_2, eluent load, pentane:Et_2O, 50:50 to 30:70) gave the desired product **S6** (1.60 g, 48%, >99:1 e.r.) as a clear oil.

The spectral data matched that previously reported in the literature.[11]

^{1}H NMR ($CDCl_3$, 400 MHz) δ = 7.39 – 7.22 (m, 5H), 3.84 (d, *J* = 13.0 Hz, 1H), 3.72 (s, 3H), 3.71 – 3.66 (m, 1H), 3.04 (d, *J* = 7.6 Hz, 1H), 1.20 (d, *J* = 6.2 Hz, 3H).

^{13}C NMR ($CDCl_3$, 101 MHz) δ = 174.2, 139.2, 128.6 (2C), 128.5 (2C), 127.5, 68.1, 67.4, 52.8, 52.1, 19.5.

Chiral HPLC (Chiralpak IA with guard, 5.0 % IPA, 95.0 % hexane, 1.0 mL/min, 25 °C, λ = 254 nm, 10 µL injection).

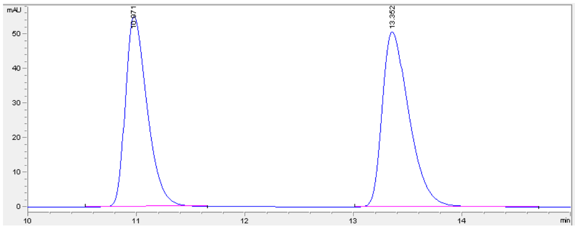

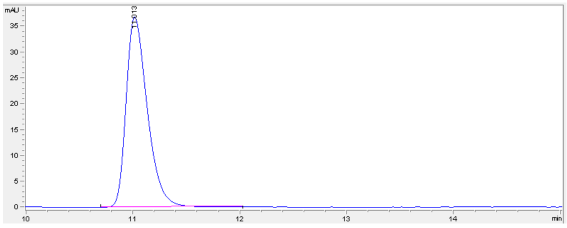

#	Time	Type	Area	Height	Width	Area%	Symmetry
1	10.971	BB	767.2	55.2	0.2143	47.076	0.605
2	13.352	BB	862.5	50.6	0.2607	52.924	0.57

#	Time	Type	Area	Height	Width	Area%	Symmetry
1	11.013	BB	509.8	36.9	0.2112	100.000	0.63

Methyl (4*S*,5*R*)-3-benzyl-5-methyloxazolidine-4-carboxylate, S7

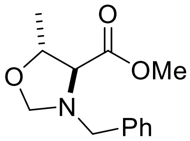

According to a literature procedure,[12] to a stirred solution of benzylated threonine methyl ester **S6** (1.34g, 6 mmol, 1.0 eq.), Na_2SO_4 (2.56 g, 18 mmol, 3.0 eq.) and camphorsulfonic acid (278 mg, 1.2 mmol, 0.2 eq.) in CH_2Cl_2 (60 mL) at RT was added formaldehyde (37% solution in water, 0.97 mL, 12 mmol, 2.0 eq.) and the reaction left to stir for 16 h. The reaction mixture was then filtered through a pad of celite (eluting with CH_2Cl_2) and the resulting solution washed with 1M aq. NaOH (30 mL) and water (30 mL), dried over Na_2SO_4, and concentrated *in vacuo*. Purification by column chromatography (SiO_2, eluent load, pentane:EtOAc, 83:17) gave the desired compound **S7** (1.34 g, 94%) as a colourless oil.

IR (film) ν_{max}/cm^{-1} = 2871, 1734, 1278, 1198, 1171, 1073, 749, 699.

^{1}H NMR ($CDCl_3$, 400 MHz) δ = 7.36 – 7.16 (m, 5H), 4.45 (d, *J* = 5.4 Hz, 1H), 4.38 (d, *J* = 5.4 Hz, 1H), 4.03 (p, *J* = 6.2 Hz, 1H), 3.87 (s, 2H), 3.63 (s, 3H), 3.12 (d, *J* = 6.7 Hz, 1H), 1.39 (d, *J* = 6.2 Hz, 3H).

^{13}C NMR ($CDCl_3$, 101 MHz) δ = 172.3, 138.1, 129.0 (2C), 128.6 (2C), 127.6, 86.6, 77.3, 71.6, 59.5, 52.3, 20.3.

HRMS (ESI$^+$) Found $[M+H]^+$ = 236.1282; $C_{13}H_{18}O_3N$ requires 236.1281, Δ 0.29 ppm.

$[\alpha]_D^{25}$ = –56.7 (c = 1.00, $CHCl_3$).

((4*R*,5*R*)-3-Benzyl-5-methyloxazolidin-4-yl)methanol, 1h

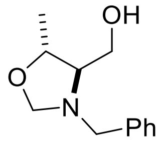

Cyclic threonine derivative **S7** (940 mg, 4 mmol, 1.0 eq.) was subjected to **General Procedure E**. Purification by column chromatography (SiO_2, eluent load, pentane:EtOAc, 70:30) gave the desired alcohol **1h** (769 mg, 93%, >99:1 e.r.) as a colourless oil.

The product **1h** was observed (in CD_2Cl_2) to exist in an approximately 4:1 equilibrium ratio with its structural isomer **1h'**, presumably interconverting through intermediate **A** on a timescale too slow to be detectable by NMR spectroscopy, but clearly visible by HPLC analysis with a characteristic asymmetric "batman" profile.

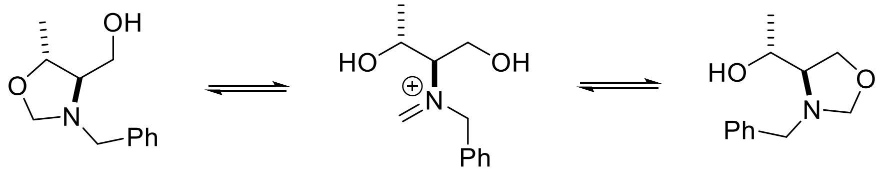

1h **A** **1h'**

IR (film) ν_{max}/cm^{-1} = 3435 (br.), 2870, 1454, 1381, 1071, 1027, 700.

^{1}H NMR (CD_2Cl_2, 400 MHz) δ = 7.41 – 7.23 (m, 5H), 4.36 (d, *J* = 6.6 Hz, 0.2H), 4.32 (s, 2H), 4.18 (d, *J* = 6.6 Hz, 0.2H), 3.99 (dd, *J* = 8.4, 7.0 Hz, 0.2H), 3.90 – 3.81 (m, 2H), 3.81 – 3.74 (m, 1H), 3.48 – 3.33 (m, 2H), 2.91 (ddd, *J* = 8.9, 7.0, 4.2 Hz, 0.2H), 2.65 (ddd, *J* = 6.4, 5.4, 4.5 Hz, 1H), 1.35 (d, *J* = 6.2 Hz, 3H), 1.03 (d, *J* = 6.1 Hz, 0.7H).

^{13}C NMR (CD_2Cl_2, 101 MHz) δ = 139.70, 139.67, 129.5 (2C), 129.3 (2C), 129.04 (2C), 129.00 (2C), 128.0, 127.9, 86.1, 85.4, 76.2, 72.3, 71.6, 67.6, 66.2, 61.3, 60.8, 60.0, 20.3, 19.1.

HRMS (ESI$^+$) Found $[M+H]^+$ = 208.1333; $C_{12}H_{18}O_2N$ requires 208.1332, Δ 0.46 ppm.

$[\alpha]_D^{25}$ = –16.1 (c = 1.00, $CHCl_3$).

Chiral HPLC (Chiralpak IA with guard, 1.0 % IPA, 99.0 % hexane, 1.0 mL/min, 25 °C, λ = 254 nm, 10 µL injection).

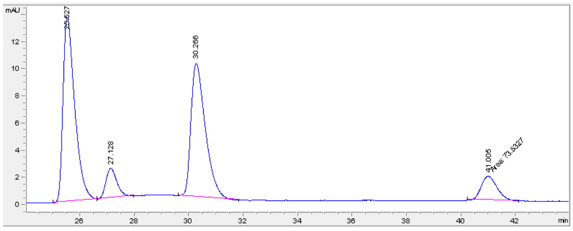

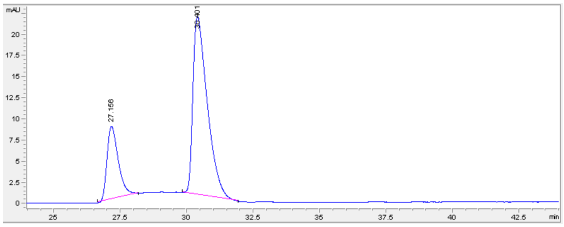

#	Time	Type	Area	Height	Width	Area%	Symmetry
1	25.527	BB	410.1	13.6	0.4506	45.144	0.529
2	27.128	BB	61.6	2.2	0.4207	6.776	0.759
3	30.266	BB	363.2	9.9	0.5355	39.984	0.528
4	41.005	MM	73.5	1.8	0.6783	8.095	0.782

#	Time	Type	Area	Height	Width	Area%	Symmetry
1	27.156	BB	257.6	8.7	0.4504	24.414	0.613
2	30.401	BB	797.5	21.1	0.5526	75.586	0.433

Methyl benzyl-*L*-serinate, S8

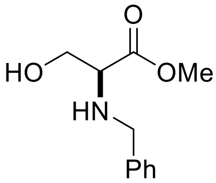

To stirring MeOH (50 mL) at RT was added acetyl chloride (8.62 mL, 120 mmol, 10 eq.) dropwise and the resulting solution left to stir for 10 min. Commercially available *N*-benzyl-*L*-serine (2.34 g, 12 mmol, 1.0 eq.) was added portionwise and the reaction heated to reflux for 18 h. Upon cooling to RT, the reaction was cautiously quenched by the addition of sat. aq. $NaHCO_3$ (100 mL) and the resulting mixture extracted with EtOAc (3 × 50 mL). The combined organic extracts were washed with brine (50 mL), dried over Na_2SO_4, and concentrated *in vacuo*. Purification by column chromatography (SiO_2, eluent load, pentane:Et_2O, 20:80) gave the desired methyl ester **S8** (1.54 g, 49%) as a colourless oil.

The spectral data matched that previously reported in the literature.[13]

^{1}H NMR ($CDCl_3$, 400 MHz) δ = 7.43 – 7.21 (m, 5H), 3.87 (dd, *J* = 13.0, 8.3 Hz, 1H), 3.81 – 3.68 (m, 5H), 3.62 (dd, *J* = 10.8, 6.1 Hz, 1H), 3.43 (ddd, *J* = 7.2, 6.1, 4.1 Hz, 1H), 2.72 – 2.67 (br. s, 1H, O*H* or N*H*), 2.58 – 2.55 (br. s, 1H, O*H* or N*H*).

^{13}C NMR ($CDCl_3$, 101 MHz) δ = 173.5, 139.3, 128.6 (2C), 128.4 (2C), 127.5, 62.6, 62.0, 52.3, 52.2.

Methyl (*S*)-3-benzyloxazolidine-4-carboxylate, S9

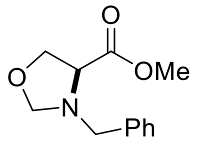

According to a literature procedure,[12] to a stirred solution of benzylated serine methyl ester **S8** (1.25g, 6 mmol, 1.0 eq.), Na_2SO_4 (2.56 g, 18 mmol, 3.0 eq.) and camphorsulfonic acid (278 mg, 1.2 mmol, 0.2 eq.) in CH_2Cl_2 (60 mL) at RT was added formaldehyde (37% solution in water, 0.97 mL, 12 mmol, 2.0 eq.) and the reaction left to stir for 16 h. The reaction mixture was then filtered through a pad of celite (eluting with CH_2Cl_2) and the resulting solution washed with 1M aq. NaOH (30 mL) and water (30 mL), dried over Na_2SO_4, and concentrated *in vacuo*. Purification by column chromatography (SiO_2, eluent load, pentane:Et_2O, 70:30) gave the desired compound **S9** (1.24 g, 94%) as a colourless oil.

IR (film) ν_{max}/cm^{-1} = 2953, 2887, 1736, 1454, 1437, 1200, 1177, 1049, 1013, 734, 700.

^{1}H NMR ($CDCl_3$, 400 MHz) δ = 7.43 – 7.22 (m, 5H), 4.43 (s, 2H), 4.16 (tt, *J* = 8.2, 1.1 Hz, 1H), 3.95 – 3.84 (m, 3H), 3.75 – 3.65 (m, 4H).

^{13}C NMR ($CDCl_3$, 101 MHz) δ = 172.5, 138.2, 128.9 (2C), 128.6 (2C), 127.6, 87.1, 67.3, 64.5, 58.8, 52.3.

HRMS (ESI^+) Found $[M+H]^+$ = 222.1126; $C_{12}H_{16}O_3N$ requires 222.1125, Δ 0.49 ppm.

$[\alpha]_D^{25}$ = –29.3 (c = 1.00, $CHCl_3$).

(3-Benzyloxazolidin-4-yl)methanol, 1i

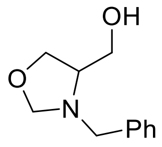

Cyclic serine derivative **S9** (940 mg, 4 mmol, 1.0 eq.) was subjected to **General Procedure E**. Purification by column chromatography (SiO_2, eluent load, pentane:EtOAc, 50:50) gave the desired alcohol **1i** (670 mg, 69%, 50:50 e.r.) as a colourless solid.

The product **1i** was observed by HPLC analysis to be racemic, with the two enantiomers interconverting with a half-life of racemisation of 2.9 min, as determined by analysis of their characteristic “batman” profile. We propose that this was a result of **1i** interconverting through achiral intermediate **B** to its enantiomer **ent-1i** in a similar manner to that observed with **1h** and **1h’**.

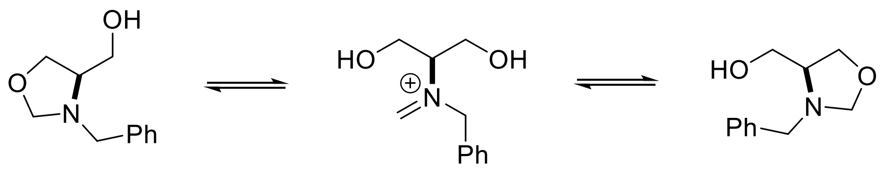

1i **B** **ent-1i**

IR (film) ν_{max}/cm^{-1} = 2877, 1454, 1357, 1041, 1012, 699.

^{1}H NMR ($CDCl_3$, 400 MHz) δ = 7.36 – 7.18 (m, 5H), 4.35 – 4.24 (m, 2H), 3.99 (dd, *J* = 8.4, 7.2 Hz, 1H), 3.75 (s, 2H), 3.43 (dd, *J* = 8.4, 5.0 Hz, 1H), 3.37 (dd, *J* = 6.1, 3.5 Hz, 2H), 3.22 (tt, *J* = 7.2, 5.0 Hz, 1H), 2.37 (br. s, 1H).

^{13}C NMR ($CDCl_3$, 101 MHz) δ = 138.7, 128.9 (2C), 128.7 (2C), 127.7, 85.7, 66.5, 64.8, 61.9, 59.2.

HRMS (ESI^+) Found $[M+H]^+$ = 194.1178; $C_{11}H_{16}O_2N$ requires 194.1176, Δ 1.09 ppm.

Chiral HPLC (Chiralpak IA with guard, 10.0 % IPA, 90.0 % hexane, 1.0 mL/min, 25 °C, λ = 254 nm, 10 µL injection).

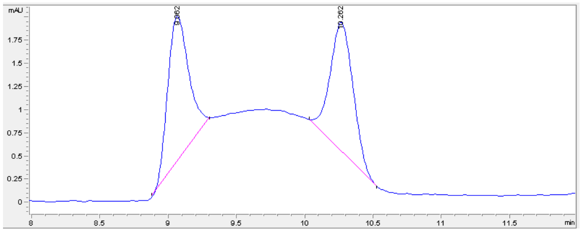

#	Time	Type	Area	Height	Width	Area%	Symmetry
1	9.062	BB	16.8	1.6	0.1645	51.128	0.824
2	10.262	BB	16.1	1.4	0.1798	48.872	0.874

Methyl trityl-*L*-tyrosinate, S10

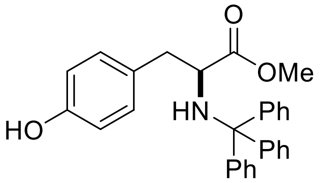

To a stirred solution of commercially available *L*-tyrosine methyl ester hydrochloride (9.24 g, 40 mmol, 1.0 eq.) and trityl chloride (11.16 g, 40 mmol, 1.0 eq.) in DMF (300 mL) at 0 °C was added Et_3N (11.07 mL, 80 mmol, 2.0 eq.) dropwise over the course of 15 min. The mixture was then warmed to RT and left to stir for 3 h. The reaction was then quenched by the addition of water (200 mL) and the mixture extracted with EtOAc (3 × 100 mL). The combined organic extracts were washed with brine (3 × 100 mL), dried over Na_2SO_4, and concentrated *in vacuo* to give the desired product **S10** (17.23 g, 99%) as an oil that crystallised under high vacuum into a colourless solid.

The spectral data matched that previously reported in the literature.[14]

^{1}H NMR ($CDCl_3$, 400 MHz) δ = 7.45 – 7.38 (m, 6H), 7.26 – 7.18 (m, 6H), 7.18 – 7.12 (m, 3H), 7.06 (d, *J* = 8.4 Hz, 2H), 6.76 (d, *J* = 8.4 Hz, 2H), 3.52 (t, *J* = 6.7 Hz, 1H), 3.05 (s, 3H), 2.96 – 2.81 (m, 2H).

^{13}C NMR ($CDCl_3$, 101 MHz) δ = 175.3, 154.7, 146.0 (3C), 131.0 (2C), 128.9 (6C), 128.1, 127.9 (6C), 126.5 (3C), 115.2 (2C), 71.1, 58.6, 51.5, 41.5.

Methyl (*S*)-3-(4-methoxyphenyl)-2-(tritylamino)propanoate, S11

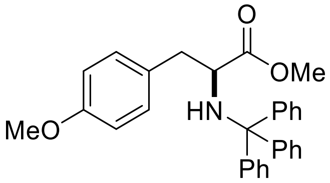

To a stirred solution of tyrosine derivative **S10** (13.11 g, 30 mmol, 1.0 eq.) and K_2CO_3 (5.38 g, 39 mmol, 1.3 eq.) in DMF (90 mL) at RT was added MeI (2.05 mL, 33 mmol, 1.1 eq.) dropwise and the reaction left to stir for 17 h. The reaction was then concentrated *in vacuo* and the residue redissolved in water (30 mL). The resulting mixture was extracted with EtOAc (3 × 30 mL) and the

combined organic extracts washed with brine (3 × 30 mL), dried over Na_2SO_4, and concentrated *in vacuo*. Purification by column chromatography (SiO_2, eluent load, pentane:Et_2O, 80:20) gave the methylated product **S11** (2.04 g, 91%) as a colourless solid.

m.p. = 83–85 °C.

IR (film) ν_{max}/cm^{-1} = 2981, 2889, 1738, 1514, 1446, 1383, 1248, 1159, 1117, 822, 783, 773, 706.

^{1}H NMR ($CDCl_3$, 400 MHz) δ = 7.46 – 7.37 (m, 6H), 7.25 – 7.18 (m, 6H), 7.18 – 7.11 (m, 5H), 6.86 (d, *J* = 8.7 Hz, 2H), 3.81 (s, 3H), 3.56 – 3.48 (m, 1H), 3.05 (s, 3H), 2.90 (dp, *J* = 13.0, 6.5 Hz, 2H).

^{13}C NMR ($CDCl_3$, 101 MHz) δ = 175.1, 158.6, 146.0 (3C), 130.9 (2C), 129.7, 129.0 (6C), 127.9 (6C), 126.4 (3C), 113.8 (2C), 71.1, 58.5, 55.4, 51.4, 41.6.

HRMS (ESI^+) Found $[M+Na]^+$ = 474.2038; $C_{30}H_{29}O_3NNa$ requires 474.2040, Δ –0.37 ppm.

$[\alpha]_D^{25}$ = +61.0 (c = 1.00, $CHCl_3$).

(*S*)-3-(4-Methoxyphenyl)-2-(tritylamino)propan-1-ol, 1j

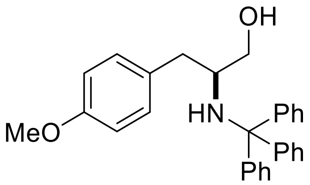

Methylated tyrosine derivative **S11** (9.02 g, 20 mmol, 1.0 eq.) was subjected to **General Procedure E**. Purification by column chromatography (SiO_2, eluent load, pentane:Et_2O, 80:20 to 60:30) gave the desired alcohol **1j** (2.24 g, 27%, >99:1 e.r.) as a colourless solid.

m.p. = 48–50 °C.

IR (film) ν_{max}/cm^{-1} = 2981, 1383, 1246, 1155, 1031, 954, 746, 705.

^{1}H NMR ($CDCl_3$, 400 MHz) δ = 7.57 (ddd, *J* = 8.3, 2.2, 1.1 Hz, 6H), 7.34 – 7.26 (m, 6H), 7.25 – 7.17 (m, 3H), 6.86 (d, *J* = 8.4 Hz, 2H), 6.74 (d, *J* = 8.4 Hz, 2H), 3.75 (s, 3H), 3.13 (dd, *J* = 11.0, 2.8 Hz, 1H), 2.94 (dd, *J* = 11.0, 4.1 Hz, 1H), 2.77 (dtd, *J* = 8.9, 4.6, 2.5 Hz, 1H), 2.47 (dd, *J* = 13.3, 9.8 Hz, 1H), 2.24 (dd, *J* = 13.3, 4.6 Hz, 1H).

^{13}C NMR ($CDCl_3$, 101 MHz) δ = 158.1, 146.7 (3C), 131.1, 130.5 (2C), 128.9 (6C), 128.1 (6C), 126.6 (3C), 113.8 (2C), 71.5, 62.5, 55.5, 55.3, 38.4.

HRMS (ESI^+) Found $[M+Na]^+$ = 446.2088; $C_{29}H_{29}O_2NNa$ requires 446.2091, Δ –0.58 ppm.

$[\alpha]_D^{25}$ = –16.1 (c = 1.00, $CHCl_3$).

Chiral HPLC (Chiralpak IA with guard, 5.0 % IPA, 95.0 % hexane, 1.0 mL/min, 25 °C, λ = 254 nm, 10 µL injection).

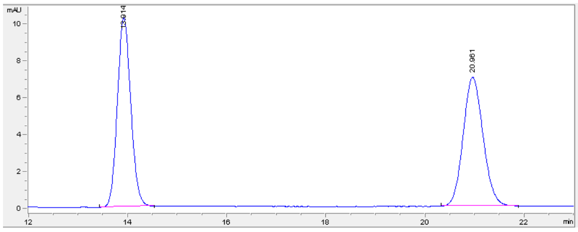

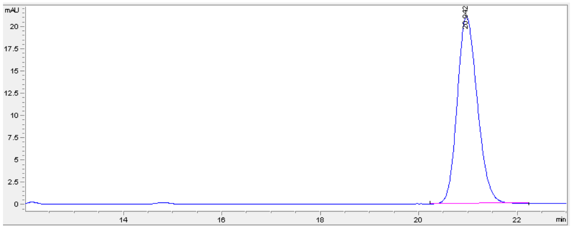

#	Time	Type	Area	Height	Width	Area%	Symmetry
1	13.914	BB	194.4	10.3	0.2896	49.768	0.882
2	20.961	BB	196.3	7	0.4284	50.232	0.889

#	Time	Type	Area	Height	Width	Area%	Symmetry
1	20.942	BB	599.9	21.1	0.4401	100.000	0.787

(*S*)-2,6-Bis(dibenzylamino)hexan-1-ol, 1k

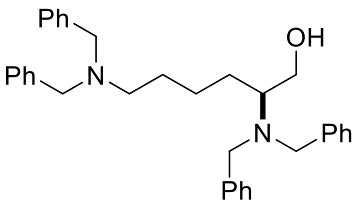

According to a literature procedure,[15] to a stirred solution of *L*-lysine (1.83 g, 10 mmol, 1.0 eq.) in EtOH (20 mL) at RT was added K_2CO_3 (11.0 g, 80 mmol, 8.0 eq.) and benzyl bromide (8.30 mL, 70 mmol, 7.0 eq.) and the reaction heated to 60 °C for 4 d. The resulting white slurry was then filtered through a pad of celite, eluting with EtOAc (50 mL) and the filtrate washed with brine (40 mL), dried over Na_2SO_4 and concentrated *in vacuo* to give a crude yellow oil that was used without further purification. The crude perbenzylated lysine ester (8.02 g) was subjected to **General Procedure E**. Purification by column chromatography (SiO_2, eluent load, pentane:Et_2O, 60:40) gave the desired alcohol **1k** (2.47 g, 50% over two steps, 98:2 e.r.) as a clear oil.

The spectral data matched that previously reported in the literature.[15]

^{1}H NMR ($CDCl_3$, 400 MHz) δ = 7.51 – 7.25 (m, 20H), 3.83 (d, *J* = 13.3 Hz, 2H), 3.73 – 3.48 (m, 5H), 3.45 – 3.37 (m, 3H), 3.22 (br. s, 1H), 2.80 (hept, *J* = 4.9 Hz, 1H), 2.50 (h, *J* = 4.9 Hz, 2H), 1.72 – 1.49 (m, 3H), 1.43 – 1.22 (m, 2H), 1.15 (dtd, *J* = 13.1, 9.6, 5.1 Hz, 1H).

^{13}C NMR ($CDCl_3$, 101 MHz) δ = 140.0 (2C), 139.5 (2C), 129.1 (4C), 128.9 (4C), 128.5 (4C), 128.3 (4C), 127.3 (2C), 126.9 (2C), 61.0, 59.0, 58.5 (2C), 53.3 (2C), 52.9, 27.3, 24.9, 24.7.

$[\alpha]_D^{25}$ = +66.8 (c = 1.00, $CHCl_3$).

Chiral HPLC (Chiralpak IA with guard, 5.0 % IPA, 95.0 % hexane, 1.0 mL/min, 25 °C, λ = 254 nm, 10 µL injection).

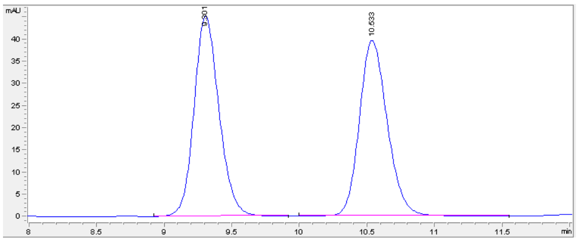

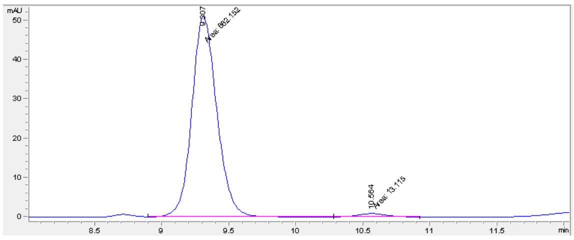

#	Time	Type	Area	Height	Width	Area%	Symmetry
1	9.301	BB	578.3	44.9	0.1981	50.435	0.813
2	10.533	BB	568.3	39.5	0.222	49.565	0.789

#	Time	Type	Area	Height	Width	Area%	Symmetry
1	9.307	MF	662.2	50.8	0.2171	98.058	0
2	10.564	FM	13.1	8.5E-1	0.2557	1.942	0.93

(*S*)-3-(1H-Indol-3-yl)-2-(tritylamino)propan-1-ol, S12

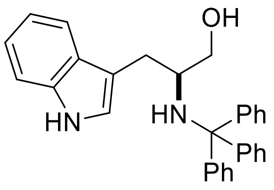

The initial tritylation was performed using the method reported by Zervas and Theodoropoulos.[8] To a stirred slurry of *L*-tryptophan (2.04 mg, 10 mmol, 1.0 eq.) in CH_2Cl_2 (16 mL) at RT was added Me_3SiCl (1.36 mL, 10.7 mmol, 1.07 eq.) and the reaction left to stir for 4 h. Et_3N (2.97 mL, 21.5 mmol, 2.15 eq.) was added, followed by the dropwise addition of trityl chloride (3.12 g, 11.2 mmol, 1.12 eq.) in CH_2Cl_2 (8 mL). The reaction was then left to stir at RT for 20 h, before being quenched by the addition of MeOH (10 mL) and concentrated *in vacuo*. The residue was redissolved in EtOAc (15 mL)

and CH_2Cl_2 (15 mL) before being sequentially washed with a 5% precooled aq. solution of citric acid (2 × 30 mL) and brine (2 × 30 mL), dried over Na_2SO_4, and concentrated *in vacuo* to give crude tritylated tyrosine that was used without further purification. The crude tritylated tryptophan (4.46 g) was subjected to **General Procedure E**. Purification by column chromatography (SiO_2, eluent load, pentane:Et_2O, 50:50) gave the desired alcohol **S12** (1.41 g, 33% over two steps) as a colourless solid.

m.p. = 75–77 °C.

IR (film) ν_{max}/cm^{-1} = 3421, 1490, 1456, 1340, 1216, 1031, 902, 744, 707, 669.

^{1}H NMR ($CDCl_3$, 400 MHz) δ = 7.95 – 7.89 (m, 1H), 7.60 – 7.50 (m, 6H), 7.35 (dq, *J* = 8.0, 1.0 Hz, 1H), 7.30 – 7.23 (m, 7H), 7.22 – 7.11 (m, 4H), 7.03 (ddd, *J* = 8.0, 7.0, 1.0 Hz, 1H), 6.85 (d, *J* = 2.4 Hz, 1H), 3.16 (dd, *J* = 10.8, 2.4 Hz, 1H), 2.97 (dtd, *J* = 9.3, 5.0, 2.5 Hz, 1H), 2.82 – 2.72 (m, 2H), 2.63 (dd, *J* = 14.0, 5.0 Hz, 1H).

^{13}C NMR ($CDCl_3$, 101 MHz) δ = 146.8 (3C), 136.3, 128.9 (6C), 128.0 (6C), 127.9, 126.6 (3C), 122.9, 122.1, 119.4, 119.3, 113.2, 111.1, 71.4, 62.9, 54.2, 29.2.

HRMS (ESI^-) Found $[M–H]^-$ = 431.2130; $C_{30}H_{27}ON_2$ requires 431.2129, Δ 0.35 ppm.

$[\alpha]_D^{25}$ = –5.0 (c = 1.00, $CHCl_3$).

(*S*)-3-(1-Methyl-1H-indol-3-yl)-2-(tritylamino)propan-1-ol, 1l

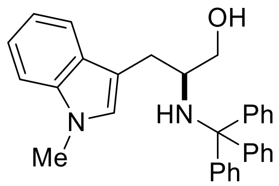

To a stirred solution of NaH (60 wt.%, 176 mg, 4.4 mmol, 1.1 eq.) in DMF (18 mL) at RT was added tritylated tryptophanol **S12** (1.74 g, 4 mmol, 1.0 eq.) in DMF (12 mL) dropwise and the reaction left to stir for 30 min. The reaction was then cooled to 0 °C and MeI (274 µL, 4.4 mmol, 1.1 eq.) was slowly added. The reaction was then warmed to RT and left to stir for 1 h. Water (30 mL) was added, and the mixture extracted with EtOAc (3 × 50 mL) and the combined organic extracts were washed with brine (3 × 50 mL), dried over Na_2SO_4, and concentrated *in vacuo*. Purification by column

chromatography (SiO_2, eluent load, pentane:Et_2O, 60:40) gave the desired compound **1l** (1.31 g, 73%, >99:1 e.r.) as a fluffy pale-yellow solid.

m.p. = 89–91 °C.

IR (film) ν_{max}/cm^{-1} = 2931, 1696, 1486, 1448, 744, 708.

^{1}H NMR ($CDCl_3$, 400 MHz) δ = 7.59 – 7.53 (m, 6H), 7.33 (dt, *J* = 8.0, 1.1 Hz, 1H), 7.30 – 7.22 (m, 7H), 7.21 – 7.16 (m, 4H), 7.01 (ddd, *J* = 8.0, 6.8, 1.1 Hz, 1H), 6.68 (s, 1H), 3.67 (s, 3H), 3.15 (dd, *J* = 10.8, 2.4 Hz, 1H), 2.99 – 2.90 (m, 1H), 2.80 – 2.69 (m, 2H), 2.60 (dd, *J* = 14.0, 5.0 Hz, 1H).

^{13}C NMR ($CDCl_3$, 101 MHz) δ = 146.8 (3C), 137.0, 128.9 (6C), 128.3, 128.0 (6C), 127.7, 126.6 (3C), 121.6, 119.4, 118.8, 111.6, 109.2, 71.4, 62.8, 54.3, 32.7, 29.1.

HRMS (ESI$^+$) Found [M+Na]$^+$ = 469.2250; $C_{31}H_{30}ON_2Na$ requires 469.2250, Δ –0.02 ppm.

$[\alpha]_D^{25}$ = –39.4 (c = 1.00, $CHCl_3$).

Chiral HPLC (Chiralpak IA with guard, 5.0 % IPA, 95.0 % hexane, 1.0 mL/min, 25 °C, λ = 210 nm, 10 µL injection).

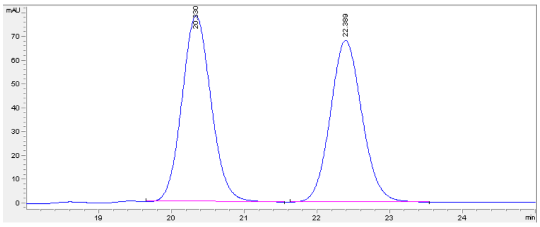

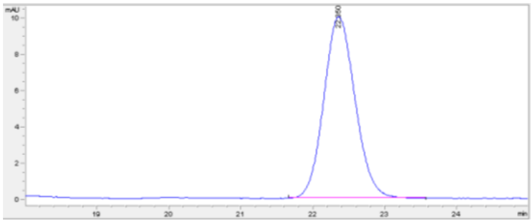

#	Time	Type	Area	Height	Width	Area%	Symmetry
1	20.33	BB	2186.8	78.1	0.4326	51.515	0.888
2	22.389	BB	2058.2	67.8	0.4681	48.485	0.892

#	Time	Type	Area	Height	Width	Area%	Symmetry
1	22.35	BB	304.1	10.1	0.4725	100.000	0.87

2-Methyl-2-(tritylamino)propan-1-ol, 1m

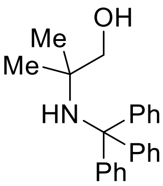

According to a literature procedure,[16] to a stirred solution of commercially available 2-amino-2-methylpropanol (0.48 mL, 5 mmol, 1.0 eq.) and Et_3N (0.76 mL, 5.5 mmol, 1.1 eq.) in DMF (30 mL) at

0 °C was added a solution of trityl chloride (1.46 g, 5.25 mmol, 1.05 eq.) in DMF (5 mL) and the warmed to 40 °C and left to stir for 18 h. The reaction was quenched by the addition of water (50 mL) and the mixture extracted with EtOAc (3 × 30 mL) and the combined organic extracts were washed with brine (3 × 30 mL), dried over Na_2SO_4, and concentrated *in vacuo*. Purification by column chromatography (SiO_2, eluent load, pentane:EtOAc, 85:15) gave the desired compound **1m** (807 mg, 49%) as a colourless solid.

The spectral data matched that previously reported in the literature.[16]

^{1}H NMR ($CDCl_3$, 400 MHz) δ = 7.67 – 7.60 (m, 6H), 7.29 – 7.23 (m, 6H), 7.22 – 7.13 (m, 3H), 2.78 (s, 2H), 0.81 (s, 6H).

^{13}C NMR ($CDCl_3$, 101 MHz) δ = 148.0 (3C), 129.2 (6C), 127.8 (6C), 126.4 (3C), 71.6, 69.8, 55.8, 26.2 (2C).

Methyl 1-aminocyclopentane-1-carboxylate hydrochloride, S13

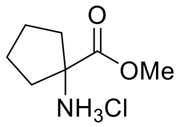

Cycloleucine (2.58 g, 20 mmol, 1.0 eq.) was subjected to **General Procedure C** to give the desired compound **S13** (3.22 g, 90%) as a colourless solid.

m.p. = 200–202 °C

IR (film) ν_{max}/cm^{-1} = 2878 (br.), 1741, 1578, 1523, 1264, 1216, 1190, 993.

^{1}H NMR (D_2O, 400 MHz) δ = 3.87 (s, 3H), 2.45 – 2.28 (m, 2H), 2.05 – 1.82 (m, 6H).

^{13}C NMR (D_2O, 101 MHz) δ = 173.9, 65.3, 53.8, 36.4 (2C), 25.1 (2C).

HRMS (ESI$^+$) Found $[M+H]^+$ = 144.1018; $C_7H_{14}O_2N$ requires 144.1019, Δ –0.44 ppm.

(1-(Tritylamino)cyclopentyl)methanol, 1n

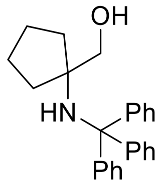

Cycloleucine methyl ester hydrochloride **S13** (2.69 g, 15 mmol, 1.0 eq.) was subjected to **General Procedure D** to give the crude tritylated methyl ester which was immediately subjected to **General Procedure E**. Purification by column chromatography (SiO_2, eluent load, pentane:Et_2O, 80:20) gave the desired alcohol **1n** as a colourless solid (2.66 g, 50% over two steps).

m.p. = 83–85 °C.

IR (film) ν_{max}/cm^{-1} = 1488, 1443, 1035, 743, 699, 632.

^{1}H NMR ($CDCl_3$, 400 MHz) δ = 7.60 – 7.52 (m, 6H), 7.29 – 7.22 (m, 6H), 7.21 – 7.14 (m, 3H), 2.49 (br. s, 1H), 2.42 (s, 2H), 1.66 – 1.54 (m, 2H), 1.53 – 1.40 (m, 2H), 1.40 – 1.17 (m, 4H).

^{13}C NMR ($CDCl_3$, 101 MHz) δ = 147.8 (3C), 129.1 (6C), 127.8 (6C), 126.5 (3C), 70.2, 67.6, 66.7, 36.2 (2C), 24.9 (2C).

HRMS (ESI^+) Found $[M+Na]^+$ = 380.1988; $C_{25}H_{27}ONNa$ requires 380.1985, Δ 0.78 ppm.

2-(Tritylamino)-3-(tritylthio)propan-1-ol, 8a

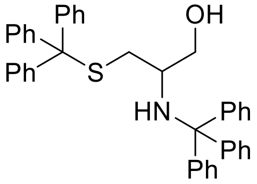

According to a literature procedure,[17] to a rapidly stirring solution of *DL*-cysteine (605 mg, 5 mmol, 1.0 eq.) in water (15 mL) at 0 °C was added Et_2NH (2.57 mL, 25 mmol, 5.0 eq.) in EtOH (15 mL), followed by the portionwise addition of trityl chloride (3.49 g, 12.5 mmol, 2.5 eq.) and the reaction left to stir for 4 h. The reaction mixture was then extracted with Et_2O (3 × 20 mL), after which the combined organic extracts were washed with water (30 mL), dried over Na_2SO_4 and concentrated *in*

vacuo to give a crude product (3.10 g) which was immediately subjected to **General Procedure E**. Purification by column chromatography (SiO_2, eluent load, pentane:Et_2O, 75:25) gave the desired alcohol **8a** (0.63 g, 21% over two steps) as a colourless oil.

m.p. = 74–76 °C.

IR (film) ν_{max}/cm^{-1} = 3057, 1595, 1489, 1446, 1216, 1032, 744, 700, 622.

^{1}H NMR ($CDCl_3$, 400 MHz) δ = 7.49 – 7.43 (m, 6H), 7.37 – 7.32 (m, 6H), 7.29 – 7.14 (m, 18H), 3.13 (dd, *J* = 11.1, 3.3 Hz, 1H), 2.71 (dd, *J* = 11.1, 5.2 Hz, 1H), 2.67 – 2.59 (m, 1H), 2.22 – 2.06 (m, 2H).

^{13}C NMR ($CDCl_3$, 101 MHz) δ = 146.7 (3C), 145.0 (3C), 129.8 (6C), 128.8 (6C), 128.0 (6C), 128.0 (6C), 126.8 (3C), 126.6 (3C), 71.3, 66.7, 63.4, 52.9, 35.1.

HRMS ($APCI^-$) Found $[M-H]^-$ = 590.2516; $C_{41}H_{36}ONS$ requires 590.2523, Δ –1.18 ppm.

***DL*-Tritylmethionine, S14**

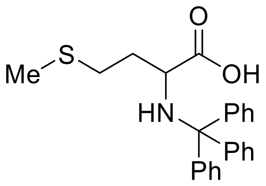

The initial tritylation was performed using the method reported by Zervas and Theodoropoulos.[8] To a stirred solution of *DL*-methionine (745 mg, 5 mmol, 1.0 eq.) in $CHCl_3$:MeCN (7.5:1.5 mL) at RT was added Me_3SiCl (634 µL, 5 mmol, 1.0 eq.) and the reaction heated to reflux for 2 h before being allowed to cool back to RT. Et_3N (1.38 mL, 10 mmol, 2.0 eq.) was slowly added, followed by trityl chloride (1.40 g, 5 mmol, 1.0 eq.) in $CHCl_3$ (5 mL) and the reaction left to stir for 1 h. The reaction mixture was then quenched by the addition of MeOH (25 mL) and the solvent was removed *in vacuo* to give a residue which was redissolved in Et_2O (25 mL) and a 5% precooled solution of citric acid (25 mL). The layers were separated, and the organic layer washed with 1M aq. NaOH (2 × 10 mL) and water (2 × 10 mL). The combined aqueous layers were washed with Et_2O (10 mL), cooled to 0 °C and carefully neutralised by the addition of glacial AcOH. The resulting solution was extracted with Et_2O

(2 × 20 mL) and the combined organic layers washed with water (2 × 20 mL), dried over Na_2SO_4 and concentrated *in vacuo* to give the pure product **S14** (1.20 g, 61%) as a pale yellow foam.

m.p. = 169–171 °C.

IR (film) ν_{max}/cm^{-1} = 2980, 1706, 1490, 1447, 1215, 747, 704, 611.

^{1}H NMR ($CDCl_3$, 400 MHz) δ = 7.50 – 7.41 (m, 6H), 7.25 (t, *J* = 7.6 Hz, 6H), 7.18 (t, *J* = 7.3 Hz, 3H), 3.49 (td, *J* = 5.4, 2.3 Hz, 1H), 2.63 (ddd, *J* = 12.9, 9.6, 5.8 Hz, 1H), 2.50 (ddd, *J* = 13.0, 9.6, 5.8 Hz, 1H), 2.11 (s, 3H), 2.00 (ddt, *J* = 14.8, 10.3, 5.4 Hz, 1H), 1.92 – 1.77 (m, 1H).

^{13}C NMR ($CDCl_3$, 101 MHz) δ = 179.1, 145.6 (3C), 128.9 (6C), 128.1 (6C), 126.9 (3C), 71.6, 55.9, 34.3, 30.1, 15.7.

HRMS (ESI$^-$) Found $[M–H]^-$ = 390.1538; $C_{24}H_{24}O_2NS$ requires 390.1533, Δ 1.13 ppm.

4-(Methylthio)-2-(tritylamino)butan-1-ol, 8b

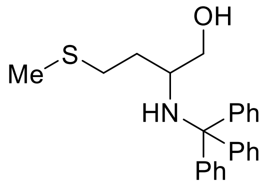

Tritylmethionine **S14** (0.78 g, 2 mmol, 1.0 eq.) was subjected to **General Procedure E**. Purification by column chromatography (SiO_2, eluent load, pentane:Et_2O, 70:30) gave the desired alcohol **8b** (0.56 g, 75%) as a colourless oil.

IR (film) ν_{max}/cm^{-1} = 1489, 1447, 1216, 1051, 1031, 901, 746, 706.

^{1}H NMR ($CDCl_3$, 400 MHz) δ = 7.59 – 7.53 (m, 6H), 7.32 – 7.26 (m, 6H), 7.23 – 7.18 (m, 3H), 3.25 (dd, *J* = 11.1, 3.1 Hz, 1H), 3.04 (dd, *J* = 11.1, 4.1 Hz, 1H), 2.81 – 2.71 (m, 1H), 2.35 (ddd, *J* = 13.0, 9.1, 5.4 Hz, 1H), 2.22 (ddd, *J* = 13.0, 8.7, 6.9 Hz, 1H), 1.94 (s, 3H), 1.64 (dtd, *J* = 14.1, 8.7, 5.4 Hz, 1H), 1.31 (dddd, *J* = 13.5, 9.2, 6.9, 4.1 Hz, 1H).

^{13}C NMR ($CDCl_3$, 101 MHz) δ = 146.6 (3C), 128.8 (6C), 128.1 (6C), 126.7 (3C), 71.5, 63.1, 53.1, 32.6, 30.8, 15.6.

HRMS (ESI$^+$) Found $[M+Na]^+$ = 400.1704; $C_{24}H_{27}ONNaS$ requires 400.1706, Δ –0.36 ppm.

4-(Methylsulfonyl)-2-(tritylamino)butan-1-ol, 8c

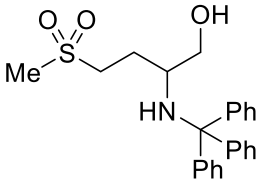

To a stirred solution of trityl methioninol **8b** (285 mg, 0.76 mmol, 1.0 eq.) in CH_2Cl_2 (7.5 mL) at RT was added *m*CPBA (77 wt. %, 357 mg, 1.59 mmol, 2.1 eq.) and the reaction left to stir for 2 h after which the starting material was shown to have been consumed by TLC analysis (EtOAc:CH_2Cl_2, 25:75). To the mixture was added sat. aq. $NaHCO_3$ (1 mL) and the resulting mixture diluted with CH_2Cl_2 (10 mL). The layers were separated, and the organic phase washed with sat. aq. $NaHCO_3$ (10 mL) and brine (10 mL), dried over Na_2SO_4, and concentrated *in vacuo*. Purification by column chromatography (SiO_2, eluent load, EtOAc:CH_2Cl_2, 25:75) gave the desired alcohol **8c** (168 mg, 54%) as a pale yellow solid.

m.p. = 122–124 °C.

IR (film) ν_{max}/cm^{-1} = 1490, 1448, 1295, 1129, 749, 708.

^{1}H NMR ($CDCl_3$, 400 MHz) δ = 7.57 – 7.51 (m, 6H), 7.33 – 7.27 (m, 6H), 7.25 – 7.19 (m, 3H), 3.21 (dd, *J* = 11.0, 3.0 Hz, 1H), 3.06 – 2.85 (m, 3H), 2.85 – 2.79 (m, 1H), 2.75 (s, 3H), 1.85 (dddd, *J* = 13.7, 11.1, 7.5, 5.2 Hz, 1H), 1.62 (dddd, *J* = 13.6, 11.1, 5.2, 3.9 Hz, 1H).

^{13}C NMR ($CDCl_3$, 101 MHz) δ = 146.5 (3C), 128.7 (6C), 128.2 (6C), 126.8 (3C), 71.3, 64.0, 52.0, 51.8, 40.3, 26.7.

HRMS (ESI$^+$) Found $[M+Na]^+$ = 432.1604; $C_{24}H_{27}O_3NNaS$ requires 432.1604, Δ –0.04 ppm.

1-(*tert*-Butyl) 2-methyl 5-oxopyrrolidine-1,2-dicarboxylate, S15

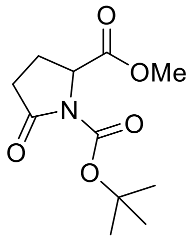

According to a literature procedure,[18] to a stirred solution of *DL*-pyroglutamic acid (15.0 g, 116 mmol, 1.0 eq.) in MeOH (60 mL) at RT was added $SOCl_2$ (16.9 mL, 231 mmol, 2.0 eq.) dropwise and the reaction left to stir for 1 h. The solvent was then removed *in vacuo* and the residue redissolved in EtOAc (200 mL). Et_3N (18.5 mL, 134 mmol, 1.15 eq.) was added dropwise, and the reaction filtered through celite to remove the formed precipitate. DMAP (1.5 g, 12.3 mmol, 0.1 eq.) and Boc_2O (27.8 g, 127 mmol, 1.1 eq.) were added and the reaction left to stir at RT for 3 h. The reaction mixture was then cooled to 0 °C before 1M aq. HCl (13.0 mL) was added and the reaction left to stir for 10 min. The layers were separated, and the organic layer washed with water (20 mL) and concentrated *in vacuo*. Methyl *tert*-butyl ether (27 mL) was added to the residue and cooled to 0 °C with stirring, resulting in precipitation of the desired product **S15** (19.7 g, 70%) as a colourless solid which was separated by filtration.

The spectral data matched that previously reported in the literature.[19]

^{1}H NMR ($CDCl_3$, 400 MHz) δ = 4.58 (dd, *J* = 9.4, 3.1 Hz, 1H), 3.75 (s, 3H), 2.59 (ddd, *J* = 17.5, 10.1, 9.5 Hz, 1H), 2.45 (ddd, *J* = 17.5, 9.4, 3.7 Hz, 1H), 2.29 (ddt, *J* = 13.3, 10.1, 9.4 Hz, 1H), 2.00 (dddd, *J* = 13.3, 9.5, 3.7, 3.1 Hz, 1H), 1.45 (s, 9H).

^{13}C NMR ($CDCl_3$, 101 MHz) δ = 173.3, 171.9, 149.3, 83.6, 58.9, 52.6, 31.2, 27.9 (3C), 21.5.

Methyl N^2-(tert-butoxycarbonyl)-N^5,N^5-dimethylglutaminate, S16

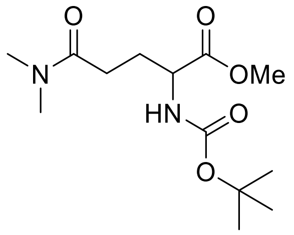

To a stirred solution of 2M Me_2NH in THF (40 mL) at RT was added Boc-protected pyroglumatic acid methyl ester **S15** (9.72 g, 40 mmol, 1.0 eq.) and the reaction left to stir for 48 h. The white precipitate formed was collected by filtration and washed with pentane to provide the desired product **S16** (9.26 g, 80%) as a fluffy white solid.

m.p. = 99–101 °C.

IR (film) ν_{max}/cm^{-1} = 3232, 1725, 1712, 1614, 1534, 1255, 1235, 1159, 1007.

^{1}H NMR ($CDCl_3$, 400 MHz) δ = 5.37 (d, *J* = 7.2 Hz, 1H), 4.34 – 4.24 (m, 1H), 3.73 (s, 3H), 2.98 (s, 3H), 2.94 (s, 3H), 2.40 (tq, *J* = 16.5, 8.0 Hz, 2H), 2.19 (dtd, *J* = 14.5, 7.2, 4.8 Hz, 1H), 1.98 (dq, *J* = 14.5, 8.0 Hz, 1H), 1.43 (s, 9H).

^{13}C NMR ($CDCl_3$, 101 MHz) δ = 173.1, 172.0, 79.9, 53.5, 52.4, 37.3, 35.7, 29.5, 28.4 (3C), 27.8.

HRMS (ESI$^+$) Found $[M+Na]^+$ = 311.1578; $C_{13}H_{24}O_5N_2Na$ requires 311.1577, Δ 0.30 ppm.

Methyl N^5,N^5-dimethylglutaminate hydrochloride, S17

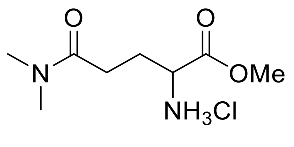

To a stirred solution of 4M HCl in dioxane (10 mL) at RT was added glutamine derivative **S16** (0.86 g, 3 mmol, 1.0 eq.) and the reaction left to stir for 2.5 h. The solvent was then removed *in vacuo* and the resulting solid taken up in toluene (10 mL) and concentrated *in vacuo*. This process was repeated with toluene (10 mL) and with Et_2O (2 × 10 mL) before being dried *in vacuo* to give desired amine hydrochloride salt **S17** (0.97 g, 99%) as a highly hygroscopic colourless gum.

IR (film) ν_{max}/cm^{-1} = 2859 (br.), 1745, 1619, 1436, 1229, 1082.

^{1}H NMR (D_2O, 400 MHz) δ = 4.21 (t, *J* = 6.7 Hz, 1H), 3.86 (s, 3H), 3.08 (s, 3H), 2.95 (s, 3H), 2.67 (q, *J* = 7.1 Hz, 2H), 2.24 (hept, *J* = 7.0 Hz, 2H).

^{13}C NMR (D_2O, 101 MHz) δ = 173.4, 170.3, 53.6, 52.3, 37.3, 35.5, 28.6, 25.1.

HRMS (ESI$^+$) Found $[M+H]^+$ = 189.1233; $C_8H_{17}O_3N_2$ requires 189.1234, Δ –0.25 ppm.

Methyl N^2,N^2-dibenzyl-N^5,N^5-dimethylglutaminate, S18

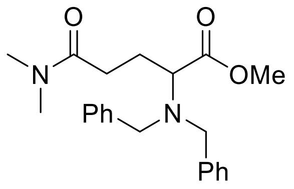

To a stirred solution of amine hydrochloride **S17** (672 mg, 3 mmol, 1.0 eq.) and K_2CO_3 (2.07 g, 15 mmol, 5.0 eq.) in MeCN (20 mL) at RT was added benzyl bromide (0.89 mL, 7.5 mmol, 2.5 eq.) and the reaction left to stir for 24 h. Water (25 mL) was added, and the mixture was extracted with EtOAc (3 × 25 mL). The combined organic extracts were washed with brine (25 mL), dried over Na_2SO_4 and concentrated *in vacuo*. Purification by column chromatography (SiO_2, eluent load, pentane:EtOAc, 40:60) gave the desired compound **S18** (482 mg, 44%) as a clear oil.

IR (film) ν_{max}/cm^{-1} = 2981, 1728, 1645, 1494, 1454, 1397, 1147, 1074, 748, 699.

^{1}H NMR ($CDCl_3$, 400 MHz) δ = 7.37 – 7.18 (m, 10H), 3.90 (d, *J* = 13.7 Hz, 2H), 3.77 (s, 3H), 3.55 (d, *J* = 13.7 Hz, 2H), 3.37 (dd, *J* = 9.1, 5.8 Hz, 1H), 2.90 (s, 3H), 2.84 (s, 3H), 2.36 (ddd, *J* = 15.8, 8.9, 5.5 Hz, 1H), 2.22 (ddd, *J* = 15.8, 9.1, 6.5 Hz, 1H), 2.15 – 2.05 (m, 1H), 2.04 – 1.94 (m, 1H).

^{13}C NMR ($CDCl_3$, 101 MHz) δ = 173.3, 172.2, 139.7 (2C), 129.1 (4C), 128.4 (4C), 127.1 (2C), 60.4, 54.7 (2C), 51.3, 37.1, 35.4, 30.0, 24.8.

HRMS (ESI$^+$) Found $[M+H]^+$ = 369.2173; $C_{22}H_{29}O_3N_2$ requires 369.2173, Δ 0.04 ppm.

4-(Dibenzylamino)-5-hydroxy-*N*,*N*-dimethylpentanamide, 8d

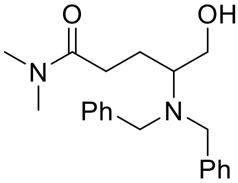

To a stirred solution of methyl ester **S18** (368 mg, 1 mmol, 1.0 eq.) in Et_2O (2 mL) and EtOH (87 µL) at 0 °C was added 4M $LiBH_4$ in THF (375 µL, 1.5 mmol, 1.5 eq.) and the reaction warmed to RT and left to stir for 4 h. Water (4 mL) and EtOAc (4 mL) were added and layers were separated. The aqueous layer was extracted with EtOAc (2 × 4 mL) and the combined extracts dried over Na_2SO_4 and concentrated *in vacuo*. Purification by column chromatography (SiO_2, eluent load, pentane:EtOAc, 20:80) gave the desired compound **8d** (285 mg, 84%) as a clear oil.

IR (film) ν_{max}/cm^{-1} = 2981, 1628, 1494, 1454, 1397, 1152, 748, 700.

^{1}H NMR ($CDCl_3$, 400 MHz) δ = 7.37 – 7.17 (m, 10H), 3.79 (d, *J* = 13.4 Hz, 2H), 3.58 – 3.48 (m, 4H), 2.93 (s, 6H), 2.73 (dddd, *J* = 9.1, 7.6, 6.0, 4.2 Hz, 1H), 2.28 – 2.20 (m, 2H), 2.20 – 2.07 (m, 1H), 1.68 – 1.54 (m, 1H).

^{13}C NMR ($CDCl_3$, 101 MHz) δ = 172.3, 139.6 (2C), 129.1 (4C), 128.4 (4C), 127.2 (2C), 61.0, 58.9, 53.4 (2C), 37.1, 35.5, 30.6, 21.0.

HRMS (ESI$^+$) Found $[M+H]^+$ = 341.2223; $C_{21}H_{29}O_2N_2$ requires 341.2224, Δ –0.19 ppm.

Methyl 5-oxo-5,6,7,8-tetrahydroimidazo[1,5-c]pyrimidine-7-carboxylate, S19

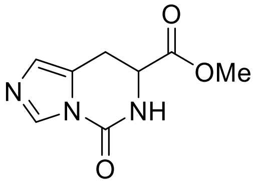

According to a literature procedure,[20] to a stirred solution of *DL*-histidine methyl ester dihydrochloride (8.44 g, 35 mmol, 1.0 eq.) in DMF (175 mL) at RT was added 1,1'-carbonyldiimidazole (5.67 g, 35 mmol, 1.0 eq.) and the reaction heated to 65 °C for 3 h. The reaction

was then concentrated *in vacuo* and the resulting oil dissolved in 1M aq. $NaHCO_3$ (300 mL). The solution was then extracted with $CHCl_3$ (8 × 50 mL) and the combined organic extracts were dried over Na_2SO_4 and concentrated *in vacuo* to give a crude solid (6.61 g) that was recrystallized from MeOH (18 mL) to give the desired product **S19** (4.24 g, 62%) as a colourless crystalline solid.

The spectral data matched that previously reported in the literature.[20]

^{1}H NMR ($CDCl_3$, 400 MHz) δ = 8.17 (d, *J* = 1.0 Hz, 1H), 6.89 (q, *J* = 1.0 Hz, 1H), 6.31 (s, 1H), 4.37 (ddd, *J* = 8.8, 5.2, 2.1 Hz, 1H), 3.82 (s, 3H), 3.39 (ddt, *J* = 15.7, 5.2, 0.9 Hz, 1H), 3.15 (ddd, *J* = 15.7, 8.8, 1.4 Hz, 1H), 2.07 (s, 1H).

^{13}C NMR ($CDCl_3$, 101 MHz) δ = 169.6, 147.8, 135.1, 126.5, 124.0, 53.5, 52.9, 23.3.

7-(Methoxycarbonyl)-2-methyl-5-oxo-5,6,7,8-tetrahydroimidazo[1,5-c]pyrimidin-2-ium iodide, S20

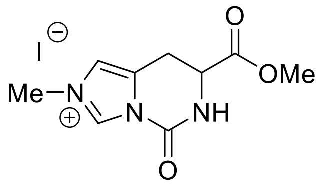

According to a literature procedure,[21] to a stirred solution of pyrimidine **S19** (1.95 g, 10 mmol, 1.0 eq.) in DMF (20 mL) at RT was added MeI (9.34 mL, 150 mmol, 15.0 eq.) and the reaction heated to reflux for 3 h. The solution was then cooled to RT and diluted with Et_2O (100 mL). The mixture was refrigerated for 16 h, resulting in the formation of a precipitate which was filtered and washed with cold Et_2O to give the desired salt **S20** (3.38 g, 99%) as a colourless, crystalline solid.

The spectral data matched that previously reported in the literature.[21]

^{1}H NMR (D_2O, 400 MHz) δ = 7.98 (s, 1H), 7.46 (t, *J* = 1.3 Hz, 1H), 4.79 (t, *J* = 5.7 Hz, 1H), 4.01 (s, 3H), 3.82 (s, 3H), 3.54 (dd, *J* = 5.7, 1.3 Hz, 2H).

^{13}C NMR (D_2O, 101 MHz) δ = 171.3, 165.0, 145.7, 128.4, 120.7, 53.2, 52.0, 36.7, 21.3.

Methyl N$^{\alpha}$-(methoxycarbonyl)-N$^{\tau}$-methylhistidinate, S21

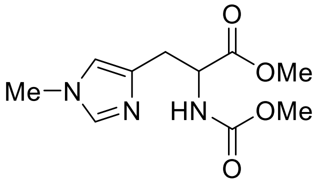

According to a literature procedure,[21] To a stirred solution of methylated salt **S20** (2.70 g, 8 mmol, 1.0 eq.) in MeOH (60 mL) at RT was added conc. H_2SO_4 (172 µL, 3.2 mmol, 0.4 eq.) dropwise and the reaction heated to reflux for 4 h. The mixture was then cooled to RT and NaOMe (200 mg, 3.7 mmol, 0.46 eq.) was added before the solution was concentrated *in vacuo* to a volume of approximately 15 mL. CH_2Cl_2 (15 mL) was added to dilute the mixture before being washed with a 1:1 mixture of sat. aq. $NaHCO_3$ and water (15 mL), dried over Na_2SO_4 and concentrated *in vacuo* to give a colourless oil that solidified under high vacuum to give pure desired product **S21** (1.44 g, 75%) as a colourless solid.

The spectral data matched that previously reported in the literature.[21]

^{1}H NMR ($CDCl_3$, 400 MHz) δ = 7.32 (d, *J* = 1.4 Hz, 1H), 6.65 – 6.60 (m, 1H), 6.31 – 6.24 (m, 1H), 4.55 (dt, *J* = 8.0, 5.1 Hz, 1H), 3.68 (s, 3H), 3.65 (s, 3H), 3.60 (s, 3H), 3.12 – 2.91 (m, 2H).

^{13}C NMR ($CDCl_3$, 101 MHz) δ = 172.4, 156.9, 137.7, 137.5, 117.9, 54.1, 52.4, 52.3, 33.5, 30.0.

3-(1-Methyl-1H-imidazol-4-yl)-2-(tritylamino)propan-1-ol, 8e

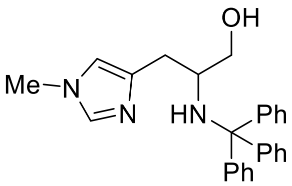

According to a literature procedure,[21] to a stirred solution of methylated histidinate **S21** (1.33 g, 5.5 mmol, 1.0 eq.) in THF (33 mL) and water (8 mL) at 0 °C was added $NaBH_4$ (1.05 g, 27.5 mmol, 5.0 eq.) portionwise before being warmed to RT and left to stir for 1 h. The solution was then cooled to 0 °C and conc. aq. HCl (4.58 mL) was added dropwise, resulting in the evolution of H_2. K_2CO_3 was then

added until the solution became saturated and the layers were separated. The aqueous layer was extracted with CH_2Cl_2 (3 × 20 mL) and the combined organic extracts dried over Na_2SO_4 and concentrated *in vacuo*. Partial purification by column chromatography (SiO_2, eluent load, 7M NH_3 in MeOH:CH_2Cl_2, 10:90) gave the intermediate alcohol (419 mg) along with an unidentified inseparable impurity as a colourless and viscous oil that was highly prone to bumping.

To a stirred solution of the intermediate alcohol (367 mg, 1.72 mmol, 1.0 eq.) in water (20 mL) and MeOH (10 mL) at RT was added $Ba(OH)_2{\cdot}8H_2O$ (1.08 g, 3.44 mmol, 2.0 eq.) before being heated to 90 °C and left to stir for 24 h, after which more $Ba(OH)_2{\cdot}8H_2O$ (0.54 g, 1.72 mmol, 1.0 eq.) was added and the reaction stirred for a further 24 h. The mixture was then cooled to RT and dry ice was added, resulting in the formation of insoluble $BaCO_3$. The reaction was stirred for a further 2 h, after which it was filtered through a pad of celite and concentrated *in vacuo* to give a crude amino alcohol (374 mg) which was used without further purification. To a stirred solution of the crude from the previous step in DMF (25 mL) at RT was added Et_3N (238 µL, 1.72 mmol, 1.0 eq.) and the reaction heated to 50 °C. After being left to stir for 30 min, trityl chloride (480 mg, 1.72 mmol, 1.0 eq.) was added and the reaction left to stir for 1 h. The reaction was then poured into water (50 mL) and placed in the freezer (–18 °C) for 2 h, resulting in formation of a precipitate. Filtration of the mixture afforded a crude solid which was purified by column chromatography (SiO_2, eluent load, 7M NH_3 in MeOH:CH_2Cl_2, 10:90) to give the desired compound **8e** (350 mg, 16% over three steps) as a colourless solid.

m.p. = 162–164 °C.

IR (film) ν_{max}/cm^{-1} = 2981, 1510, 1489, 1448, 1216, 1159, 1031, 746, 706, 643, 621.

^{1}H NMR ($CDCl_3$, 400 MHz) δ = 7.54 – 7.46 (m, 6H), 7.25 (d, *J* = 1.4 Hz, 1H), 7.23 – 7.16 (m, 6H), 7.13 – 7.08 (m, 3H), 6.36 (d, *J* = 1.4 Hz, 1H), 3.52 (s, 3H), 3.39 – 3.31 (m, 1H), 2.90 – 2.77 (m, 2H), 2.34 (dd, *J* = 14.4, 3.5 Hz, 1H), 1.97 (dd, *J* = 14.4, 6.2 Hz, 1H).

^{13}C NMR ($CDCl_3$, 101 MHz) δ = 147.2 (3C), 139.2, 136.9, 128.9 (6C), 128.0 (6C), 126.4 (3C), 118.2, 71.3, 64.9, 53.2, 33.5, 31.2.

HRMS (ESI^+) Found $[M+H]^+$ = 398.2227; $C_{26}H_{28}ON_3$ requires 398.2227, Δ 0.07 ppm.

Benzyl dibenzylglutaminate, S22

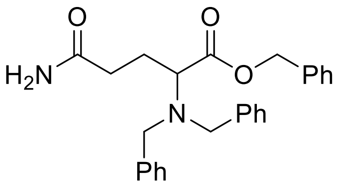

To a stirred solution of *DL*-glutamine (7.30 g, 50 mmol, 1.0 eq.) and K_2CO_3 (33.53 g, 243 mmol, 4.83 eq.) in water (350 mL) at RT was added benzyl bromide (23.75 mL, 200 mmol, 4.0 eq.) and the reaction left to stir for 12 d. The reaction mixture was concentrated *in vacuo* and purified by column chromatography (SiO_2, eluent load, CH_2Cl_2:MeOH, 96:4) to give the desired compound **S22** (4.46 g, 21%) as a colourless oil.

The spectral data matched that previously reported in the literature.[22]

^{1}H NMR ($CDCl_3$, 400 MHz) δ = 7.46 – 7.19 (m, 15H), 5.31 – 5.13 (m, 2H), 4.98 (d, *J* = 5.1 Hz, 1H), 3.88 (d, *J* = 13.7 Hz, 2H), 3.53 (d, *J* = 13.7 Hz, 2H), 3.37 (dd, *J* = 8.8, 5.8 Hz, 1H), 2.34 – 2.20 (m, 1H), 2.18 – 1.98 (m, 3H).

^{13}C NMR ($CDCl_3$, 101 MHz) δ = 174.6, 172.3, 139.8, 139.5 (2C), 136.1, 129.2 (4C), 128.9, 128.8 (2C), 128.7 (2C), 128.6, 128.5 (4C), 128.4, 127.3 (2C), 127.0, 66.4, 60.3, 58.1, 54.7 (2C), 32.3, 25.2.

4-(Dibenzylamino)-5-hydroxypentanamide, S23

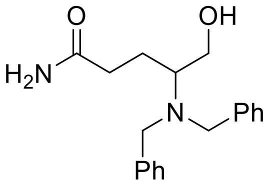

Glutamine derivative **S22** (1.75 g, 4 mmol, 1.0 eq.) was subjected to **General Procedure E**. Purification by column chromatography (SiO_2, eluent load, CH_2Cl_2:MeOH, 97:3 to 95:5) gave the desired alcohol **S23** (0.90 g, 73%) as an off-white powder.

m.p. = 95–97 °C.

IR (film) ν_{max}/cm^{-1} = 3308, 2932, 1679, 1636, 1451, 1407, 738, 696.

^{1}H NMR ($CDCl_3$, 400 MHz) δ = 7.39 – 7.16 (m, 10H), 5.55 (br. s, 1H), 3.80 (d, *J* = 13.3 Hz, 2H), 3.60 – 3.45 (m, 4H), 2.77 (d, *J* = 11.3 Hz, 1H), 2.26 – 2.04 (m, 3H), 1.64 – 1.51 (m, 1H).

^{13}C NMR ($CDCl_3$, 101 MHz) δ = 175.0, 139.2 (2C), 129.3 (4C), 128.6 (4C), 127.4 (2C), 60.9, 58.9, 53.5 (2C), 32.9, 21.4.

HRMS (ESI$^+$) Found $[M+H]^+$ = 313.1908; $C_{19}H_{25}O_2N_2$ requires 313.1911, Δ –0.83 ppm.

5-(Dibenzylamino)tetrahydro-2H-pyran-2-one, S24

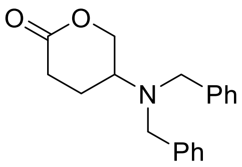

A stirred solution of glutamine derived alcohol **S23** (420 mg, 1.37 mmol, 1.0 eq.) in toluene (40 mL) was heated to reflux for 21 h. The mixture was then concentrated *in vacuo* and purified by column chromatography (SiO_2, eluent load, pentane:EtOAc, 70:30) to give the desired lactone **S24** (281 mg, 69%) as a colourless oil.

IR (film) ν_{max}/cm^{-1} = 3027, 1733, 1454, 1247, 1171, 1138, 1055, 749.

^{1}H NMR ($CDCl_3$, 400 MHz) δ = 7.41 – 7.21 (m, 10H), 4.38 – 4.24 (m, 2H), 3.75 – 3.62 (m, 4H), 3.24

(dtd, J = 9.1, 6.9, 5.6 Hz, 1H), 2.64 (ddd, J = 16.9, 6.3, 4.9 Hz, 1H), 2.42 (ddd, J = 16.9, 10.4, 6.9 Hz, 1H), 2.11 (ddd, J = 13.7, 11.7, 6.3 Hz, 1H), 2.03 – 1.91 (m, 1H).

^{13}C NMR ($CDCl_3$, 101 MHz) δ = 172.0, 139.3 (2C), 128.6 (4C), 128.5 (4C), 127.4 (2C), 69.0, 54.5 (2C), 52.0, 29.1, 20.4.

HRMS (ESI$^+$) Found $[M+H]^+$ = 296.1645; $C_{19}H_{22}O_2N$ requires 296.1645, Δ –0.19 ppm.

4-(Dibenzylamino)-5-hydroxypentanoic acid, 8f

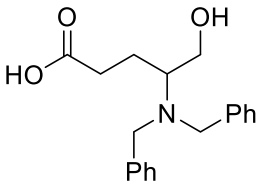

To a stirred solution of lactone **S24** (249 mg, 0.84 mmol, 1.0 eq.) in methanol (25 mL) was added a solution of NaOH (151 mg, 3.78 mmol, 4.5 eq.) in water (4.5 mL). The reaction was then heated to 60 °C and left to stir for 3 h. Upon cooling to RT, the reaction mixture was neutralised by the dropwise addition of 3M aq. HCl and concentrated *in vacuo*. Purification by column chromatography (SiO_2, eluent load, CH_2Cl_2:MeOH, 95:5) gave the desired alcohol **8f** (194 mg, 76%) as a colourless solid along with an inseparable impurity detectable by ^{13}C NMR.

m.p. = 95–97 °C.

IR (film) ν_{max}/cm^{-1} = 2806, 1727, 1495, 1454, 1245, 1216, 1174, 750, 699.

^{1}H NMR ($CDCl_3$, 400 MHz) δ = 8.28 (br. s), 7.37 – 7.03 (m, 10H), 3.61 (d, J = 13.8 Hz, 2H), 3.49 (d, J = 13.8 Hz, 2H), 3.47 – 3.41 (m, 2H), 2.68 – 2.55 (m, 1H), 2.21 (dt, J = 15.2, 7.1 Hz, 1H), 2.15 – 2.05 (m, 1H), 1.90 – 1.77 (m, 1H), 1.62 (dt, J = 14.6, 7.1 Hz, 1H).

^{13}C NMR ($CDCl_3$, 101 MHz) δ = 177.9, 136.7 (2C), 129.9 (4C), 128.8 (4C), 128.0 (2C), 60.9, 59.9, 53.8 (2C), 33.2, 21.4.

HRMS (ESI$^+$) Found $[M+H]^+$ = 314.1750; $C_{19}H_{24}O_3N$ requires 314.1751, Δ –0.34 ppm.

4.3 Hydrogen Borrowing Alkylations of 1,2-Amino Alcohols

(*S*)-3-(1-Benzylpyrrolidin-2-yl)-1-(2,3,4,5,6-pentamethylphenyl)propan-1-one, 3a

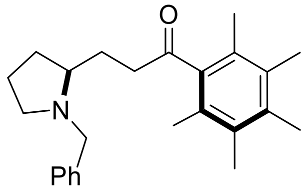

Commercially available (*S*)-*N*-benzyl-*L*-prolinol **1a** (96 mg, 0.5 mmol, 1.0 eq.) was subjected to **General Procedure A** at 85 °C. Purification by column chromatography (SiO_2, eluent load, CH_2Cl_2:MeOH, 95:5) gave the desired compound **3a** (131 mg, 72%, 94:6 e.r.) as a tan solid.

m.p. = 75–77 °C.

IR (film) ν_{max}/cm^{-1} = 2943, 2788, 1699, 1495, 1452, 1407, 1379, 1118, 737.

^{1}H NMR ($CDCl_3$, 400 MHz) δ = 7.40 – 7.20 (m, 5H), 4.12 (d, *J* = 13.0 Hz, 1H), 3.25 (d, *J* = 13.0 Hz, 1H), 2.94 (t, *J* = 8.4 Hz, 1H), 2.91 – 2.82 (m, 1H), 2.77 – 2.64 (m, 1H), 2.58 (s, 1H), 2.25 (s, 3H), 2.24 – 2.14 (m, 8H), 2.12 (s, 6H), 1.99 – 1.88 (m, 1H), 1.83 (ddd, *J* = 13.7, 9.1, 4.8 Hz, 1H), 1.68 (ddtd, *J* = 12.4, 9.1, 6.5, 3.5 Hz, 2H), 1.60 – 1.45 (m, 1H).

^{13}C NMR ($CDCl_3$, 101 MHz) δ = 212.1, 141.0, 135.5, 133.2 (2C), 129.1 (2C), 128.3 (2C), 127.4 (2C), 127.0 (2C), 63.3, 58.5, 54.2, 41.9, 30.0, 26.8, 22.2, 17.4 (2C), 16.8, 16.1 (2C).

HRMS (ESI$^+$) Found $[M+H]^+$ = 364.2635; $C_{25}H_{34}ON$ requires 364.2635, Δ –0.01 ppm.

$[\alpha]_D^{25}$ = –67.2 (c = 1.00, $CHCl_3$).

Chiral HPLC (Chiralpak IA with guard, 1.0 % IPA, 99.0 % hexane, 1.0 mL/min, 25 °C, λ = 254 nm, 10 µL injection).

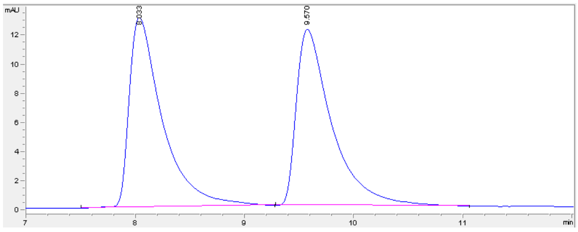

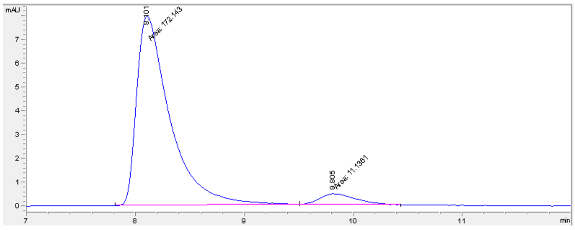

#	Time	Type	Area	Height	Width	Area%	Symmetry
1	8.033	BB	278.3	13.1	0.3101	50.151	0.403
2	9.57	BB	276.6	12.1	0.3377	49.849	0.41

#	Time	Type	Area	Height	Width	Area%	Symmetry
1	8.101	MF	172.1	8	0.3603	93.924	0
2	9.805	FM	11.1	4.7E-1	0.3923	6.076	0.595

571 mg of **3a** was recrystallised from ~2 mL of boiling MeOH to give 506 mg (89% recovery) of **3a**, the e.r. of which was measured to be 98:2 by HPLC analysis.

Chiral HPLC (Chiralpak IA with guard, 1.0 % IPA, 99.0 % hexane, 1.0 mL/min, 25 °C, λ = 254 nm, 10 µL injection).

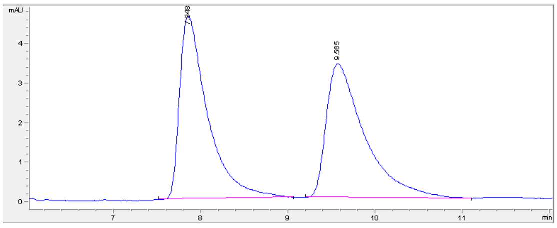

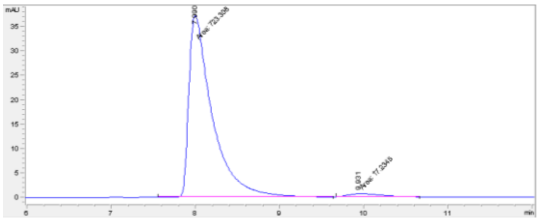

#	Time	Type	Area	Height	Width	Area%	Symmetry
1	7.848	BB	103.5	4.6	0.3214	49.963	0.405
2	9.565	BB	103.7	3.4	0.4342	50.037	0.398

#	Time	Type	Area	Height	Width	Area%	Symmetry
1	7.99	MM	723.3	37.2	0.324	97.673	0.355
2	9.931	MM	17.2	6.1E-1	0.4673	2.327	0.422

(*S*)-4-(Isoindolin-2-yl)-1-(2,3,4,5,6-pentamethylphenyl)pentan-1-one, 6a

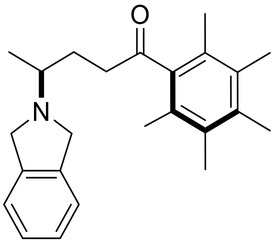

Xylyl protected alaninol **4a** (89 mg, 0.5 mmol, 1.0 eq.) was subjected to **General Procedure A** at 85 °C. Purification by column chromatography (SiO_2, eluent load, pentane:Et_2O, 70:30) gave the desired compound **6a** (20 mg, 11%, 54:46 e.r.) as a dark brown gum.

IR (film) ν_{max}/cm^{-1} = 2980, 1697, 1454, 1381, 910, 732.

^{1}**H NMR** ($CDCl_3$, 400 MHz) δ = 7.24 – 7.15 (m, 4H), 3.99 (s, 4H), 2.95 – 2.71 (m, 3H), 2.23 (s, 3H), 2.18 (s, 6H), 2.11 (s, 6H), 2.08 – 2.00 (m, 1H), 1.98 – 1.90 (m, 1H), 1.15 (d, *J* = 6.4 Hz, 3H).

^{13}**C NMR** ($CDCl_3$, 101 MHz) δ = 212.5, 141.1, 140.2 (2C), 135.4, 133.2 (2C), 127.4 (2C), 126.8 (2C), 122.5 (2C), 56.9, 56.1 (2C), 41.5, 28.7, 17.3 (2C), 17.1, 16.8, 16.1 (2C).

HRMS (ESI^+) Found $[M+H]^+$ = 350.2480; $C_{24}H_{32}ON$ requires 250.2478, Δ 0.50 ppm.

$[\alpha]_D^{25}$ = +1.4 (c = 1.00, $CHCl_3$).

Chiral HPLC (Chiralpak IA with guard, 5.0 % IPA, 95.0 % hexane, 1.0 mL/min, 25 °C, λ = 254 nm, 10 µL injection).

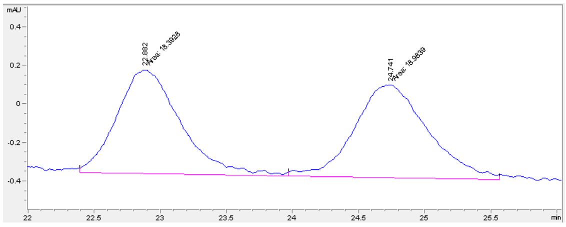

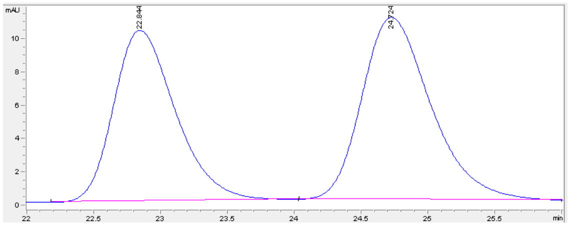

#	Time	Type	Area	Height	Width	Area%	Symmetry
1	22.882	MF	18.4	5.4E-1	0.564	49.209	0
2	24.741	FM	19	4.9E-1	0.6513	50.791	0.936

#	Time	Type	Area	Height	Width	Area%	Symmetry
1	22.844	BB	331.8	10.3	0.4916	45.608	0.707
2	24.724	BB	395.7	11	0.5358	54.392	0.714

(*S*)-4-(Dibenzylamino)-1-(2,3,4,5,6-pentamethylphenyl)pentan-1-one, 6b

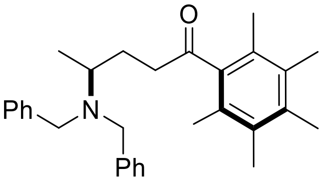

Dibenzyl protected alaninol **4b** (102 mg, 0.4 mmol, 1.0 eq.) was subjected to **General Procedure A** at 85 °C. Purification by column chromatography (SiO_2, eluent load, pentane:Et_2O, 95:5) gave the desired compound **6b** (120 mg, 70%, 73:27 e.r.) as a pale brown solid.

m.p. = 70–72 °C.

IR (film) ν_{max}/cm^{-1} = 2980, 1698, 1454, 1381, 1154, 1072, 746, 727, 698.

^{1}**H NMR** ($CDCl_3$, 400 MHz) δ = 7.38 – 7.16 (m, 10H), 3.76 (d, *J* = 13.8 Hz, 2H), 3.40 (d, *J* = 13.8 Hz, 2H),

3.00 (ddd, *J* = 18.4, 10.7, 4.7 Hz, 1H), 2.82 – 2.69 (m, 1H), 2.56 (ddd, *J* = 18.4, 10.6, 5.1 Hz, 1H), 2.25 (s, 3H), 2.19 (s, 6H), 2.10 – 1.98 (m, 7H), 1.71 (ddt, *J* = 14.3, 10.7, 5.1 Hz, 1H), 1.06 (d, *J* = 6.6 Hz, 3H).

^{13}C NMR ($CDCl_3$, 101 MHz) δ = 212.7, 141.1, 140.5 (2C), 135.4, 133.2 (2C), 128.8 (4C), 128.3 (4C), 127.5 (2C), 126.9 (2C), 53.4 (2C), 52.4, 43.3, 27.5, 17.3 (2C), 16.8, 16.1 (2C), 13.3.

HRMS (ESI^+) Found $[M+H]^+$ = 428.2944; $C_{30}H_{38}ON$ requires 428.2948, Δ –0.91 ppm.

$[\alpha]_D^{25}$ = –3.6 (c = 1.00, $CHCl_3$).

Chiral HPLC (Chiralpak IA with guard, 1.0 % IPA, 99.0 % hexane, 1.0 mL/min, 25 °C, λ = 254 nm, 10 µL injection).

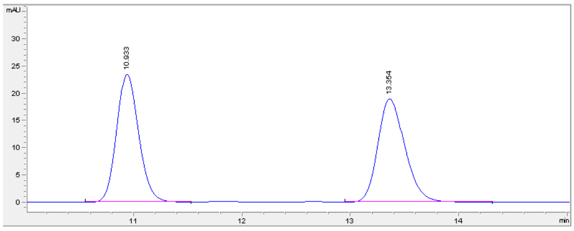

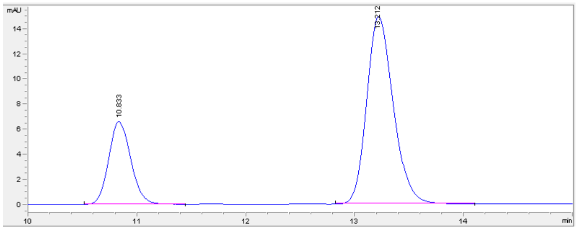

#	Time	Type	Area	Height	Width	Area%	Symmetry
1	10.933	BB	328.9	23.5	0.2151	50.095	0.835
2	13.354	BB	327.7	19	0.2652	49.905	0.757

#	Time	Type	Area	Height	Width	Area%	Symmetry
1	10.833	BB	92	6.6	0.2123	26.537	0.85
2	13.212	BB	254.8	14.9	0.2608	73.463	0.772

(*S*)-1-(2,3,4,5,6-Pentamethylphenyl)-4-(tritylamino)pentan-1-one, 6c and 3c

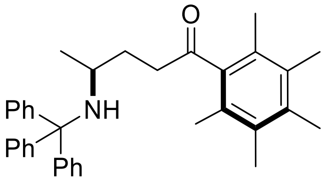

Tritylated alaninol **4c** (127 mg, 0.4 mmol, 1.0 eq.) was subjected to **General Procedure A** at 85 °C. Purification by column chromatography (SiO_2, eluent load, pentane:Et_2O, 95:5 to 90:10) gave the desired compound **6c** (155 mg, 79%, 96:4 e.r.) as a fluffy yellow solid.

Tritylated alaninol **4c** (1.27 g, 4 mmol, 1.0 eq.) was subjected to **General Procedure A**. Purification by column chromatography (SiO_2, eluent load, pentane:Et_2O, 95:5 to 90:10) gave the desired compound **3c** (1.67 g, 85%, 96:4 e.r.) as a fluffy yellow solid.

m.p. = 58–60 °C.

IR (film) ν_{max}/cm^{-1} = 2928, 1697, 1489, 1447, 1216, 748, 706.

^{1}H NMR ($CDCl_3$, 400 MHz) δ = 7.67 – 7.57 (m, 6H), 7.31 (t, *J* = 7.6 Hz, 6H), 7.26 – 7.19 (m, 3H), 2.81 – 2.64 (m, 2H), 2.55 (ddd, *J* = 17.8, 10.2, 5.8 Hz, 1H), 2.30 (s, 3H), 2.24 (s, 6H), 2.12 (s, 6H), 1.66 – 1.57 (m, 2H), 0.73 (d, *J* = 6.4 Hz, 3H).

^{13}C NMR ($CDCl_3$, 101 MHz) δ = 212.0, 147.1 (3C), 140.9, 135.3, 133.1 (2C), 128.8 (6C), 127.8 (6C), 127.3 (2C), 126.3 (3C), 71.5, 47.9, 42.2, 32.0, 21.8, 17.4 (2C), 16.8, 16.0 (2C).

HRMS (ESI^+) Found $[M+Na]^+$ = 512.2925; $C_{35}H_{39}ONNa$ requires 512.2924, Δ 0.29 ppm.

$[\alpha]_D^{25}$ = –27.2 (c = 1.00, $CHCl_3$).

Chiral HPLC (Chiralpak IA with guard, 1.0 % IPA, 99.0 % hexane, 1.0 mL/min, 25 °C, λ = 254 nm, 10 µL injection).

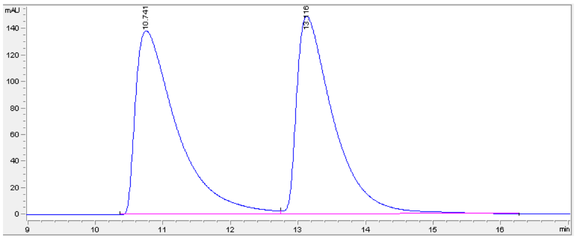

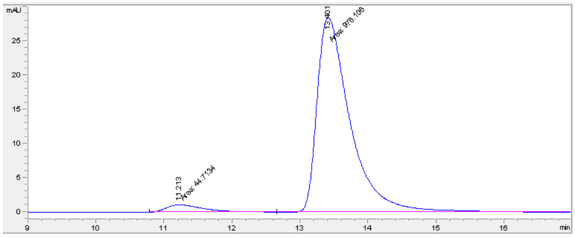

#	Time	Type	Area	Height	Width	Area%	Symmetry
1	10.741	BV	5896.7	138.1	0.6282	51.049	0.307
2	13.116	VB	5654.5	148.8	0.5614	48.951	0.34

#	Time	Type	Area	Height	Width	Area%	Symmetry
1	11.213	MF	44.7	1.1	0.6717	4.380	0.478
2	13.401	FM	976.1	28.7	0.5668	95.620	0.478

4-(Isoindolin-2-yl)-1-(2,3,4,5,6-pentamethylphenyl)butan-1-one, 7a

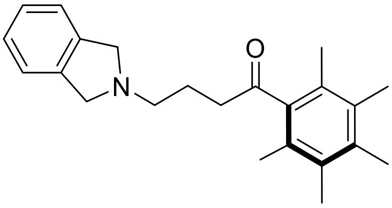

Xylyl protected deuterated glycinol **5a** (99 mg, 0.6 mmol, 1.0 eq.) was subjected to **General Procedure A** at 85 °C. Purification by column chromatography (SiO_2, eluent load, pentane:Et_2O, 60:40) gave the desired compound **7a** (72 mg, 36%, <10% D incorporation) as a pale brown solid.

m.p. = 74–76 °C.

IR (film) ν_{max}/cm^{-1} = 2929, 1694, 1447, 1355, 1144, 1133, 742, 701.

^{1}H NMR ($CDCl_3$, 400 MHz) δ = 7.20 (s, 4H), 3.97 (s, 4H), 2.83 (td, *J* = 7.2, 4.7 Hz, 4H), 2.23 (s, 3H), 2.18 (s, 6H), 2.11 (s, 6H), 2.03 (dd, *J* = 8.2, 5.9 Hz, 2H).

^{13}C NMR ($CDCl_3$, 101 MHz) δ = 212.0, 140.8, 140.0 (2C), 135.5, 133.2 (2C), 127.4 (2C), 126.9 (2C), 122.4 (2C), 59.1 (2C), 55.2, 43.1, 22.5, 17.3 (2C), 16.8, 16.1 (2C).

HRMS (ESI$^+$) Found [M+H]$^+$ = 336.2318; $C_{23}H_{30}ON$ requires 336.2322, Δ –1.14 ppm.

4-(Dibenzylamino)-1-(2,3,4,5,6-pentamethylphenyl)butan-1-one-4,4-*d*$_2$, 7b

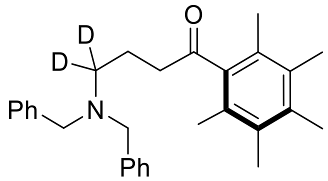

Dibenzyl protected deuterated glycinol **5b** (146 mg, 0.6 mmol, 1.0 eq.) was subjected to **General Procedure A** at 85 °C. Purification by column chromatography (SiO_2, eluent load, pentane:Et_2O, 92:8) gave the desired compound **7b** (198 mg, 80%, 70% D incorporation) as a pale orange solid.

m.p. = 69–71 °C.

IR (film) ν_{max}/cm^{-1} = 2789, 1700, 1452, 1123, 915, 749, 731, 698.

^{1}H NMR ($CDCl_3$, 400 MHz) δ = 7.42 – 7.21 (m, 10H), 3.61 (s, 4H), 2.72 (dd, *J* = 7.2, 1.5 Hz, 2H), 2.58 – 2.49 (m, 0.6H), 2.26 (s, 3H), 2.21 (s, 6H), 2.08 (s, 6H), 1.97 (t, *J* = 7.2 Hz, 2H).

^{13}C NMR ($CDCl_3$, 101 MHz) δ = 212.2, 140.9, 139.8 (2C), 135.4, 133.1 (2C), 128.9 (4C), 128.3 (4C), 127.4 (2C), 126.9 (2C), 58.3 (2C), 52.8, 43.3, 20.5, 17.3 (2C), 16.8, 16.1 (2C).

^{2}H NMR ($CHCl_3$, 77 MHz) δ = 2.50.

HRMS (ESI$^+$) Found [M+H]$^+$ = 416.2907; $C_{29}H_{34}D_2ON$ requires 416.2917, Δ –2.32 ppm.

1-(2,3,4,5,6-Pentamethylphenyl)-4-(tritylamino)butan-1-one-4,4-*d*$_2$, 7c

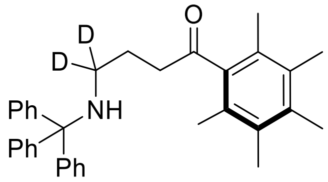

Trityl protected deuterated glycinol **5c** (183 mg, 0.6 mmol, 1.0 eq.) was subjected to **General Procedure A** at 85 °C. Purification by column chromatography (SiO_2, eluent load, pentane:Et_2O, 92:8) gave the desired compound **7c** (202 mg, 71%, >95% D incorporation) as a fluffy, off-white solid.

m.p. = 119–121 °C.

IR (film) ν_{max}/cm^{-1} = 2161, 1694, 1490, 1447, 1154, 1122, 744, 702.

^{1}H NMR ($CDCl_3$, 400 MHz) δ = 7.50 (d, *J* = 7.6 Hz, 6H), 7.30 (t, *J* = 7.6 Hz, 6H), 7.21 (t, *J* = 7.6 Hz, 3H), 2.80 (t, *J* = 7.3 Hz, 2H), 2.25 (s, 3H), 2.20 (s, 6H), 2.10 (s, 6H), 1.92 (t, *J* = 7.3 Hz, 2H).

^{13}C NMR ($CDCl_3$, 101 MHz) δ = 212.0, 146.2 (3C), 140.8, 135.5, 133.2 (2C), 128.7 (6C), 127.9 (6C), 127.4 (2C), 126.4 (3C), 71.0, 43.7, 43.3, 24.4, 17.4 (2C), 16.8, 16.1 (2C).

^{2}H NMR ($CHCl_3$, 77 MHz) δ = 2.22.

HRMS (ESI$^+$) Found $[M+Na]^+$ = 500.2890; $C_{34}H_{35}D_2ONNa$ requires 500.2893, Δ –0.63 ppm.

4-(Dibenzylamino)-1-(2,3,4,5,6-pentamethylphenyl)butan-1-one, 3b

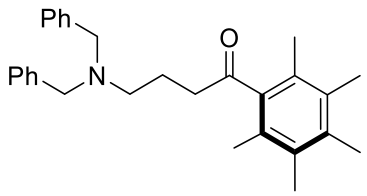

N,*N*-Dibenzyl ethanolamine **1b** (241 mg, 1.0 mmol, 1.0 eq.) was subjected to **General Procedure A** at 85 °C. Purification by column chromatography (SiO_2, eluent load, pentane:Et_2O, 92:8) gave the desired compound **3b** (305 mg, 74%) as a pale yellow solid.

m.p. = 68–70 °C.

IR (film) ν_{max}/cm^{-1} = 2929, 2797, 1698, 1494, 1453, 1381, 1125, 745, 699.

^{1}H NMR ($CDCl_3$, 400 MHz) δ = 7.36 – 7.17 (m, 10H), 3.56 (s, 4H), 2.70 (dd, *J* = 8.1, 6.9 Hz, 2H), 2.50 (t, *J* = 6.9 Hz, 2H), 2.22 (s, 3H), 2.16 (s, 6H), 2.04 (s, 6H), 1.95 (dq, *J* = 8.1, 6.9 Hz, 2H).

^{13}C NMR ($CDCl_3$, 101 MHz) δ = 212.2, 140.8, 139.7 (2C), 135.4, 133.1 (2C), 128.9 (4C), 128.3 (4C), 127.4 (2C), 126.9 (2C), 58.2 (2C), 52.7, 43.3, 20.7, 17.3 (2C), 16.8, 16.0 (2C).

HRMS (ESI^+) Found $[M+H]^+$ = 414.2783; $C_{29}H_{36}ON$ requires 414.2791, Δ –1.98 ppm.

(*R*)-6-Methyl-1-(2,3,4,5,6-pentamethylphenyl)-4-(tritylamino)heptan-1-one, 3d

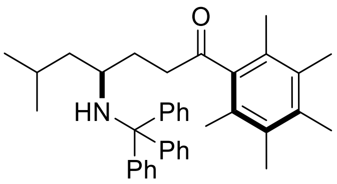

Tritylated leucinol **1d** (1.44 g, 4.0 mmol, 1.0 eq.) was subjected to **General Procedure A** at 85 °C. Purification by column chromatography (SiO_2, eluent load, toluene) gave the desired compound **3d** (1.32 g, 62%, 98:2 e.r.) as an exceptionally viscous oil.

IR (film) ν_{max}/cm^{-1} = 3057, 2954, 1698, 1596, 1490, 1448, 1407, 757, 707.

^{1}H NMR ($CDCl_3$, 400 MHz) δ = 7.56 – 7.47 (m, 6H), 7.27 – 7.20 (m, 6H), 7.19 – 7.13 (m, 3H), 2.90 – 2.76 (m, 1H), 2.56 (dt, *J* = 9.2, 4.6 Hz, 1H), 2.53 – 2.42 (m, 1H), 2.25 (s, 3H), 2.20 (s, 6H), 2.08 (s, 6H), 1.66 – 1.58 (m, 2H), 1.45 (br. s, 1H), 1.41 – 1.30 (m, 1H), 1.01 (ddd, *J* = 14.0, 9.2, 5.2 Hz, 1H), 0.58 (dd, *J* = 14.4, 6.6 Hz, 7H).

^{13}C NMR ($CDCl_3$, 101 MHz) δ = 212.5, 147.1 (3C), 141.0, 135.4, 133.2 (2C), 129.0 (6C), 127.9 (6C), 127.4 (2C), 126.4 (3C), 71.5, 49.5, 44.1, 40.9, 26.8, 25.0, 23.7, 21.8, 17.4 (2C), 16.8, 16.1 (2C).

HRMS ($APCI^-$) Found $[M–H]^-$ = 530.3423; $C_{38}H_{44}ON$ requires 530.3428, Δ –1.11 ppm.

$[\alpha]_D^{25}$ = –35.4 (c = 1.00, $CHCl_3$).

Chiral HPLC (Chiralpak IA with guard, 1.0 % IPA, 99.0 % hexane, 1.0 mL/min, 25 °C, λ = 254 nm, 10 µL injection).

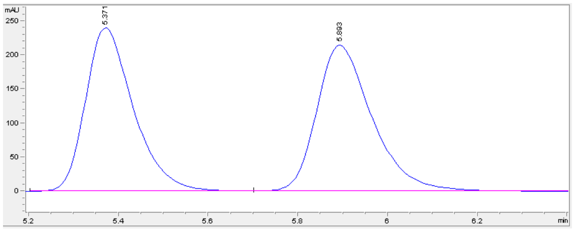

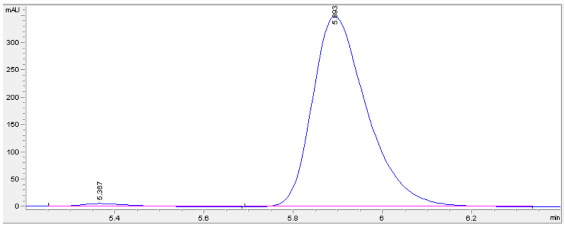

#	Time	Type	Area	Height	Width	Area%	Symmetry
1	5.371	BV	1782.3	240.5	0.1104	49.182	0.677
2	5.893	VB	1841.5	214.6	0.1301	50.818	0.664

#	Time	Type	Area	Height	Width	Area%	Symmetry
1	5.367	VB	48.3	5.8	0.123	1.602	0.645
2	5.893	BB	2965.2	349.5	0.129	98.398	0.652

(*R*)-1-(2,3,4,5,6-Pentamethylphenyl)-5-phenyl-4-(tritylamino)pentan-1-one, 3e

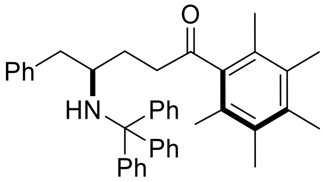

Tritylated phenylalaninol **1e** (118 mg, 0.3 mmol, 1.0 eq.) was subjected to **General Procedure A** at 110 °C. Purification by column chromatography (SiO_2, eluent load, pentane:Et_2O, 95:5 to 92:8) gave the desired compound **3e** (98 mg, 58%, 94:6 e.r.) as a fluffy yellow solid.

m.p. = 80–82 °C.

IR (film) ν_{max}/cm^{-1} = 3023, 2931, 1697, 1491, 1448, 746, 707.

^{1}H NMR ($CDCl_3$, 400 MHz) δ = 7.61 – 7.52 (m, 6H), 7.31 – 7.24 (m, 6H), 7.24 – 7.11 (m, 6H), 6.94 – 6.87 (m, 2H), 2.83 – 2.69 (m, 2H), 2.57 (ddd, *J* = 17.7, 11.4, 4.8 Hz, 1H), 2.39 – 2.24 (m, 5H), 2.21 (s, 6H), 2.06 (s, 6H), 1.68 – 1.54 (m, 1H), 1.42 (dddt, *J* = 14.3, 11.4, 4.8, 2.9 Hz, 1H).

^{13}C NMR ($CDCl_3$, 101 MHz) δ = 211.9, 147.0 (3C), 140.8, 139.4, 135.4, 133.1 (2C), 129.3 (2C), 128.9 (6C), 128.4 (2C), 127.9 (6C), 127.4 (2C), 126.5 (3C), 126.2, 71.5, 53.6, 41.8, 41.2, 27.2, 17.4 (2C), 16.8, 16.1 (2C).

HRMS (APCI$^-$) Found $[M–H]^-$ = 564.3268; $C_{41}H_{42}ON$ requires 564.3272, Δ –0.61 ppm.

$[\alpha]_D^{25}$ = –33.8 (c = 1.00, $CHCl_3$).

Chiral HPLC (Chiralpak IA with guard, 1.0 % IPA, 99.0 % hexane, 1.0 mL/min, 25 °C, λ = 254 nm, 10 µL injection).

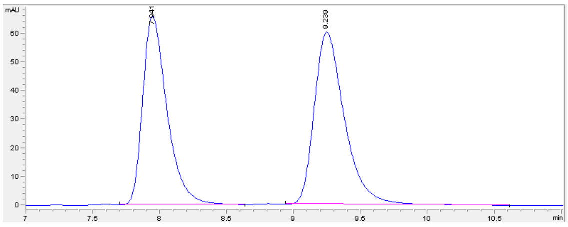

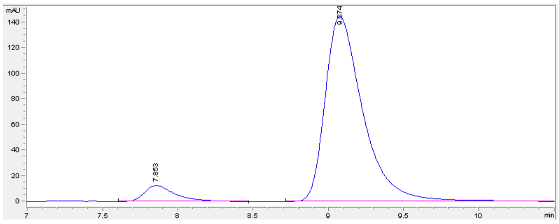

#	Time	Type	Area	Height	Width	Area%	Symmetry
1	7.941	BB	831.4	65.8	0.1913	46.903	0.618
2	9.239	BB	941.2	60	0.237	53.097	0.644

#	Time	Type	Area	Height	Width	Area%	Symmetry
1	7.853	BB	174.8	12.4	0.2109	6.443	0.574
2	9.074	BB	2538.4	144.6	0.2648	93.557	0.577

(*R*)-4-(Dibenzylamino)-5-methyl-1-(2,3,4,5,6-pentamethylphenyl)hexan-1-one, 3f

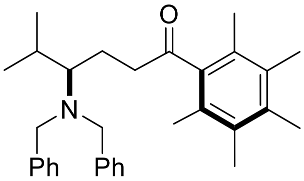

Dibenzylated valinol **1f** (113 mg, 0.4 mmol, 1.0 eq.) was subjected to **General Procedure A** at 110 °C. Purification by column chromatography (SiO_2, eluent load, pentane:Et_2O, 80:20) gave the desired compound **3a** (76 mg, 42%, 79:21 e.r.) as a thick, clear oil.

IR (film) ν_{max}/cm^{-1} = 2954, 1699, 1454, 747, 699.

^{1}H NMR ($CDCl_3$, 400 MHz) δ = 7.36 – 7.17 (m, 10H), 3.68 (s, 4H), 2.90 (ddd, *J* = 18.6, 10.8, 4.8 Hz, 1H), 2.57 (ddd, *J* = 18.6, 10.8, 5.1 Hz, 1H), 2.31 – 2.23 (m, 4H), 2.20 (d, *J* = 3.7 Hz, 6H), 2.11 – 2.00 (m, 8H), 1.83 – 1.70 (m, 1H), 0.98 (d, *J* = 6.7 Hz, 3H), 0.92 (d, *J* = 6.7 Hz, 3H).

^{13}C NMR ($CDCl_3$, 101 MHz) δ = 212.5, 141.0, 140.5 (2C), 135.4, 133.2 (2C), 129.0 (4C), 128.3 (4C), 127.4 (2C), 126.9 (2C), 62.5, 54.3 (2C), 44.8, 29.1, 22.0, 20.3, 20.2, 17.3 (2C), 16.8, 16.1 (2C).

HRMS (ESI$^+$) Found $[M+H]^+$ = 456.3261; $C_{32}H_{42}ON$ requires 456.3261, Δ 0.04 ppm.

$[\alpha]_D^{25}$ = –22.6 (c = 1.00, $CHCl_3$).

Chiral HPLC (Chiralpak IA with guard, 1.0 % IPA, 99.0 % hexane, 1.0 mL/min, 25 °C, λ = 254 nm, 10 μL injection).

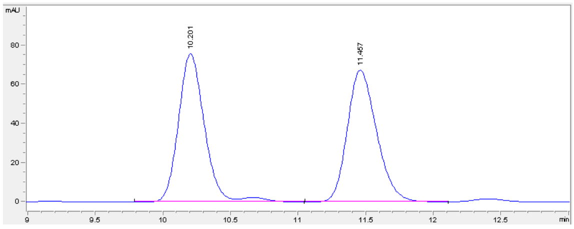

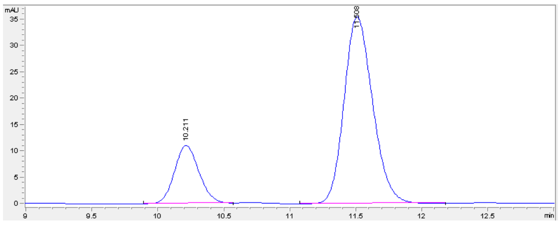

#	Time	Type	Area	Height	Width	Area%	Symmetry
1	10.201	BV R	1029.2	76.1	0.2032	50.191	0.837
2	11.457	BB	1021.4	67.6	0.2322	49.809	0.755

#	Time	Type	Area	Height	Width	Area%	Symmetry
1	10.211	BB	142	11	0.2002	20.950	0.874
2	11.508	BB	535.9	35.6	0.2319	79.050	0.8

(*R*)-1-(2,3,4,5,6-Pentamethylphenyl)-4-phenyl-4-(tritylamino)butan-1-one, 3g

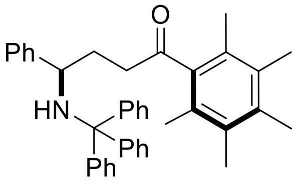

Tirtylated phenylglycinol **1g** (227 mg, 0.6 mmol, 1.0 eq.) was subjected to **General Procedure A** at 110 °C. Purification by column chromatography (SiO_2, eluent load, pentane:Et_2O, 95:5 to 92:8) gave the desired compound **3g** (159 mg, 48%, 93:7 e.r.) as a fluffy, pale-yellow solid.

m.p. = 70–72 °C.

IR (film) ν_{max}/cm^{-1} = 2981, 1699, 1448, 1382, 1154, 1072, 772, 746, 706.

^{1}H NMR ($CDCl_3$, 400 MHz) δ = 7.54 – 7.43 (m, 6H), 7.23 – 7.09 (m, 12H), 7.06 (dt, *J* = 6.1, 1.6 Hz, 2H), 3.51 (dd, *J* = 9.3, 3.9 Hz, 1H), 2.18 (s, 3H), 2.11 (s, 8H), 1.81 (s, 6H), 1.67 – 1.58 (m, 1H), 1.58 – 1.49 (m, 4H).

^{13}C NMR ($CDCl_3$, 101 MHz) δ = 211.5, 146.7 (3C), 145.4, 140.7, 135.3, 133.0 (2C), 129.1 (6C), 128.2 (2C), 127.8 (6C), 127.3 (2C), 127.2 (2C), 126.44 (3C), 126.39, 72.3, 57.7, 42.4, 32.4, 17.1 (2C), 16.7, 16.0 (2C).

HRMS (ESI$^+$) Found $[M+Na]^+$ = 574.3078; $C_{40}H_{41}ONNa$ requires 574.3080, Δ –0.34 ppm.

$[\alpha]_D^{25}$ = +14.6 (c = 1.00, $CHCl_3$).

Chiral HPLC (Chiralpak IA with guard, 1.0 % IPA, 99.0 % hexane, 1.0 mL/min, 25 °C, λ = 254 nm, 10 µL injection).

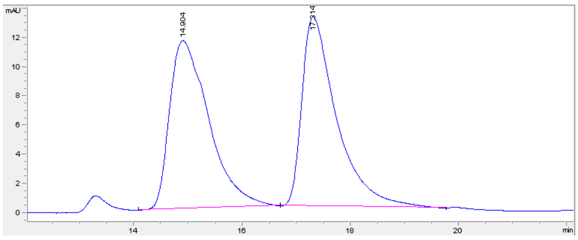

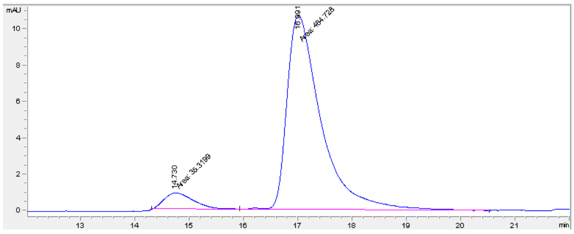

#	Time	Type	Area	Height	Width	Area%	Symmetry
1	14.904	BB	582.1	11.5	0.7145	50.047	0.485
2	17.314	BB	581	13	0.6425	49.953	0.443

#	Time	Type	Area	Height	Width	Area%	Symmetry
1	14.73	MF	35.3	9.1E-1	0.6497	7.063	0
2	16.991	FM	464.7	10.6	0.728	92.937	0.484

3-((4*R*,5*R*)-3-Benzyl-5-methyloxazolidin-4-yl)-1-(2,3,4,5,6-pentamethylphenyl)propan-1-one, 3h

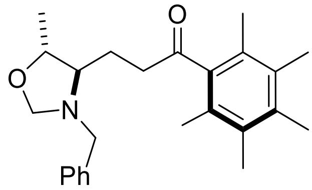

Benzyl protected cyclic threoninol **1h** (62 mg, 0.3 mmol, 1.0 eq.) was subjected to **General Procedure A** at 85 °C. Purification by column chromatography (SiO_2, eluent load, pentane:Et_2O, 80:20) gave the desired compound **3h** (64 mg, 56%, 99:1 e.r.) as a pale brown solid.

m.p. = 84–86 °C.

IR (film) ν_{max}/cm^{-1} = 2970, 2926, 1698, 1453, 1381, 1071, 998, 699.

^{1}H NMR ($CDCl_3$, 400 MHz) δ = 7.37 – 7.20 (m, 5H), 4.29 (s, 2H), 3.79 (s, 2H), 3.68 (p, *J* = 6.1 Hz, 1H), 2.88 (ddd, *J* = 18.5, 9.0, 5.7 Hz, 1H), 2.75 (ddd, *J* = 18.5, 8.8, 6.3 Hz, 1H), 2.63 (ddd, *J* = 7.8, 6.3, 5.1 Hz, 1H), 2.25 (s, 3H), 2.20 (s, 6H), 2.10 (s, 6H), 1.95 – 1.81 (m, 2H), 1.42 (d, *J* = 6.1 Hz, 3H).

^{13}C NMR ($CDCl_3$, 101 MHz) δ = 211.9, 140.7, 139.5, 135.5, 133.1 (2C), 128.7 (2C), 128.4 (2C), 127.3, 127.2 (2C), 85.0, 78.7, 70.3, 59.7, 42.3, 26.1, 20.4, 17.3 (2C), 16.7, 16.0 (2C).

HRMS (ESI^+) Found $[M+H]^+$ = 380.2585; $C_{25}H_{34}O_2N$ requires 380.2584, Δ 0.29 ppm.

$[\alpha]_D^{25}$ = –2.5 (c = 1.00, $CHCl_3$).

Chiral HPLC (Chiralpak IA with guard, 1.0 % IPA, 99.0 % hexane, 1.0 mL/min, 25 °C, λ = 210 nm, 10 µL injection).

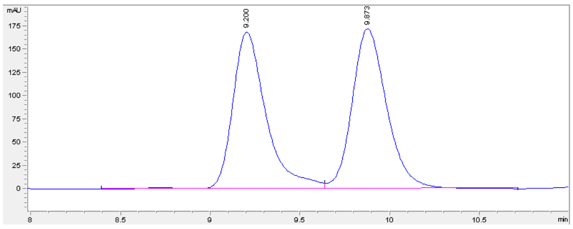

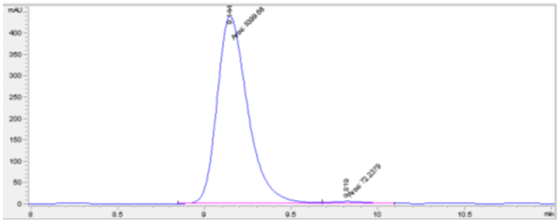

#	Time	Type	Area	Height	Width	Area%	Symmetry
1	9.2	VV R	2198.5	169.1	0.1942	48.716	0.693
2	9.873	VB	2314.3	172.6	0.2045	51.284	0.766

#	Time	Type	Area	Height	Width	Area%	Symmetry
1	9.144	MF	5399.7	439.5	0.2048	98.680	0
2	9.819	FM	72.2	5.2	0.2311	1.320	0.812

3-(3-Benzyloxazolidin-4-yl)-1-(2,3,4,5,6-pentamethylphenyl)propan-1-one, 3i

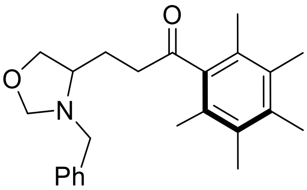

Benzyl protected cyclic serinol **1i** (116 mg, 0.6 mmol, 1.0 eq.) was subjected to **General Procedure A** at 85 °C. Purification by column chromatography (SiO_2, eluent load, pentane:Et_2O, 80:20 to 75:25) gave the desired compound **3i** (71 mg, 32%, 50:50 e.r.) as a clear oil.

IR (film) ν_{max}/cm^{-1} = 2980, 1697, 1454, 1383, 1154, 1073, 699.

^{1}H NMR ($CDCl_3$, 400 MHz) δ = 7.37 – 7.18 (m, 5H), 4.29 (s, 2H), 4.09 (dd, *J* = 8.0, 6.9 Hz, 1H), 3.73 (d, *J* = 1.8 Hz, 2H), 3.42 (dd, *J* = 8.0, 4.9 Hz, 1H), 3.18 (ddt, *J* = 8.7, 6.9, 5.3 Hz, 1H), 2.85 (ddd, *J* = 18.6, 8.7, 5.5 Hz, 1H), 2.72 (ddd, *J* = 18.6, 8.7, 6.3 Hz, 1H), 2.24 (s, 3H), 2.19 (s, 6H), 2.08 (s, 6H), 1.93 – 1.76 (m, 2H).

^{13}C NMR ($CDCl_3$, 101 MHz) δ = 211.9, 140.8, 139.3, 135.6, 133.2 (2C), 128.9 (2C), 128.5 (2C), 127.37, 127.35 (2C), 85.5, 69.5, 63.2, 59.1, 42.5, 27.1, 17.3 (2C), 16.8, 16.1 (2C).

HRMS (ESI$^+$) Found $[M+H]^+$ = 366.2429; $C_{24}H_{32}O_2N$ requires 366.2428, Δ 0.28 ppm.

Chiral HPLC (Chiralpak IA with guard, 1.0 % IPA, 99.0 % hexane, 1.0 mL/min, 25 °C, λ = 254 nm, 10 µL injection).

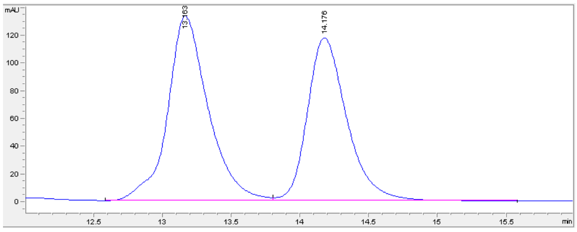

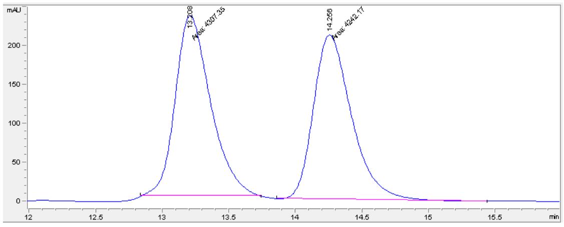

#	Time	Type	Area	Height	Width	Area%	Symmetry
1	13.163	BV	2748.9	133	0.3031	53.862	0.758
2	14.176	VB	2354.7	117.3	0.3041	46.138	0.706

#	Time	Type	Area	Height	Width	Area%	Symmetry
1	13.208	MM	4307.3	231.4	0.3103	50.381	0.716
2	14.256	FM	4242.2	210.8	0.3354	49.619	0.691

(*R*)-5-(4-Methoxyphenyl)-1-(2,3,4,5,6-pentamethylphenyl)-4-(tritylamino)pentan-1-one, 3j

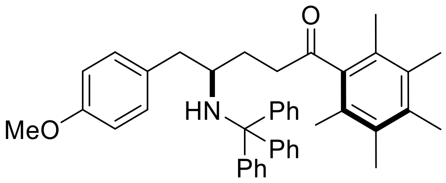

Trityl protected methyl tyrosinol **1j** (170 mg, 0.4 mmol, 1.0 eq.) was subjected to **General Procedure A** at 110 °C. Purification by column chromatography (SiO_2, eluent load, pentane:Et_2O, 85:15) gave the desired compound **3j** (127 mg, 53%, 90:10 e.r.) as a colourless and fluffy solid.

m.p. = 75–77 °C.

IR (film) ν_{max}/cm^{-1} = 2981, 2889, 1696, 1511, 1382, 1247, 1157, 954, 706.

^{1}H NMR ($CDCl_3$, 400 MHz) δ = 7.60 – 7.48 (m, 6H), 7.29 – 7.22 (m, 6H), 7.22 – 7.12 (m, 3H), 6.80 (dt, *J* = 8.7, 2.2 Hz, 2H), 6.70 (dt, *J* = 8.7, 2.2 Hz, 2H), 3.75 (s, 3H), 2.78 – 2.63 (m, 2H), 2.51 (ddd, *J* = 17.7, 11.5, 4.8 Hz, 1H), 2.31 – 2.22 (m, 4H), 2.22 – 2.16 (m, 7H), 2.03 (s, 6H), 1.62 – 1.50 (m, 1H), 1.39 (dddd, *J* = 14.5, 11.5, 5.0, 3.2 Hz, 1H).

^{13}C NMR ($CDCl_3$, 101 MHz) δ = 211.9, 158.1, 147.0 (3C), 140.8, 135.4, 133.2 (2C), 131.4, 130.2 (2C), 128.9 (6C), 127.9 (6C), 127.4 (2C), 126.5 (3C), 113.8 (2C), 71.5, 55.3, 53.7, 41.9, 40.3, 27.1, 17.4 (2C), 16.8, 16.1 (2C).

HRMS (ESI^+) Found $[M+Na]^+$ = 618.3341; $C_{42}H_{45}O_2NNa$ requires 618.3343, Δ –0.26 ppm.

$[\alpha]_D^{25}$ = –20.2 (c = 1.00, $CHCl_3$).

Chiral HPLC (Chiralpak IA with guard, 1.0 % IPA, 99.0 % hexane, 1.0 mL/min, 25 °C, λ = 254 nm, 10 µL injection).

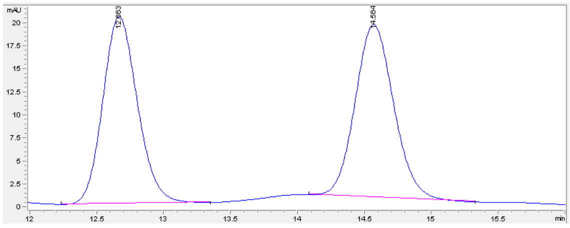

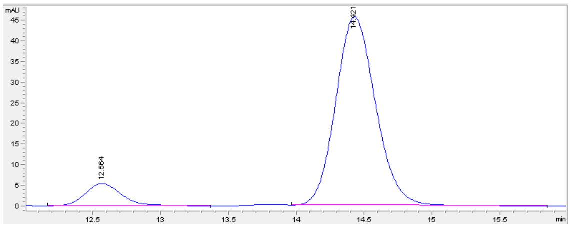

#	Time	Type	Area	Height	Width	Area%	Symmetry
1	12.663	BB	366.6	20.3	0.2763	49.314	0.843
2	14.564	BB	376.8	18.8	0.3082	50.686	0.875

#	Time	Type	Area	Height	Width	Area%	Symmetry
1	12.564	BB	99.8	5.4	0.2832	9.645	0.801
2	14.421	BB	934.6	45.8	0.316	90.355	0.845

(*S*)-4,8-Bis(dibenzylamino)-1-(2,3,4,5,6-pentamethylphenyl)octan-1-one, 3k

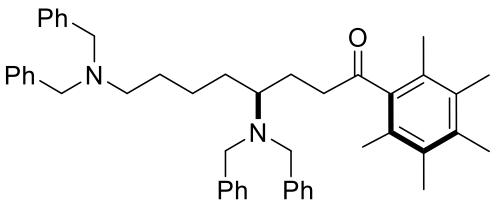

Benzyl protected lysinol **1k** (1.48 g, 3.0 mmol, 1.0 eq.) was subjected to **General Procedure A** at 85 °C. Purification by column chromatography (SiO_2, eluent load, pentane:Et_2O, 90:10) gave the desired compound **3k** (1.59 g, 80%, 85:15 e.r.) as a thick, clear oil.

IR (film) ν_{max}/cm^{-1} = 2933, 1698, 1494, 1453, 1028, 746, 698.

^{1}H NMR ($CDCl_3$, 400 MHz) δ = 7.41 – 7.14 (m, 20H), 3.62 (d, *J* = 13.7 Hz, 2H), 3.59 – 3.46 (m, 6H), 2.86 (ddd, *J* = 18.6, 10.2, 4.9 Hz, 1H), 2.55 – 2.33 (m, 4H), 2.25 (s, 3H), 2.19 (s, 6H), 2.03 (s, 7H), 1.74 – 1.57 (m, 2H), 1.49 – 1.25 (m, 3H), 1.14 (dt, *J* = 14.6, 7.2 Hz, 1H).

^{13}C NMR ($CDCl_3$, 101 MHz) δ = 212.6, 141.1, 140.6 (2C), 140.2 (2C), 135.4, 133.2 (2C), 129.0 (4C), 128.9 (4C), 128.3 (8C), 127.5 (2C), 126.9 (4C), 58.5 (2C), 57.2, 53.43 (2C), 53.38, 43.6, 29.3, 27.4, 24.8, 23.5, 17.3 (2C), 16.8, 16.1 (2C).

HRMS (ESI$^+$) Found $[M+H]^+$ = 665.4461; $C_{47}H_{57}ON_2$ requires 665.4465, Δ –0.65 ppm.

$[\alpha]_D^{25}$ = –0.8 (c = 1.00, $CHCl_3$).

Chiral HPLC (Chiralpak IA with guard, 5.0 % IPA, 95.0 % hexane, 1.0 mL/min, 25 °C, λ = 254 nm, 10 µL injection).

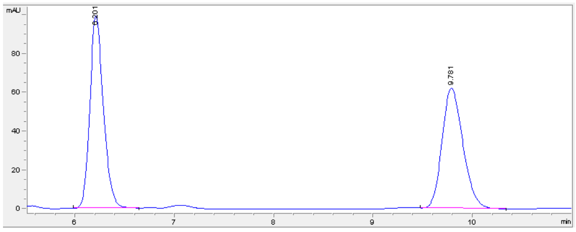

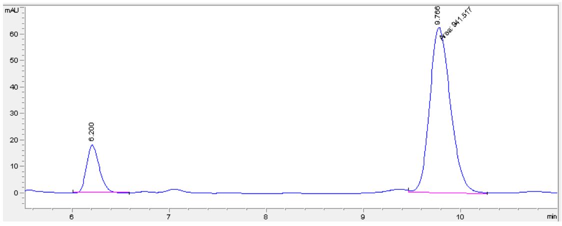

#	Time	Type	Area	Height	Width	Area%	Symmetry
1	6.201	BB	939.9	99	0.1448	51.009	0.757
2	9.781	BB	902.7	61.6	0.2268	48.991	0.772

#	Time	Type	Area	Height	Width	Area%	Symmetry
1	6.2	BB	167.5	18.3	0.141	15.102	0.755
2	9.766	MM	941.5	62.8	0.2497	84.898	0.78

(*R*)-5-(1-Methyl-1*H*-indol-3-yl)-1-(2,3,4,5,6-pentamethylphenyl)-4-(tritylamino)pentan-1-one, 3l

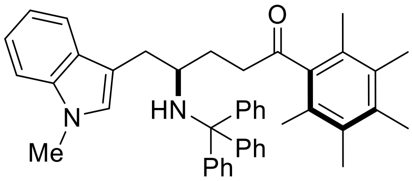

Trityl protected methyl tryptophanol **1l** (134 mg, 0.3 mmol, 1.0 eq.) was subjected to **General Procedure A** at 110 °C. Purification by column chromatography (SiO_2, eluent load, pentane:Et_2O:Et_3N, 85:15:0.5 to 80:20:0.5) gave the desired compound **3l** (95 mg, 51%, 95:5 e.r.) as a highly acid-sensitive, colourless, and fluffy solid.

m.p. = 89–91 °C.

IR (film) ν_{max}/cm^{-1} = 2931, 1696, 1486, 1448, 744, 708.

^{1}H NMR (CD_2Cl_2, 400 MHz) δ = 7.56 – 7.49 (m, 6H), 7.31 (dt, *J* = 8.0, 1.0 Hz, 1H), 7.27 – 7.21 (m, 7H), 7.20 – 7.14 (m, 4H), 6.98 (ddd, *J* = 8.0, 6.9, 1.1 Hz, 1H), 6.70 (s, 1H), 3.68 (s, 3H), 2.80 (dt, *J* = 15.4, 6.3 Hz, 1H), 2.73 – 2.61 (m, 2H), 2.55 (dd, *J* = 14.3, 5.8 Hz, 1H), 2.42 (ddd, *J* = 17.5, 11.6, 4.8 Hz, 1H), 2.24 (s, 3H), 2.17 (s, 6H), 1.94 (s, 6H), 1.44 (dddd, *J* = 13.8, 11.6, 7.2, 4.7 Hz, 1H), 1.32 (dddd, *J* = 12.6, 7.7, 5.3, 2.3 Hz, 1H).

^{13}**C NMR** (CD_2Cl_2, 101 MHz) δ = 212.1, 147.7 (3C), 141.2, 137.5, 135.6, 133.4 (2C), 129.6, 129.4 (6C), 128.3 (6C), 127.9, 127.7 (2C), 126.8 (3C), 121.9, 119.7, 119.0, 112.1, 109.6, 71.9, 53.4, 42.6, 33.0, 31.3, 28.2, 17.6 (2C), 16.9, 16.3 (2C).

HRMS (APCI$^-$) Found $[M–H]^-$ = 617.3531; $C_{44}H_{45}ON_2$ requires 617.3537, Δ –0.96 ppm.

$[\alpha]_D^{25}$ = –39.4 (c = 1.00, $CHCl_3$).

Chiral HPLC (Chiralpak IA with guard, 5.0 % IPA, 95.0 % hexane, 1.0 mL/min, 25 °C, λ = 254 nm, 10 µL injection).

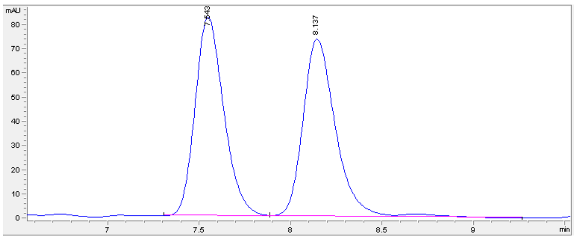

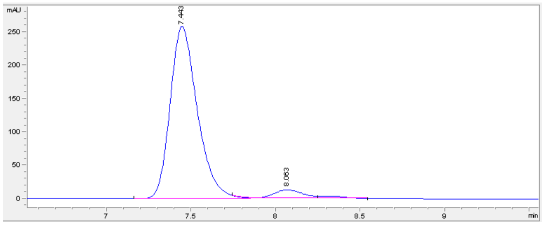

#	Time	Type	Area	Height	Width	Area%	Symmetry
1	7.543	BV	895.1	82.1	0.1672	49.447	0.768
2	8.137	VV R	915.1	73.3	0.1863	50.553	0.72

#	Time	Type	Area	Height	Width	Area%	Symmetry
1	7.443	BV R	2904.6	257.2	0.1702	94.938	0.724
2	8.063	VV E	154.9	12.9	0.1844	5.062	0.832

4-Methyl-1-(2,3,4,5,6-pentamethylphenyl)-4-(tritylamino)pentan-1-one, 3m

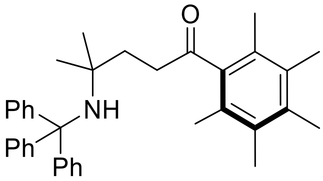

Trityl protected alcohol **1m** (331 mg, 1.0 mmol, 1.0 eq.) was subjected to **General Procedure A** at 110 °C. Purification by column chromatography (SiO_2, eluent load, pentane:Et_2O:Et_3N, 97:3:0.5) gave the desired compound **3m** (204 mg, 41%,) as an off-white solid.

m.p. = 58–60 °C.

IR (film) ν_{max}/cm^{-1} = 1701, 1487, 1446, 1176, 1047, 1030, 748, 699, 639.

^{1}**H NMR** ($CDCl_3$, 400 MHz) δ = 7.65 – 7.58 (m, 6H), 7.22 (dd, *J* = 8.4, 6.8 Hz, 6H), 7.18 – 7.09 (m, 3H), 2.91 (t, *J* = 8.1 Hz, 2H), 2.26 (s, 3H), 2.21 (s, 6H), 2.13 (s, 6H), 1.69 (t, *J* = 8.1 Hz, 2H), 1.63 (br. s, 1H), 0.69 (s, 6H).

^{13}**C NMR** ($CDCl_3$, 101 MHz) δ = 212.6, 148.4 (3C), 141.2, 135.5, 133.3 (2C), 129.3 (6C), 127.6 (6C), 127.5 (2C), 126.2 (3C), 70.0, 54.2, 41.4, 39.9, 28.5 (2C), 17.5 (2C), 16.8, 16.1 (2C).

HRMS (ESI^-) Found $[M-H]^-$ = 502.3107; $C_{36}H_{40}ON$ requires 502.3104, Δ 0.43 ppm.

1-(2,3,4,5,6-Pentamethylphenyl)-3-(1-(tritylamino)cyclopentyl)propan-1-one, 3n

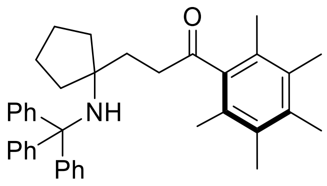

Trityl protected cycloleucinol **1n** (183 mg, 0.6 mmol, 2.0 eq.) was subjected to **General Procedure A** at 110 °C, with pentamethylacetophenone **2** (1.0 eq.) as the limiting reagent. Purification by column chromatography (SiO_2, eluent load, pentane:Et_2O:Et_3N, 95:5:0.5) gave the desired compound **3n** (215 mg, 68%) as a colourless solid.

m.p. = 149–151 °C.

IR (film) ν_{max}/cm^{-1} = 2970, 2871, 2161, 2027, 1698, 1485, 1447, 1309, 709, 694, 652.

^{1}**H NMR** ($CDCl_3$, 400 MHz) δ = 7.59 – 7.52 (m, 6H), 7.24 – 7.19 (m, 6H), 7.18 – 7.12 (m, 3H), 2.79 (d, *J* = 8.0 Hz, 2H), 2.25 (s, 3H), 2.20 (s, 6H), 2.09 (s, 6H), 1.59 – 1.48 (m, 2H), 1.48 – 1.38 (m, 4H), 1.33 (d, *J* = 8.0 Hz, 2H), 1.27 – 1.19 (m, 2H).

^{13}**C NMR** ($CDCl_3$, 101 MHz) δ = 212.4, 148.3 (3C), 141.2, 135.5, 133.2 (2C), 129.2 (6C), 127.7 (6C), 127.4 (2C), 126.3 (3C), 70.1, 65.4, 41.8, 38.3 (2C), 35.1, 24.5 (2C), 17.4 (2C), 16.8, 16.1 (2C).

HRMS (ESI^+) Found $[M+Na]^+$ = 552.3239; $C_{38}H_{44}ONNa$ requires 552.3237, Δ 0.36 ppm.

4.4 Acid-Mediated Cleavage

(*S*)-3-(1-benzylpyrrolidin-2-yl)propanoic acid hydrochloride, 9a

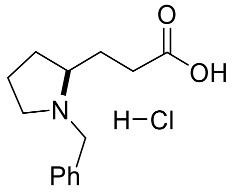

Proline-derived hydrogen borrowing product **3a** (73 mg, 0.2 mmol, 1.0 eq.) was subjected to **General Procedure B** to give the desired compound **9a** (52 mg, 97%) as a pale yellow solid.

m.p. = 160–162 °C.

IR (film) ν_{max}/cm^{-1} = 3345, 2963 (br.), 1619, 1543, 1461, 1378, 1071, 1049, 719, 645.

^{1}H NMR (D_2O, 400 MHz) δ = 7.53 – 7.48 (m, 5H), 4.49 (d, *J* = 13.0 Hz, 1H), 4.24 (d, *J* = 13.0 Hz, 1H), 3.59 (dtd, *J* = 10.2, 8.0, 4.3 Hz, 1H), 3.43 (ddd, *J* = 11.7, 7.6, 5.5 Hz, 1H), 3.26 (dt, *J* = 11.7, 8.0 Hz, 1H), 2.51 – 2.29 (m, 3H), 2.16 – 1.88 (m, 3H), 1.88 – 1.70 (m, 2H).

^{13}C NMR (D_2O, 101 MHz) δ = 176.5, 130.6 (2C), 130.1, 129.9, 129.4 (2C), 66.7, 57.5, 54.1, 30.2, 28.9, 26.1, 21.4.

HRMS (ESI$^+$) Found [M+H]$^+$ = 234.1490; $C_{14}H_{20}O_2N$ requires 234.1489, Δ 0.50 ppm.

$[\alpha]_D^{25}$ = –18.1 (c = 1.00, MeOH).

4-(Dibenzylamino)butanoic acid hydrochloride, 9b

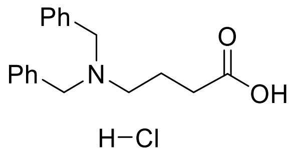

Glycine-derived hydrogen borrowing product **3b** (41 mg, 0.1 mmol, 1.0 eq.) was subjected to **General Procedure B** to give the desired compound **9b** (32 mg, 99%) as a thick, clear oil.

The spectral data matched that previously reported in the literature.[23]

IR (film) ν_{max}/cm^{-1} = 2950 (br.), 1720, 1456, 1210, 1177, 741, 699.

^{1}H NMR (D_2O, 400 MHz) δ = 7.58 – 7.40 (m, 10H), 4.38 (d, *J* = 5.9 Hz, 4H), 3.16 (dt, *J* = 8.2, 5.9 Hz, 2H), 2.38 (t, *J* = 6.9 Hz, 2H), 2.10 – 1.98 (m, 2H).

^{13}C NMR (D_2O, 101 MHz) δ = 176.7, 131.0 (4C), 130.2 (2C), 129.4 (4C), 129.0, 57.2 (2C), 51.3, 30.5, 18.6.

HRMS (ESI$^+$) Found [M+H]$^+$ = 284.1642; $C_{18}H_{22}O_2N$ requires 284.1645, Δ –1.24 ppm.

(*S*)-4-Aminopentanoic acid hydrochloride, 9c

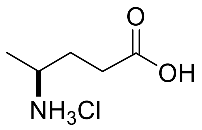

Alanine-derived hydrogen borrowing product **3c** (49 mg, 0.1 mmol, 1.0 eq.) was subjected to **General Procedure B** to give the desired compound **9c** (15 mg, 99%) as a colourless powder.

m.p. = 155–157 °C.

IR (film) v_{max}/cm^{-1} = 2940 (br.), 1714, 1603, 1508, 1407, 1376, 1191, 1131, 800, 649.

^{1}H NMR (D_2O, 400 MHz) δ = 3.41 (h, *J* = 6.7 Hz, 1H), 2.52 (dt, *J* = 8.2, 6.7 Hz, 2H), 1.99 (dddd, *J* = 14.1, 8.3, 7.0, 5.8 Hz, 1H), 1.84 (dtd, *J* = 14.7, 8.0, 5.8 Hz, 1H), 1.30 (d, *J* = 6.7 Hz, 3H).

^{13}C NMR (D_2O, 101 MHz) δ = 177.0, 47.1, 29.8, 28.9, 17.3.

HRMS (ESI$^+$) Found [M+H]$^+$ = 118.0865; $C_5H_{12}O_2N$ requires 118.0863, Δ 1.75 ppm.

$[α]_D^{25}$ = –4.0 (c = 1.00, MeOH).

(*R*)-4-Amino-6-methylheptanoic acid hydrochloride, 9d

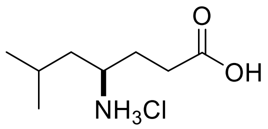

Leucine-derived hydrogen borrowing product **3d** (266 mg, 0.5 mmol, 1.0 eq.) was subjected to **General Procedure B** to give the desired compound **9d** (79 mg, 81%) as a colourless powder.

The spectral data matched that previously reported in the literature.[24]

m.p. = 144–146 °C.

IR (film) ν_{max}/cm^{-1} = 2981 (br.), 2888, 1700, 1385, 1245, 1157, 953, 705.

^{1}H NMR (D_2O, 400 MHz) δ = 3.39 (p, *J* = 6.7 Hz, 1H), 2.55 (t, *J* = 7.5 Hz, 2H), 2.05 – 1.85 (m, 2H), 1.70 (dh, *J* = 13.3, 6.7 Hz, 1H), 1.58 – 1.44 (m, 2H), 0.93 (dd, *J* = 6.7, 3.1 Hz, 6H).

^{13}C NMR (D_2O, 101 MHz) δ = 177.1, 49.4, 40.9, 29.5, 27.4, 23.8, 21.7, 21.2.

HRMS (ESI$^+$) Found [M+H]$^+$ = 160.1332; $C_8H_{18}O_2N$ requires 160.1332, Δ 0.11 ppm.

[α]$_D$^{25} = –0.8 (c = 1.00, MeOH).

(*R*)-4-Amino-5-phenylpentanoic acid hydrochloride, 9e

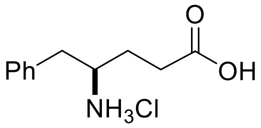

Phenylalanine-derived hydrogen borrowing product **3e** (104 mg, 0.2 mmol, 1.0 eq.) was subjected to **General Procedure B** to give the desired compound **9e** (43 mg, 94%) as a tan solid.

The spectral data matched that previously reported in the literature.[25]

m.p. = 75–77 °C.

IR (film) ν_{max}/cm^{-1} = 2916 (br.), 1715, 1690, 1485, 1373, 1231, 1217, 860, 741, 724, 691, 642.

^{1}H NMR (D_2O, 400 MHz) δ = 7.48 – 7.29 (m, 5H), 3.63 (dq, *J* = 8.0, 6.5 Hz, 1H), 3.09 (dd, *J* = 14.4, 6.3 Hz, 1H), 2.92 (dd, *J* = 14.3, 8.0 Hz, 1H), 2.57 (dd, *J* = 8.1, 7.0 Hz, 2H), 1.99 (dddd, *J* = 8.5, 7.6, 6.5, 2.0 Hz, 2H).

^{13}C NMR (D_2O, 101 MHz) δ = 176.8, 135.5, 129.4 (2C), 129.1 (2C), 127.6, 52.4, 37.9, 29.7, 27.0.

HRMS (ESI$^+$) Found [M+H]$^+$ = 194.1179; $C_{11}H_{16}O_2N$ requires 194.1176, Δ 1.77 ppm.

[α]$_D$^{25} = –18.1 (c = 1.00, MeOH).

3-((4*R*,5*R*)-3-Benzyl-5-methyloxazolidin-4-yl)propanoic acid hydrochloride, 9f

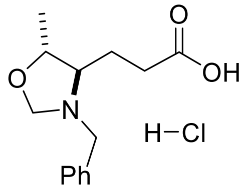

Threonine-derived hydrogen borrowing product **3h** (76 mg, 0.2 mmol, 1.0 eq.) was subjected to **General Procedure B** to give the desired compound **9f** (20 mg, 37%) as a colourless residue.

IR (film) ν_{max}/cm^{-1} = 3374, 2981 (br.), 1720, 1394, 750, 702.

^{1}H NMR (D_2O, 400 MHz) δ = 7.61 – 7.43 (m, 5H), 5.09 (d, *J* = 6.6 Hz, 1H), 4.85 (d, *J* = 6.6 Hz, 1H), 4.58 (d, *J* = 13.0 Hz, 1H), 4.41 (d, *J* = 13.0 Hz, 1H), 4.13 (dq, *J* = 7.5, 6.2 Hz, 1H), 3.41 (dt, *J* = 7.5, 6.9 Hz, 1H), 2.26 – 2.07 (m, 2H), 1.90 (qd, *J* = 6.9, 2.7 Hz, 2H), 1.48 (d, *J* = 6.2 Hz, 3H).

^{13}C NMR (D_2O, 101 MHz) δ = 176.1, 130.9 (2C), 130.5, 129.6 (2C), 129.0, 83.3, 81.3, 68.8, 58.6, 29.2, 24.0, 17.2.

HRMS (ESI^+) Found $[M+H]^+$ = 250.1437; $C_{14}H_{20}O_3N$ requires 250.1438, Δ –0.31 ppm.

$[\alpha]_D^{25}$ = –7.3 (c = 1.00, MeOH).

(*R*)-4-Amino-5-(4-methoxyphenyl)pentanoic acid hydrochloride, 9g

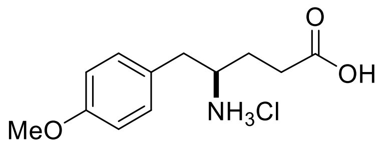

Tyrosine-derived hydrogen borrowing product **3j** (119 mg, 0.2 mmol, 1.0 eq.) was subjected to **General Procedure B** to give the desired compound **9g** (43 mg, 83%) as a thick, clear oil.

IR (film) ν_{max}/cm^{-1} = 2935 (br.), 1711, 1612, 1512, 1247, 908, 729.

^{1}H NMR (D_2O, 400 MHz) δ = 7.31 – 7.21 (m, 2H), 7.03 – 6.99 (m, 2H), 3.83 (s, 3H), 3.58 (dd, *J* = 7.8, 6.3 Hz, 1H), 3.02 (dd, *J* = 14.3, 6.3 Hz, 1H), 2.86 (dd, *J* = 14.3, 7.8 Hz, 1H), 2.55 (dd, *J* = 8.3, 6.7 Hz, 2H), 1.97 (dddd, *J* = 8.3, 7.8, 6.7, 2.1 Hz, 2H).

^{13}C NMR (D_2O, 101 MHz) δ = 176.8, 158.2, 130.7 (2C), 128.0, 114.6 (2C), 55.4, 52.5, 37.1, 29.7, 26.9.

HRMS (ESI$^+$) Found $[M+H]^+$ = 224.1283; $C_{12}H_{18}O_3N$ requires 224.1281, Δ 0.99 ppm.

$[\alpha]_D^{25}$ = –0.4 (c = 1.00, MeOH).

(*S*)-4,8-Bis(dibenzylamino)octanoic acid dihydrochloride, 9h

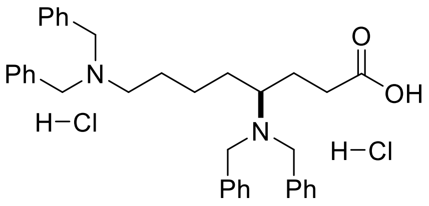

Lysine-derived hydrogen borrowing product **3k** (134 mg, 0.2 mmol, 1.0 eq.) was subjected to **General Procedure B** to give the desired compound **9h** (104 mg, 86%) as an opaque gum.

IR (film) ν_{max}/cm^{-1} = 3398, 2981 (br.), 1458, 1024, 1002, 756, 702.

^{1}H NMR (D_2O, 400 MHz) δ = 7.56 – 7.44 (m, 18H), 7.29 (d, *J* = 7.3 Hz, 2H), 4.60 (d, *J* = 13.3 Hz, 1H), 4.47 – 4.27 (m, 6H), 4.21 (d, *J* = 13.3 Hz, 1H), 3.09 (dt, *J* = 10.6, 6.0 Hz, 3H), 2.62 – 2.50 (m, 1H), 2.35 – 2.20 (m, 2H), 1.91 (td, *J* = 11.2, 6.8 Hz, 2H), 1.81 – 1.59 (m, 3H), 1.38 – 1.26 (m, 1H), 1.20 (q, *J* = 8.5 Hz, 1H).

^{13}C NMR (D_2O, 101 MHz) δ = 177.9, 131.0, 130.8, 130.6, 130.19, 130.16, 129.8, 129.7, 129.4, 129.3, 129.0, 62.1, 57.3, 54.8, 53.9, 51.3, 31.6, 27.3, 23.6, 22.9, 22.7.

HRMS (ESI$^+$) Found $[M+H]^+$ = 535.3320; $C_{36}H_{43}O_2N_2$ requires 535.3319, Δ 0.24 ppm.

$[\alpha]_D^{25}$ = +4.1 (c = 1.00, MeOH).

(*R*)-4-Amino-4-phenylbutanoic acid hydrochloride, 9i

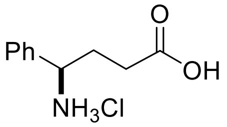

Phenylglycine-derived hydrogen borrowing product **3g** (110 mg, 0.2 mmol, 1.0 eq.) was subjected to **General Procedure B** to give the desired compound **9i** (42 mg, 98%) as a colourless powder.

m.p. = 195–197 °C.

IR (film) ν_{max}/cm^{-1} = 2904 (br.), 1719, 1498, 1413, 1217, 1177, 1126, 821, 765, 697.

^{1}H NMR (D_2O, 400 MHz) δ = 7.60 – 7.43 (m, 5H), 4.44 (td, *J* = 5.9, 3.4 Hz, 1H), 2.47 – 2.23 (m, 4H).

^{13}C NMR (D_2O, 101 MHz) δ = 176.6, 135.2, 129.7, 129.4 (2C), 127.4 (2C), 54.7, 29.9, 28.4.

HRMS (ESI$^+$) Found $[M+H]^+$ = 180.1020; $C_{10}H_{14}O_2N$ requires 180.1019, Δ 0.73 ppm.

$[\alpha]_D^{25}$ = –17.5 (c = 1.00, MeOH).

4-Amino-4-methylpentanoic acid hydrochloride, 9j

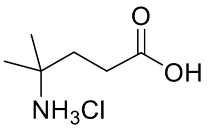

Dimethyl hydrogen borrowing product **3m** (80 mg, 0.16 mmol, 1.0 eq.) was subjected to **General Procedure B** to give the desired compound **9j** (26 mg, 97%) as a colourless powder.

m.p. = 170–172 °C.

IR (film) ν_{max}/cm^{-1} = 2932 (br.), 1706, 1414, 1189, 843, 648.

^{1}H NMR (D_2O, 400 MHz) δ = 2.51 (t, *J* = 8.1 Hz, 2H), 1.97 (t, *J* = 8.1 Hz, 2H), 1.35 (s, 6H).

^{13}C NMR (D_2O, 101 MHz) δ = 177.3, 53.9, 34.3, 28.5, 24.1 (2C).

HRMS (ESI$^+$) Found $[M+H]^+$ = 132.1019; $C_6H_{14}O_2N$ requires 132.1019, Δ –0.28 ppm.

3-(1-Aminocyclopentyl)propanoic acid hydrochloride, 9k

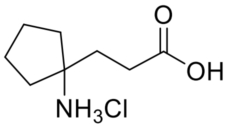

Cycloleucine-derived hydrogen borrowing product **3n** (106 mg, 0.2 mmol, 1.0 eq.) was subjected to **General Procedure B** to give the desired compound **9k** (28 mg, 90%) as a colourless powder.

m.p. = 184–186 °C.

IR (film) ν_{max}/cm^{-1} = 2879 (br.), 1742, 1704, 1580, 1522, 1410, 1214, 1191, 886.

^{1}H NMR (D_2O, 400 MHz) δ = 2.56 (t, *J* = 7.9 Hz, 2H), 2.08 (t, *J* = 7.9 Hz, 2H), 1.95 – 1.73 (m, 8H).

^{13}C NMR (D_2O, 101 MHz) δ = 177.3, 64.1, 35.8 (2C), 32.4, 28.9, 23.7 (2C).

HRMS (ESI^+) Found $[M+H]^+$ = 158.1175; $C_8H_{16}O_2N$ requires 158.1176, Δ –0.17 ppm.

4.5 Experiments from Scheme 4

Benzyl (*R*)-4-(dibenzylamino)-4-phenylbutanoate, 10

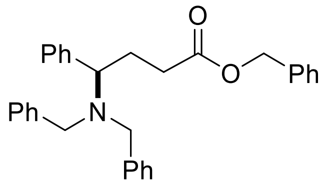

To a stirred suspension of phenylglycine derived cleavage product **9i** (11 mg, 0.05 mmol, 1.0 eq.) and K_2CO_3 (28 mg, 0.2 mmol, 4.0 eq.) in MeCN (1 mL) at RT was added benzyl bromide (20 µL, 0.175 mmol, 3.5 eq.). The resulting solution was then warmed to 80 °C and left to stir for 16 h. The reaction mixture was then cooled to RT and water (3 mL) was added. The resulting mixture was then extracted with EtOAc (3 × 4 mL), and the organic extracts washed with brine (10 mL), dried over Na_2SO_4, and concentrated *in vacuo*. Purification by column chromatography (SiO_2, eluent load, pentane:Et_2O, 95:5) gave the desired compound **10** (18 mg, 80%, 93:7 e.r.) as a colourless oil.

IR (film) v_{max}/cm^{-1} = 2980, 1734, 1494, 1454, 1158, 746, 698.

^{1}H NMR ($CDCl_3$, 400 MHz) δ = 7.34 (m, 16H), 7.25 – 7.18 (m, 4H), 5.04 (dd, *J* = 19.0, 12.2 Hz, 2H), 3.85 (d, *J* = 13.8 Hz, 2H), 3.70 (t, *J* = 7.0 Hz, 1H), 3.12 (d, *J* = 13.8 Hz, 2H), 2.63 – 2.52 (m, 1H), 2.51 – 2.32 (m, 2H), 2.16 – 2.03 (m, 1H).

^{13}C NMR ($CDCl_3$, 101 MHz) δ = 173.6, 140.1 (2C), 138.0, 136.2, 129.1 (2C), 128.9 (4C), 128.7 (2C), 128.44 (4C), 128.36 (2C), 128.3, 128.2 (2C), 127.5, 127.0 (2C), 66.3, 61.4, 53.7 (2C), 32.0, 26.7.

HRMS (ESI^+) Found $[M+H]^+$ = 450.2425; $C_{31}H_{32}O_2N$ requires 450.2428, Δ –0.52 ppm.

$[\alpha]_D^{25}$ = +37.2 (c = 1.00, $CHCl_3$).

Chiral HPLC (Chiralpak IA with guard, 1.0 % IPA, 99.0 % hexane, 1.0 mL/min, 25 °C, λ = 254 nm, 10 µL injection).

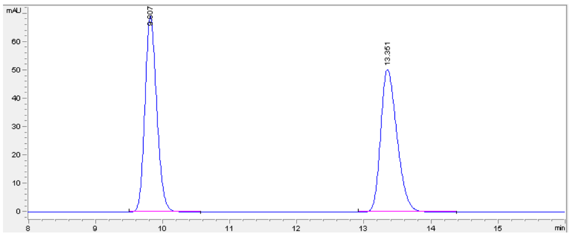

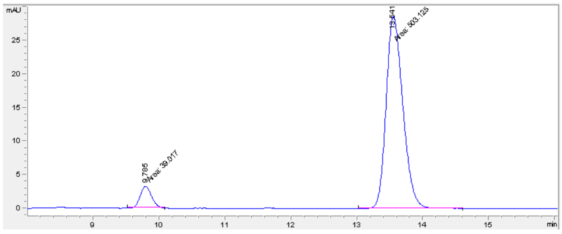

#	Time	Type	Area	Height	Width	Area%	Symmetry
1	9.807	BB	844.2	69	0.1888	49.697	0.799
2	13.351	BB	854.5	50.3	0.2622	50.303	0.679

#	Time	Type	Area	Height	Width	Area%	Symmetry
1	9.785	MM	39	3.2	0.2004	7.197	0.88
2	13.541	MM	503.1	28.6	0.2932	92.803	0.745

Chromatography-free two-step reaction to form 9c

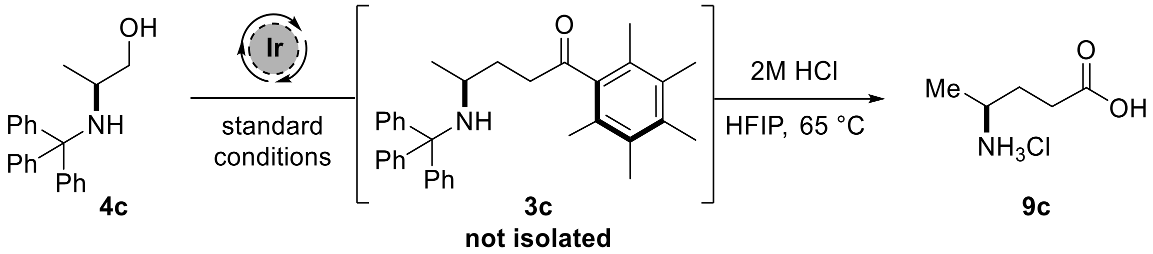

A 10–20 mL Biotage® microwave vial equipped with a stirrer bar, was sequentially charged with the trityl protected alaninol **4c** (1.27 g, 4.0 mmol, 1.0 eq.), pentamethylacetophenone **2** (1.14 g, 6.0 mmol, 1.5 eq.), [Cp*IrCl$_2$]$_2$ (64 mg, 0.08 mmol, 2 mol%), NaO^{t}Bu (192 mg, 2.0 mmol, 0.5 eq.), and *tert*-butanol (1.6 mL). The reaction vessel was sealed with a microwave vial cap (containing a Reseal™ septum), pierced with a needle attached to a nitrogen balloon, and the vial was heated to 85 in a preheated oil bath for 16 h. The mixture was cooled to RT and filtered through a SiO_2 plug, eluting with Et_2O (100 mL). The resulting solution was then washed with water (2 × 50 mL) and brine (50 mL), dried over Na_2SO_4, and concentrated *in vacuo* to give a crude black tar that was used for the next step without further purification. An aliquot was removed, purified by small-scale preparative TLC (pentane:Et_2O, 95:5), and subjected to HPLC analysis to determine the e.r. of intermediate **3c** to be 95:5 (Chiralpak IA with guard, 5.0 % IPA, 95.0 % hexane, 1.0 mL/min, 25 °C, λ = 254 nm, 10 µL injection).

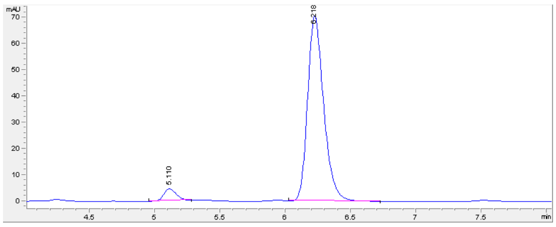

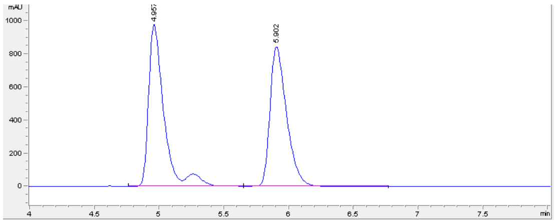

#	Time	Type	Area	Height	Width	Area%	Symmetry
1	5.11	BB	30.8	4.6	0.1018	4.858	0.787
2	6.218	BB	603.3	70.9	0.1293	95.142	0.739

#	Time	Type	Area	Height	Width	Area%	Symmetry
1	4.957	BV R	8113.7	979.2	0.1134	51.864	0.566
2	5.902	BV R	7530.6	842.7	0.1361	48.136	0.592

In a 10–20 mL Biotage® microwave vial equipped with a stirrer bar, the crude hydrogen borrowing product was dissolved in HFIP (35.2 mL) and conc. HCl (5.2 mL) was added. The reaction vessel was sealed with a microwave vial cap (containing a Reseal™ septum) and heated at 65 °C for 48 h. The reaction was then cooled to RT and diluted by the addition of water (30 mL). The mixture was then washed with Et_2O (3 × 50 mL) and concentrated *in vacuo* to give a tan solid. ^{1}H analysis found the product to be contaminated with detritylated, but Ph* containing, **3c** in an 11:1 ratio. The crude product was therefore recrystallised from boiling EtOH:Et_2O to give the pure desired product **9c** (571 mg, 92%) as fluffy white crystals.

To establish the e.r. of the cleavage product **9c**, it was perbenzylated and analysed by chiral HPLC in a similar manner to the procedure used for cleavage product **9i** to give compound **S25**.

Benzyl (*S*)-4-(dibenzylamino)pentanoate, S25

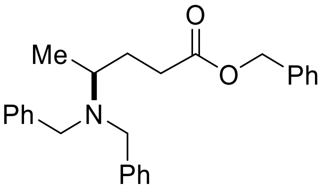

To a stirred suspension of alanine derived cleavage product **9c** (153 mg, 1.0 mmol, 1.0 eq.) and K_2CO_3 (552 mg, 4.0 mmol, 4.0 eq.) in MeCN (10 mL) at RT was added benzyl bromide (416 µL, 3.5 mmol, 3.5 eq.). The resulting solution was then warmed to 80 °C and left to stir for 16 h. The reaction mixture was then cooled to RT and water (10 mL) was added. The resulting mixture was then extracted with

EtOAc (3 × 15 mL), and the organic extracts washed with brine (30 mL), dried over Na_2SO_4, and concentrated *in vacuo*. Purification by column chromatography (SiO_2, eluent load, pentane:Et_2O, 95:5) gave the desired compound **S25** (329 mg, 85%, 98:2 e.r.) as a colourless oil.

IR (film) ν_{max}/cm^{-1} = 1733, 1494, 1454, 1166, 743, 696.

^{1}H NMR ($CDCl_3$, 400 MHz) δ = 7.47 – 7.11 (m, 15H), 5.13 – 4.98 (m, 2H), 3.75 (dd, *J* = 13.8, 3.4 Hz, 2H), 3.38 (dt, *J* = 13.8, 2.4 Hz, 2H), 2.75 (dddd, *J* = 11.6, 9.5, 6.4, 3.9 Hz, 1H), 2.66 – 2.52 (m, 1H), 2.42 – 2.28 (m, 1H), 1.98 – 1.83 (m, 1H), 1.72 – 1.54 (m, 1H), 1.09 – 1.01 (m, 3H).

^{13}C NMR ($CDCl_3$, 101 MHz) δ = 173.9, 140.4 (2C), 136.3, 128.9 (4C), 128.7 (2C), 128.33 (5C), 128.28 (2C), 126.9 (2C), 66.2, 53.3 (2C), 52.1, 31.8, 29.1, 13.0.

HRMS (ESI$^+$) Found $[M+H]^+$ = 388.2278; $C_{26}H_{30}O_2N$ requires 388.2271, Δ 1.90 ppm.

$[\alpha]_D^{25}$ = –9.9 (c = 1.00, $CHCl_3$).

Chiral HPLC (Chiralpak IA with guard, 1.0 % IPA, 99.0 % hexane, 1.0 mL/min, 25 °C, λ = 254 nm, 10 µL injection).

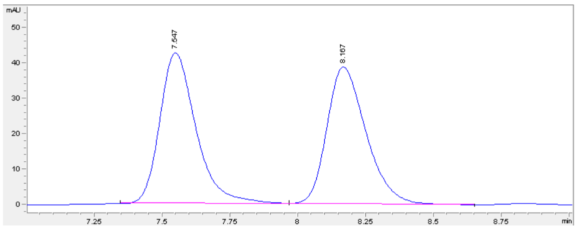

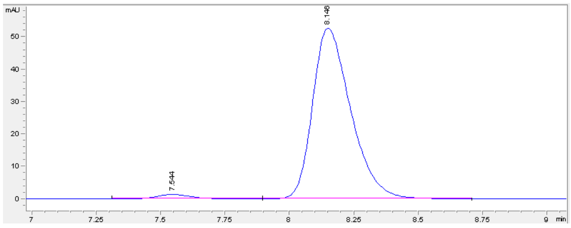

#	Time	Type	Area	Height	Width	Area%	Symmetry
1	7.547	BV	399.2	42.7	0.1431	50.502	0.697
2	8.167	VB	391.3	38.8	0.1537	49.498	0.689

#	Time	Type	Area	Height	Width	Area%	Symmetry
1	7.544	BB	12.3	1.3	0.1408	2.207	0.818
2	8.146	BB	543.8	52.9	0.158	97.793	0.642

5. *References*

[1] A. B. Pangborn, M. A. Gairdello, R. H. Grubbs, R. K. Rosen, F. J. Timmers, *Organometallics*, **1996**, *15*, 1518–1520.

[2] *Purification of Laboratory Chemicals*, 3rd edition. D.D. Perrin, W. L. F. Armarego, *Pergamon Press*, Oxford, **1988**.

[3] P. Kiprof, J. Li, C. L. Renish, E. K. Kalombo, V. G. Young, *J. Organomet. Chem.* **2001**, *620*, 113–118.

[4] J. J. Klein, S. Hecht, *Org. Lett.* **2012**, *14*, 330–333.

[5] H.-S. Chong, Y. Chen, *Org. Lett.* **2013**, *15*, 5912–5915.

[6] G. M. Almiento, D. Balducci, A. Bottoni, M. Calvaresi, G. Porzi, *Tetrahedron: Asymmetry* **2007**, *18*, 2695–2711.

[7] A. Albeck, R. Persky, *J. Org. Chem.* **1994**, *59*, 653–657.

[8] L. Zervas, D. M. Theodoropoulos, *J. Am. Chem. Soc.* **1956**, *78*, 1359–1363.

[9] M. B. Widegren, M. L. Clarke, *Org. Lett.* **2018**, *20*, 2654–2658.

[10] E. K. Dolence, C. E. Lin, M. J. Miller, S. M. Payne, *J. Med. Chem.* **1991**, *34*, 956–968.

[11] P. G. Andersson, D. Guijarro, D. Tanner, *J. Org. Chem.* **1997**, *62*, 7364–7375.

[12] L. E. Overman, J. Shim, *J. Org. Chem.* **1993**, *58*, 4662–4672.

[13] J. M. Chalker, A. Yang, K. Deng, T. Cohen, *Org. Lett.* **2007**, *9*, 3825–3828.

[14] H. Tohma, Y. Harayama, M. Hashizume, M. Iwata, Y. Kiyono, M. Egi, Y. Kita, *J. Am. Chem. Soc.* **2003**, *125*, 11235–11240.

[15] G. Deniau, T. Moraux, D. O'Hagan, A. M. Z. Slawin, *Tetrahedron: Asymmetry* **2008**, *19*, 2330–2333.

[16] M. U. Luescher, J. W. Bode, *Synlett* **2019**, *30*, 464–470.

[17] K. Y. Zee-Cheng, C. C. Cheng, *J. Med. Chem.* **1972**, *15*, 13–16.

[18] W.-C. Chen, S.-Y. Chou, M.-C. Hsu, C.-H. R. King, B. Shi, J. Yuan, *Antibiotic Drug*, **2010**, WO2010009014A2.

[19] A. H. Dardir, N. Hazari, S. J. Miller, C. R. Shugrue, *Org. Lett.* **2019**, *21*, 5762–5766.

[20] A. Noordam, L. Maat, H. C. Beyerman, *Recueil des Travaux Chimiques des Pays-Bas* **1978**, *97*, 293–295.

[21] C. J. Fahrni, A. Pfaltz, *Helvetica Chimica Acta* **1998**, *81*, 491–506.

[22] T. Lehmann, D. Michel, M. Glänzel, R. Waibel, P. Gmeiner, *Heterocycles* **1999**, 51, 1389–1400.

[23] D. Mirk, H. Luftmann, S. R. Waldvogel, *Zeitschrift für Naturforschung B* **2005**, *60*, 1077–1082.

[24] S. J. Kulkarni, Y. Pedduri, A. G. Chittiboyina, M. A. Avery, *J. Org. Chem.* **2010**, *75*, 3113–3116.

[25] K. Laqua, M. Klemm, M. Richard-Greenblatt, A. Richter, L. Liebe, T. Huang, S. Lin, A. Guardia, E. Pérez-Herran, L. Ballell, Y. Av-Gay, P. Imming, *Bioorganic & Medicinal Chemistry* **2018**, *26*, 3166–3190.

6. NMR Spectra

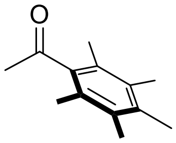

2

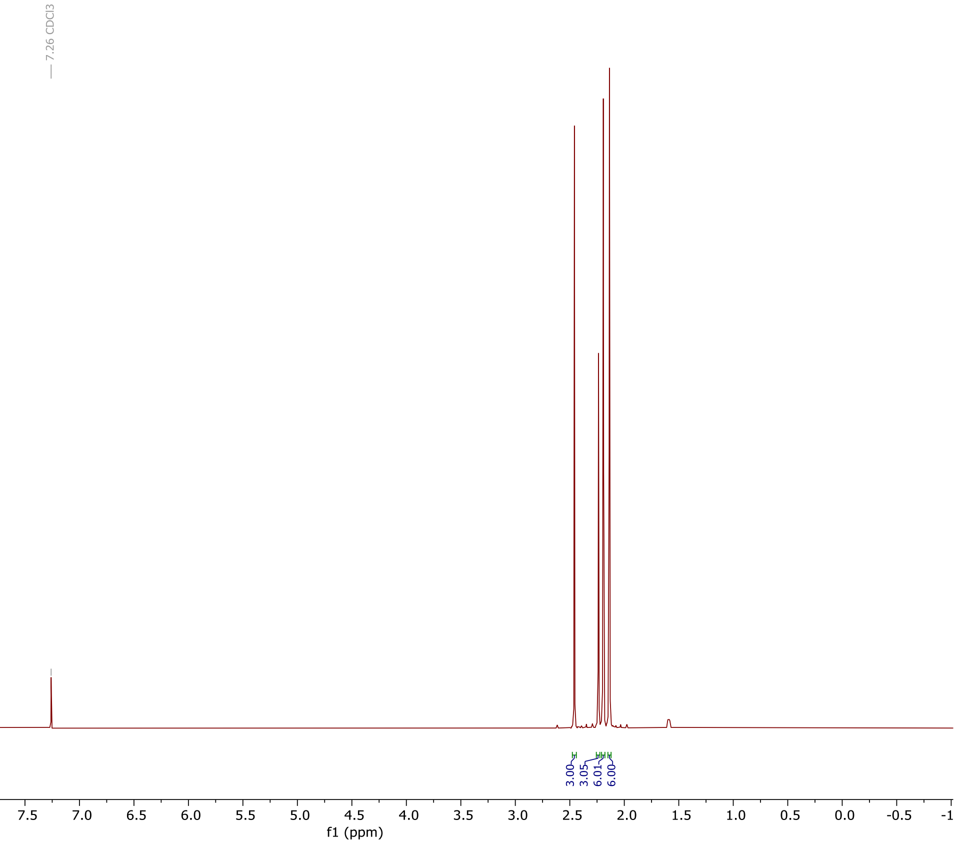

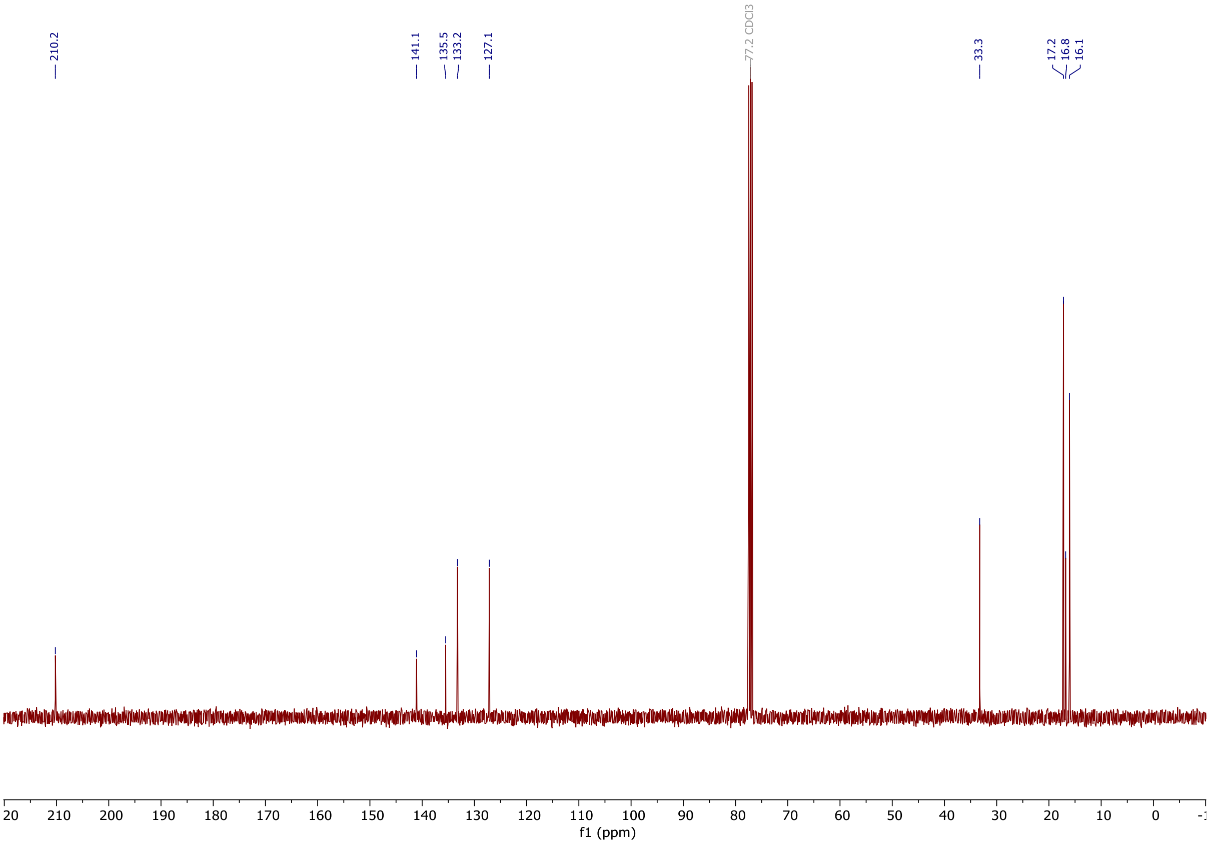

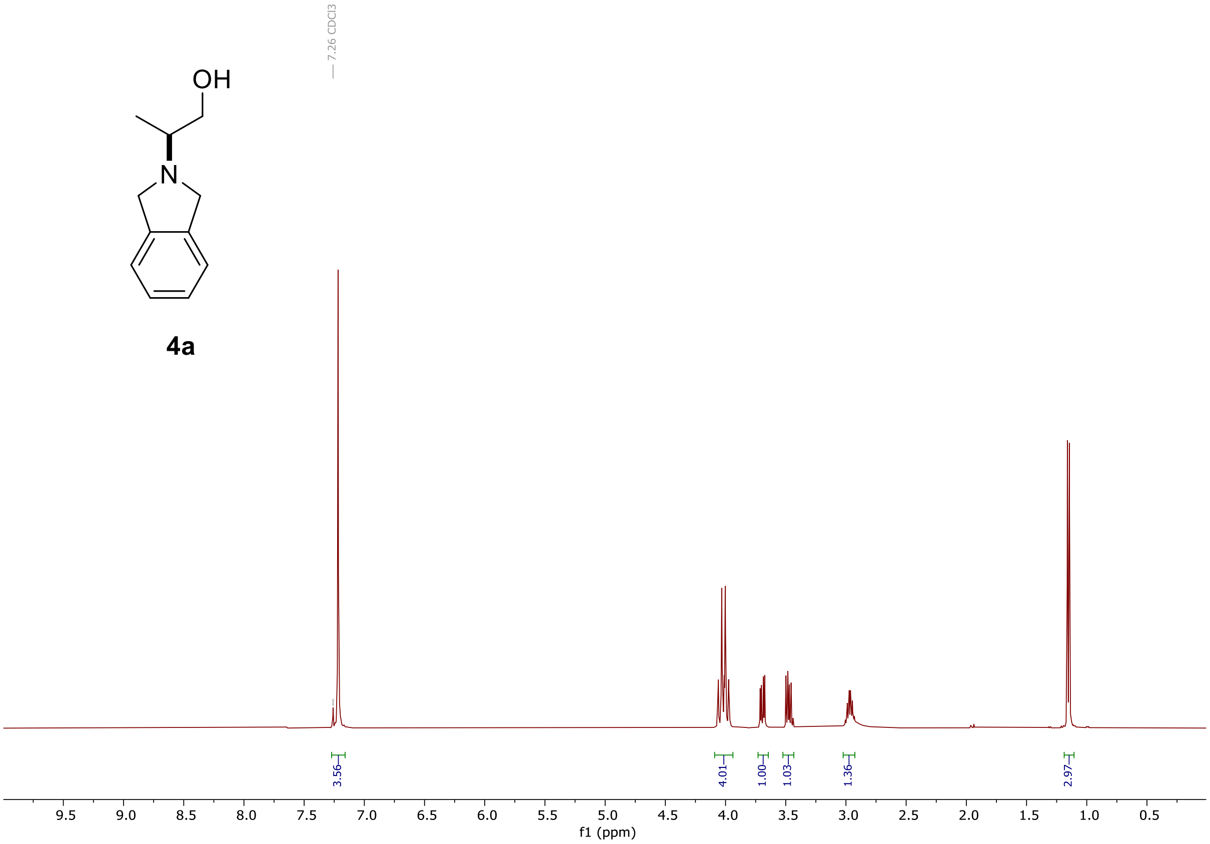
OH
N
7.26 CDCl3
3.56
4.01
1.00
1.03
1.36
2.97
f1 (ppm)

4a

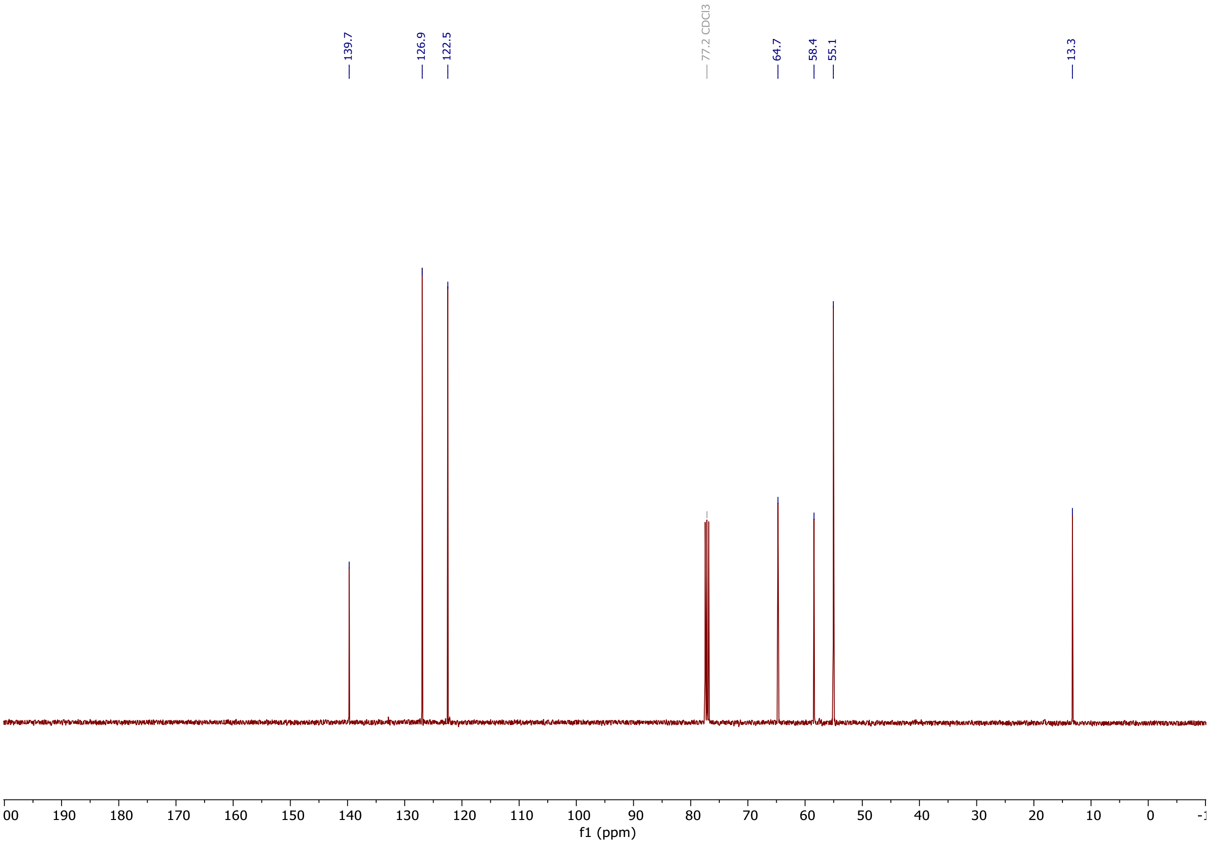
139.7
126.9
122.5
77.2 CDCl3
64.7
58.4
55.1
13.3
f1 (ppm)

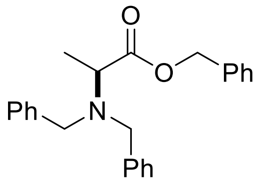

S1

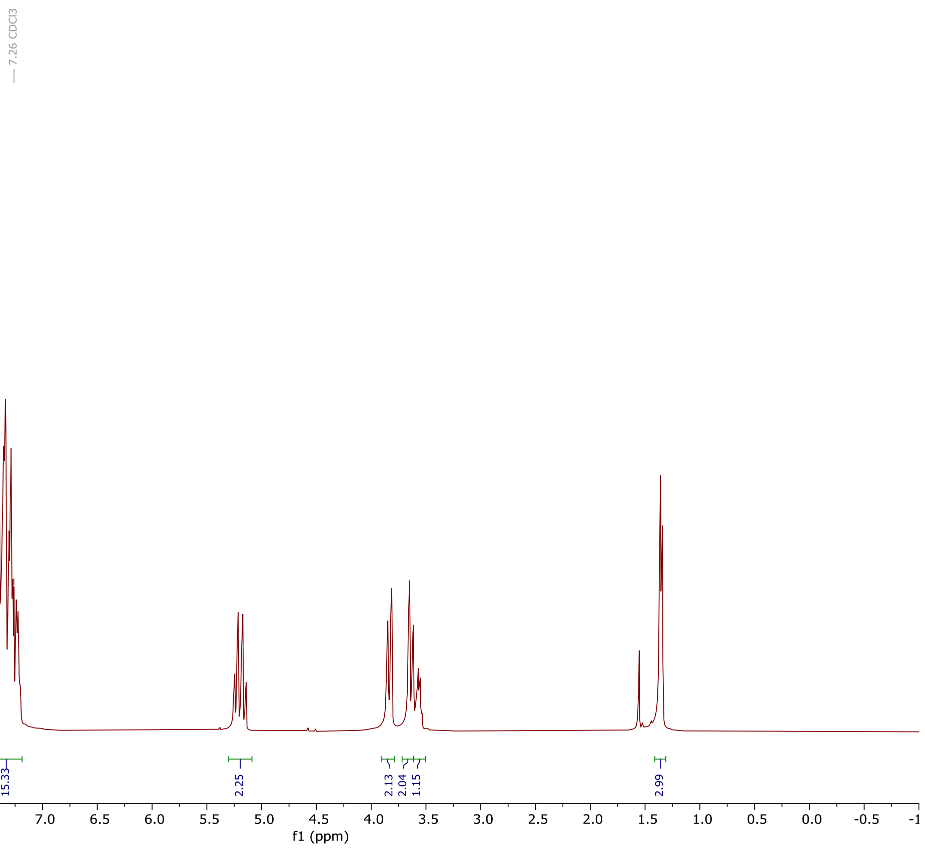
7.26 CDCl3
15.33
2.25
2.13
2.04
1.15
2.99
9.5 9.0 8.5 8.0 7.5 7.0 6.5 6.0 5.5 5.0 4.5 4.0 3.5 3.0 2.5 2.0 1.5 1.0 0.5 0.0 -0.5
f1 (ppm)

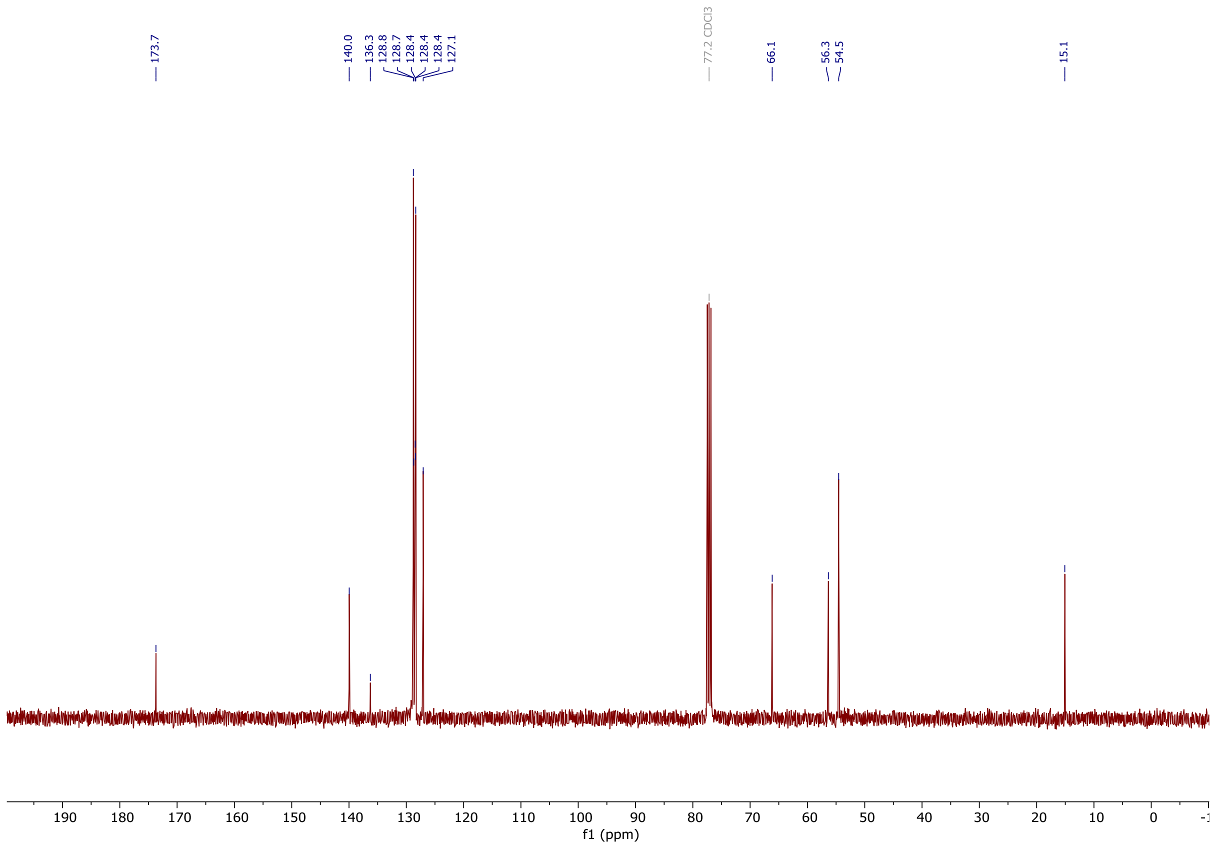
173.7
140.0
136.3
128.8
128.7
128.4
128.4
128.4
127.1
77.2 CDCl3
66.1
56.3
54.5
15.1
190 180 170 160 150 140 130 120 110 100 90 80 70 60 50 40 30 20 10 0
f1 (ppm)

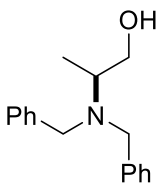
OH
Ph
N
Ph

4b

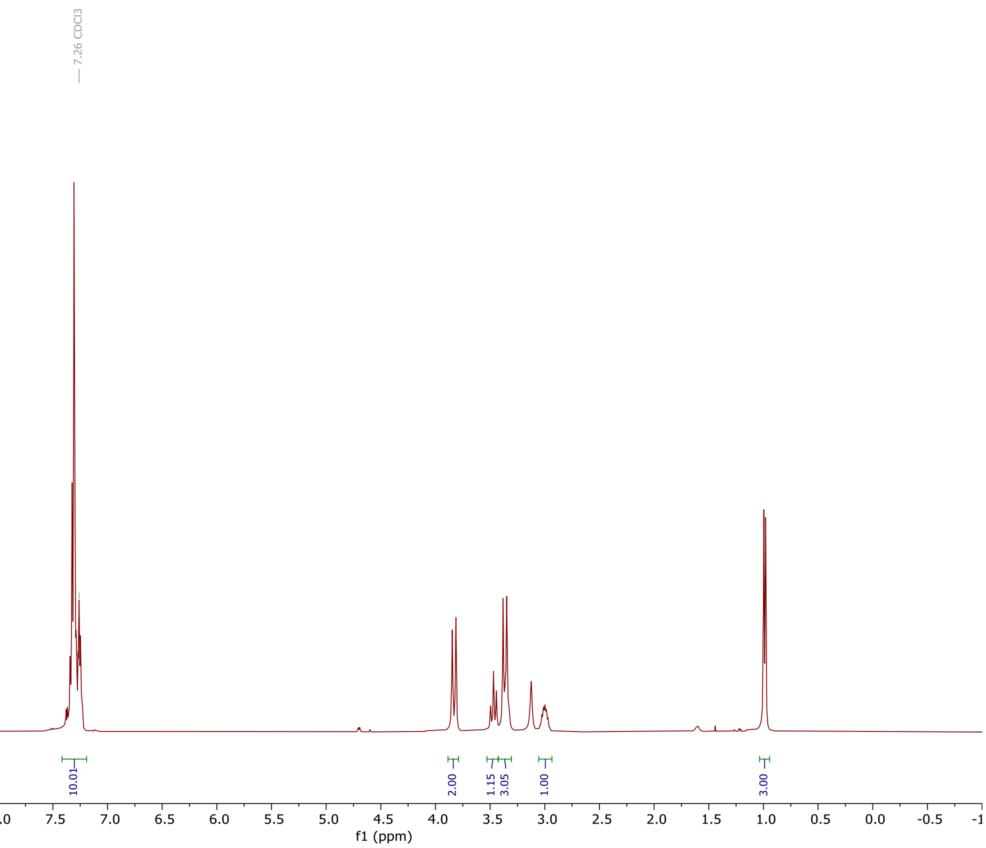
7.26 CDCl3
10.01
2.00
1.15
3.05
1.00
3.00
9.5
9.0
8.5
8.0
7.5
7.0
6.5
6.0
5.5
5.0
4.5
4.0
3.5
3.0
2.5
2.0
1.5
1.0
0.5
0.0
-0.5
f1 (ppm)

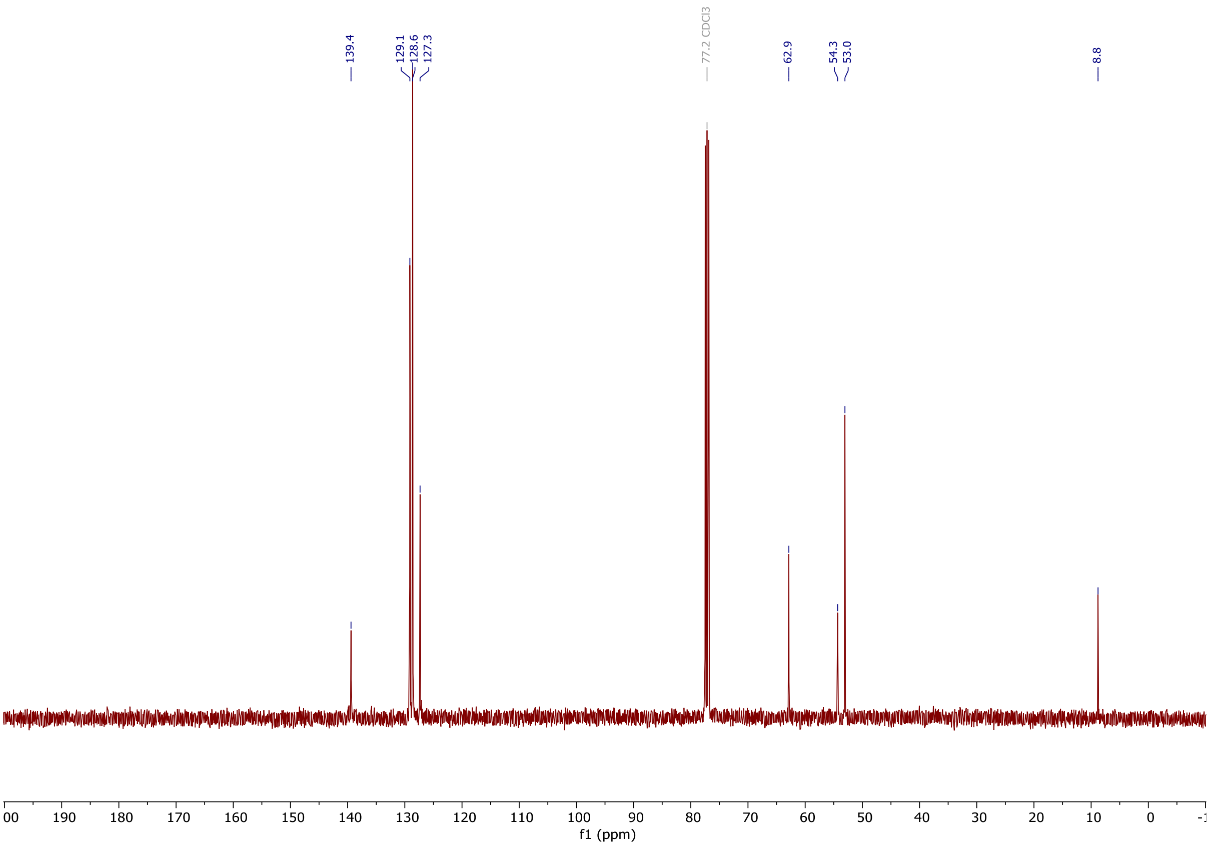
139.4
129.1
128.6
127.3
77.2 CDCl3
62.9
54.3
53.0
8.8
190
180
170
160
150
140
130
120
110
100
90
80
70
60
50
40
30
20
10
0
f1 (ppm)

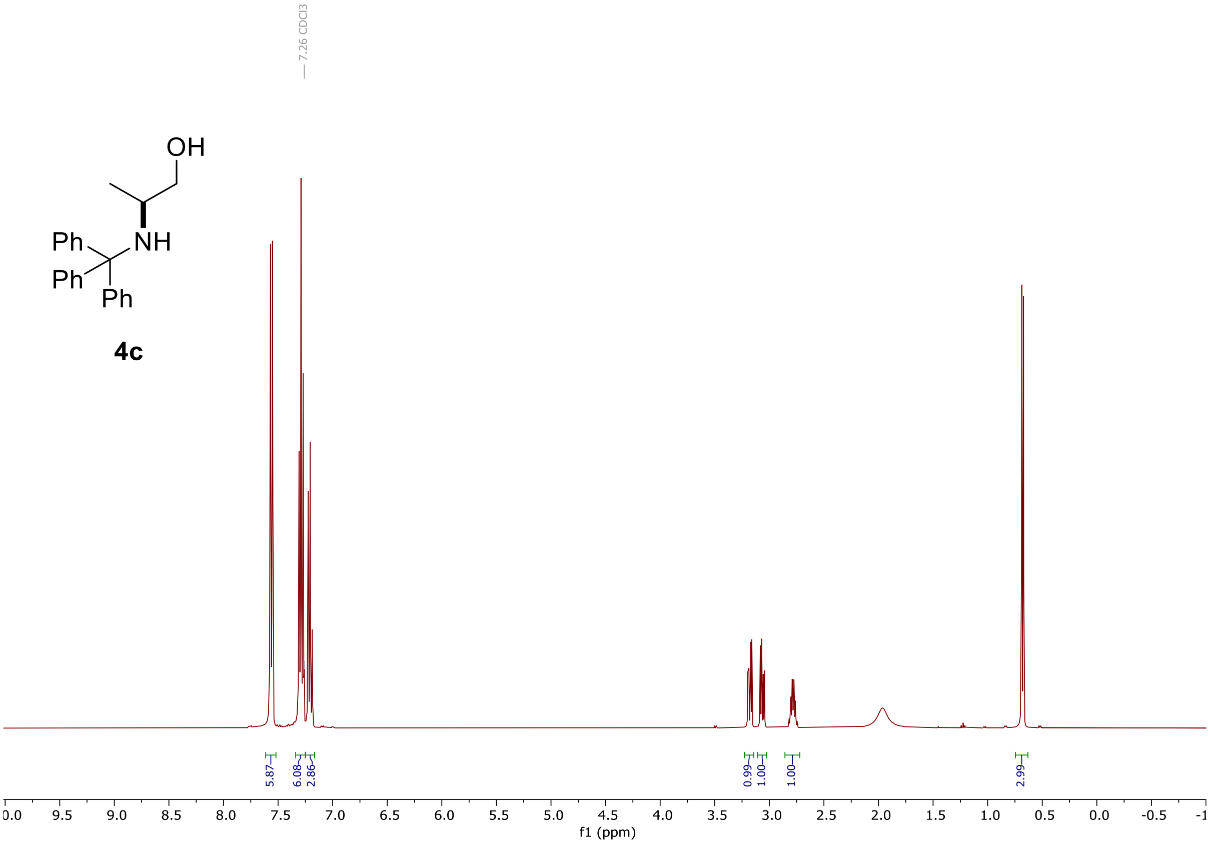
7.26 CDCl3
OH
NH
Ph
Ph
Ph
4c
5.87
6.08
2.86
0.99
1.00
1.00
2.99
f1 (ppm)

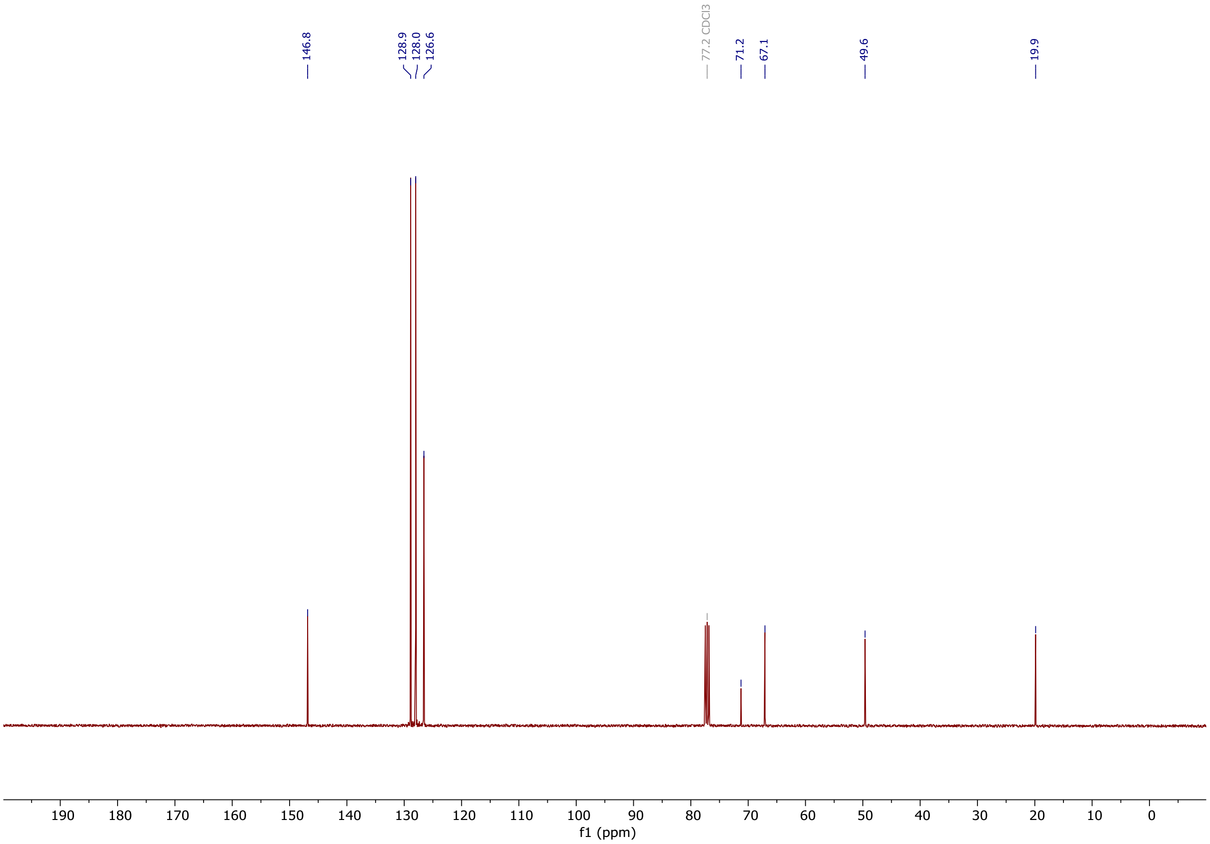
146.8
128.9
128.0
126.6
77.2 CDCl3
71.2
67.1
49.6
19.9
f1 (ppm)

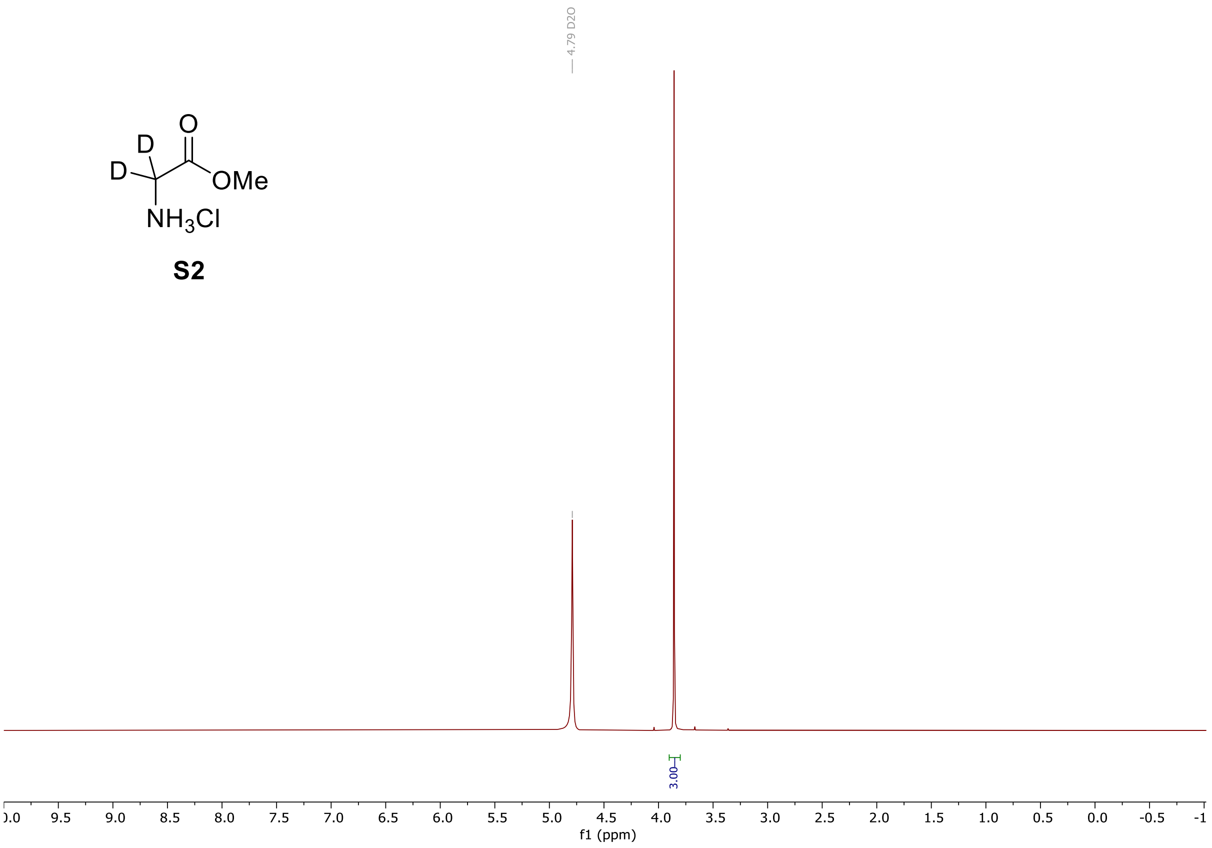
D
D
O
OMe
NH3Cl
S2
4.79 D2O
3.00
f1 (ppm)

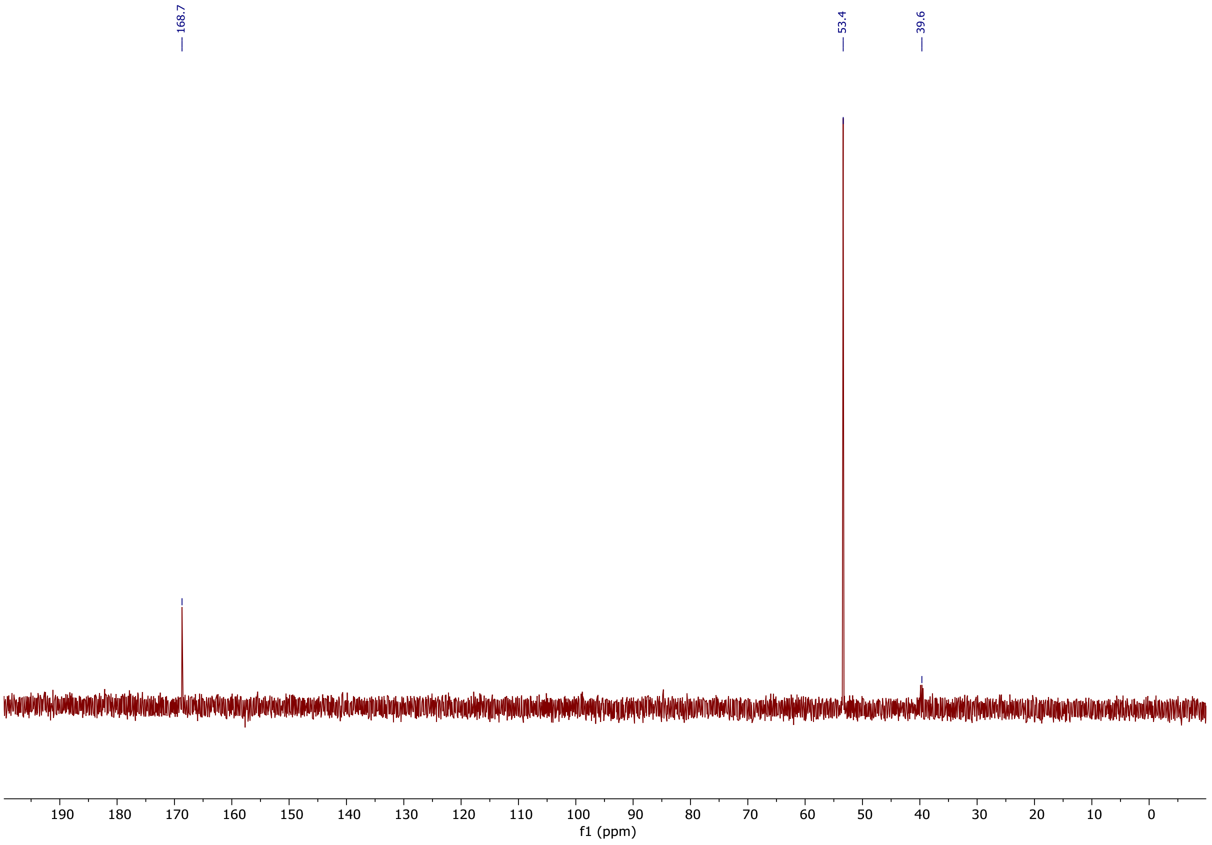
168.7
53.4
39.6
f1 (ppm)

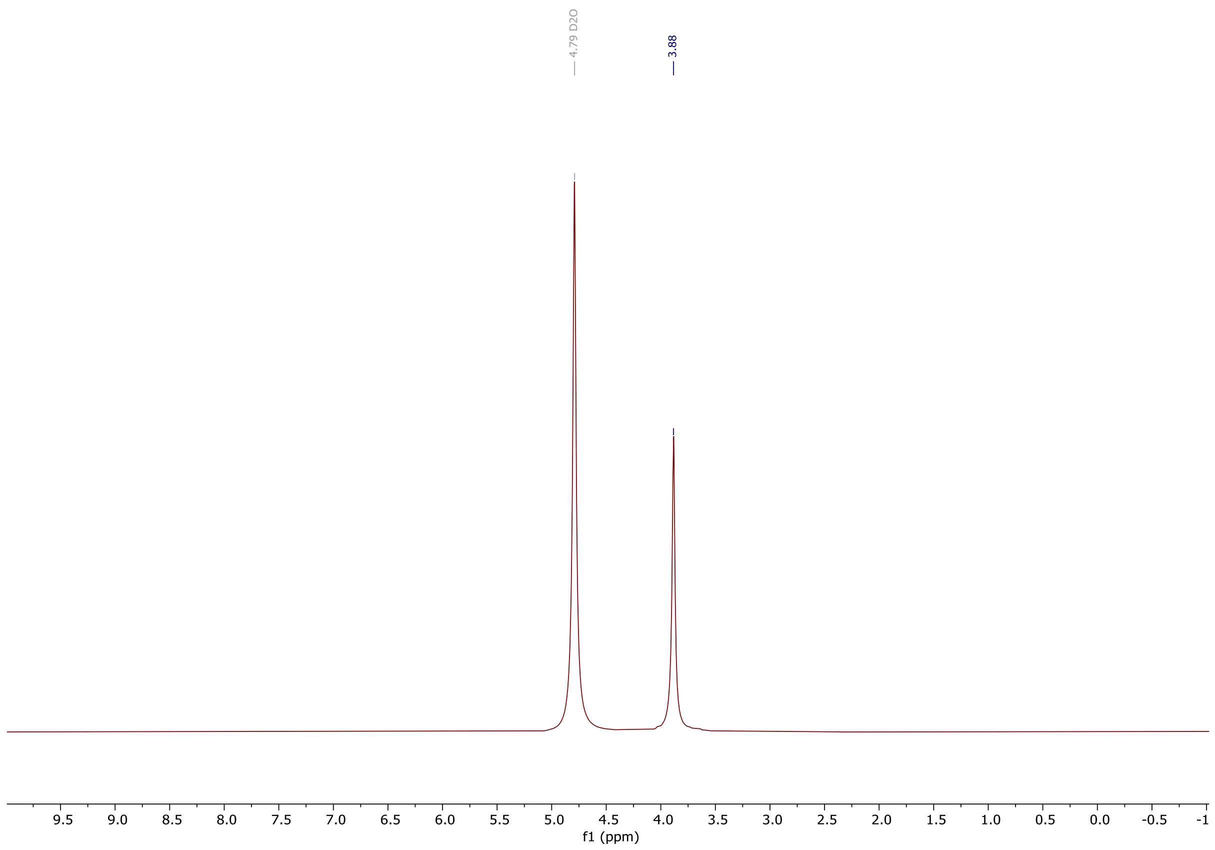

4.79 D2O
3.88
9.5 9.0 8.5 8.0 7.5 7.0 6.5 6.0 5.5 5.0 4.5 4.0 3.5 3.0 2.5 2.0 1.5 1.0 0.5 0.0 -0.5 -1
f1 (ppm)

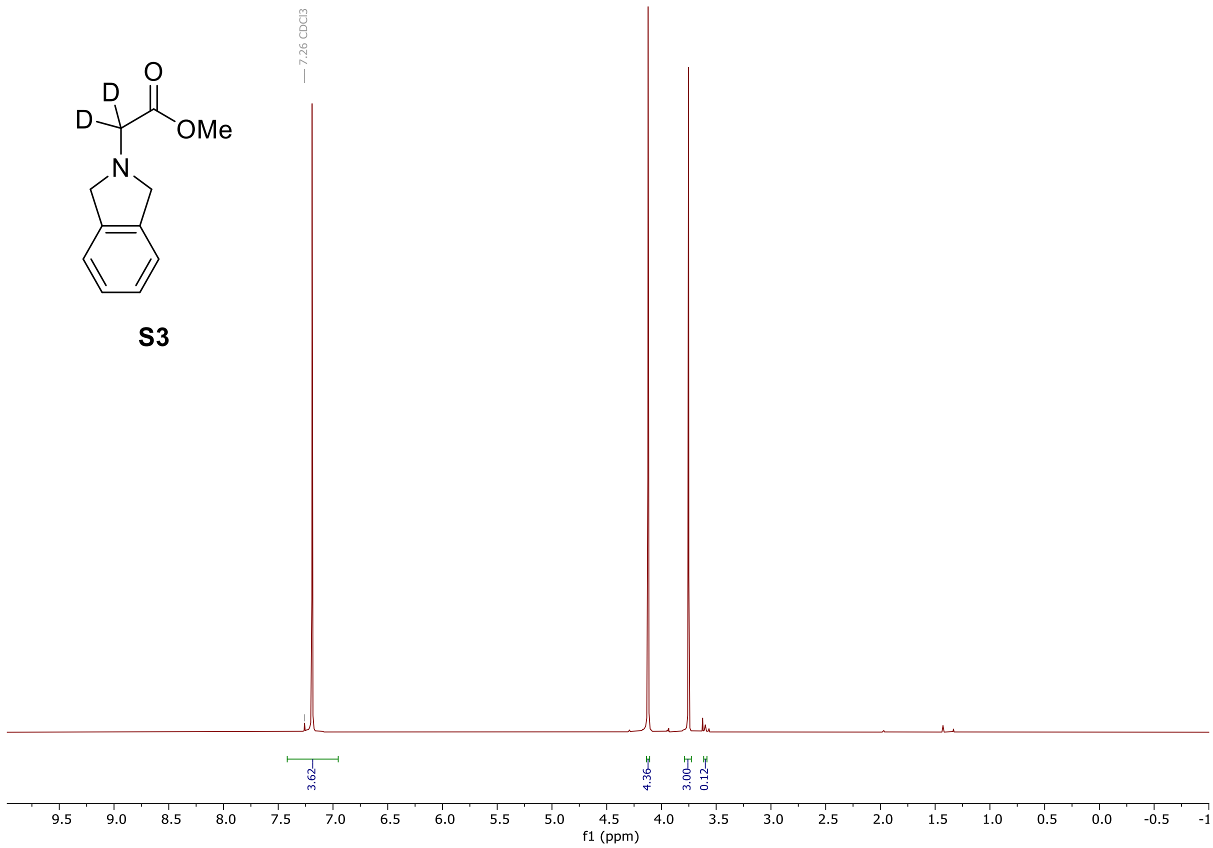

D
D
O
OMe
N
S3
7.26 CDCl3
3.62
4.36
3.00
0.12
9.5 9.0 8.5 8.0 7.5 7.0 6.5 6.0 5.5 5.0 4.5 4.0 3.5 3.0 2.5 2.0 1.5 1.0 0.5 0.0 -0.5
f1 (ppm)

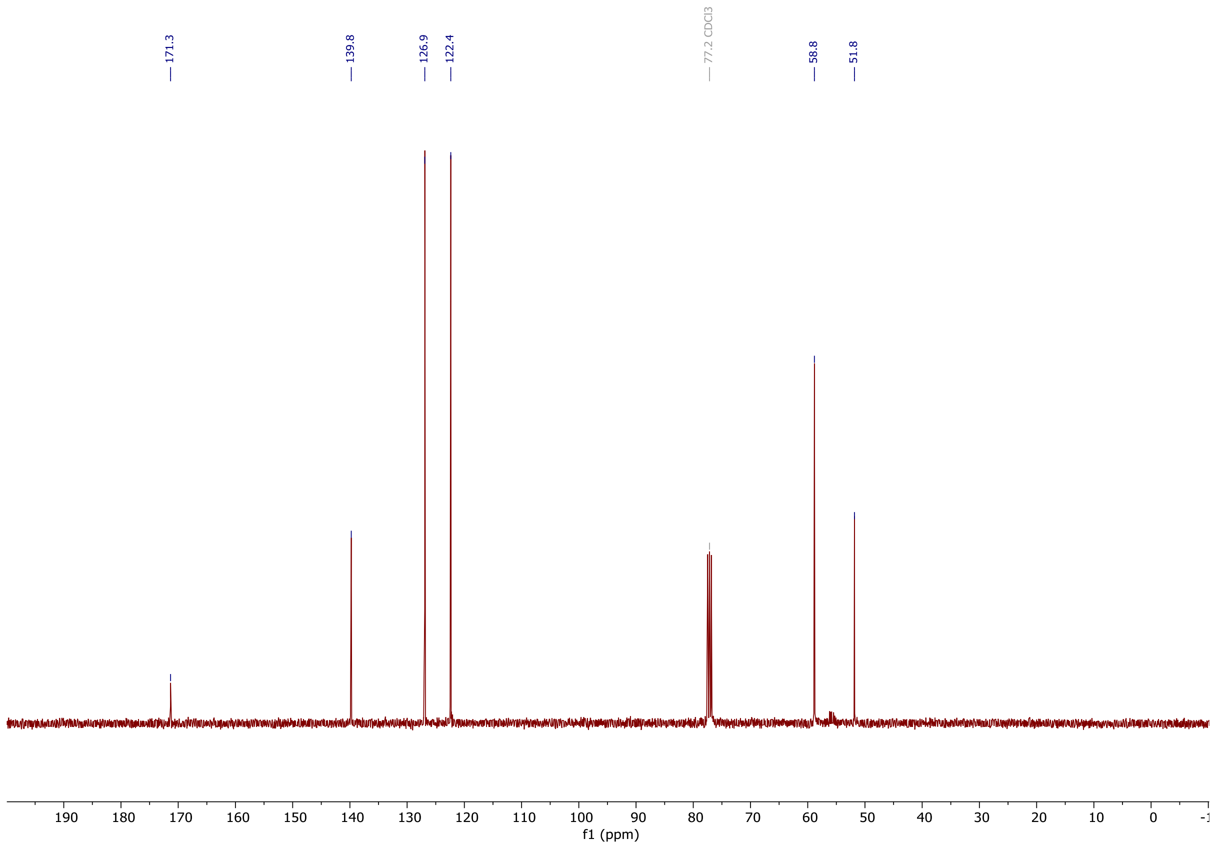

171.3
139.8
126.9
122.4
77.2 CDCl3
58.8
51.8
190 180 170 160 150 140 130 120 110 100 90 80 70 60 50 40 30 20 10 0
f1 (ppm)

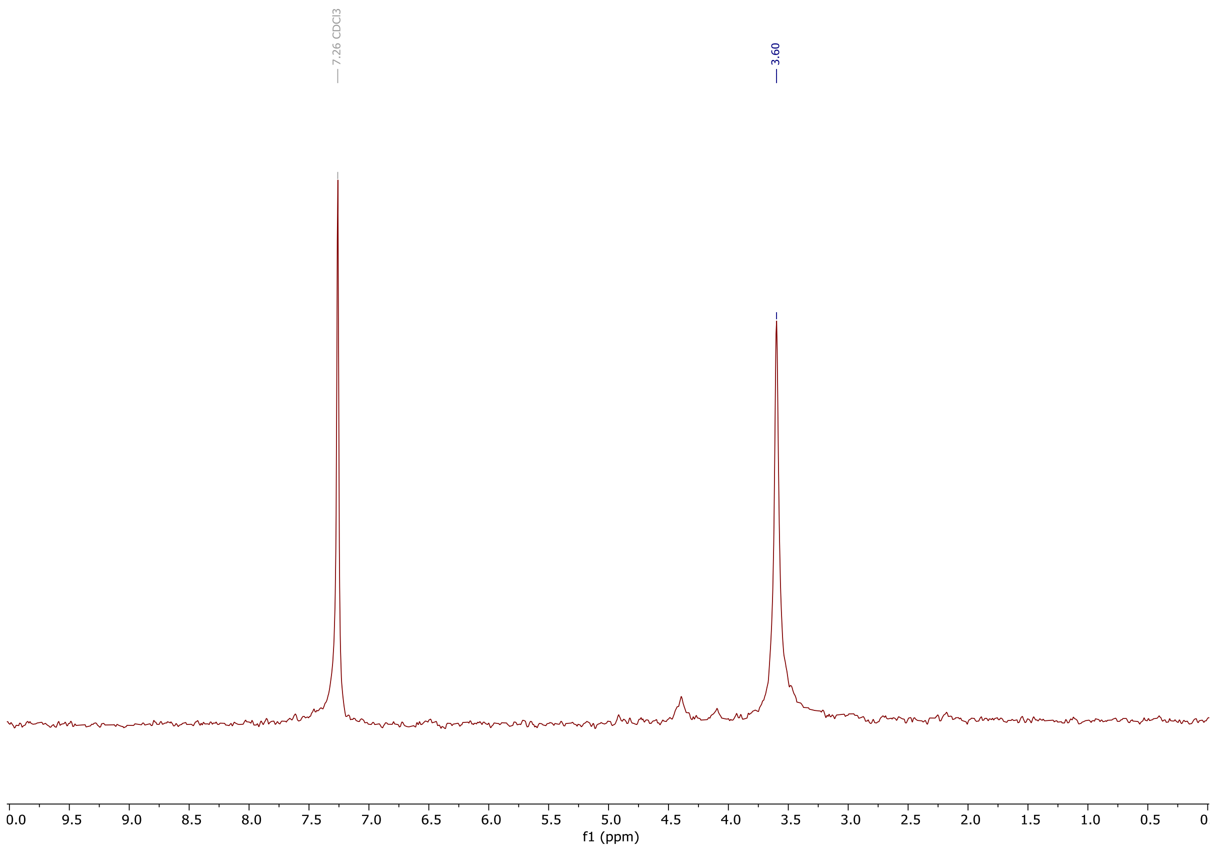

7.26 CDCl3
3.60
0.0
9.5
9.0
8.5
8.0
7.5
7.0
6.5
6.0
5.5
5.0
4.5
4.0
3.5
3.0
2.5
2.0
1.5
1.0
0.5
0
f1 (ppm)

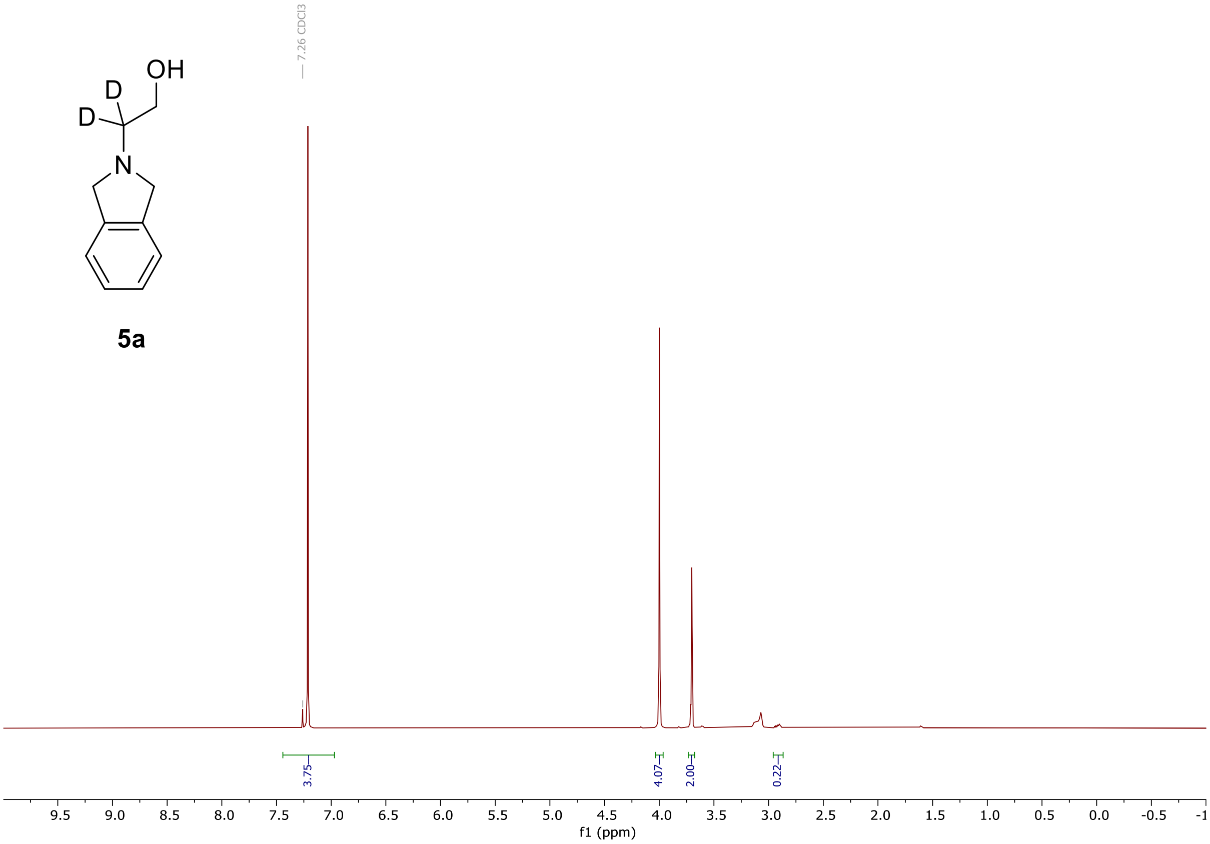
OH
D
D
N
5a
7.26 CDCl3
3.75
4.07
2.00
0.22
9.5 9.0 8.5 8.0 7.5 7.0 6.5 6.0 5.5 5.0 4.5 4.0 3.5 3.0 2.5 2.0 1.5 1.0 0.5 0.0 -0.5
f1 (ppm)

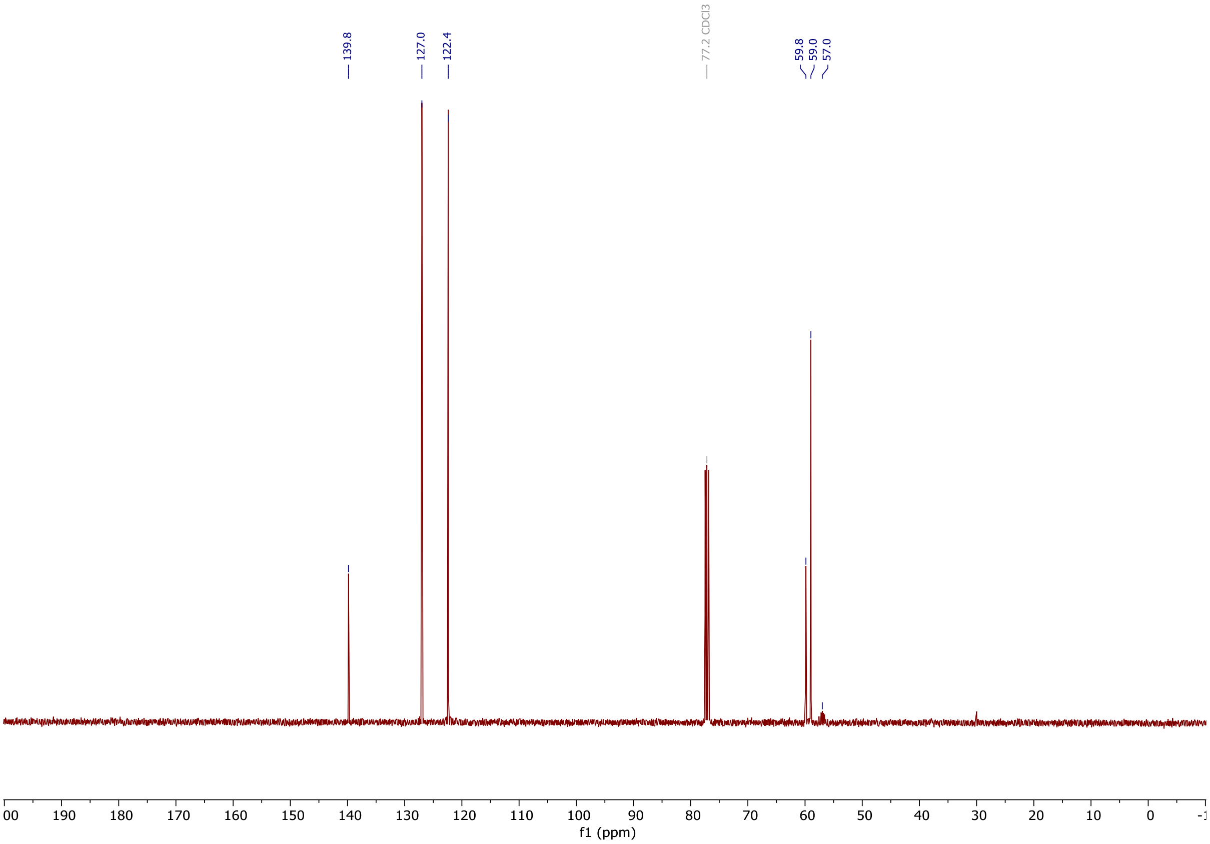
139.8
127.0
122.4
77.2 CDCl3
59.8
59.0
57.0
190 180 170 160 150 140 130 120 110 100 90 80 70 60 50 40 30 20 10 0
f1 (ppm)

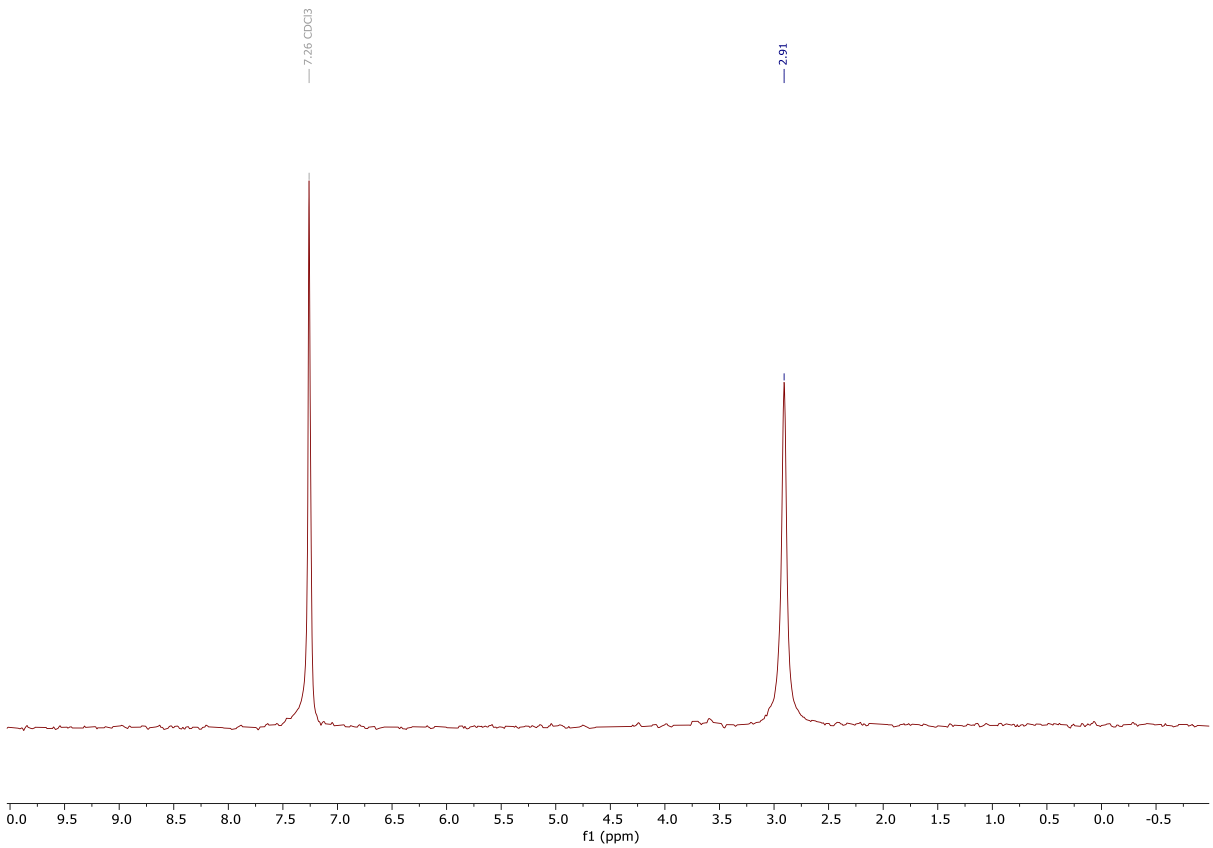
7.26 CDCl3
2.91
0.0
9.5
9.0
8.5
8.0
7.5
7.0
6.5
6.0
5.5
5.0
4.5
4.0
3.5
3.0
2.5
2.0
1.5
1.0
0.5
0.0
-0.5
f1 (ppm)

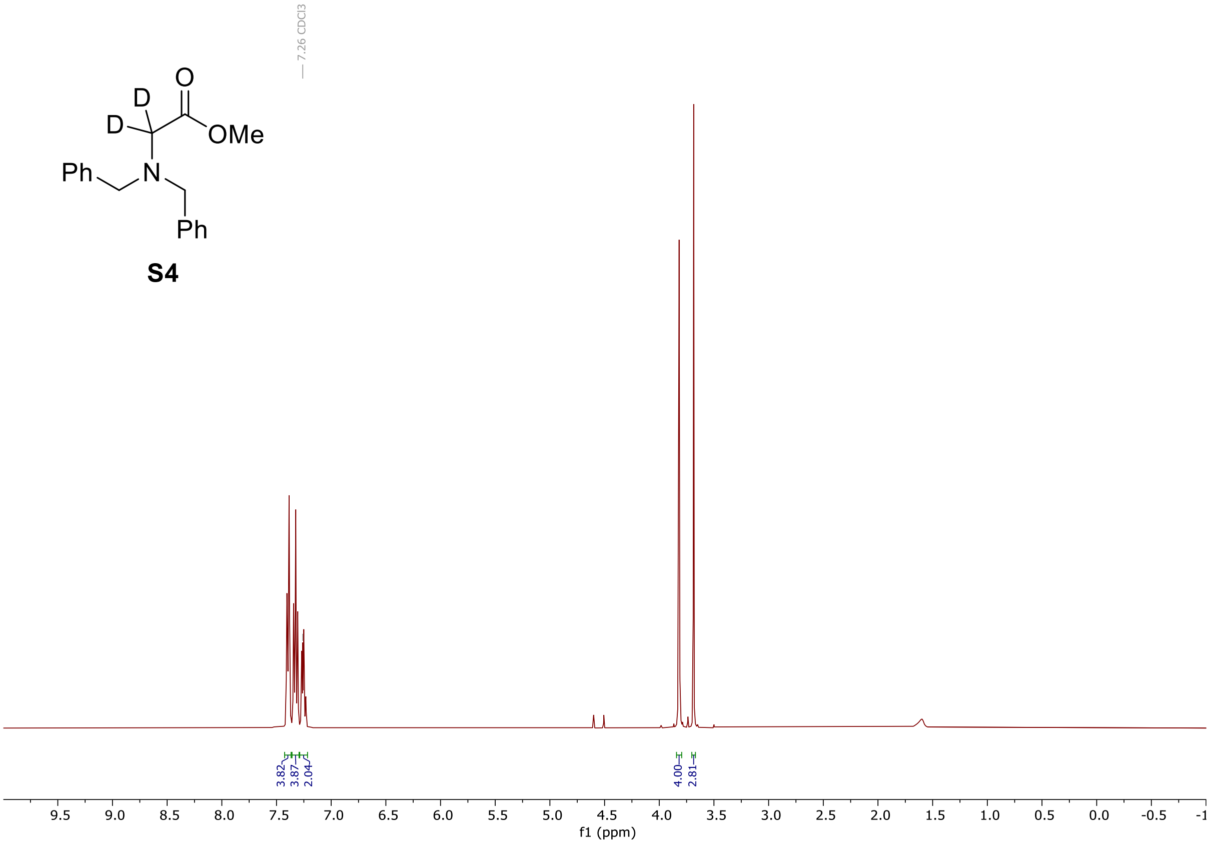
D
D
O
OMe
Ph
N
Ph
S4
7.26 CDCl3
3.82
3.87
2.04
4.00
2.81
9.5 9.0 8.5 8.0 7.5 7.0 6.5 6.0 5.5 5.0 4.5 4.0 3.5 3.0 2.5 2.0 1.5 1.0 0.5 0.0 -0.5
f1 (ppm)

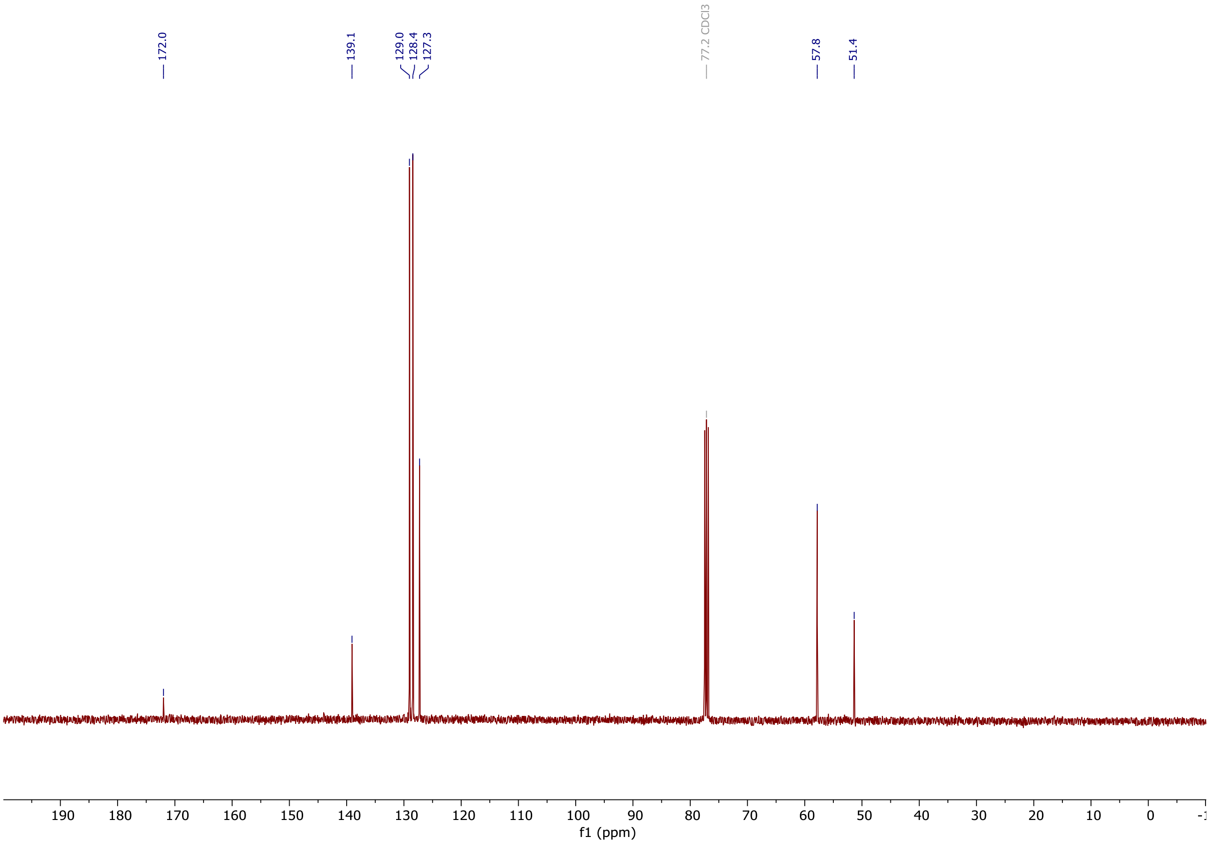
172.0
139.1
129.0
128.4
127.3
77.2 CDCl3
57.8
51.4
190 180 170 160 150 140 130 120 110 100 90 80 70 60 50 40 30 20 10 0
f1 (ppm)

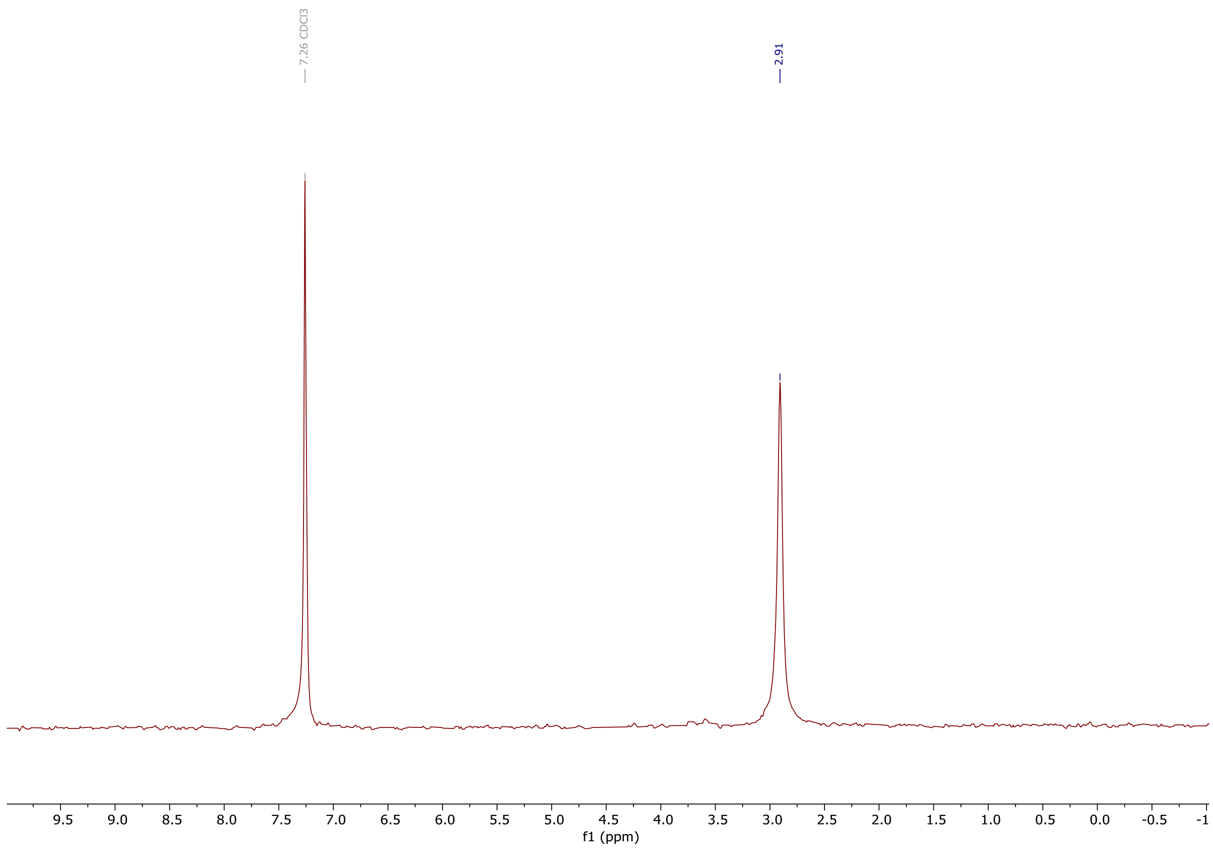

7.26 CDCl3
2.91
9.5
9.0
8.5
8.0
7.5
7.0
6.5
6.0
5.5
5.0
4.5
4.0
3.5
3.0
2.5
2.0
1.5
1.0
0.5
0.0
-0.5
-1
f1 (ppm)

5b

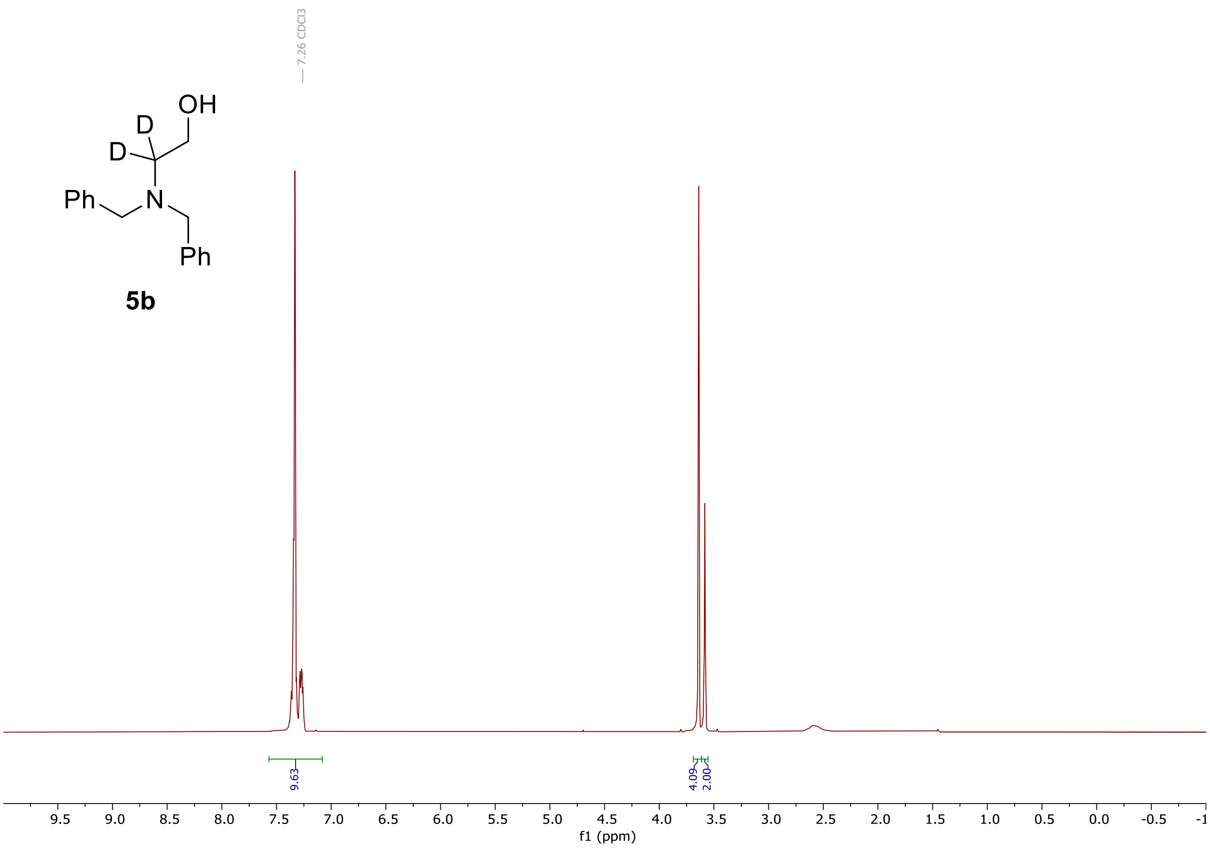
OH
D
D
Ph
N
Ph
7.26 CDCl3
9.63
4.09
2.00
9.5 9.0 8.5 8.0 7.5 7.0 6.5 6.0 5.5 5.0 4.5 4.0 3.5 3.0 2.5 2.0 1.5 1.0 0.5 0.0 -0.5 -1
f1 (ppm)

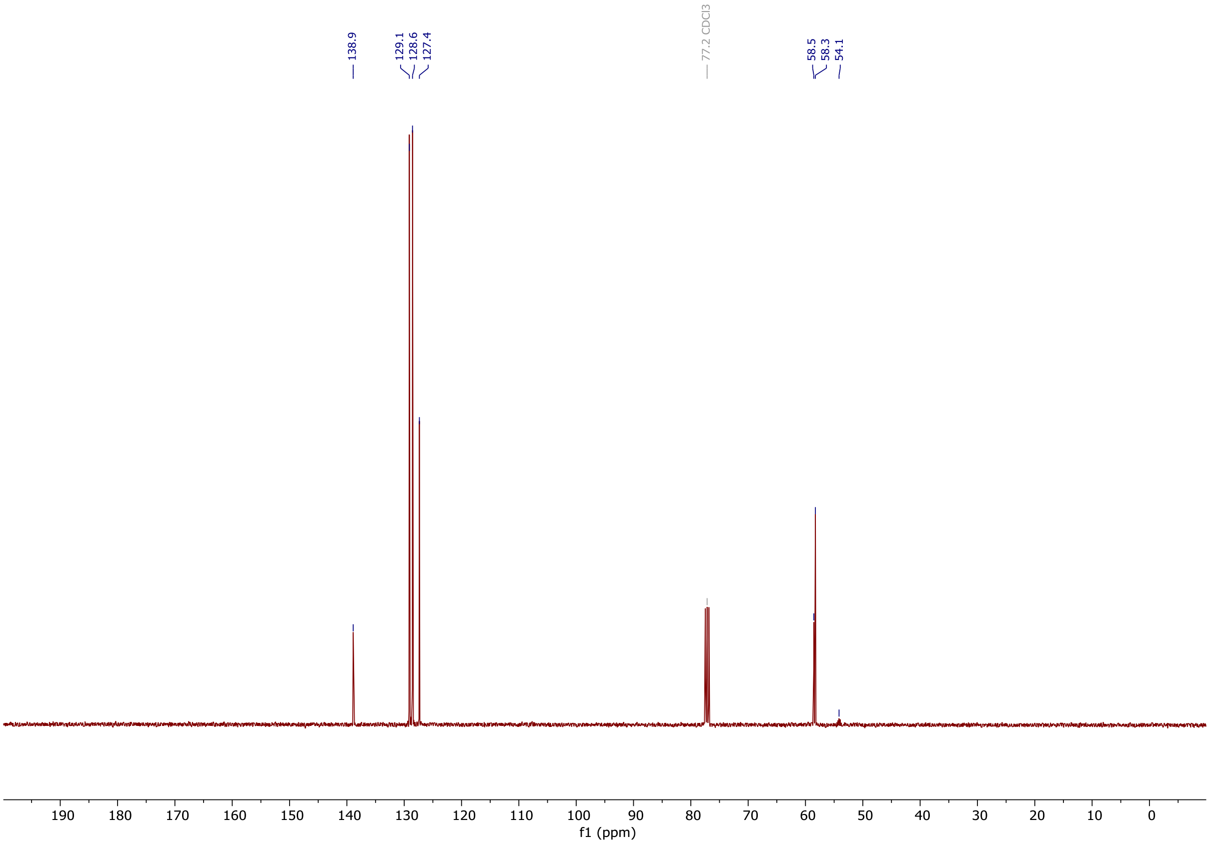
138.9
129.1
128.6
127.4
77.2 CDCl3
58.5
58.3
54.1
190 180 170 160 150 140 130 120 110 100 90 80 70 60 50 40 30 20 10 0
f1 (ppm)

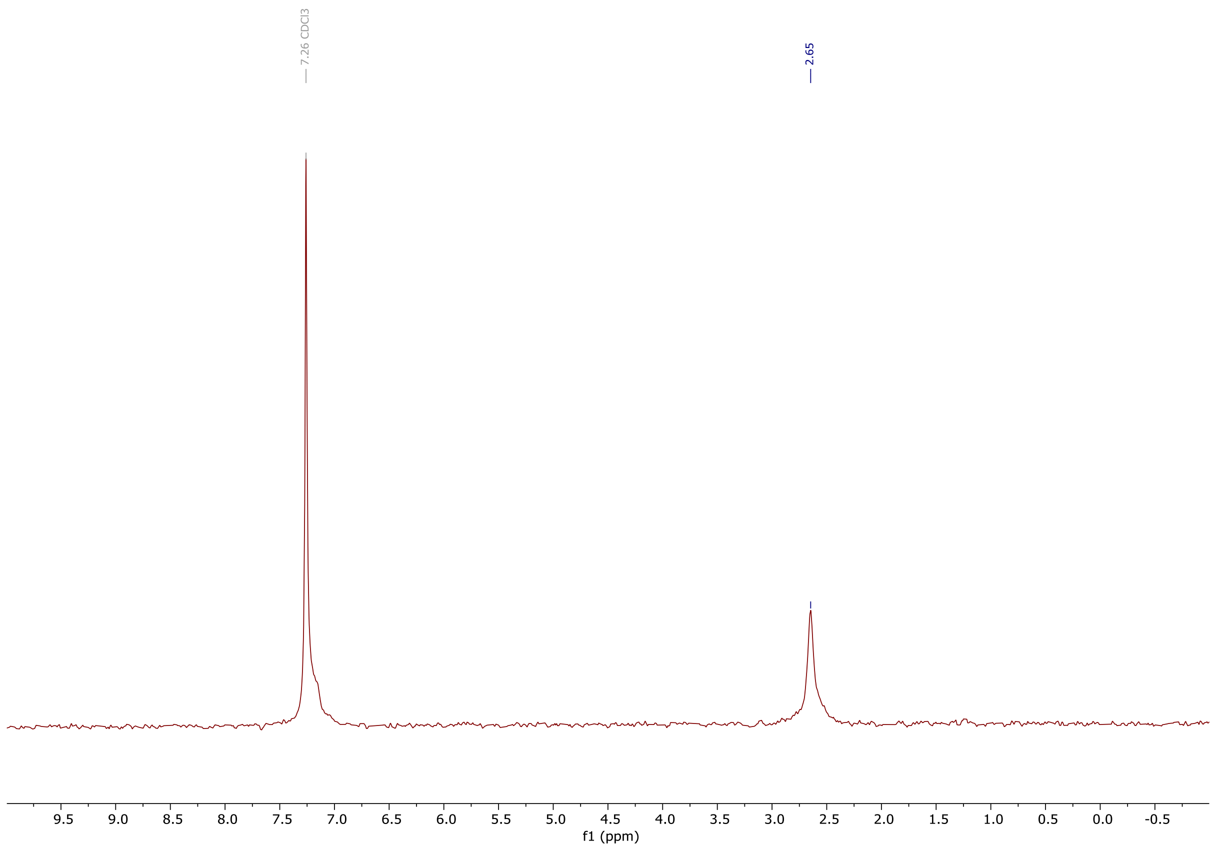

7.26 CDCl3
2.65
9.5
9.0
8.5
8.0
7.5
7.0
6.5
6.0
5.5
5.0
4.5
4.0
3.5
3.0
2.5
2.0
1.5
1.0
0.5
0.0
-0.5
f1 (ppm)

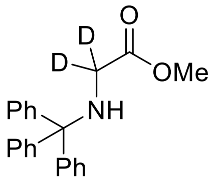
D
D
O
OMe
NH
Ph
Ph
Ph

S5

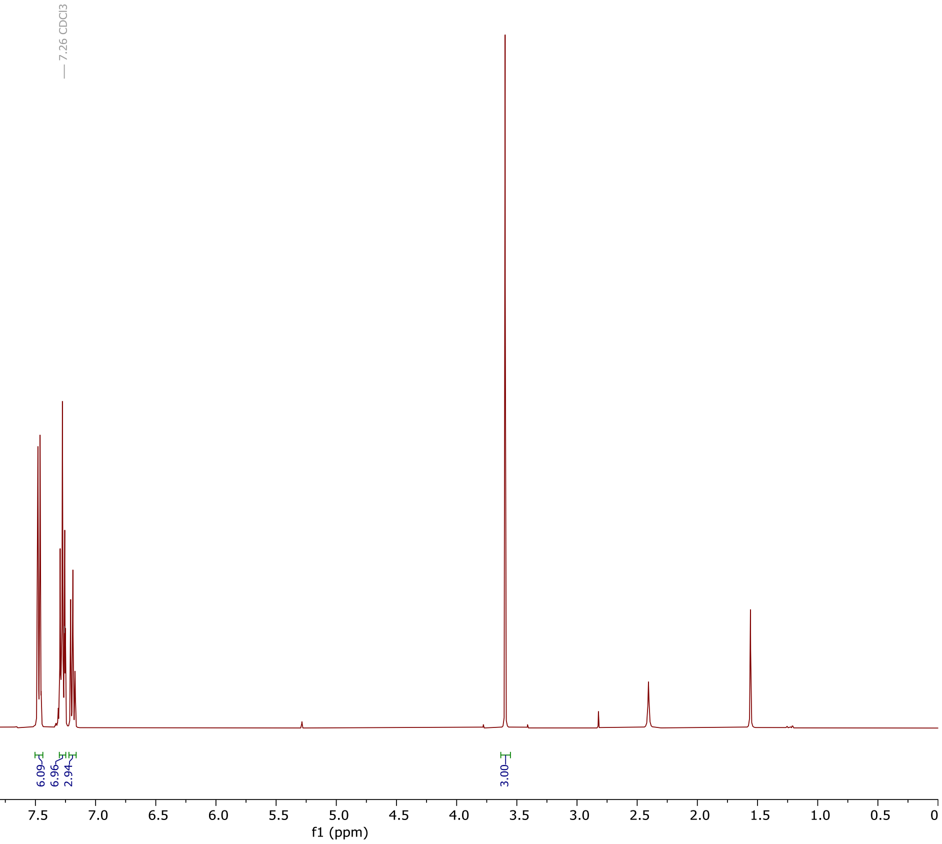
7.26 CDCl3
6.09
6.96
2.94
3.00
9.5 9.0 8.5 8.0 7.5 7.0 6.5 6.0 5.5 5.0 4.5 4.0 3.5 3.0 2.5 2.0 1.5 1.0 0.5 0
f1 (ppm)

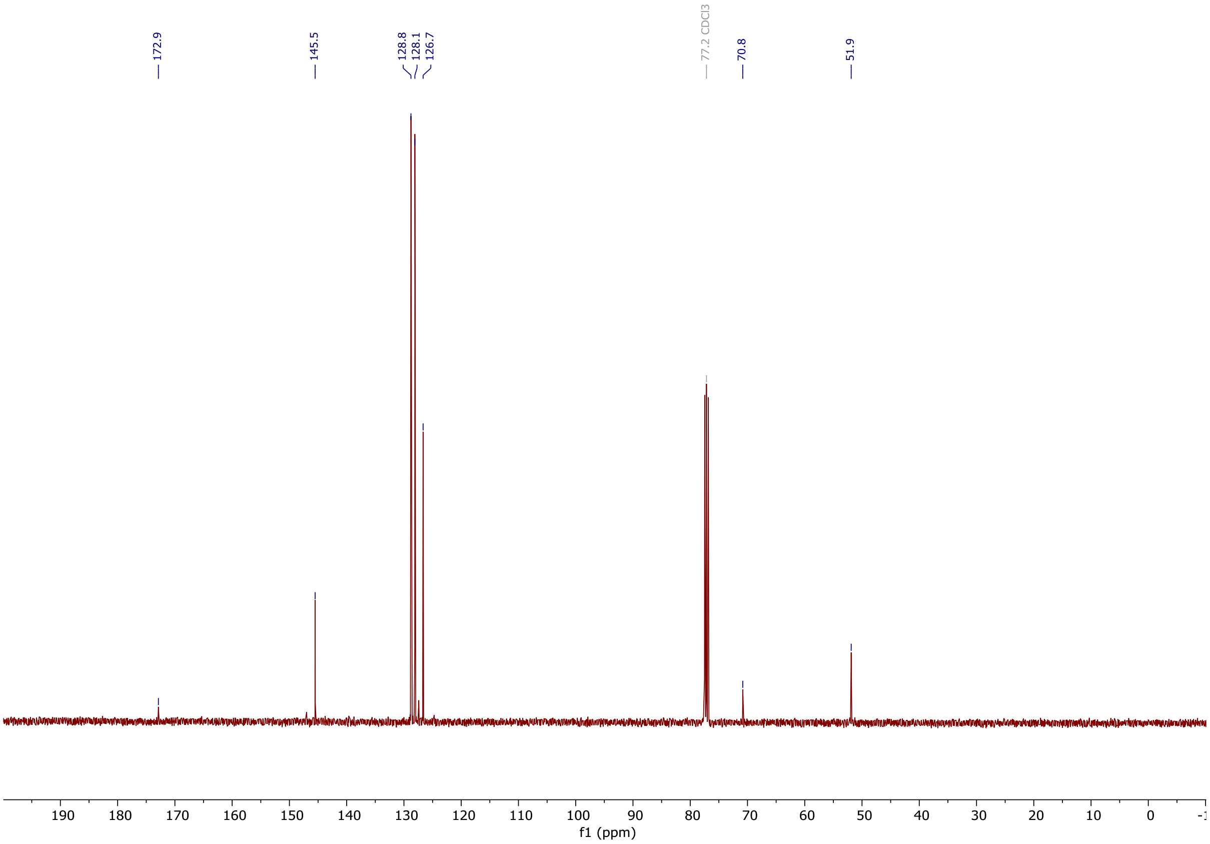
172.9
145.5
128.8
128.1
126.7
77.2 CDCl3
70.8
51.9
190 180 170 160 150 140 130 120 110 100 90 80 70 60 50 40 30 20 10 0
f1 (ppm)

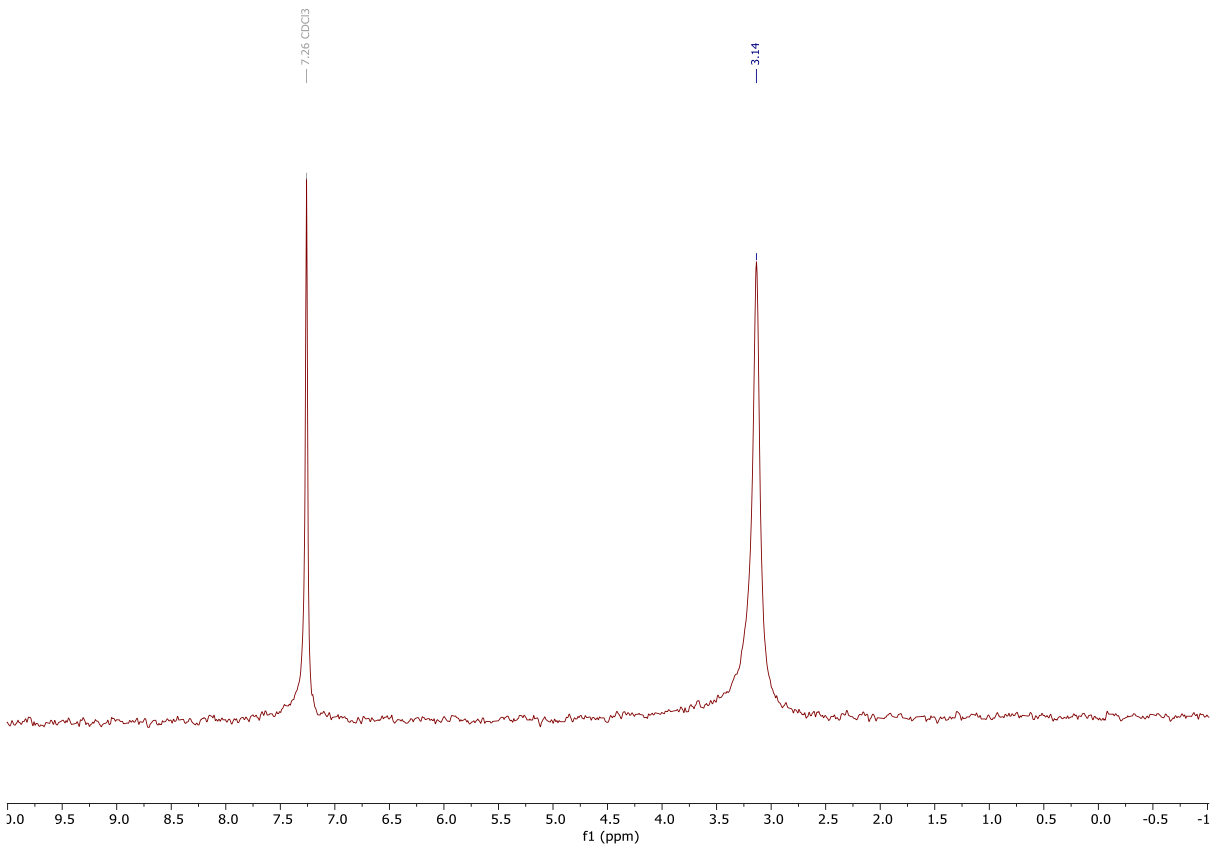
7.26 CDCl3
3.14
0.0 9.5 9.0 8.5 8.0 7.5 7.0 6.5 6.0 5.5 5.0 4.5 4.0 3.5 3.0 2.5 2.0 1.5 1.0 0.5 0.0 -0.5 -1
f1 (ppm)

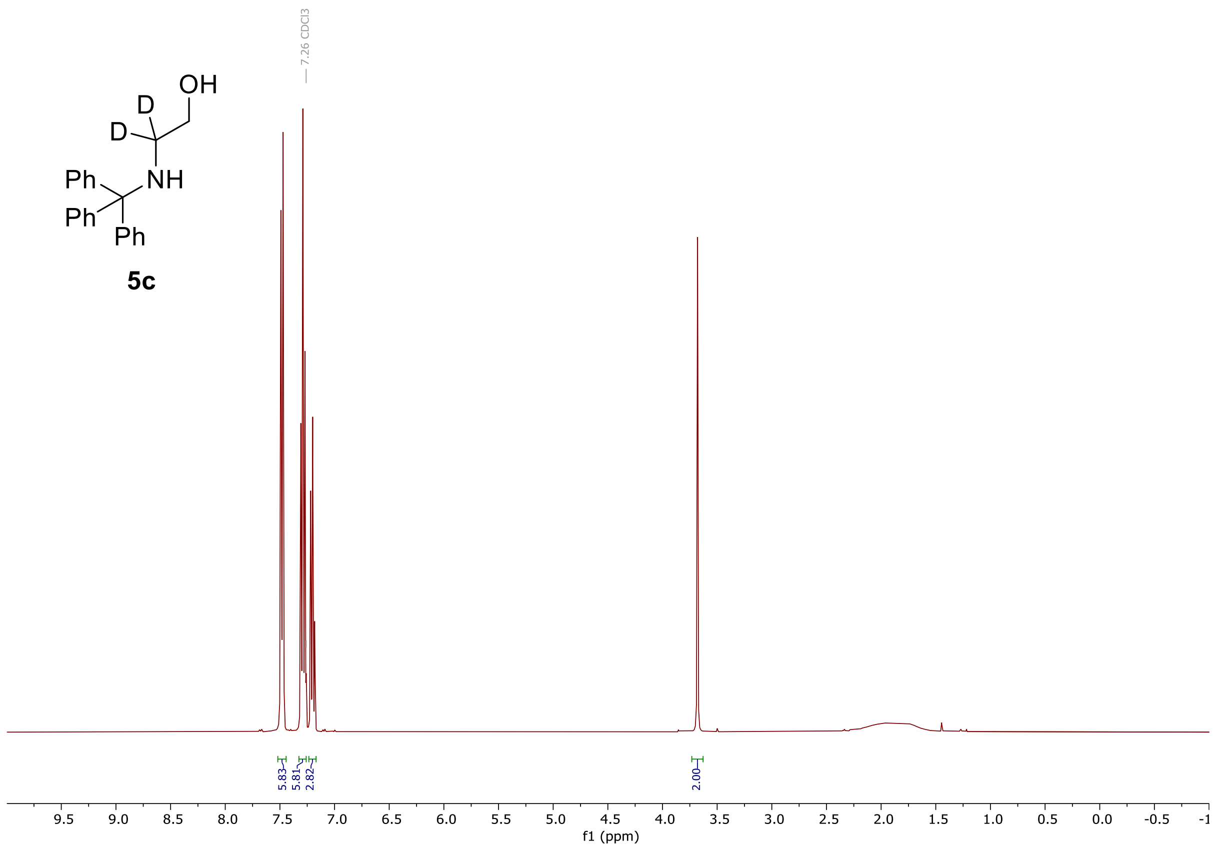
OH
D
D
NH
Ph
Ph
Ph
5c
7.26 CDCl3
5.83
5.81
2.82
2.00
9.5 9.0 8.5 8.0 7.5 7.0 6.5 6.0 5.5 5.0 4.5 4.0 3.5 3.0 2.5 2.0 1.5 1.0 0.5 0.0 -0.5
f1 (ppm)

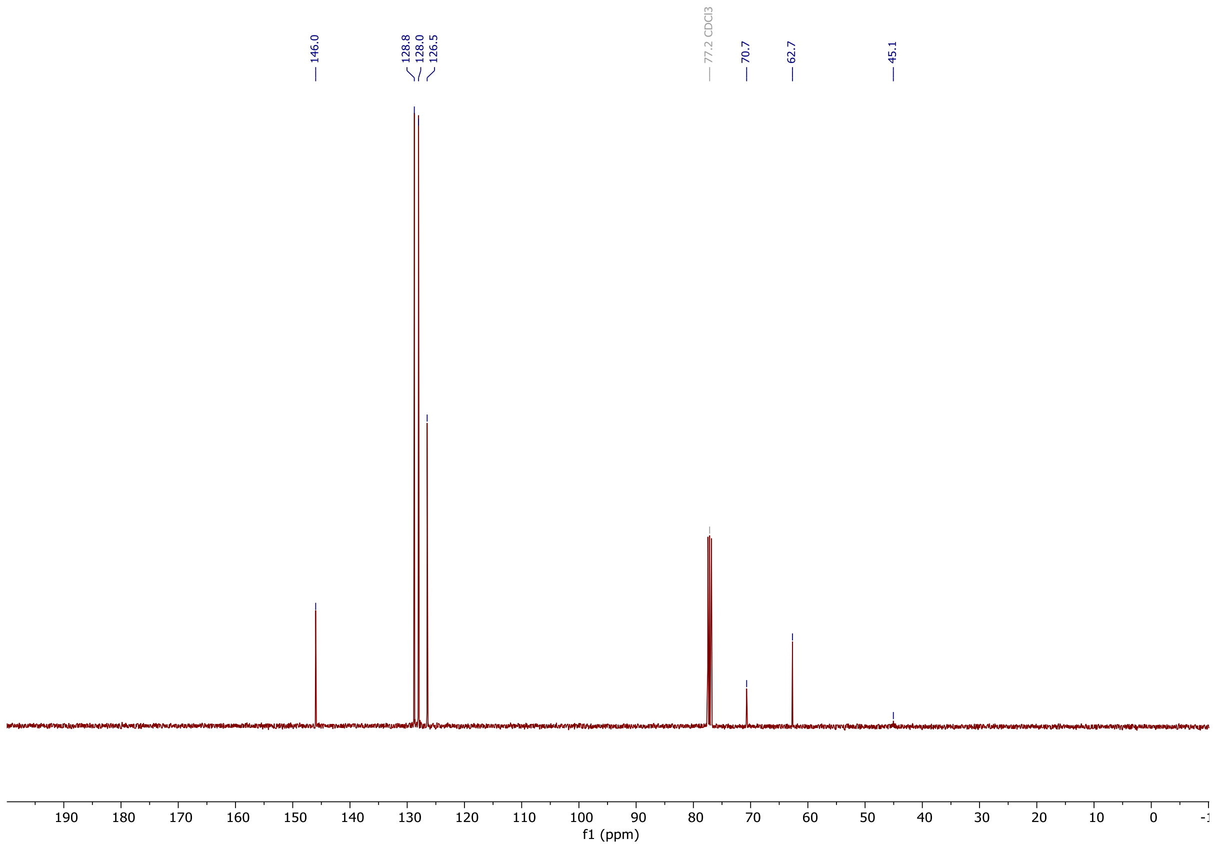
146.0
128.8
128.0
126.5
77.2 CDCl3
70.7
62.7
45.1
190 180 170 160 150 140 130 120 110 100 90 80 70 60 50 40 30 20 10 0
f1 (ppm)

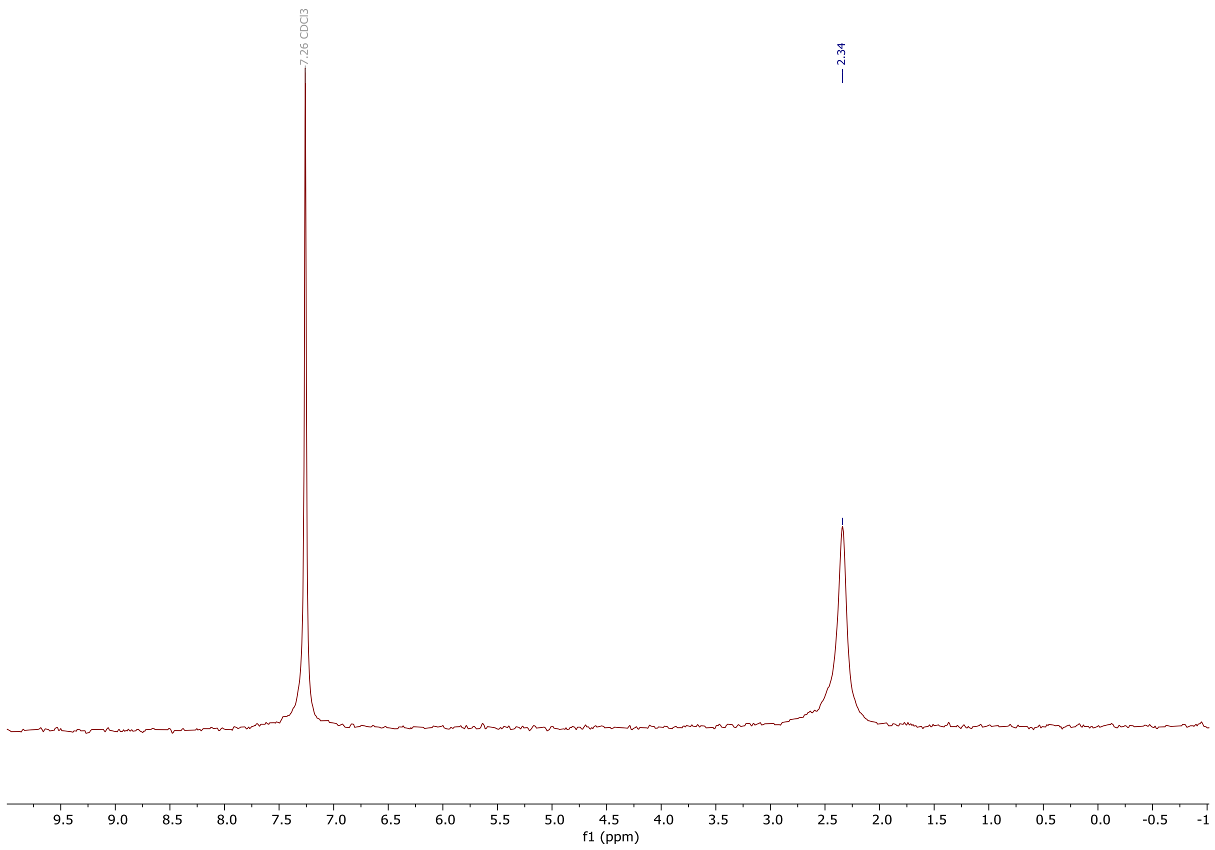

7.26 CDCl3
2.34
9.5
9.0
8.5
8.0
7.5
7.0
6.5
6.0
5.5
5.0
4.5
4.0
3.5
3.0
2.5
2.0
1.5
1.0
0.5
0.0
-0.5
-1
f1 (ppm)

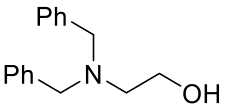
Ph
Ph
N
OH

1b

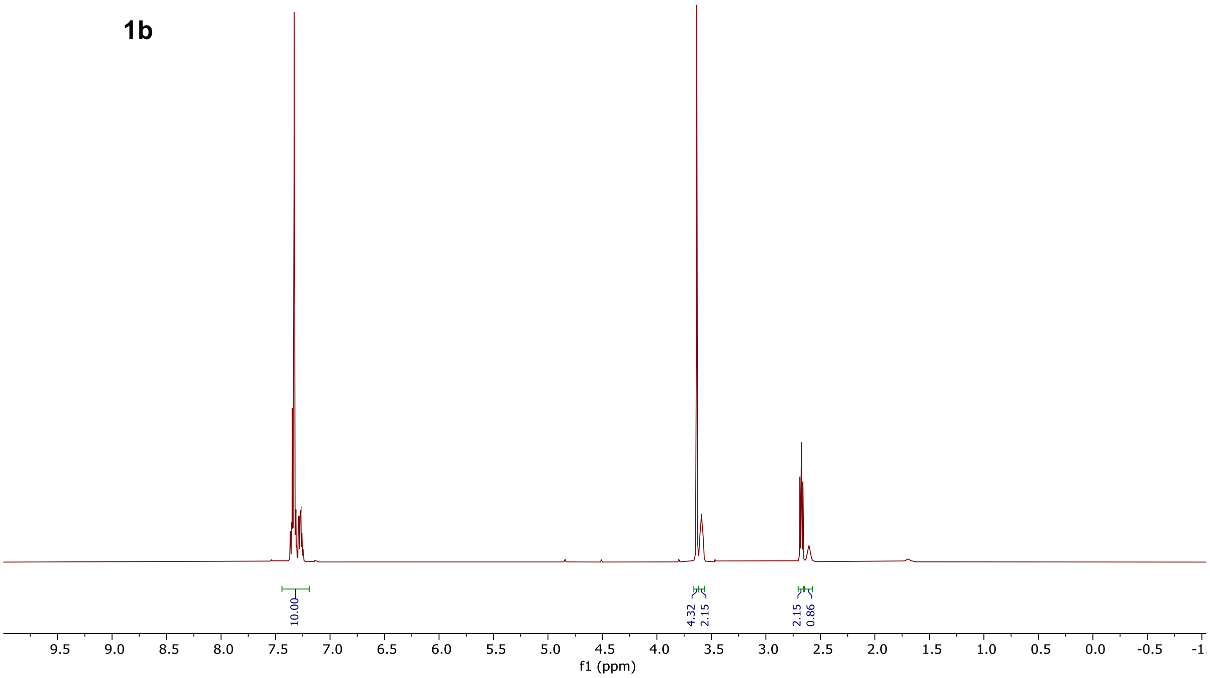
7.26 CDCl3
10.00
4.32
2.15
2.15
0.86
9.5 9.0 8.5 8.0 7.5 7.0 6.5 6.0 5.5 5.0 4.5 4.0 3.5 3.0 2.5 2.0 1.5 1.0 0.5 0.0 -0.5 -1
f1 (ppm)

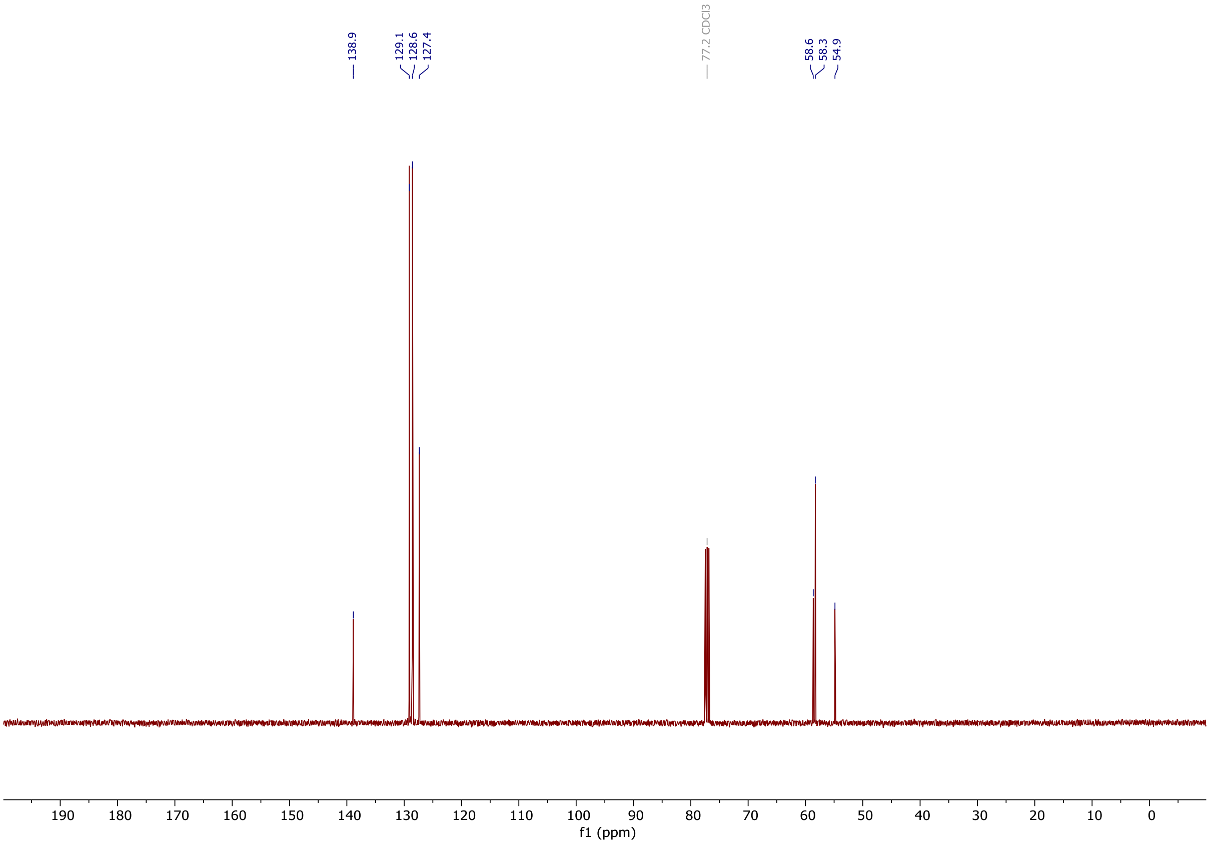
138.9
129.1
128.6
127.4
77.2 CDCl3
58.6
58.3
54.9
190 180 170 160 150 140 130 120 110 100 90 80 70 60 50 40 30 20 10 0
f1 (ppm)

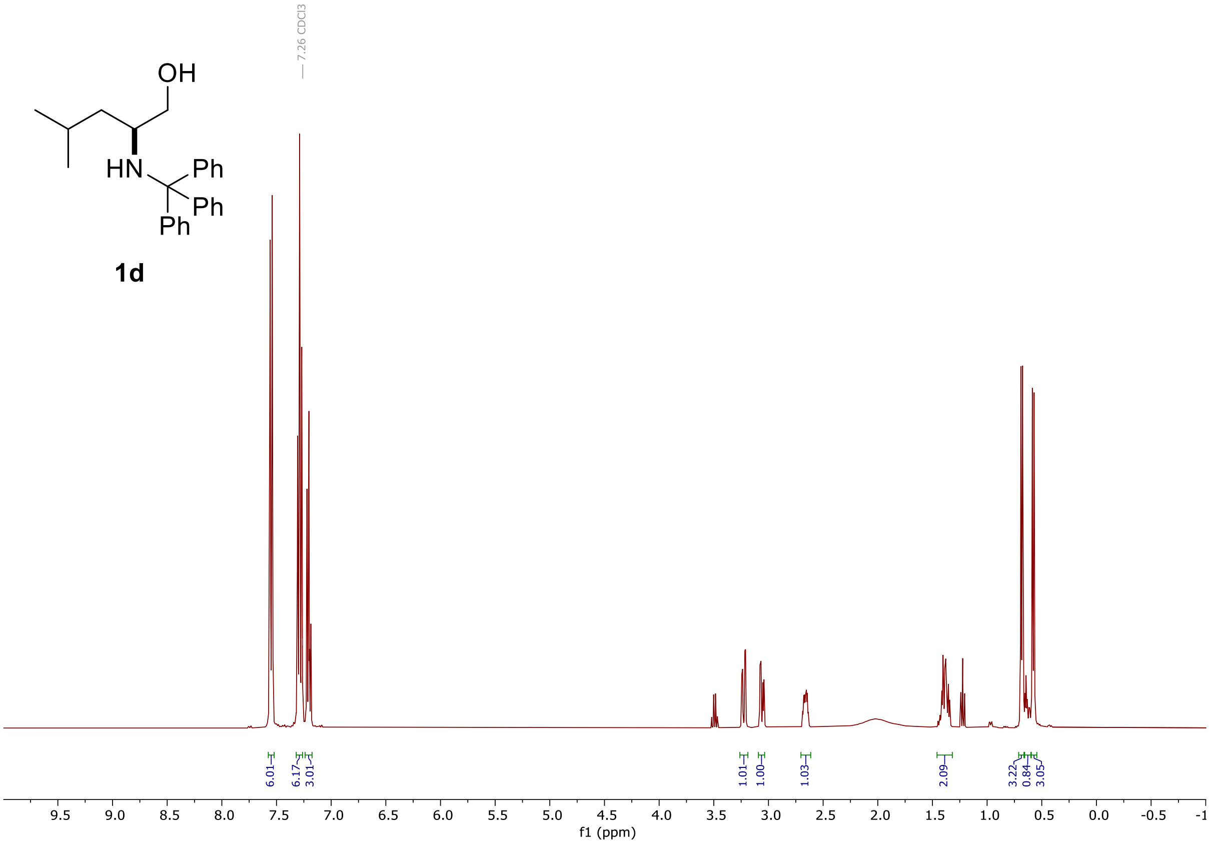

1d

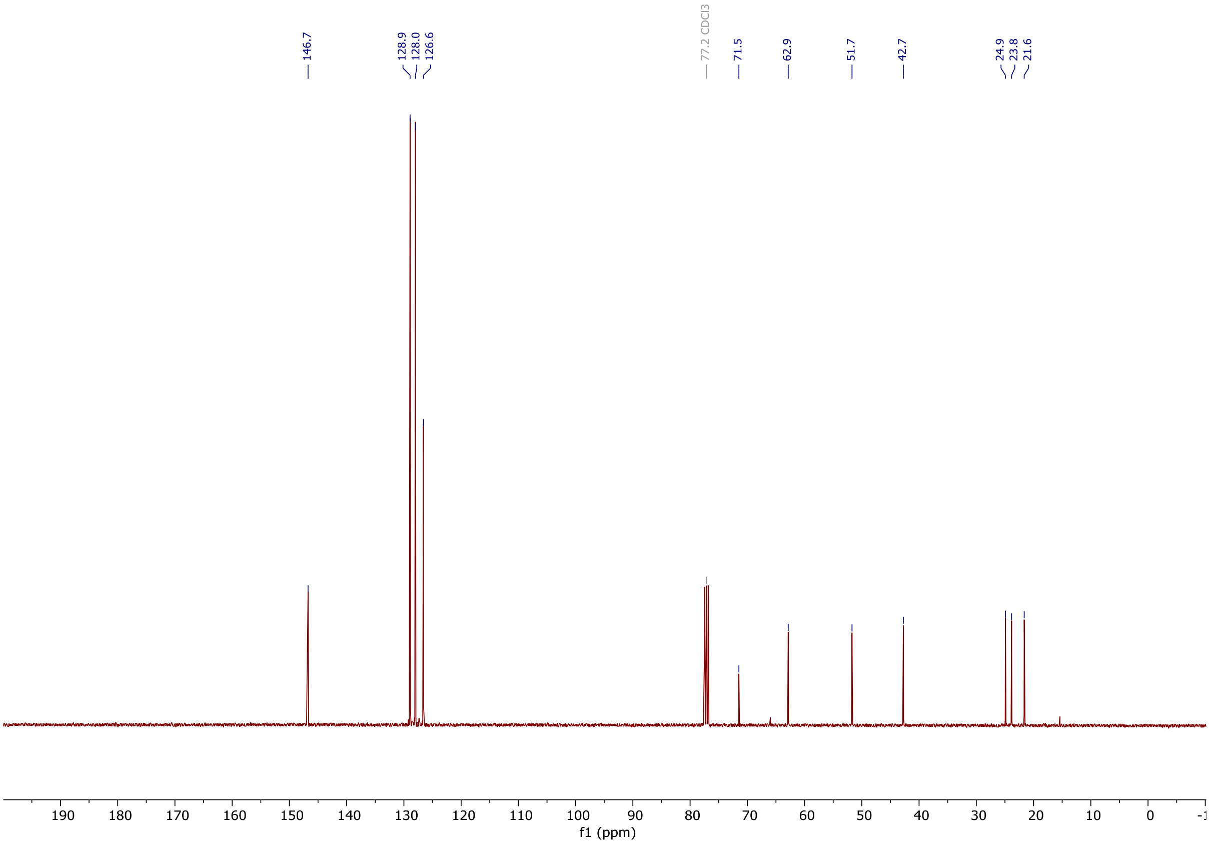

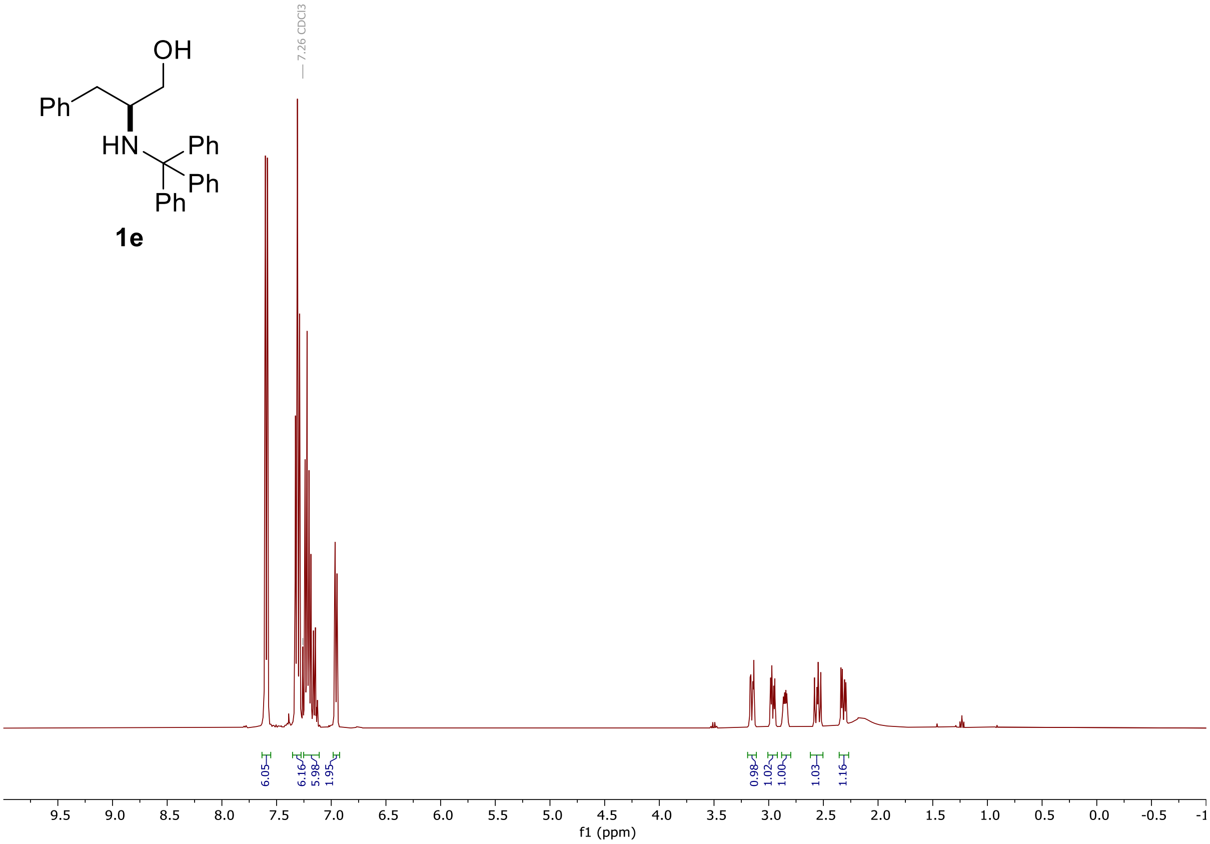
OH
Ph
HN
Ph
Ph
Ph
1e
7.26 CDCl3
6.05
6.16
5.98
1.95
0.98
1.02
1.00
1.03
1.16
9.5
9.0
8.5
8.0
7.5
7.0
6.5
6.0
5.5
5.0
4.5
4.0
3.5
3.0
2.5
2.0
1.5
1.0
0.5
0.0
-0.5
f1 (ppm)

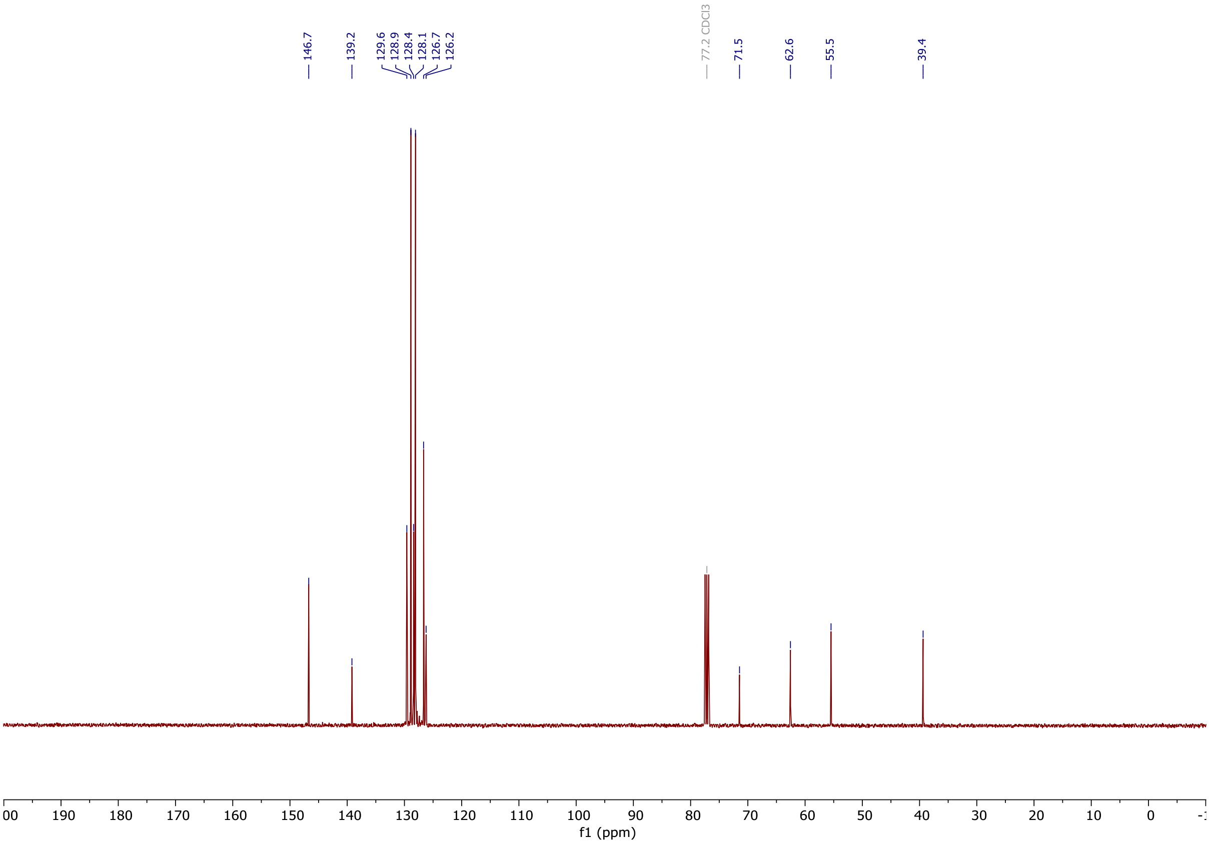
146.7
139.2
129.6
128.9
128.4
128.1
126.7
126.2
77.2 CDCl3
71.5
62.6
55.5
39.4
190
180
170
160
150
140
130
120
110
100
90
80
70
60
50
40
30
20
10
0
f1 (ppm)

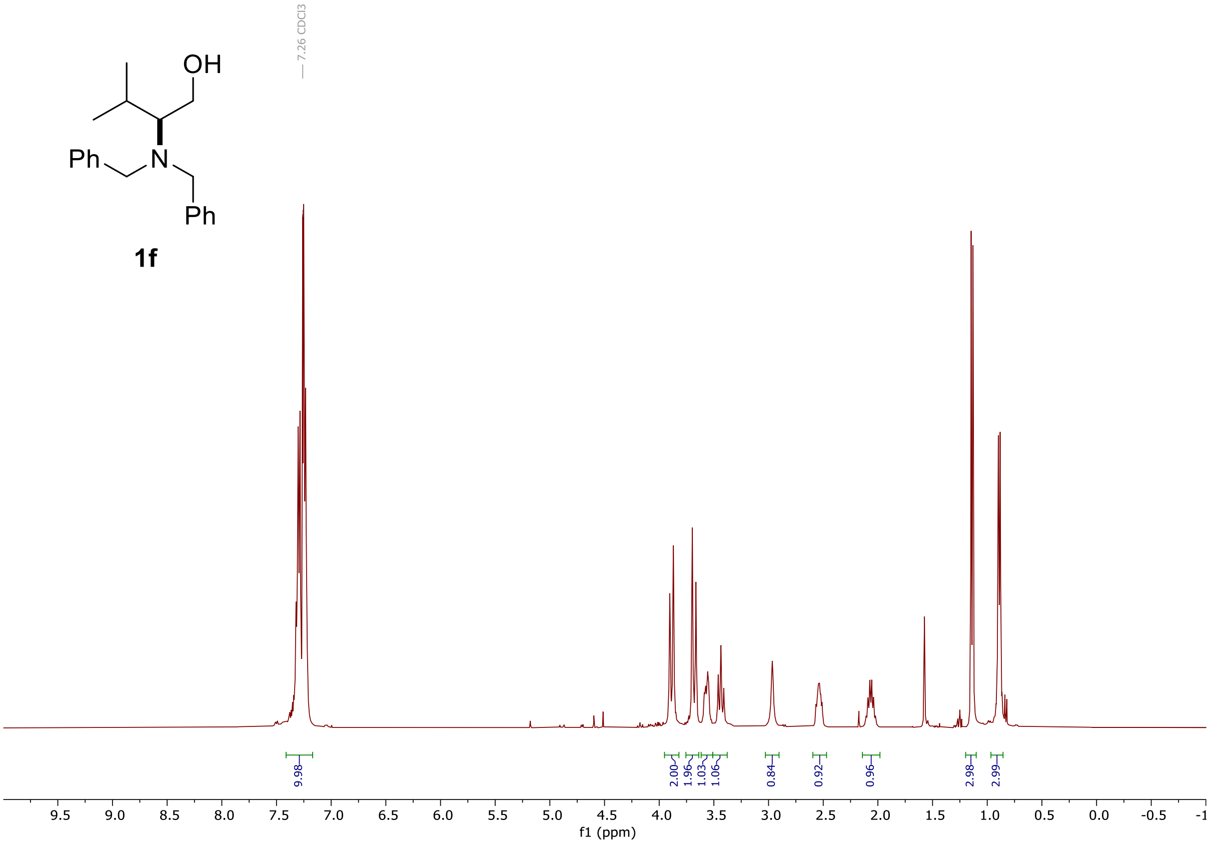
OH
Ph
N
Ph
7.26 CDCl3
9.98
2.00
1.96
1.03
1.06
0.84
0.92
0.96
2.98
2.99
f1 (ppm)

1f

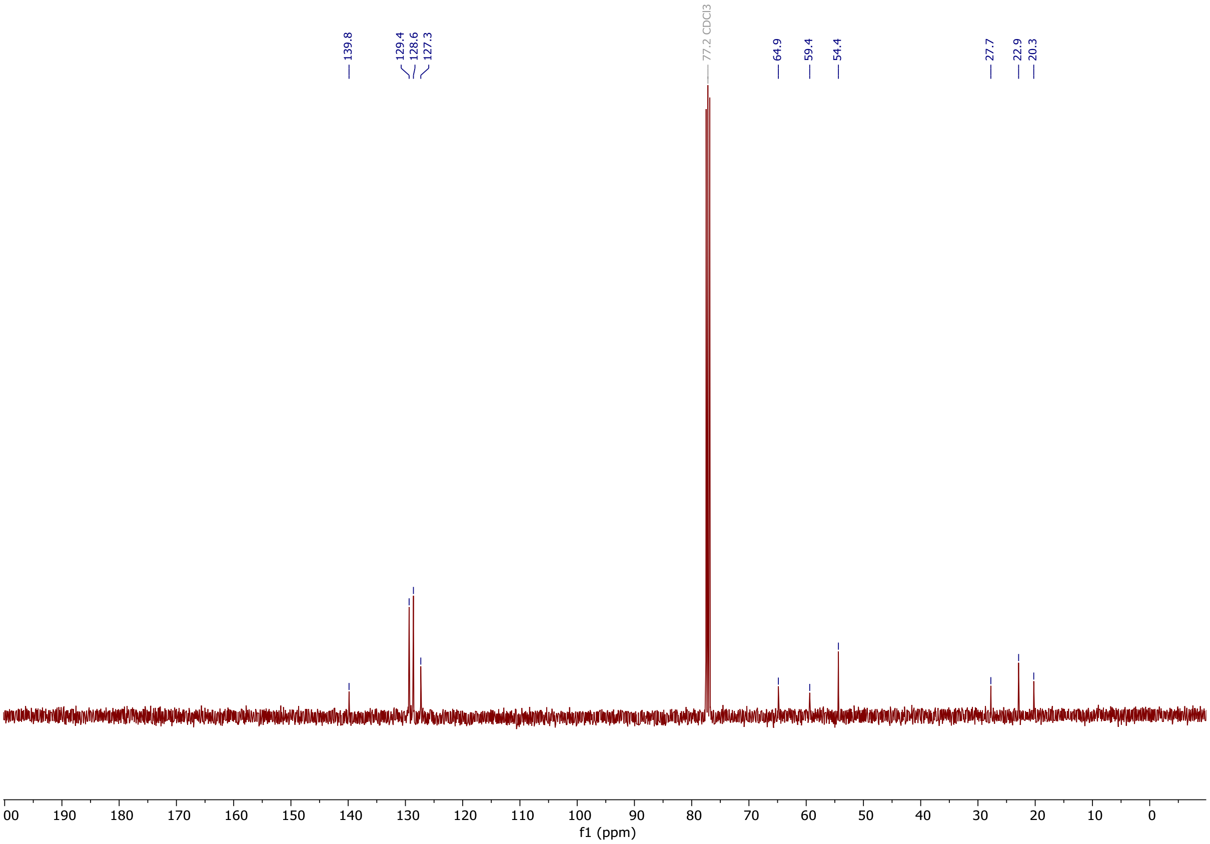
139.8
129.4
128.6
127.3
77.2 CDCl3
64.9
59.4
54.4
27.7
22.9
20.3
f1 (ppm)

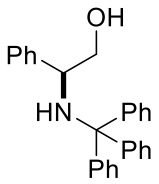
OH
Ph
HN
Ph
Ph
Ph

1g

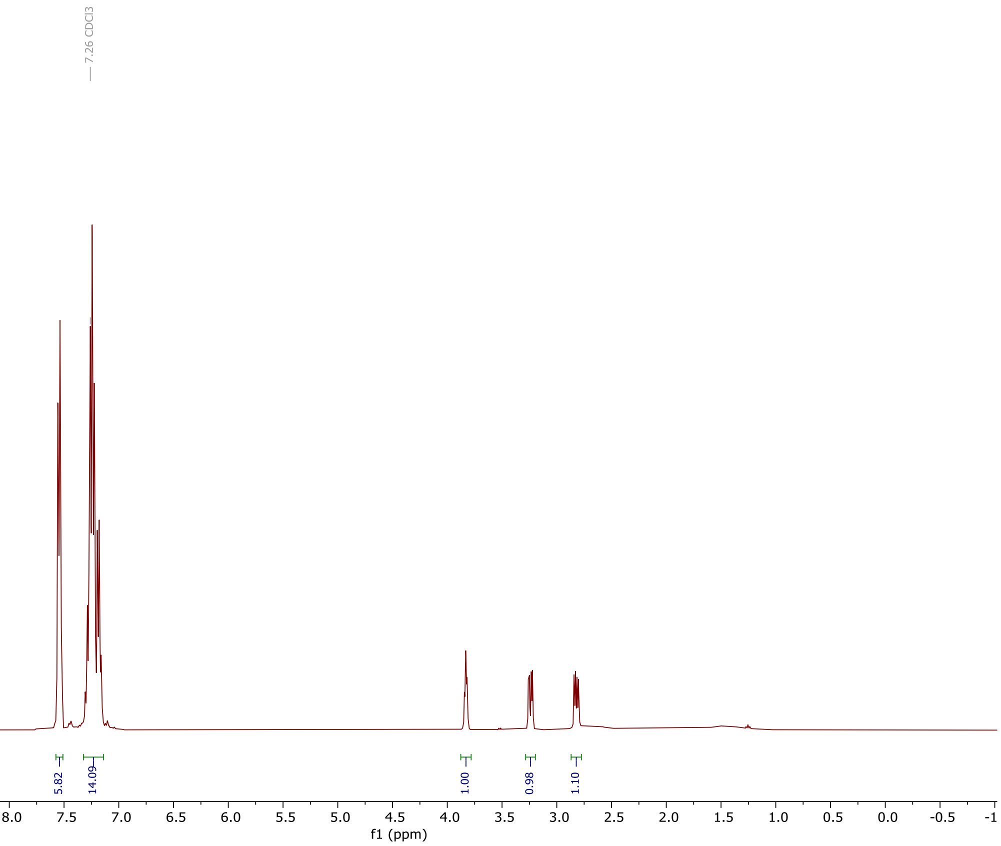
7.26 CDCl3
5.82
14.09
1.00
0.98
1.10
9.5
9.0
8.5
8.0
7.5
7.0
6.5
6.0
5.5
5.0
4.5
4.0
3.5
3.0
2.5
2.0
1.5
1.0
0.5
0.0
-0.5
-1
f1 (ppm)

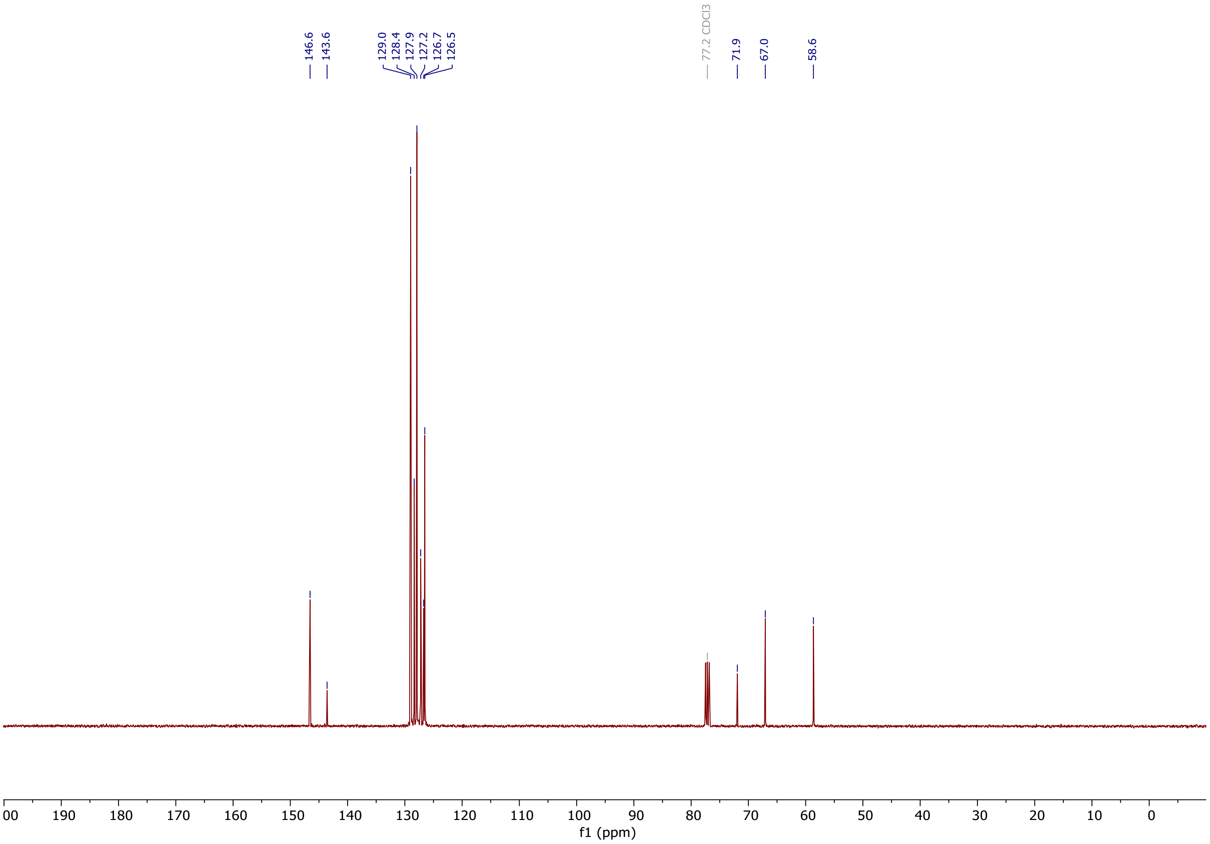
146.6
143.6
129.0
128.4
127.9
127.2
126.7
126.5
77.2 CDCl3
71.9
67.0
58.6
00
190
180
170
160
150
140
130
120
110
100
90
80
70
60
50
40
30
20
10
0
f1 (ppm)

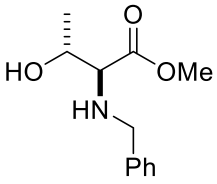
O
HO
OMe
HN
Ph

S6

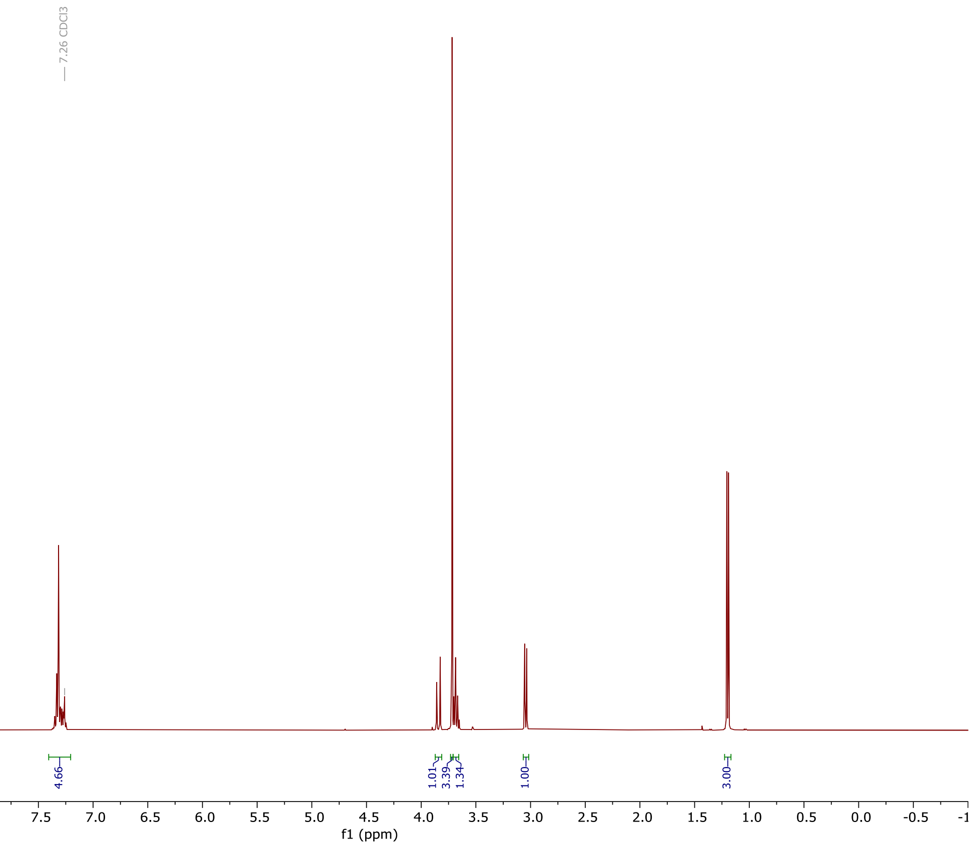
7.26 CDCl3
4.66
1.01
3.39
1.34
1.00
3.00
f1 (ppm)

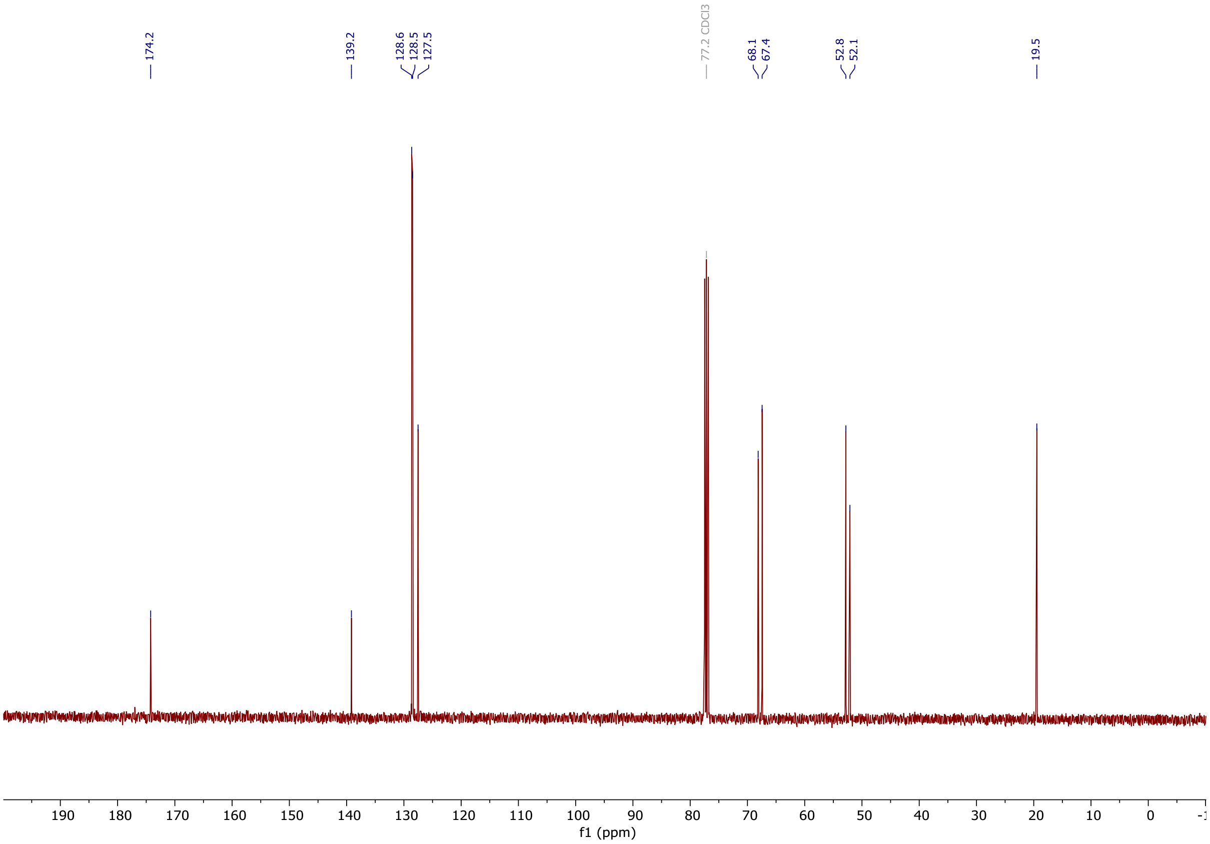
174.2
139.2
128.6
128.5
127.5
77.2 CDCl3
68.1
67.4
52.8
52.1
19.5
f1 (ppm)

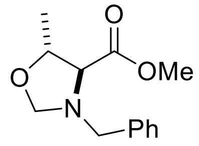
O
OMe
O
N
Ph

S7

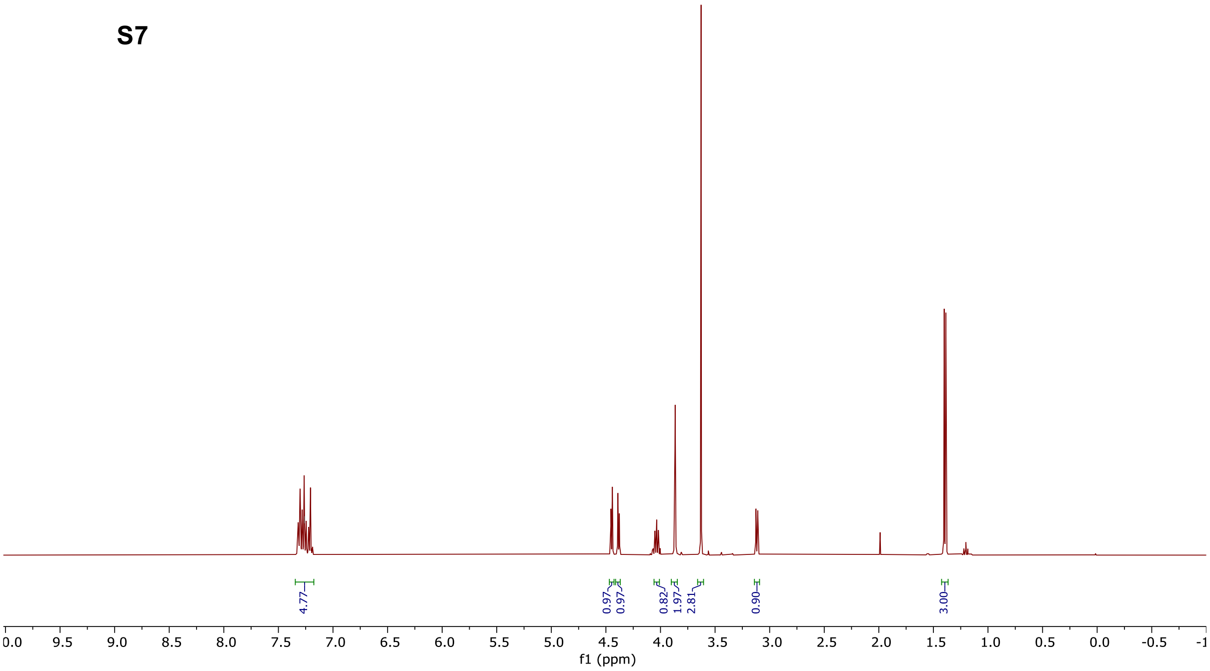
7.26 CDCl3
4.77
0.97
0.97
0.82
1.97
2.81
0.90
3.00
0.0 9.5 9.0 8.5 8.0 7.5 7.0 6.5 6.0 5.5 5.0 4.5 4.0 3.5 3.0 2.5 2.0 1.5 1.0 0.5 0.0 -0.5
f1 (ppm)

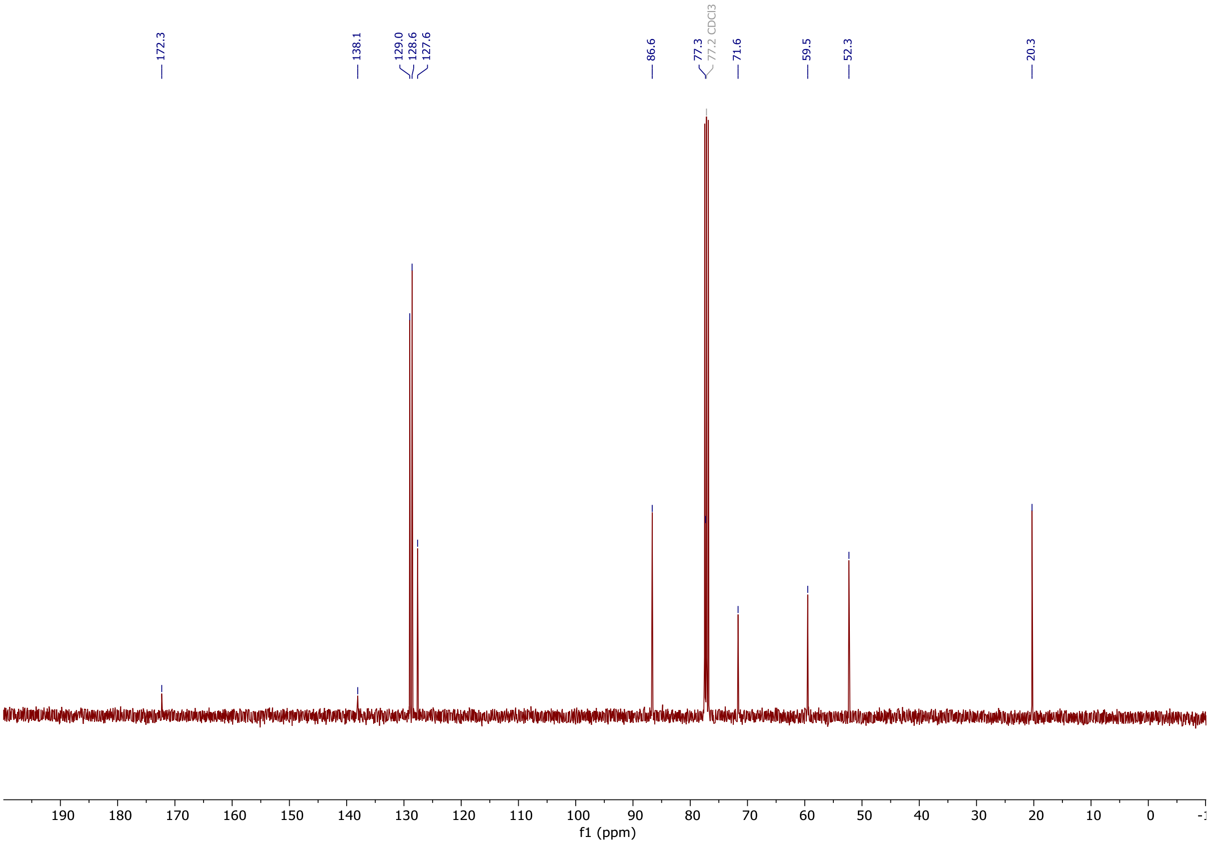
172.3
138.1
129.0
128.6
127.6
86.6
77.3
77.2 CDCl3
71.6
59.5
52.3
20.3
190 180 170 160 150 140 130 120 110 100 90 80 70 60 50 40 30 20 10 0
f1 (ppm)

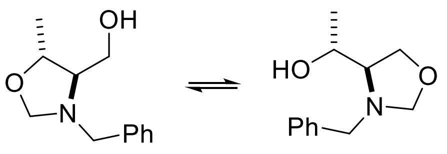
OH
O
N
Ph
HO
O
N
Ph

1h **1h'**

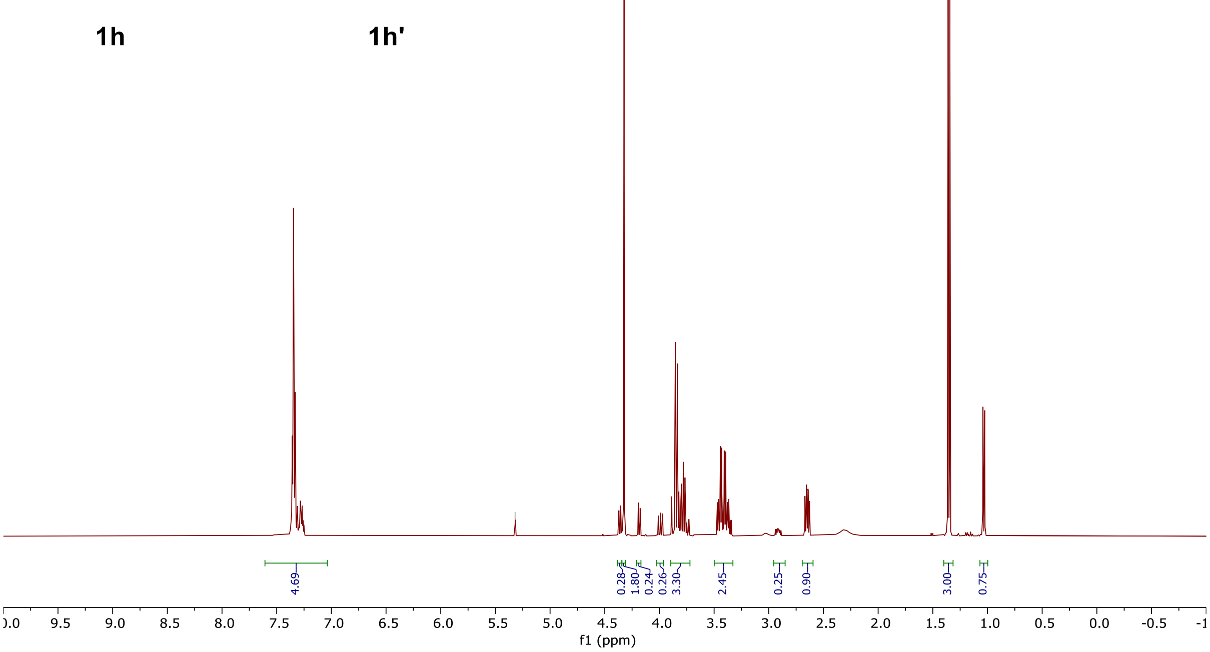
5.32 CD2Cl2
4.69
0.28
1.80
0.24
0.26
3.30
2.45
0.25
0.90
3.00
0.75
f1 (ppm)

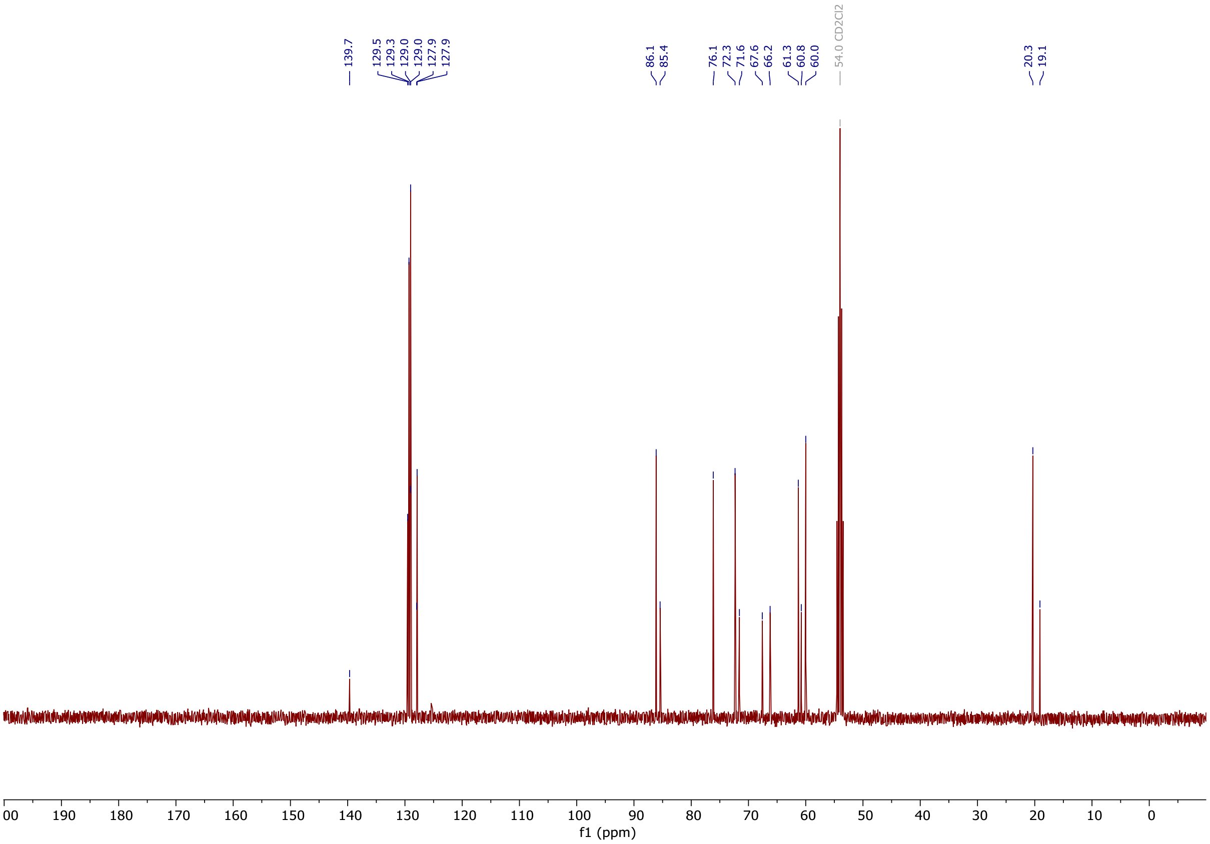
139.7
129.5
129.3
129.0
129.0
127.9
127.9
86.1
85.4
76.1
72.3
71.6
67.6
66.2
61.3
60.8
60.0
54.0 CD2Cl2
20.3
19.1
f1 (ppm)

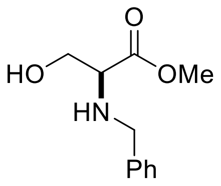

HO
OMe
O
HN
Ph

S8

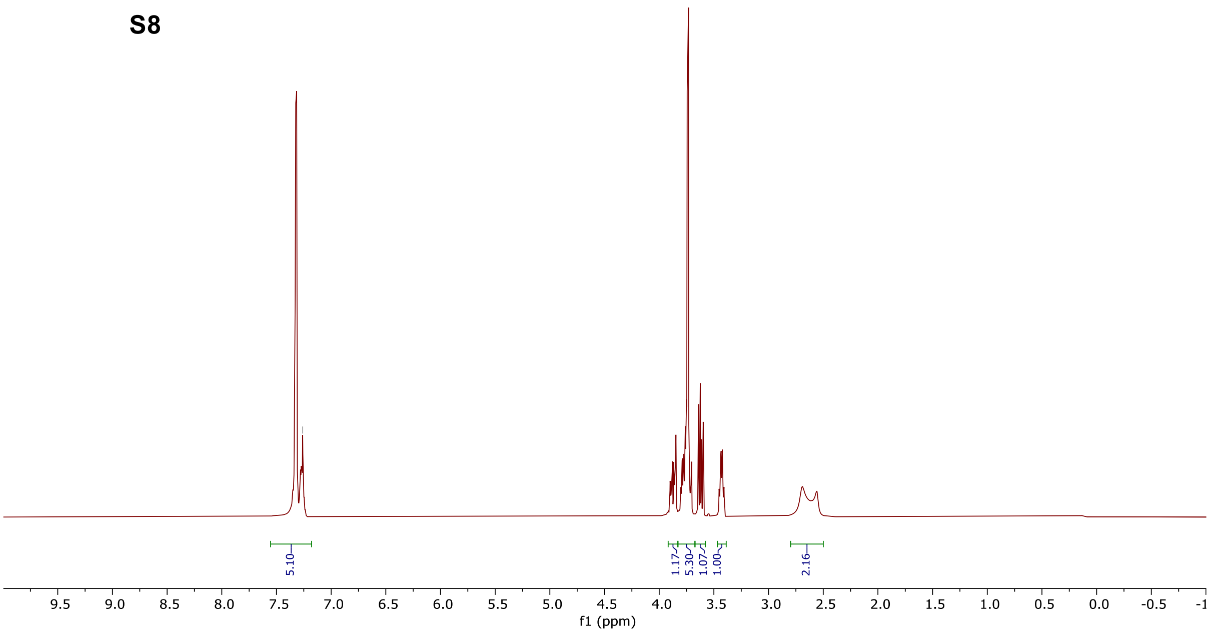

7.26 CDCl3
5.10
1.17
5.30
1.07
1.00
2.16
f1 (ppm)

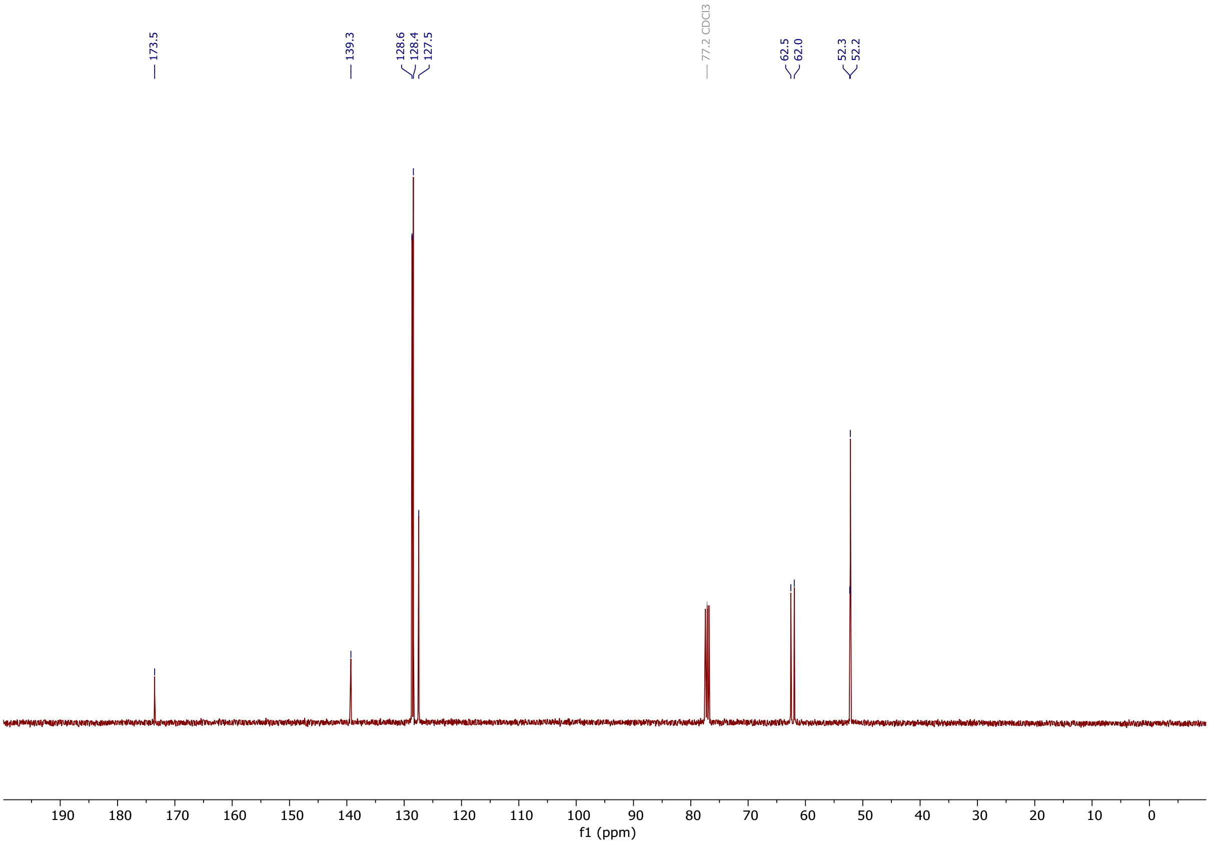

173.5
139.3
128.6
128.4
127.5
77.2 CDCl3
62.5
62.0
52.3
52.2
f1 (ppm)

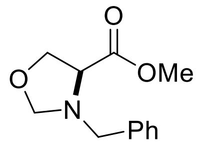

S9

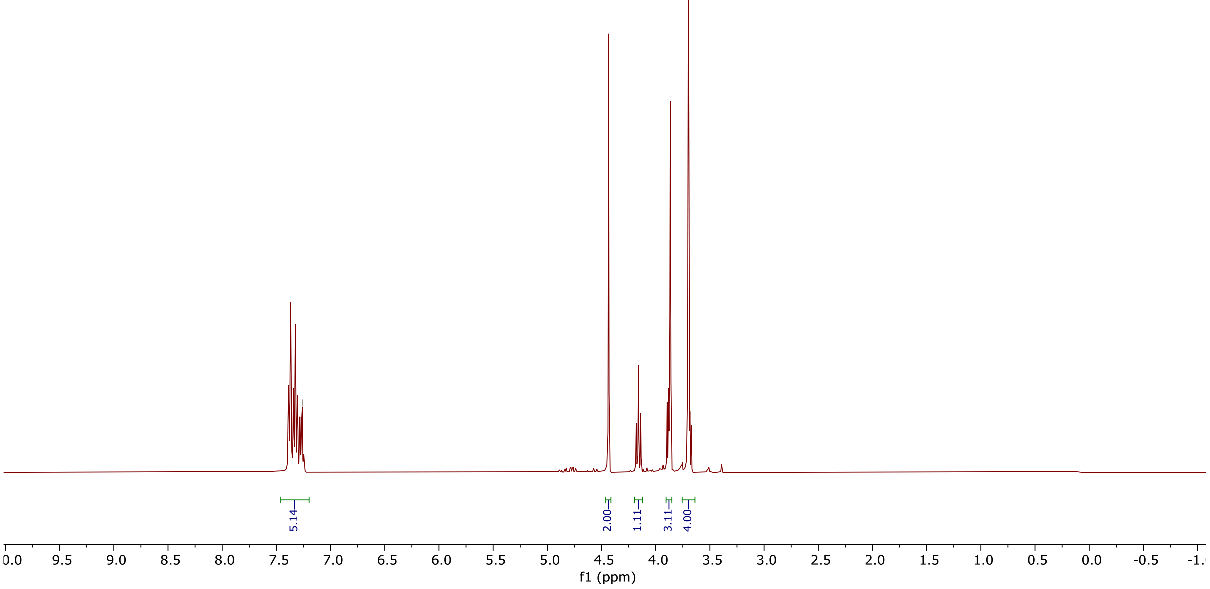
7.26 CDCl3
5.14
2.00
1.11
3.11
4.00
f1 (ppm)

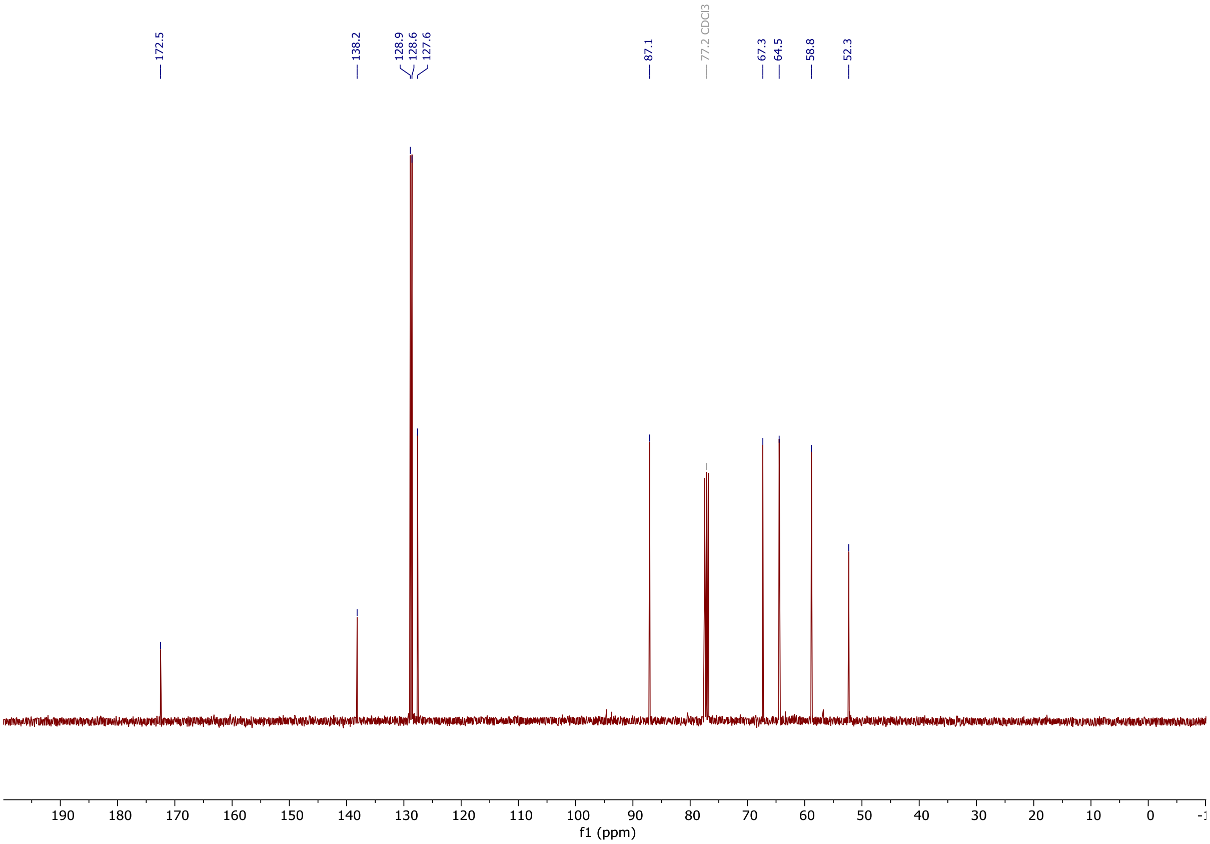
172.5
138.2
128.9
128.6
127.6
87.1
77.2 CDCl3
67.3
64.5
58.8
52.3
f1 (ppm)

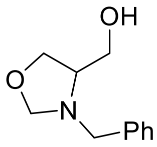
OH
O
N
Ph

1i

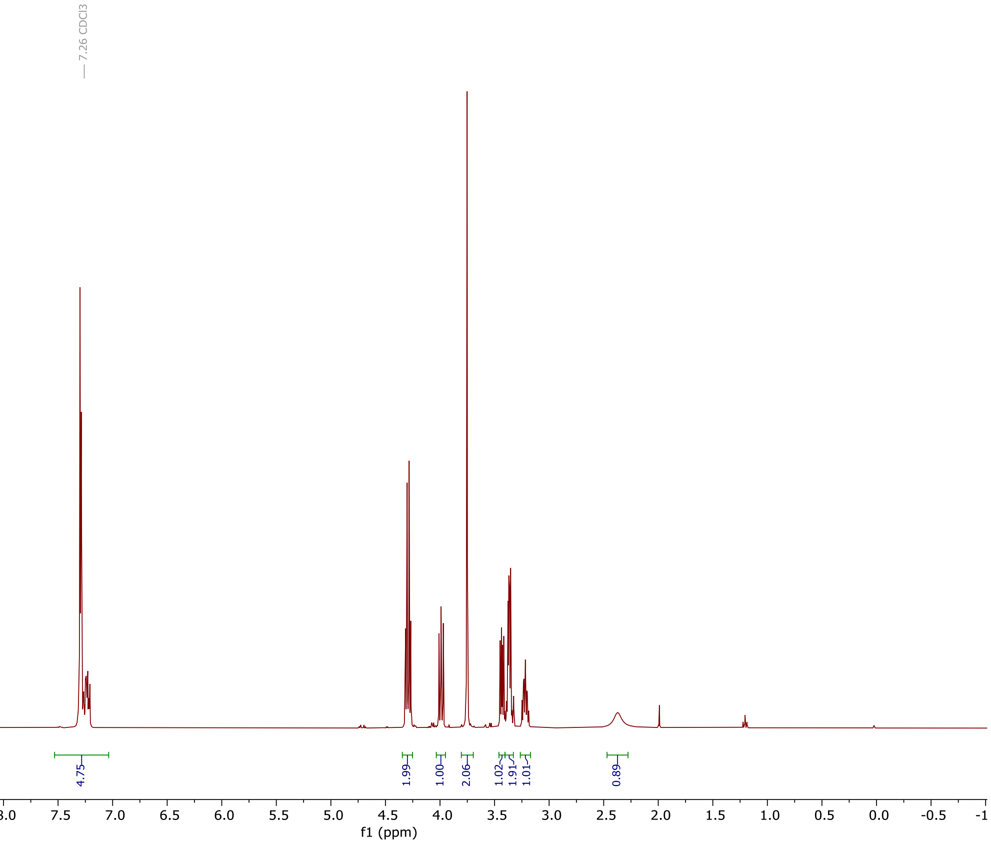
7.26 CDCl3
4.75
1.99
1.00
2.06
1.02
1.91
1.01
0.89
f1 (ppm)

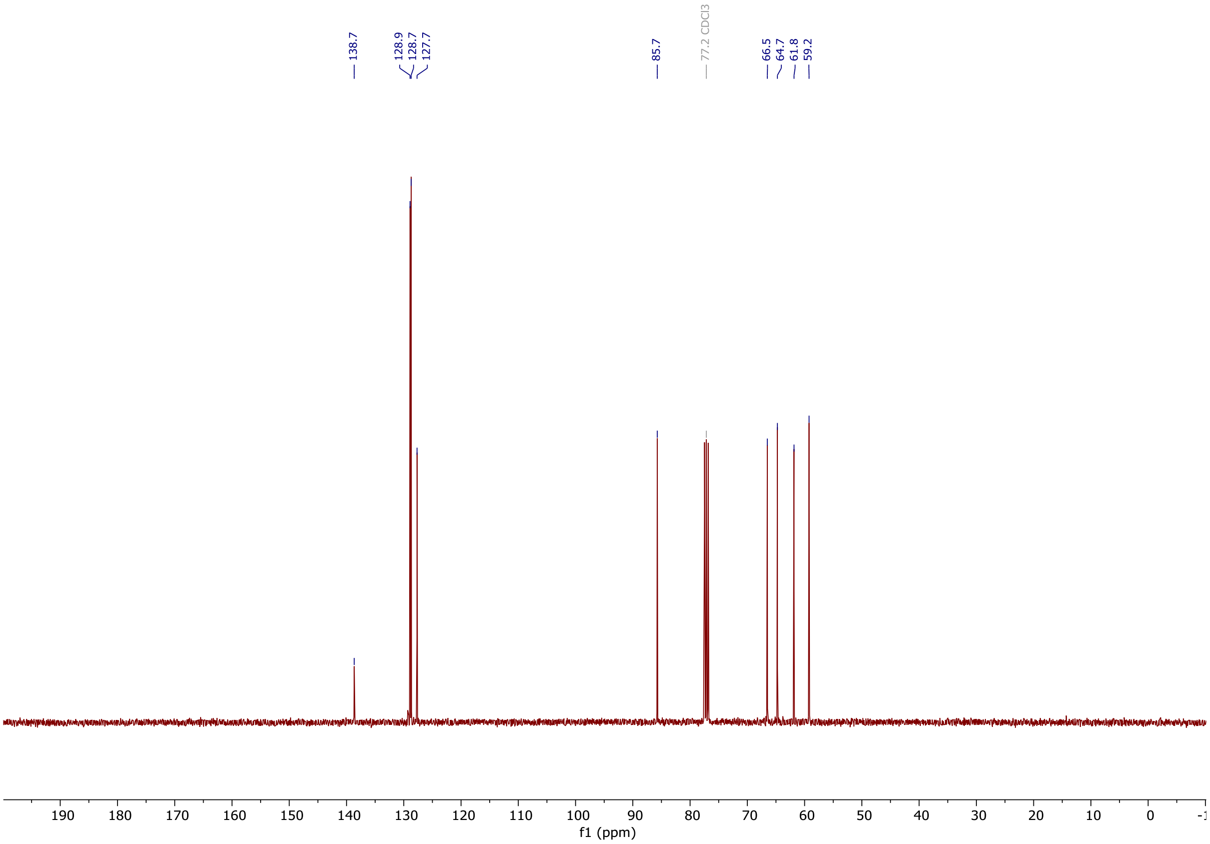
138.7
128.9
128.7
127.7
85.7
77.2 CDCl3
66.5
64.7
61.8
59.2
f1 (ppm)

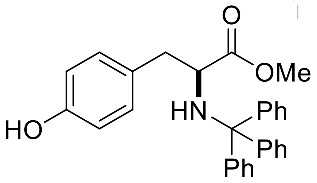
O
OMe
HN
Ph
Ph
Ph
HO

S10

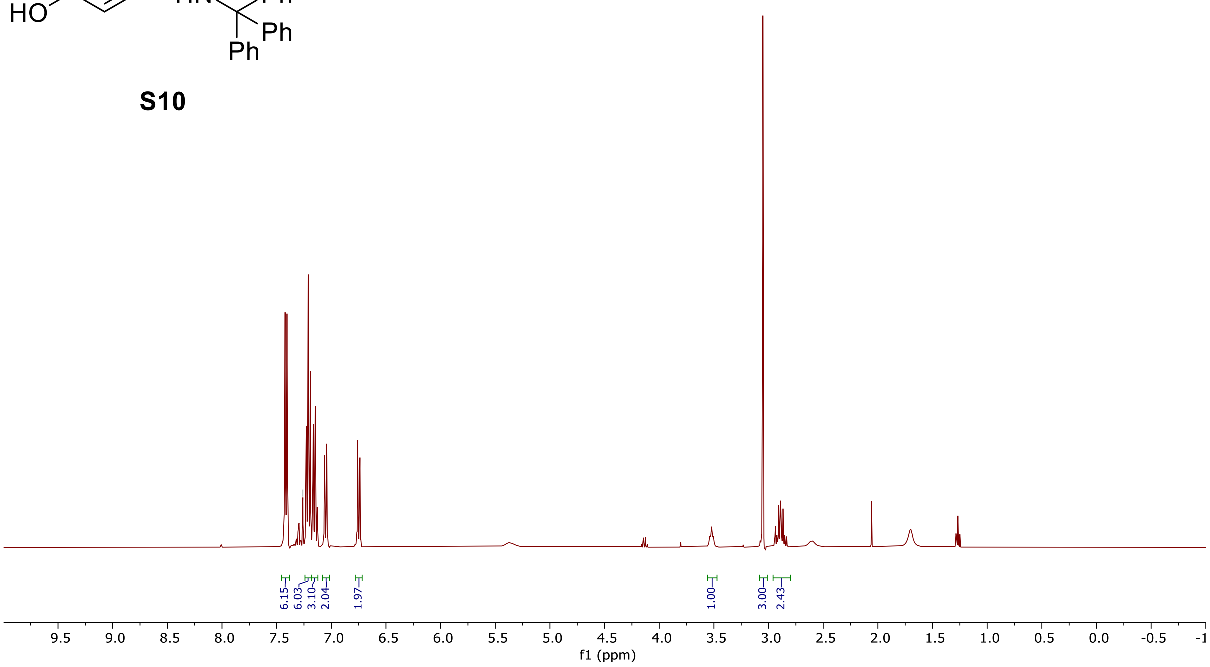
7.26 CDCl3
6.15
6.03
3.10
2.04
1.97
1.00
3.00
2.43
9.5 9.0 8.5 8.0 7.5 7.0 6.5 6.0 5.5 5.0 4.5 4.0 3.5 3.0 2.5 2.0 1.5 1.0 0.5 0.0 -0.5
f1 (ppm)

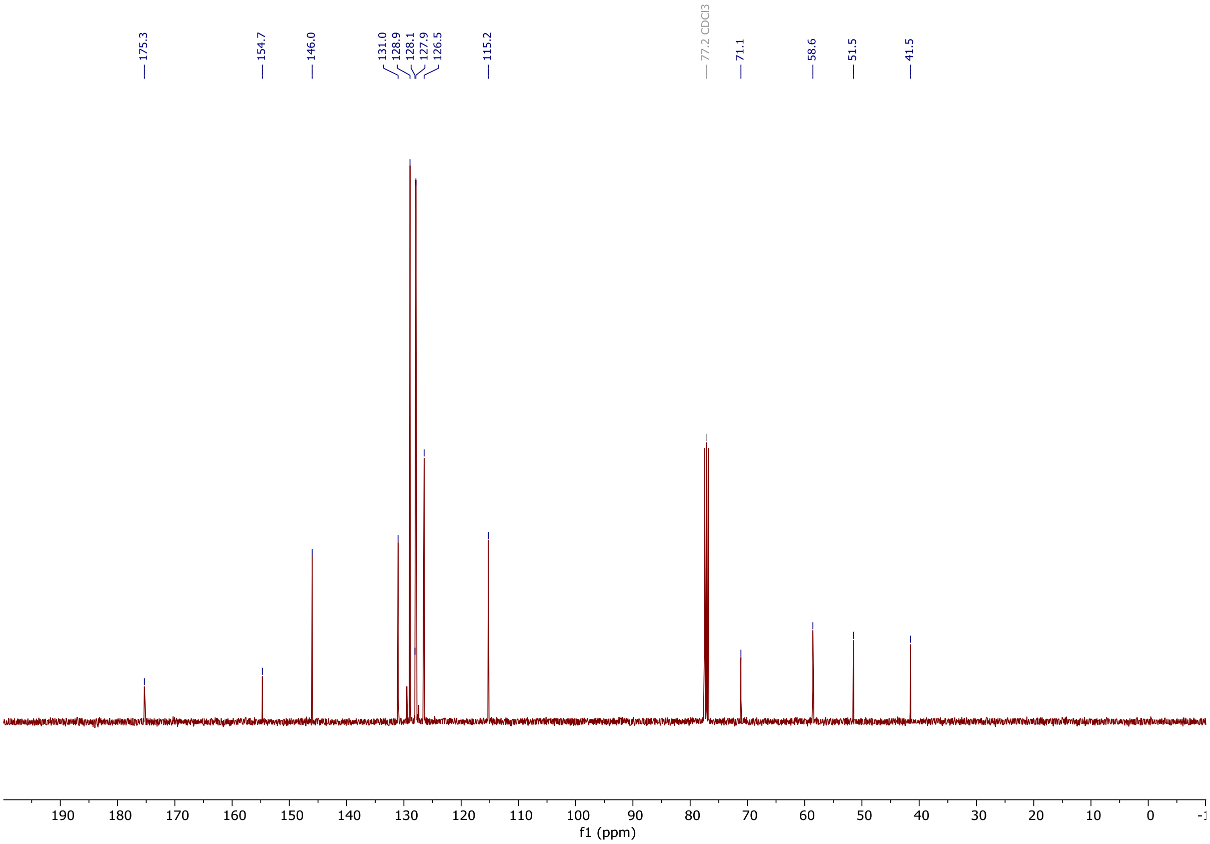
175.3
154.7
146.0
131.0
128.9
128.1
127.9
126.5
115.2
77.2 CDCl3
71.1
58.6
51.5
41.5
190 180 170 160 150 140 130 120 110 100 90 80 70 60 50 40 30 20 10 0
f1 (ppm)

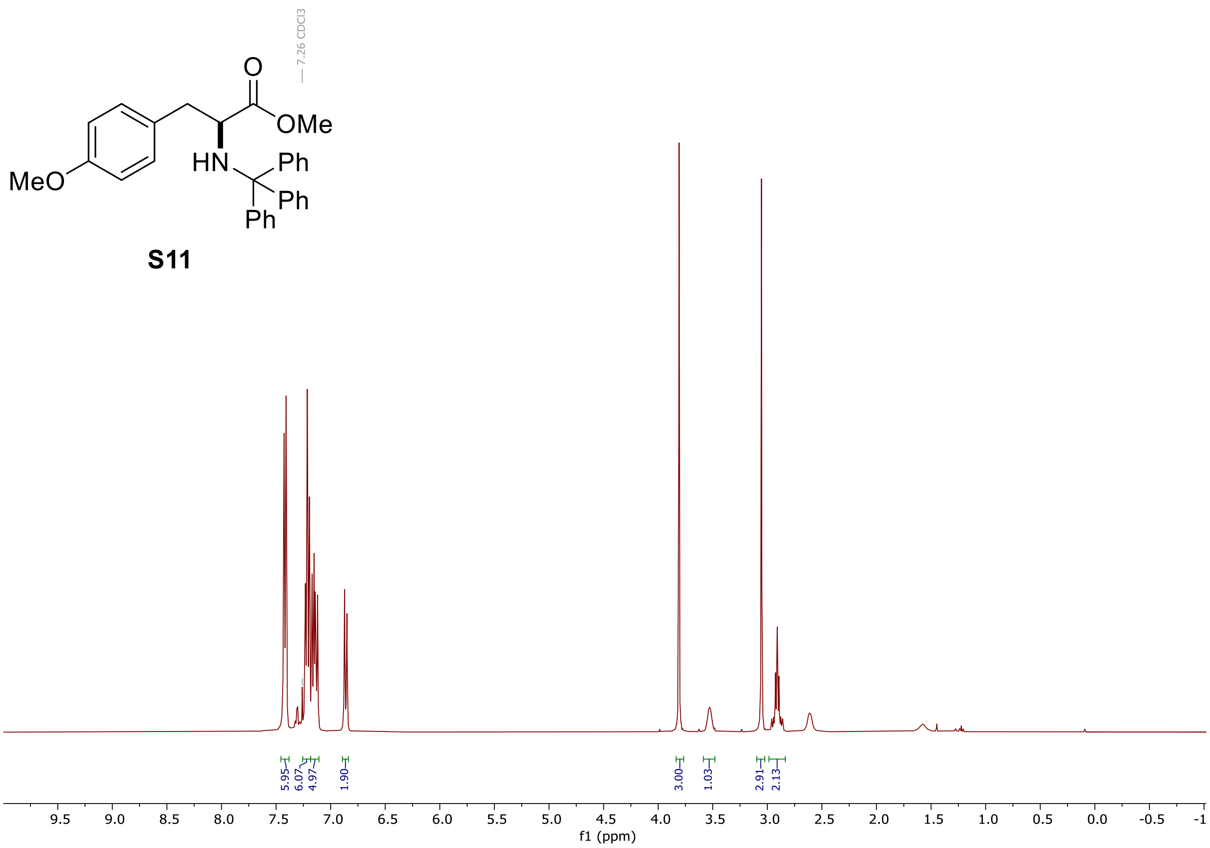
MeO
O
OMe
HN
Ph
Ph
Ph
S11
7.26 CDCl3
5.95
6.07
4.97
1.90
3.00
1.03
2.91
2.13
9.5
9.0
8.5
8.0
7.5
7.0
6.5
6.0
5.5
5.0
4.5
4.0
3.5
3.0
2.5
2.0
1.5
1.0
0.5
0.0
-0.5
f1 (ppm)

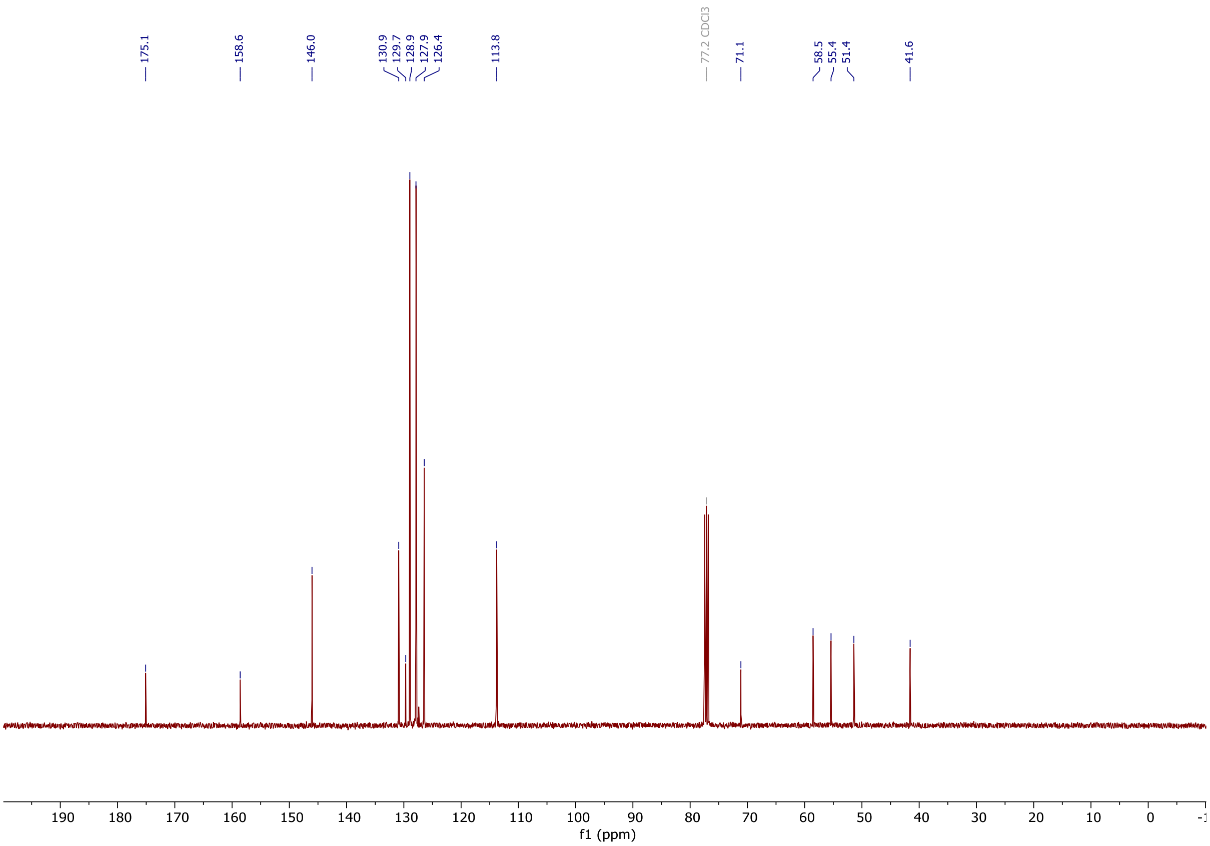
175.1
158.6
146.0
130.9
129.7
128.9
127.9
126.4
113.8
77.2 CDCl3
71.1
58.5
55.4
51.4
41.6
190
180
170
160
150
140
130
120
110
100
90
80
70
60
50
40
30
20
10
0
f1 (ppm)

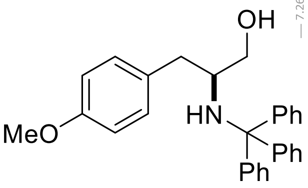

OH
HN
Ph
Ph
Ph
MeO

1j

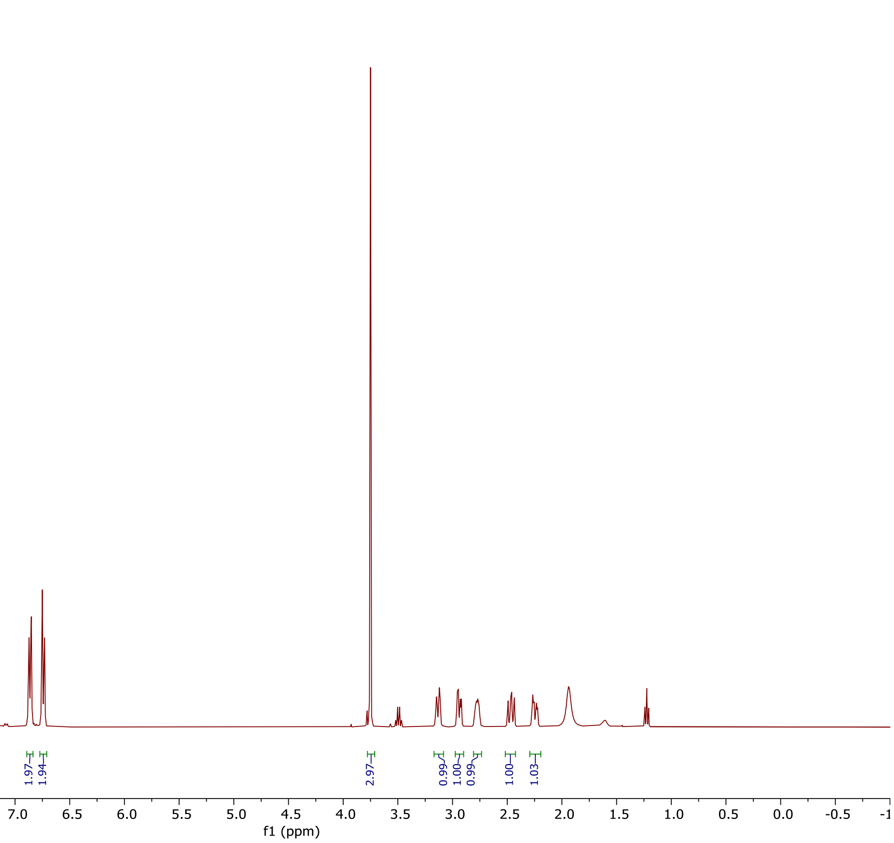

7.26 CDCl3
6.02
6.09
3.05
1.97
1.94
2.97
0.99
1.00
0.99
1.00
1.03
f1 (ppm)

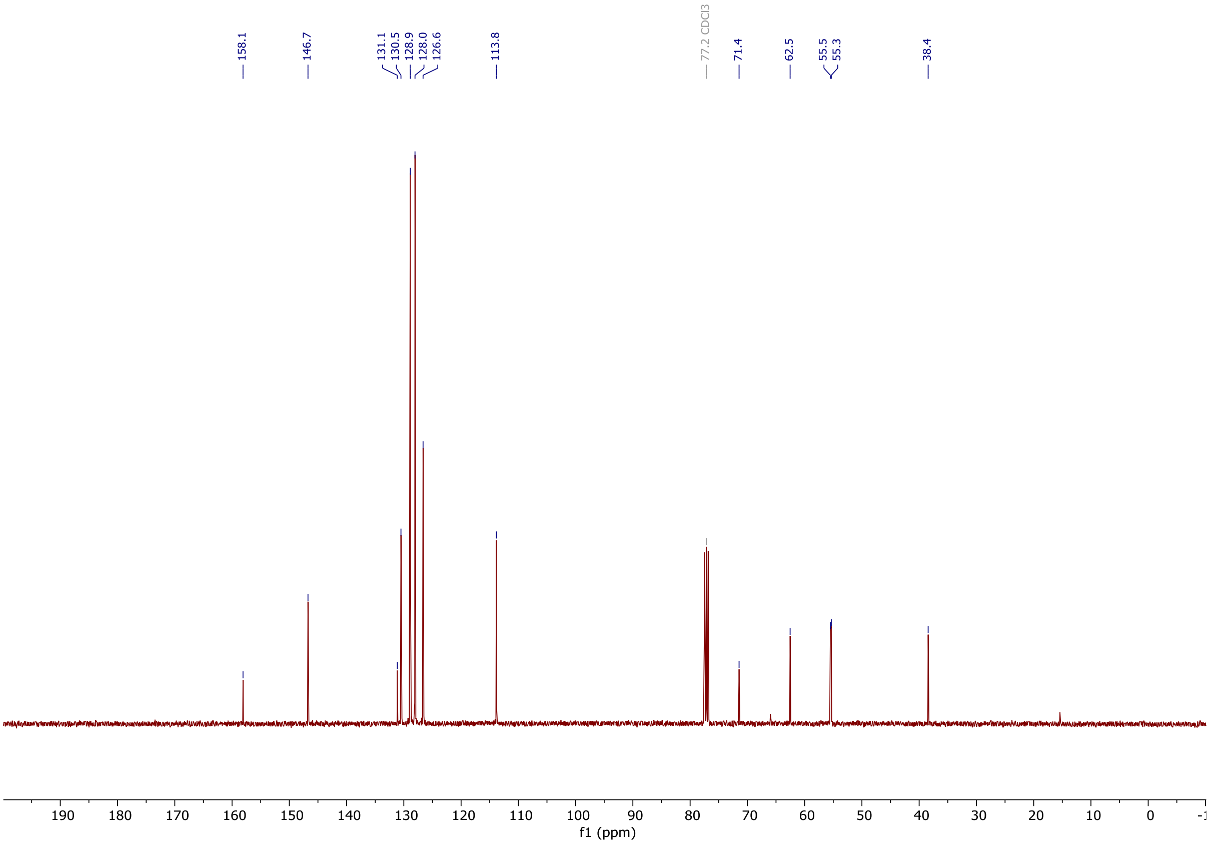

158.1
146.7
131.1
130.5
128.9
128.0
126.6
113.8
77.2 CDCl3
71.4
62.5
55.5
55.3
38.4
f1 (ppm)

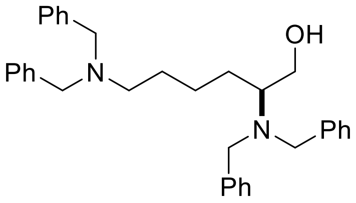
Ph
Ph
N
OH
N
Ph
Ph

1k

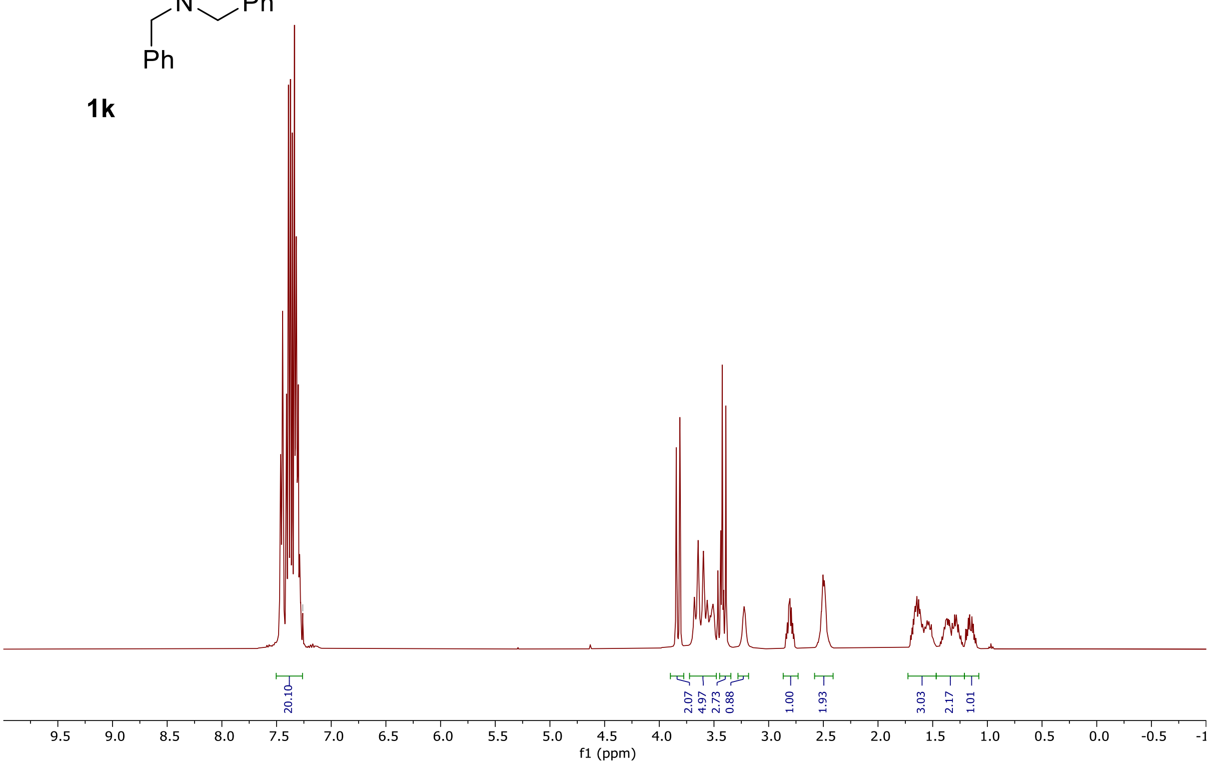
7.26 CDCl3
20.10
2.07
4.97
2.73
0.88
1.00
1.93
3.03
2.17
1.01
f1 (ppm)

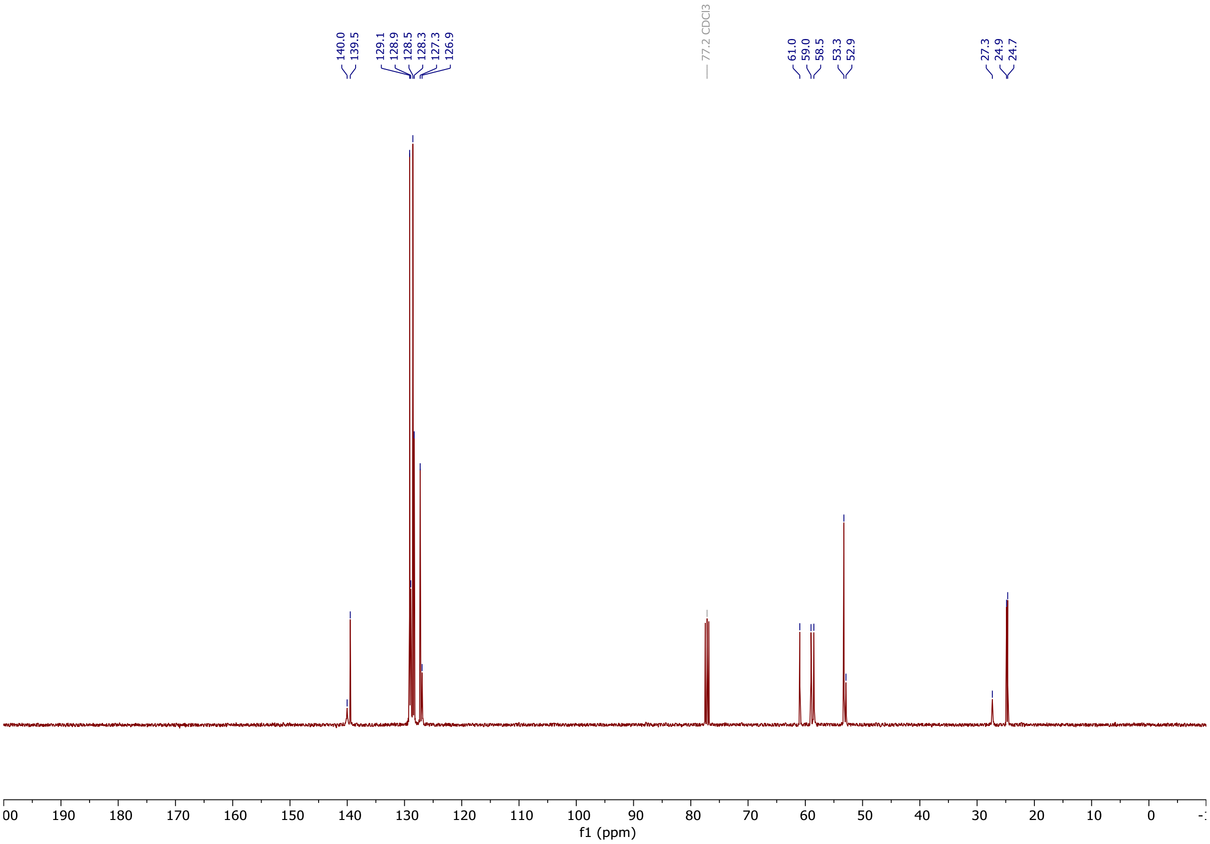
140.0
139.5
129.1
128.9
128.5
128.3
127.3
126.9
77.2 CDCl3
61.0
59.0
58.5
53.3
52.9
27.3
24.9
24.7
f1 (ppm)

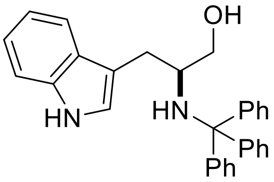
OH
HN
HN
Ph
Ph
Ph

S12

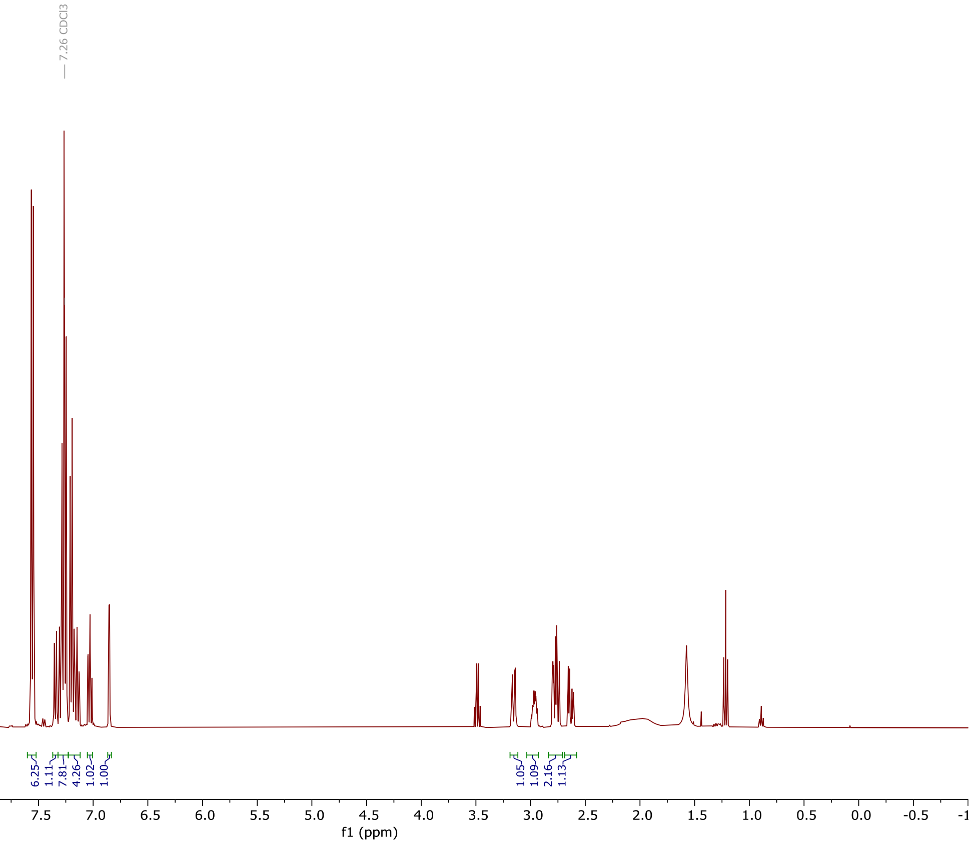
7.26 CDCl3
0.94
6.25
1.11
7.81
4.26
1.02
1.00
1.05
1.09
2.16
1.13
f1 (ppm)

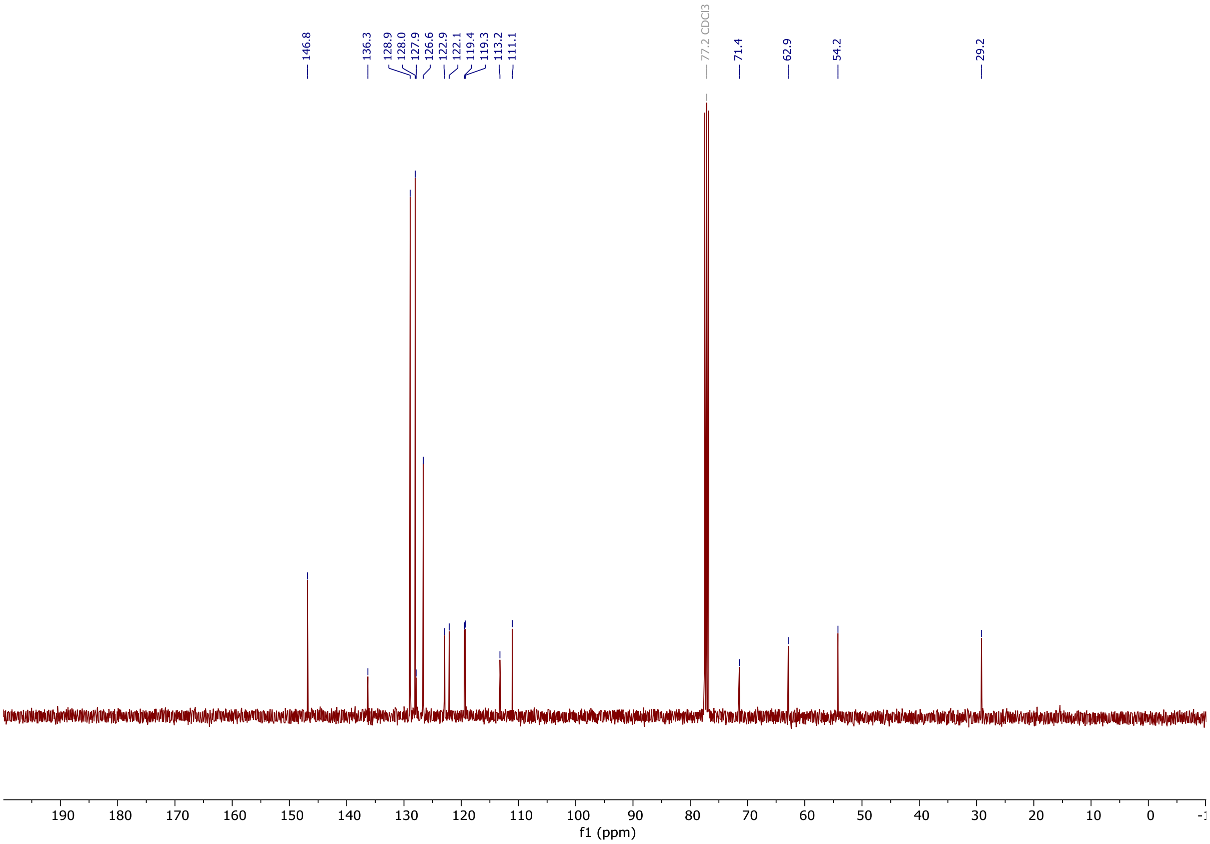
146.8
136.3
128.9
128.0
127.9
126.6
122.9
122.1
119.4
119.3
113.2
111.1
77.2 CDCl3
71.4
62.9
54.2
29.2
f1 (ppm)

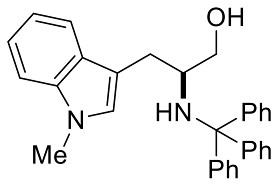

1l

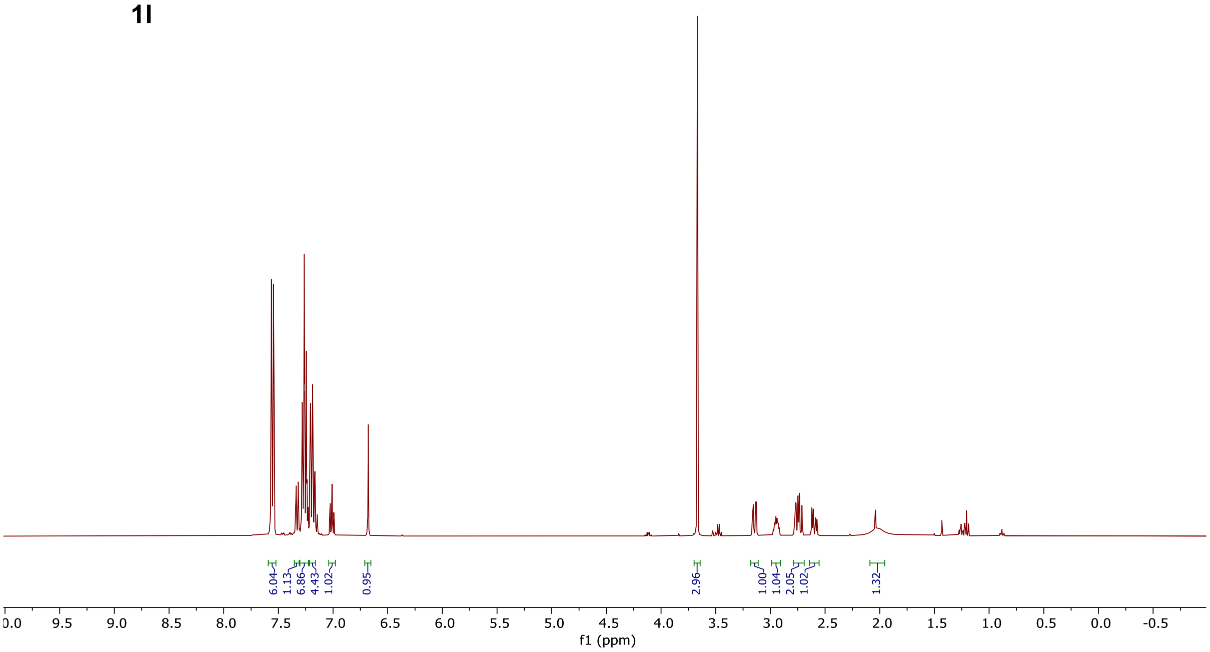
7.26 CDCl3
6.04
1.13
6.86
4.43
1.02
0.95
2.96
1.00
1.04
2.05
1.02
1.32
f1 (ppm)

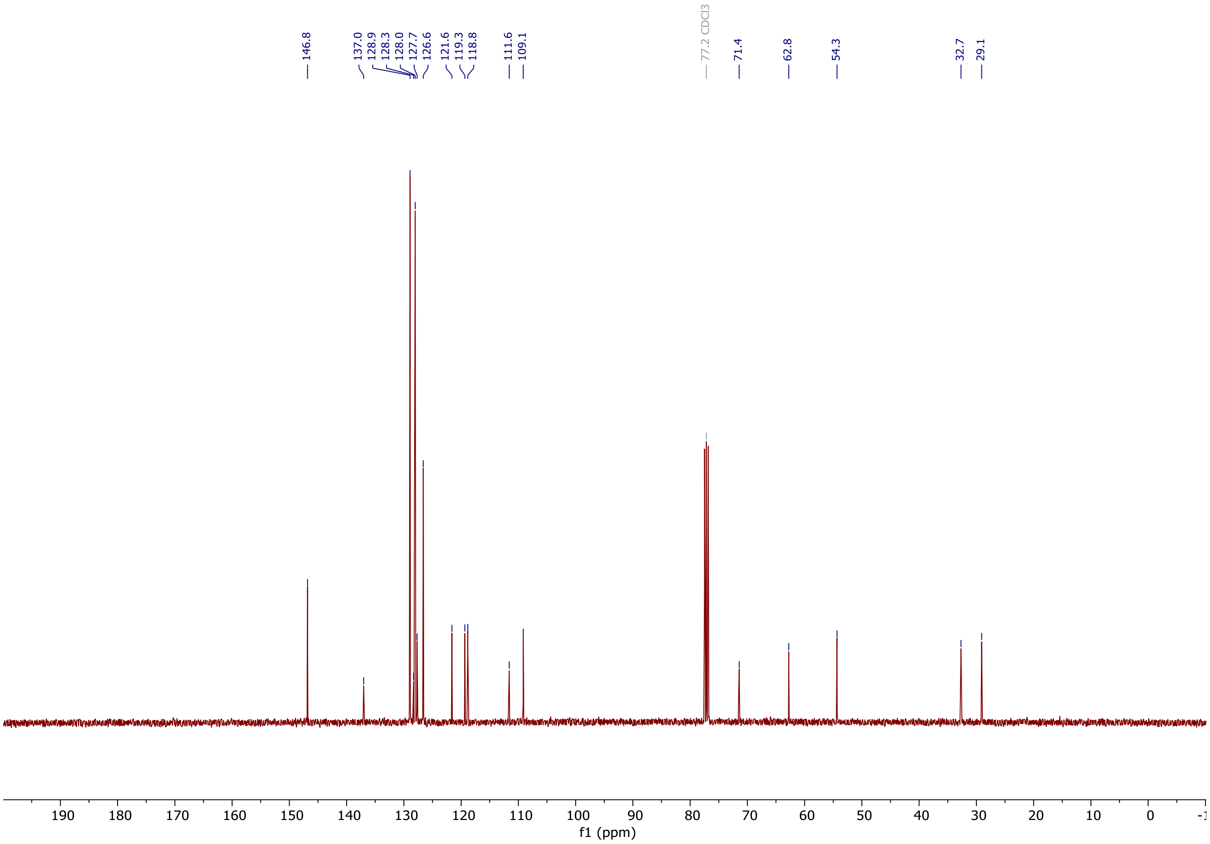
146.8
137.0
128.9
128.3
128.0
127.7
126.6
121.6
119.3
118.8
111.6
109.1
77.2 CDCl3
71.4
62.8
54.3
32.7
29.1
f1 (ppm)

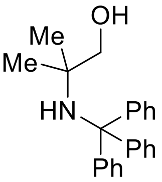
OH
Me
Me
HN
Ph
Ph
Ph

1m

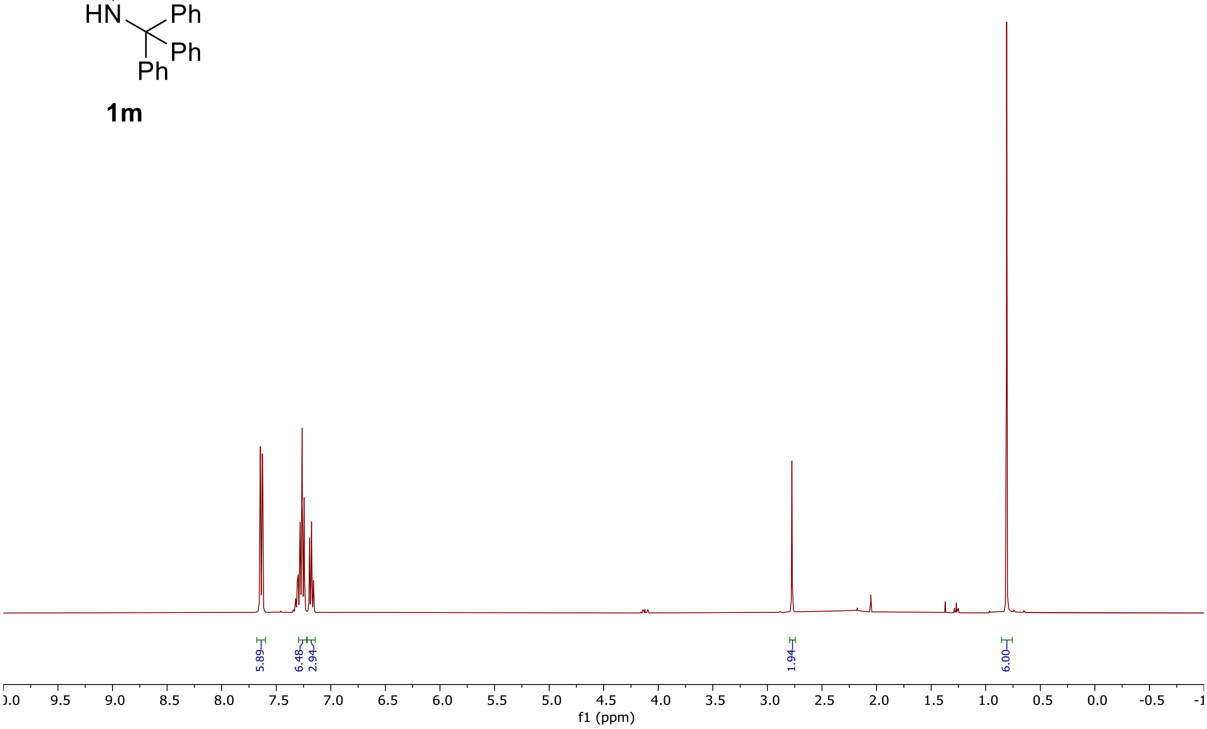
7.26 CDCl3
5.89
6.48
2.94
1.94
6.00
f1 (ppm)

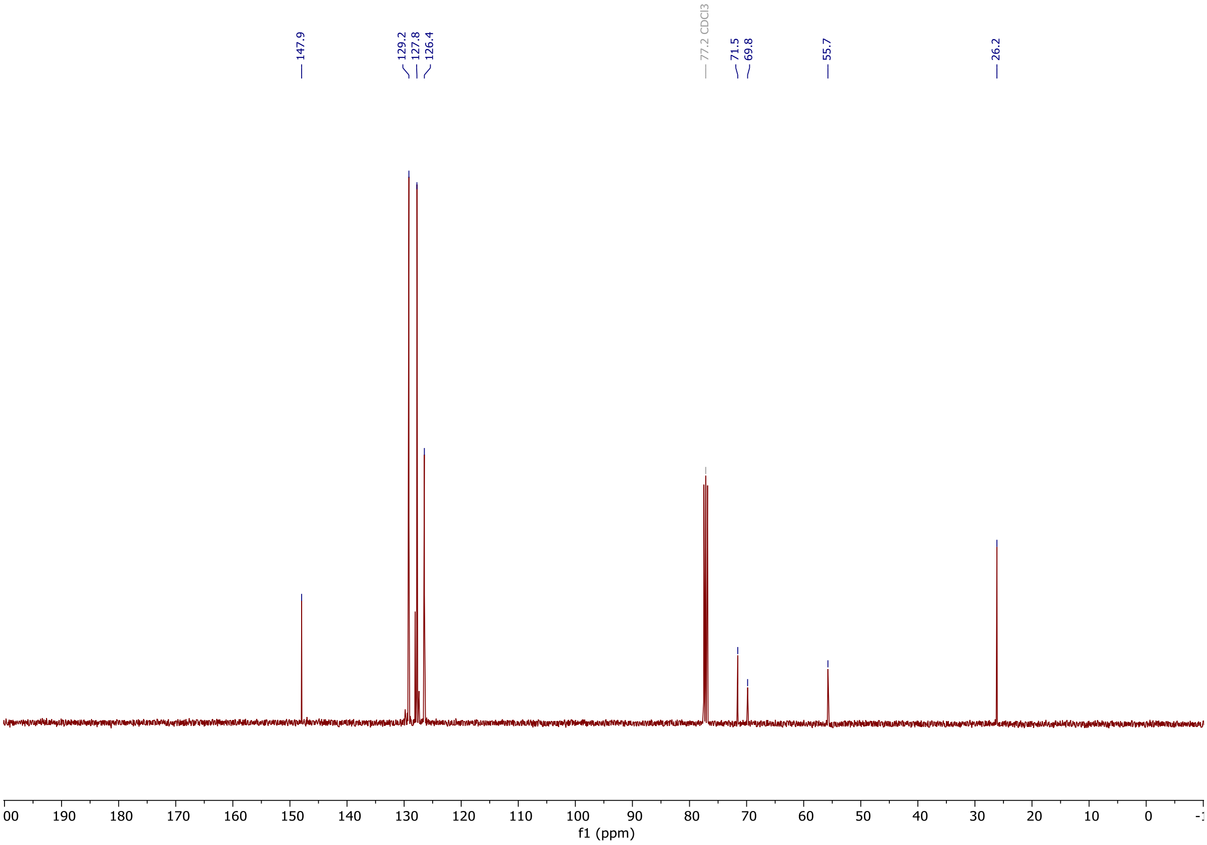
147.9
129.2
127.8
126.4
77.2 CDCl3
71.5
69.8
55.7
26.2
f1 (ppm)

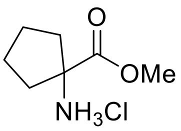
O
OMe
NH3Cl

S13

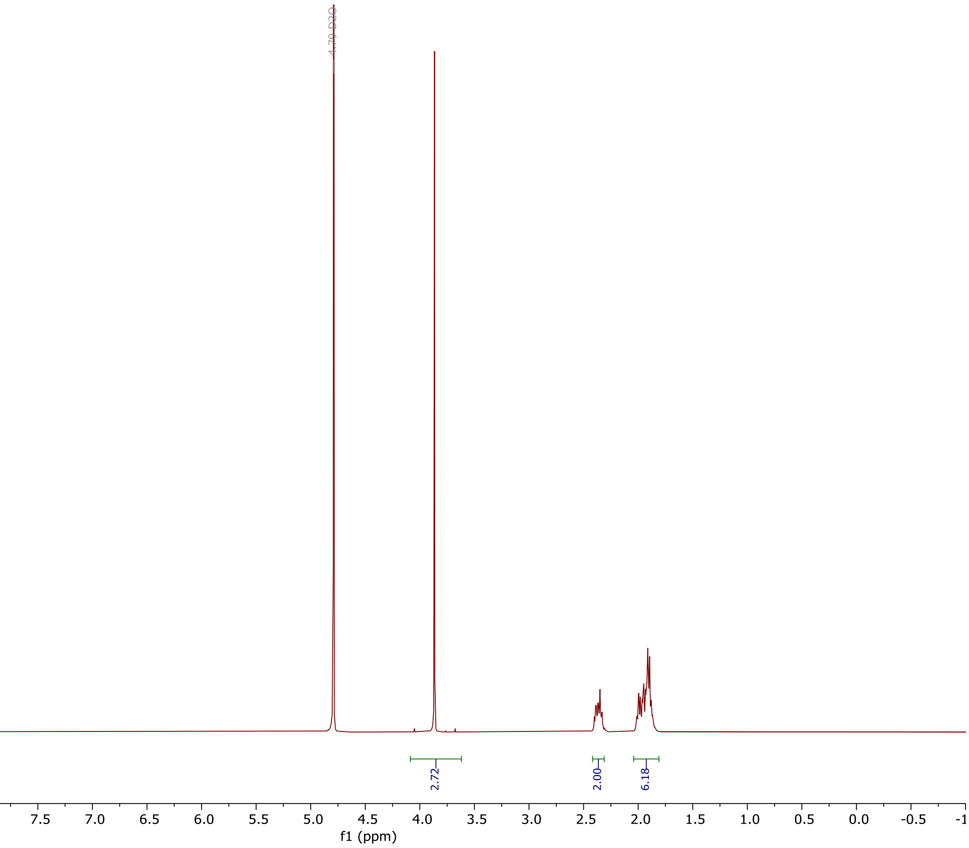
2.72
2.00
6.18
9.5 9.0 8.5 8.0 7.5 7.0 6.5 6.0 5.5 5.0 4.5 4.0 3.5 3.0 2.5 2.0 1.5 1.0 0.5 0.0 -0.5
f1 (ppm)

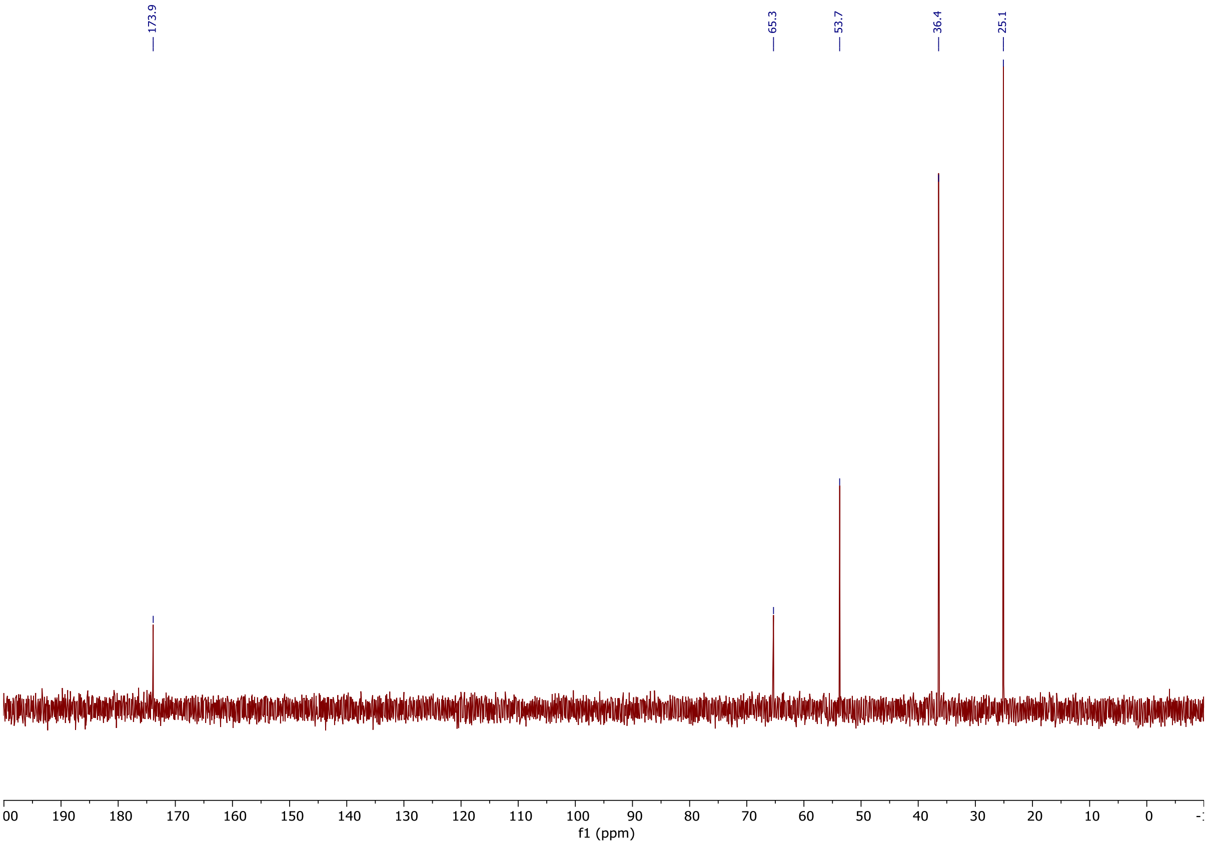
173.9
65.3
53.7
36.4
25.1
190 180 170 160 150 140 130 120 110 100 90 80 70 60 50 40 30 20 10 0
f1 (ppm)

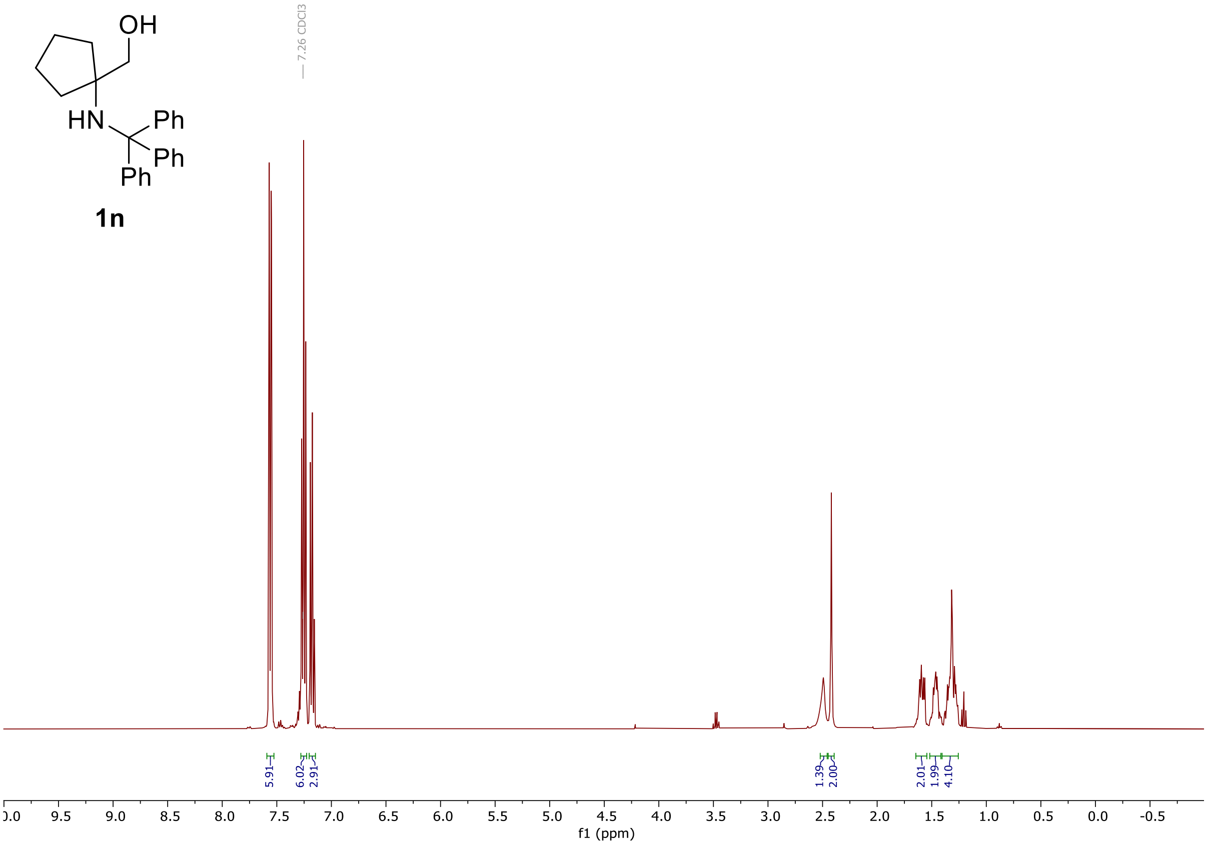
OH
HN
Ph
Ph
Ph
1n
7.26 CDCl3
5.91
6.02
2.91
1.39
2.00
2.01
1.99
4.10
f1 (ppm)

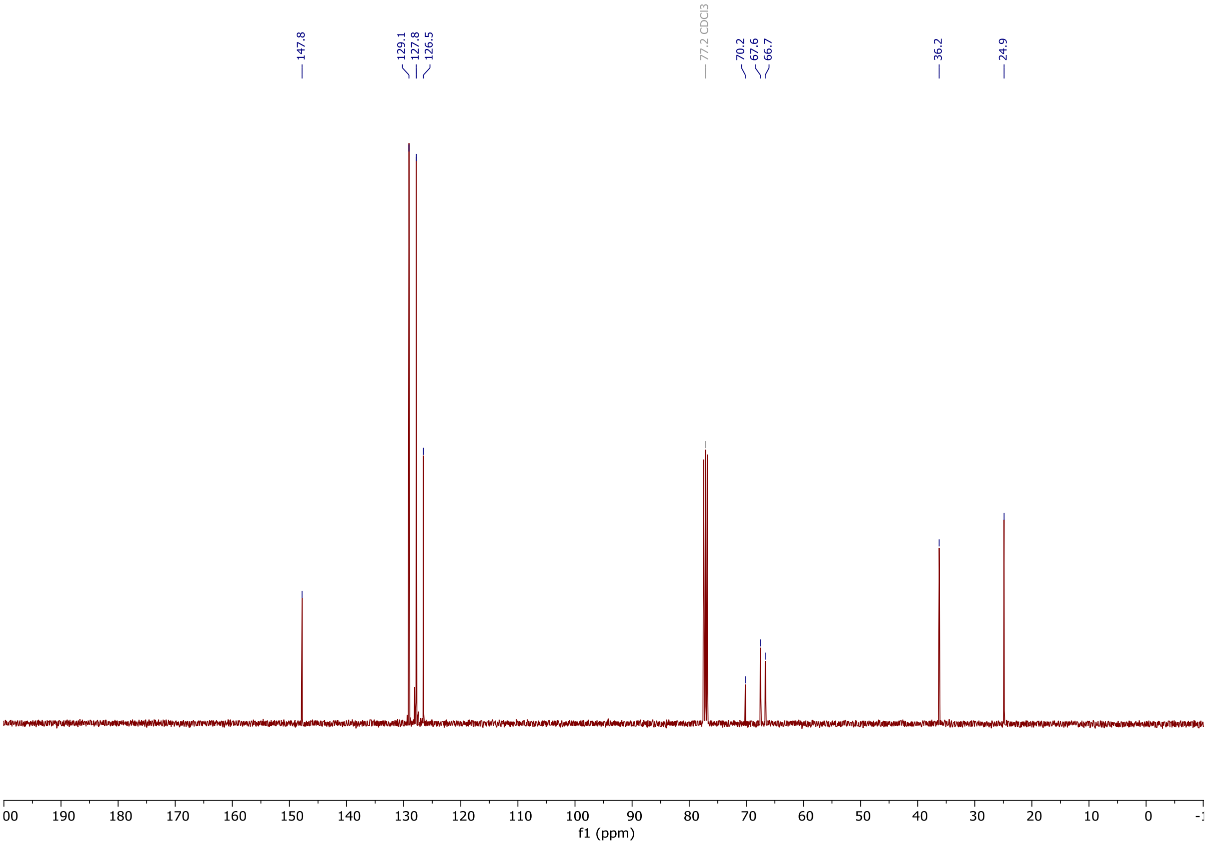
147.8
129.1
127.8
126.5
77.2 CDCl3
70.2
67.6
66.7
36.2
24.9
f1 (ppm)

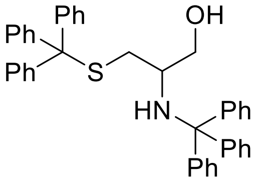

Ph
Ph
Ph
S
OH
HN
Ph
Ph
Ph

8a

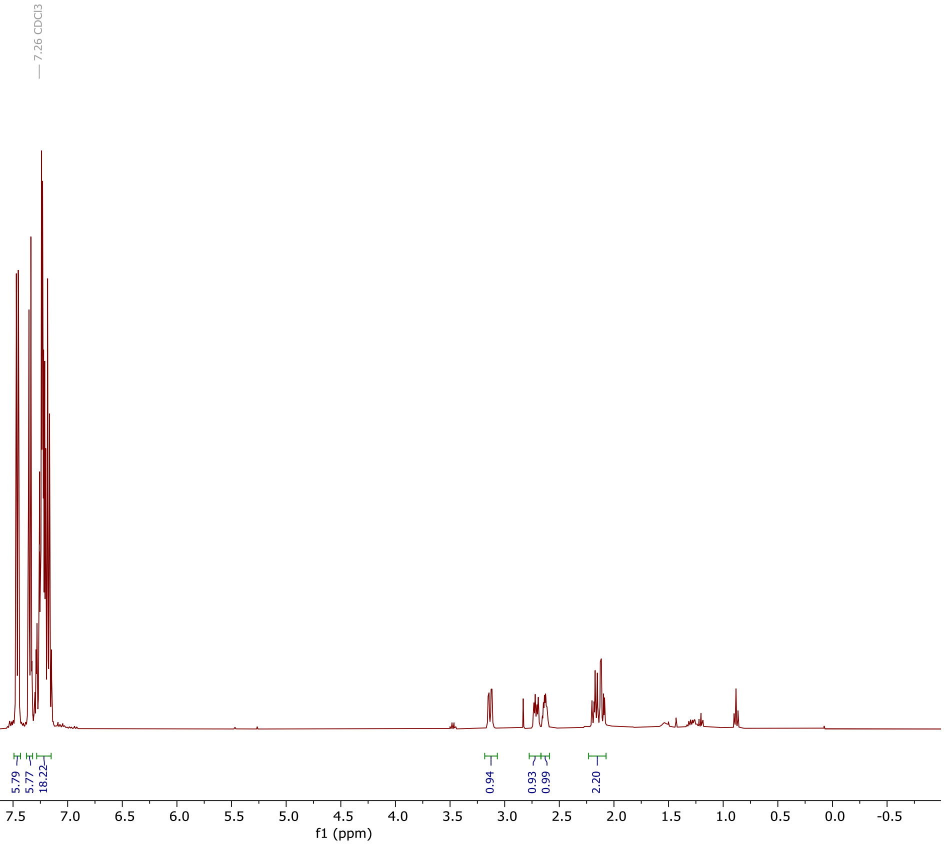

7.26 CDCl3
5.79
5.77
18.22
0.94
0.93
0.99
2.20
9.5 9.0 8.5 8.0 7.5 7.0 6.5 6.0 5.5 5.0 4.5 4.0 3.5 3.0 2.5 2.0 1.5 1.0 0.5 0.0 -0.5
f1 (ppm)

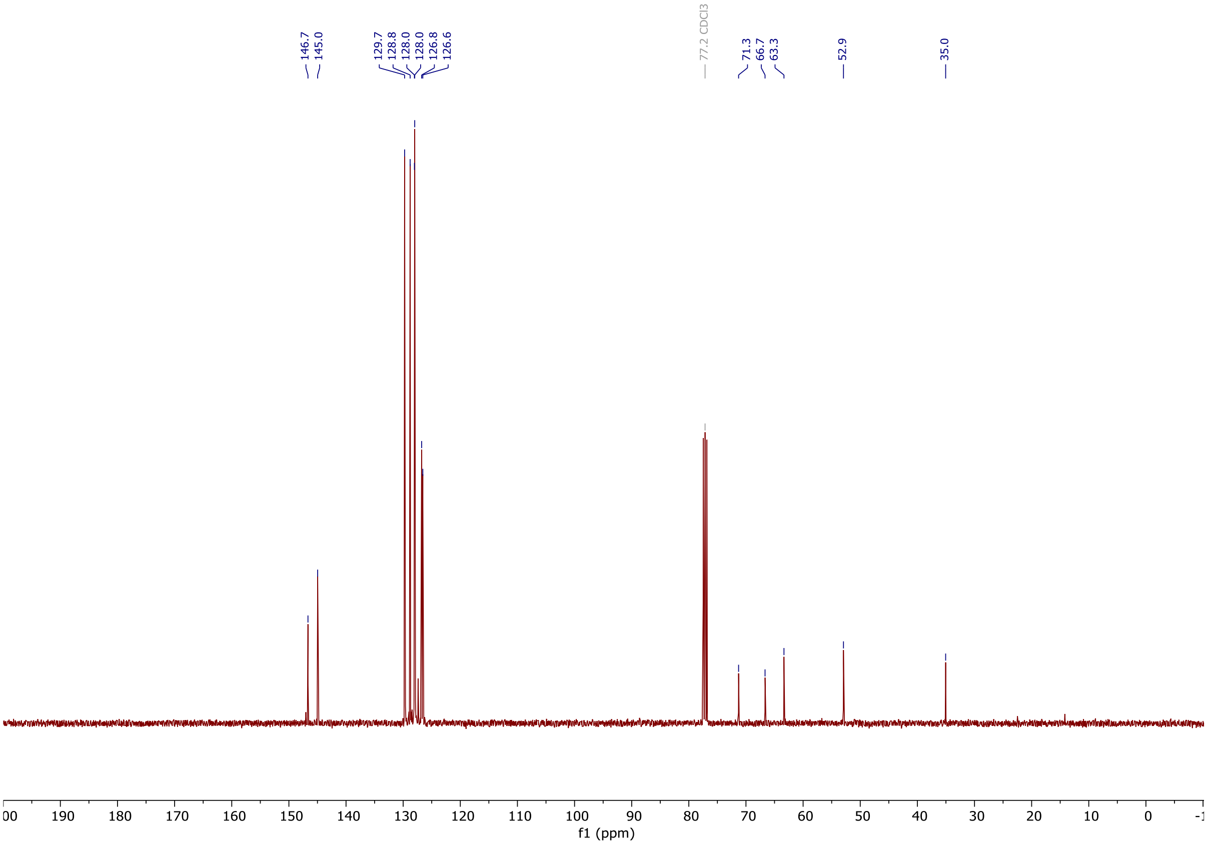

146.7
145.0
129.7
128.8
128.0
128.0
126.8
126.6
77.2 CDCl3
71.3
66.7
63.3
52.9
35.0
190 180 170 160 150 140 130 120 110 100 90 80 70 60 50 40 30 20 10 0
f1 (ppm)

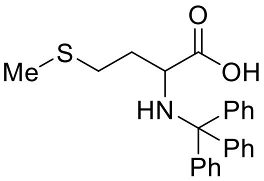
O
Me
S
OH
HN
Ph
Ph
Ph

S14

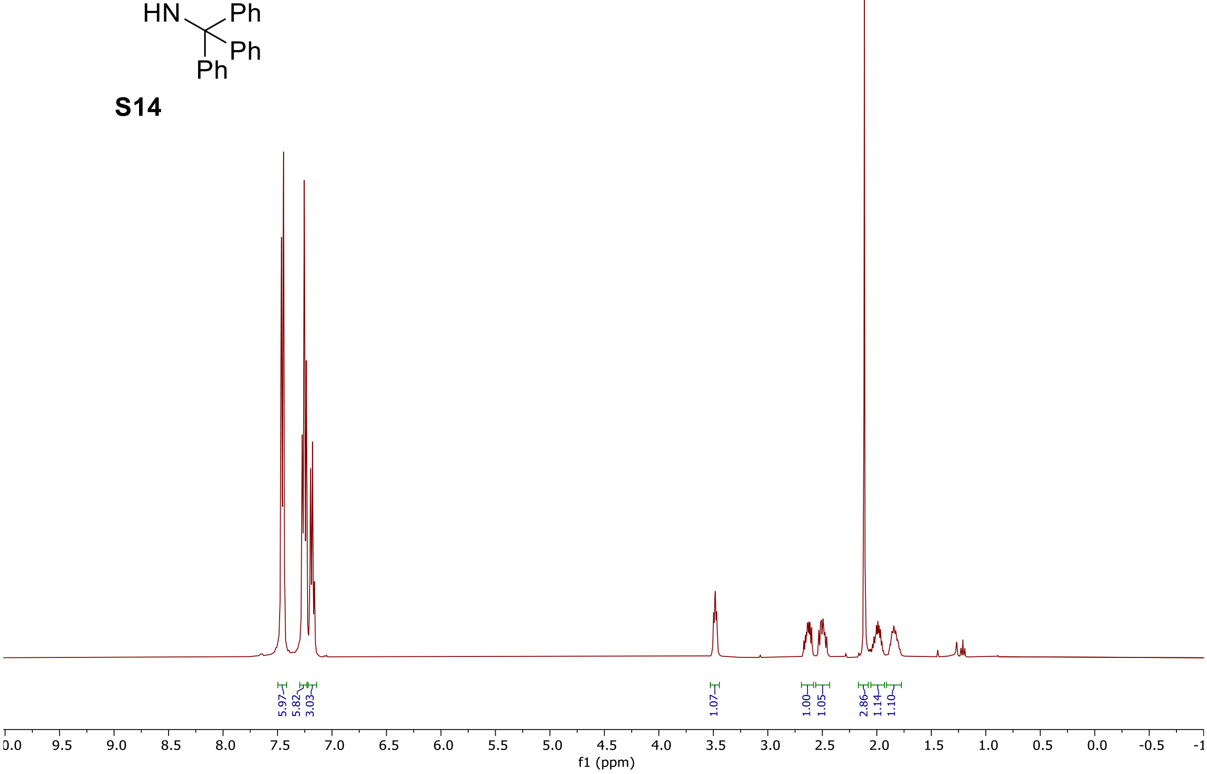
5.97
5.82
3.03
1.07
1.00
1.05
2.86
1.14
1.10
0.0
9.5
9.0
8.5
8.0
7.5
7.0
6.5
6.0
5.5
5.0
4.5
4.0
3.5
3.0
2.5
2.0
1.5
1.0
0.5
0.0
-0.5
f1 (ppm)

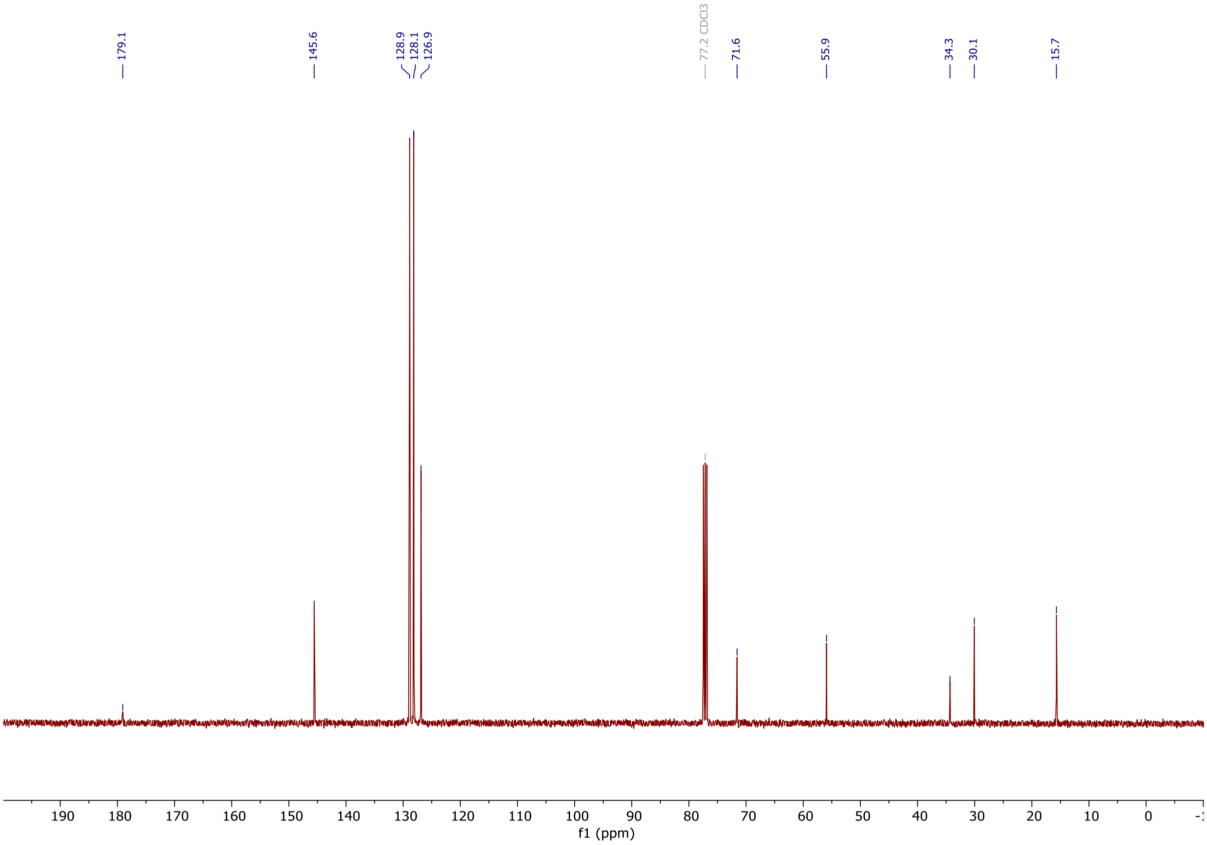
179.1
145.6
128.9
128.1
126.9
77.2 CDCl3
71.6
55.9
34.3
30.1
15.7
190
180
170
160
150
140
130
120
110
100
90
80
70
60
50
40
30
20
10
0
f1 (ppm)

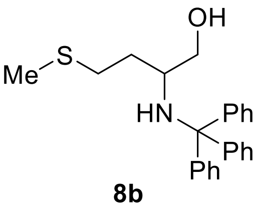
OH
Me
S
HN
Ph
Ph
Ph

8b

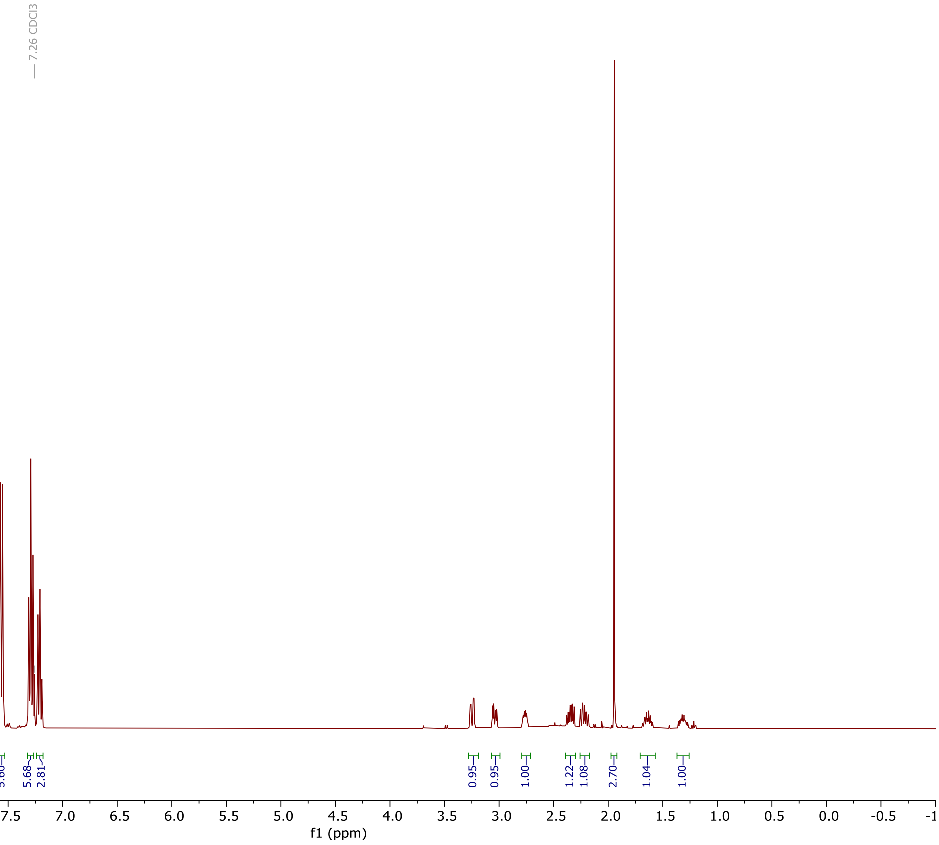
7.26 CDCl3
5.60
5.68
2.81
0.95
0.95
1.00
1.22
1.08
2.70
1.04
1.00
f1 (ppm)

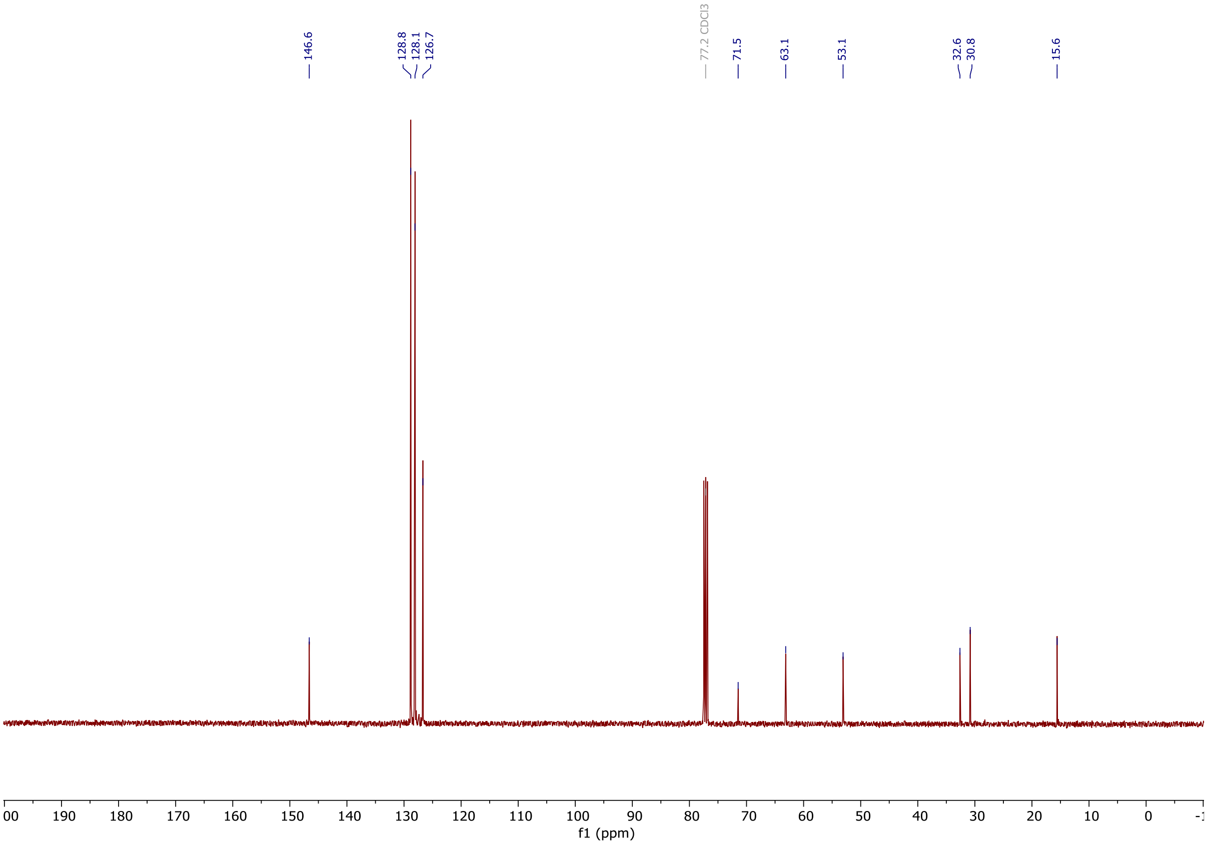
146.6
128.8
128.1
126.7
77.2 CDCl3
71.5
63.1
53.1
32.6
30.8
15.6
f1 (ppm)

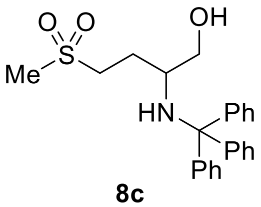

8c

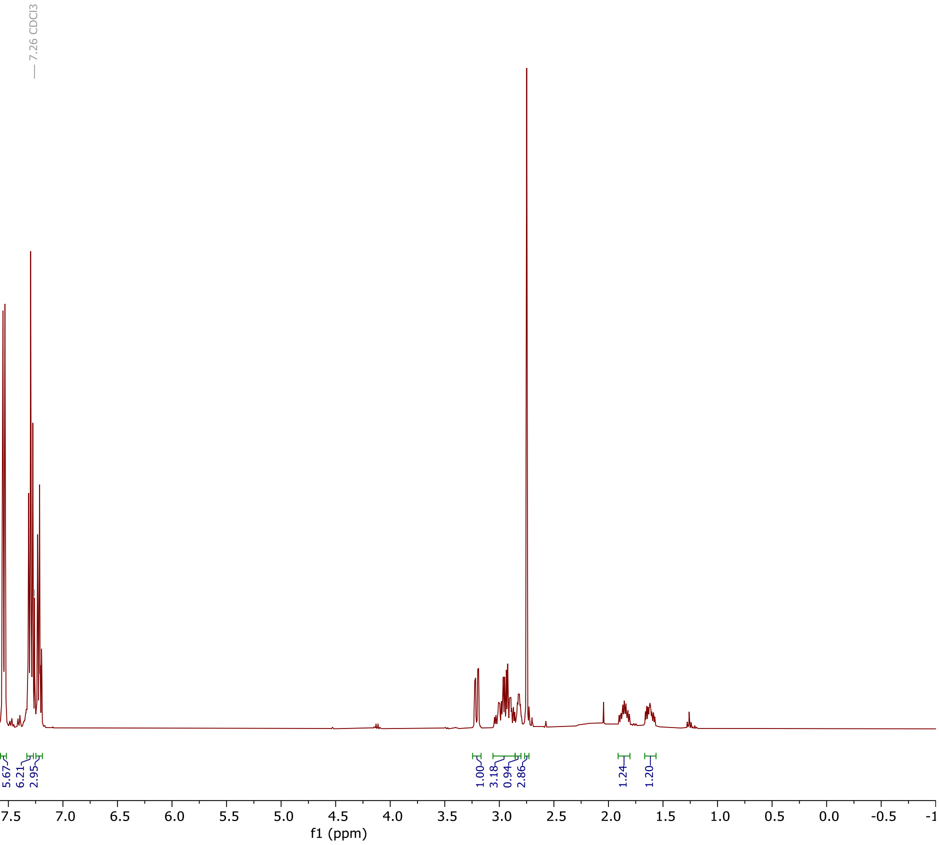

7.26 CDCl3
5.67
6.21
2.95
1.00
3.18
0.94
2.86
1.24
1.20
9.5
9.0
8.5
8.0
7.5
7.0
6.5
6.0
5.5
5.0
4.5
4.0
3.5
3.0
2.5
2.0
1.5
1.0
0.5
0.0
-0.5
f1 (ppm)

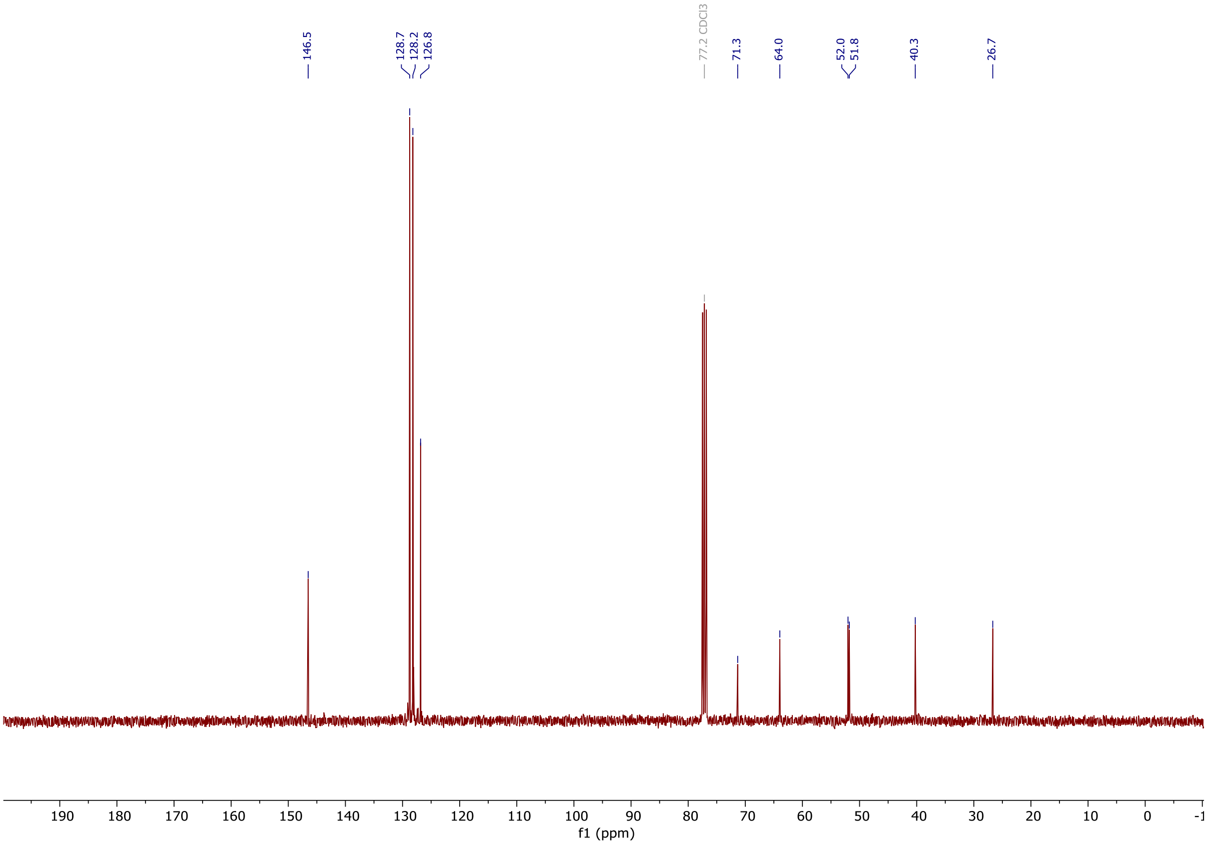

146.5
128.7
128.2
126.8
77.2 CDCl3
71.3
64.0
52.0
51.8
40.3
26.7
190
180
170
160
150
140
130
120
110
100
90
80
70
60
50
40
30
20
10
0
f1 (ppm)

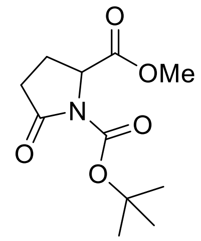
O
OMe
N
O
O
O

S15

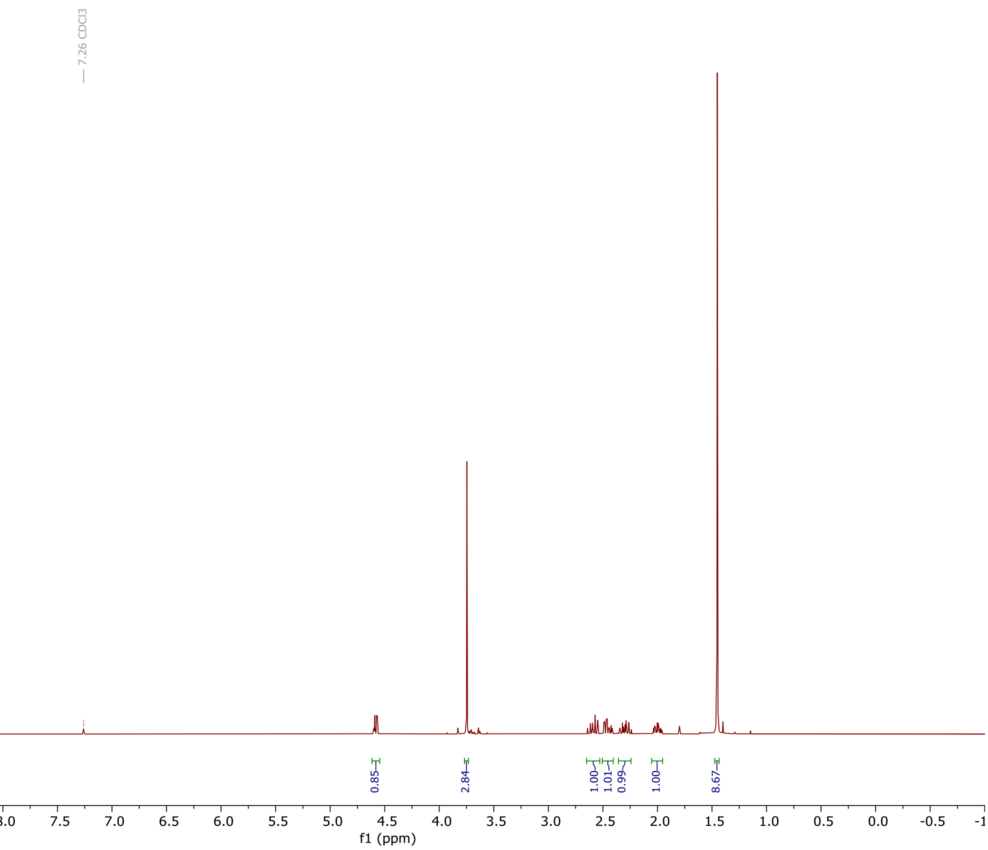
7.26 CDCl3
0.85
2.84
1.00
1.01
0.99
1.00
8.67
f1 (ppm)

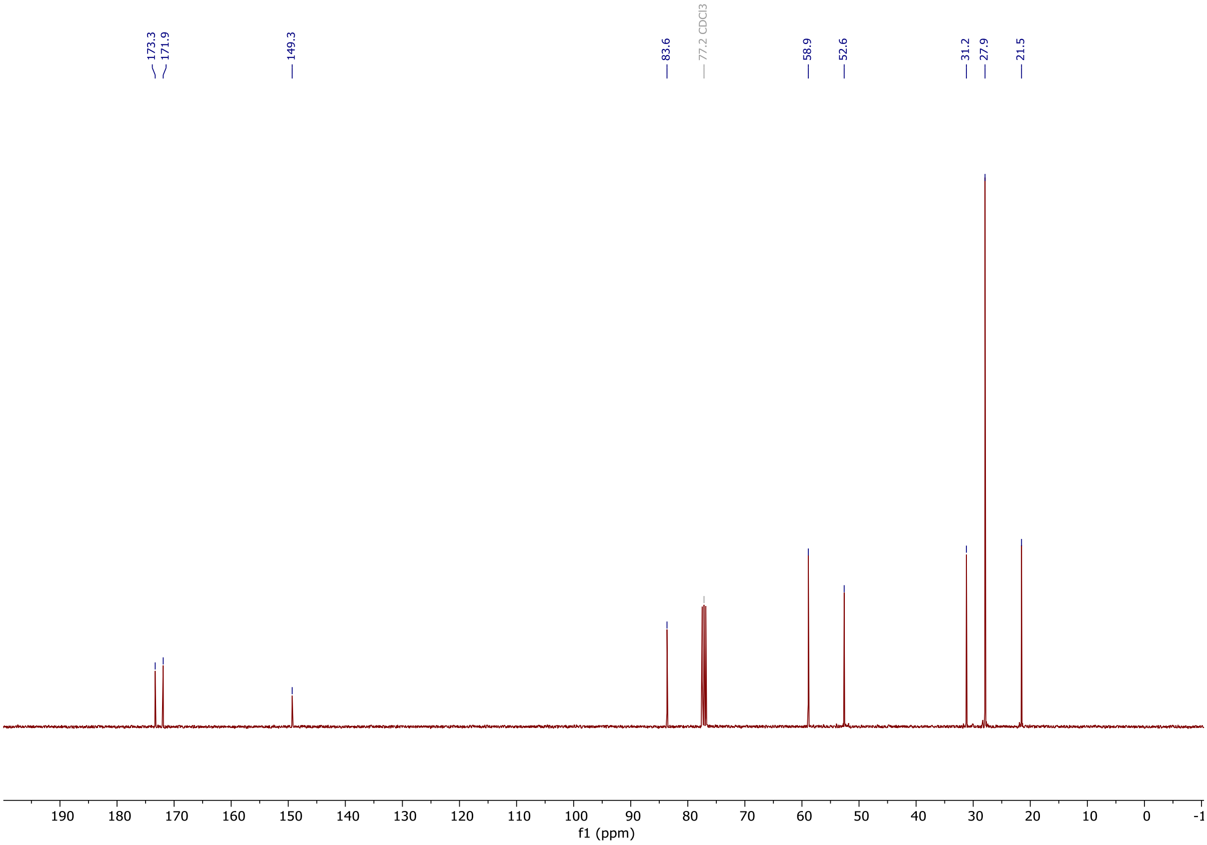
173.3
171.9
149.3
83.6
77.2 CDCl3
58.9
52.6
31.2
27.9
21.5
f1 (ppm)

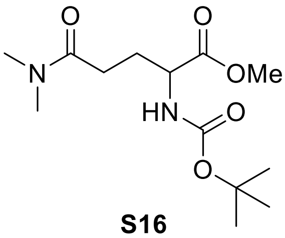
O
O
N
OMe
HN
O
O

S16

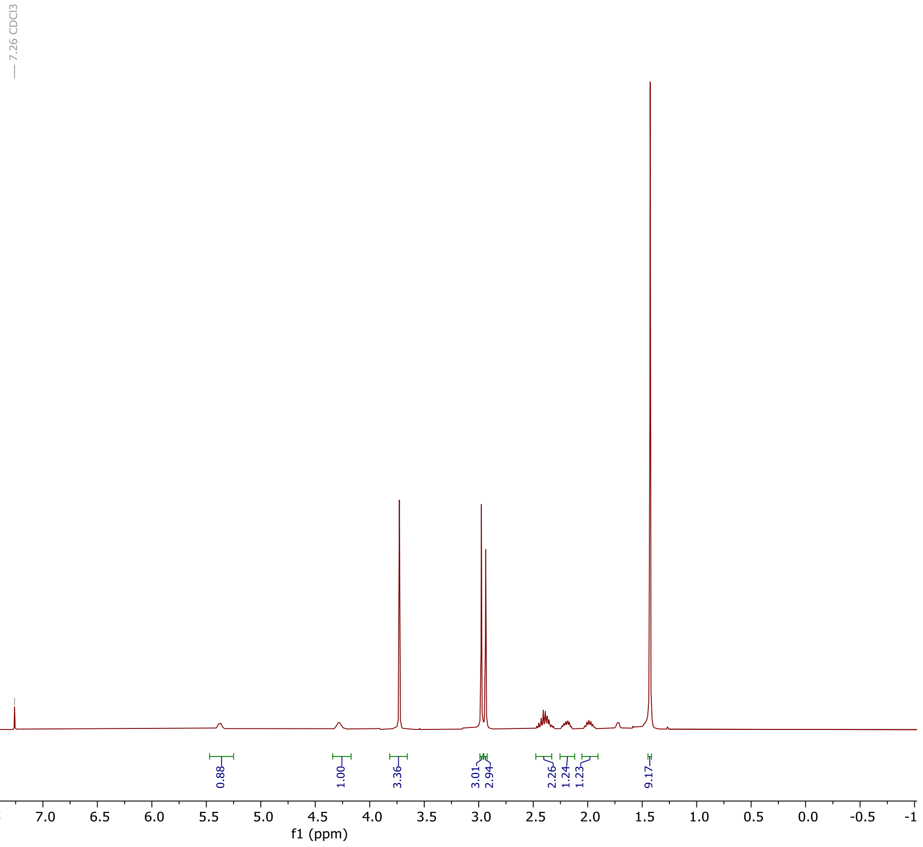
7.26 CDCl3
0.88
1.00
3.36
3.01
2.94
2.26
1.24
1.23
9.17
9.5 9.0 8.5 8.0 7.5 7.0 6.5 6.0 5.5 5.0 4.5 4.0 3.5 3.0 2.5 2.0 1.5 1.0 0.5 0.0 -0.5 -1
f1 (ppm)

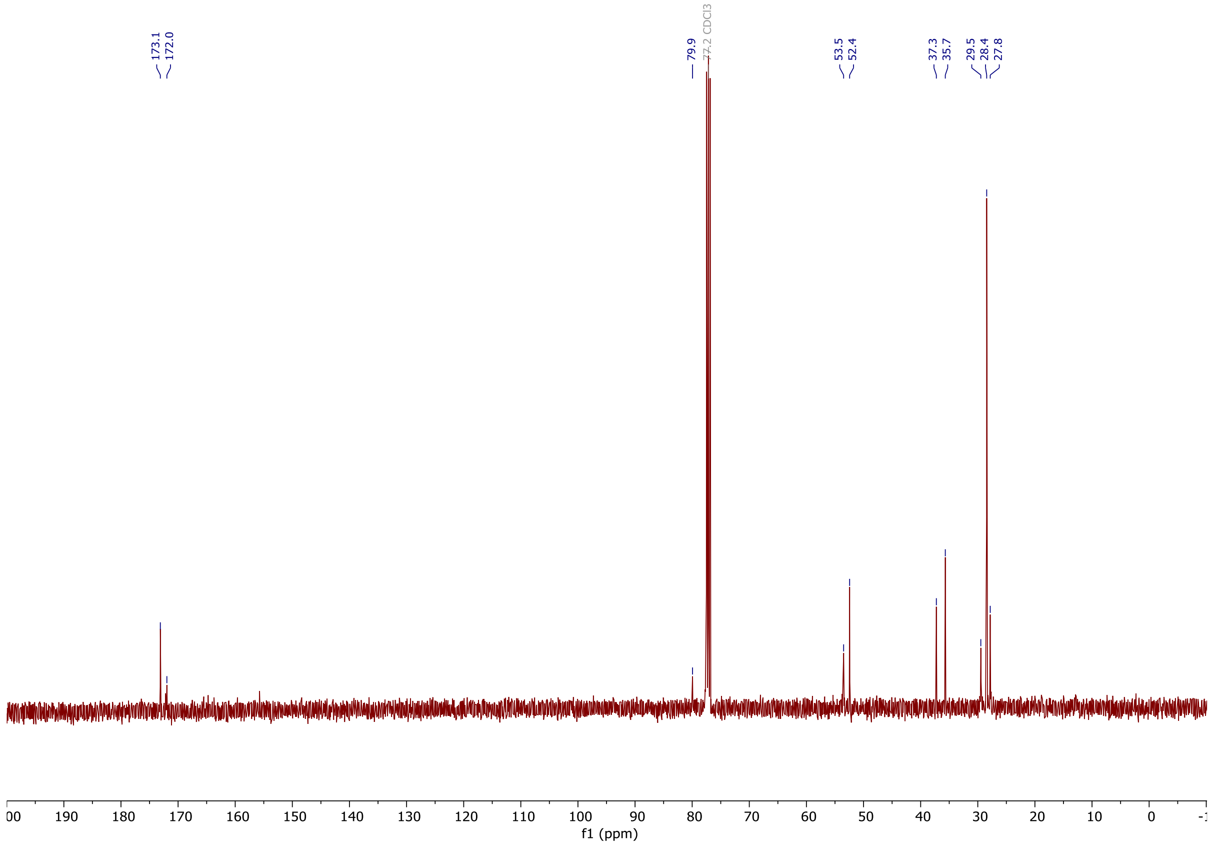
173.1
172.0
79.9
77.2 CDCl3
53.5
52.4
37.3
35.7
29.5
28.4
27.8
190 180 170 160 150 140 130 120 110 100 90 80 70 60 50 40 30 20 10 0
f1 (ppm)

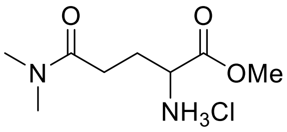
O
O
N
OMe
NH3Cl

S17

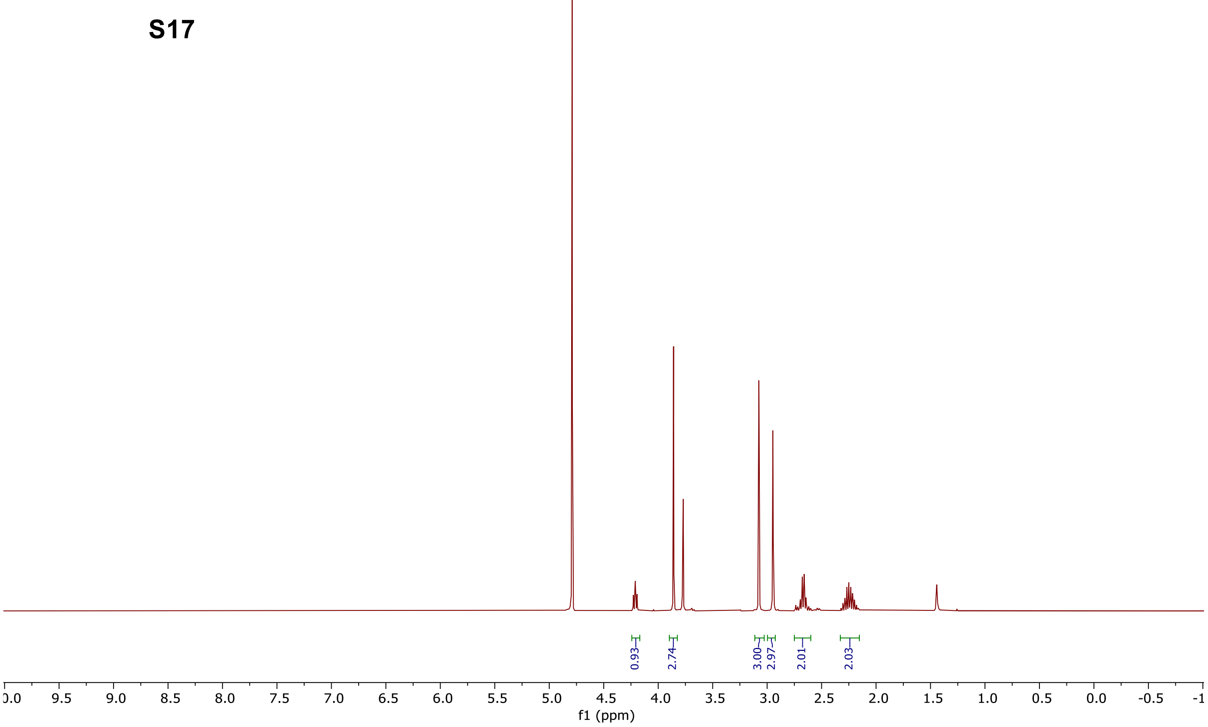
4.79 D2O
0.93
2.74
3.00
2.97
2.01
2.03
f1 (ppm)

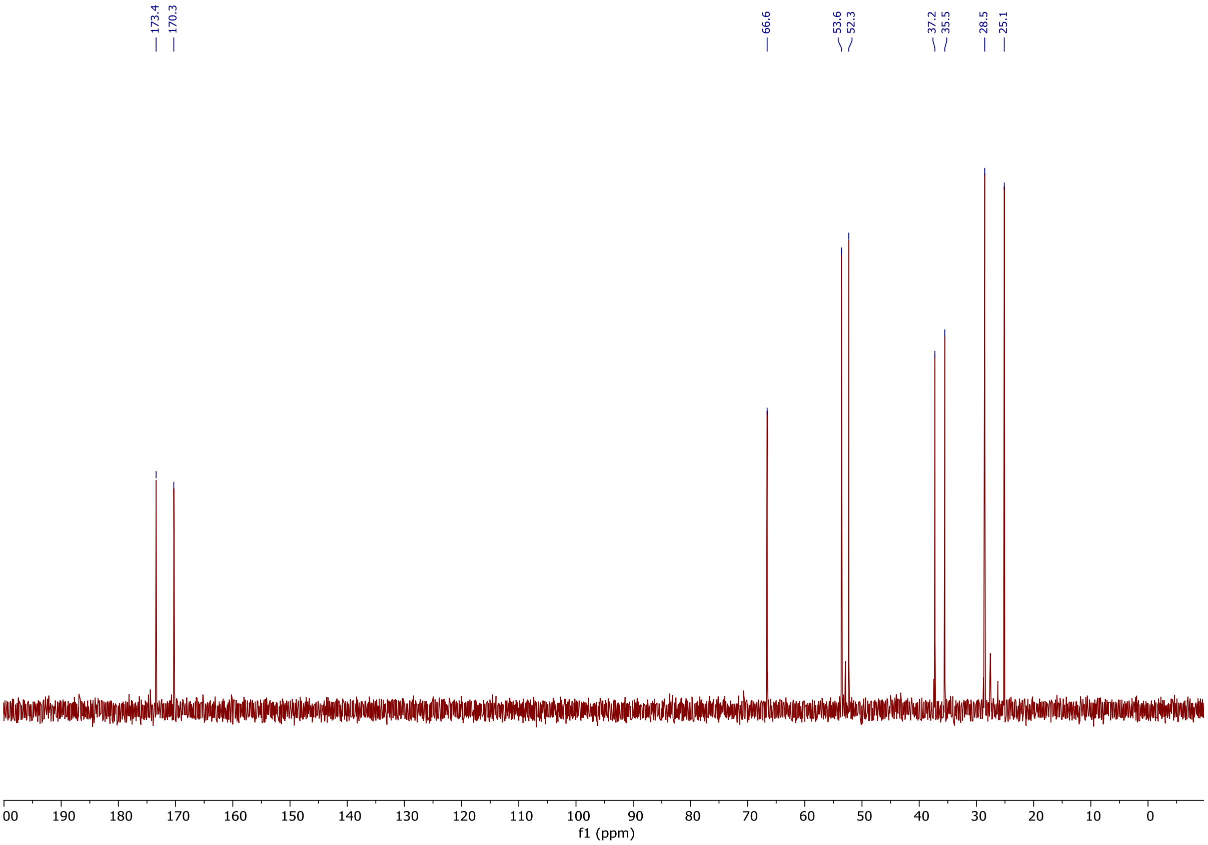
173.4
170.3
66.6
53.6
52.3
37.2
35.5
28.5
25.1
f1 (ppm)

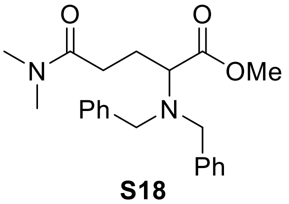
O
O
N
OMe
Ph
N
Ph

S18

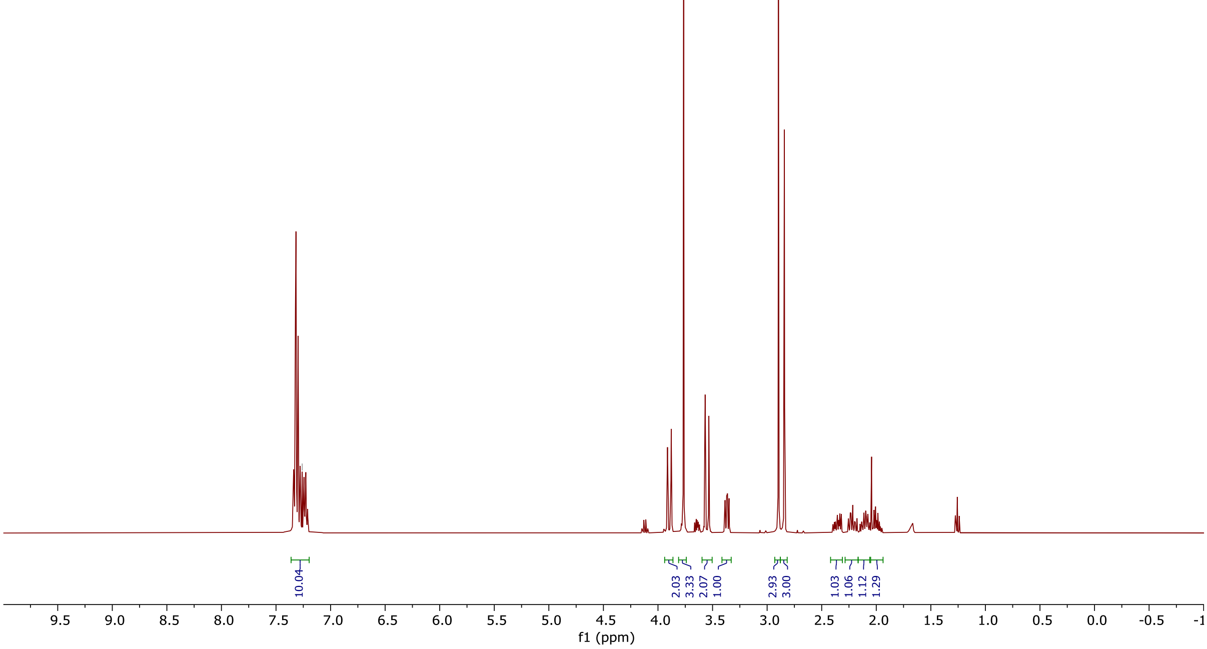
7.26 CDCl3
10.04
2.03
3.33
2.07
1.00
2.93
3.00
1.03
1.06
1.12
1.29
9.5 9.0 8.5 8.0 7.5 7.0 6.5 6.0 5.5 5.0 4.5 4.0 3.5 3.0 2.5 2.0 1.5 1.0 0.5 0.0 -0.5
f1 (ppm)

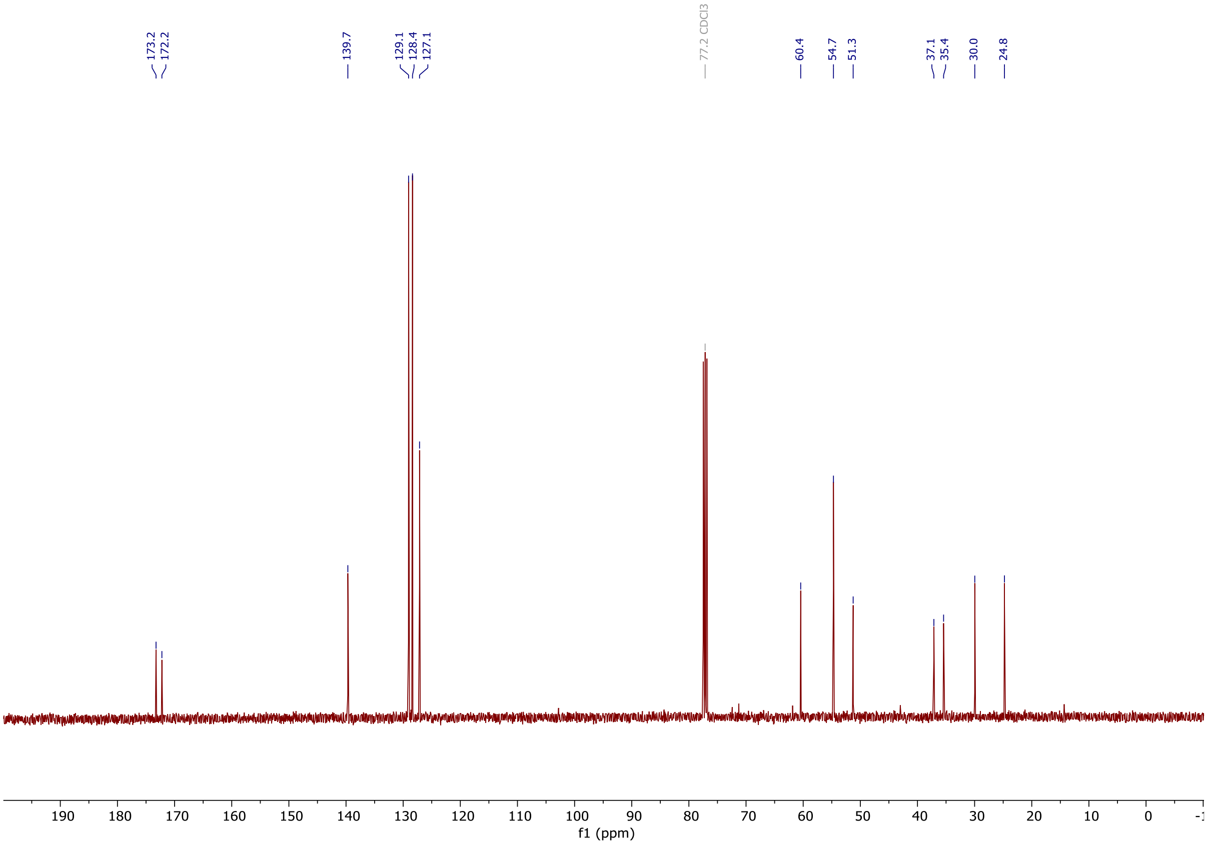
173.2
172.2
139.7
129.1
128.4
127.1
77.2 CDCl3
60.4
54.7
51.3
37.1
35.4
30.0
24.8
190 180 170 160 150 140 130 120 110 100 90 80 70 60 50 40 30 20 10 0
f1 (ppm)

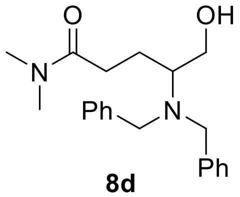
O
OH
N
Ph
N
Ph

8d

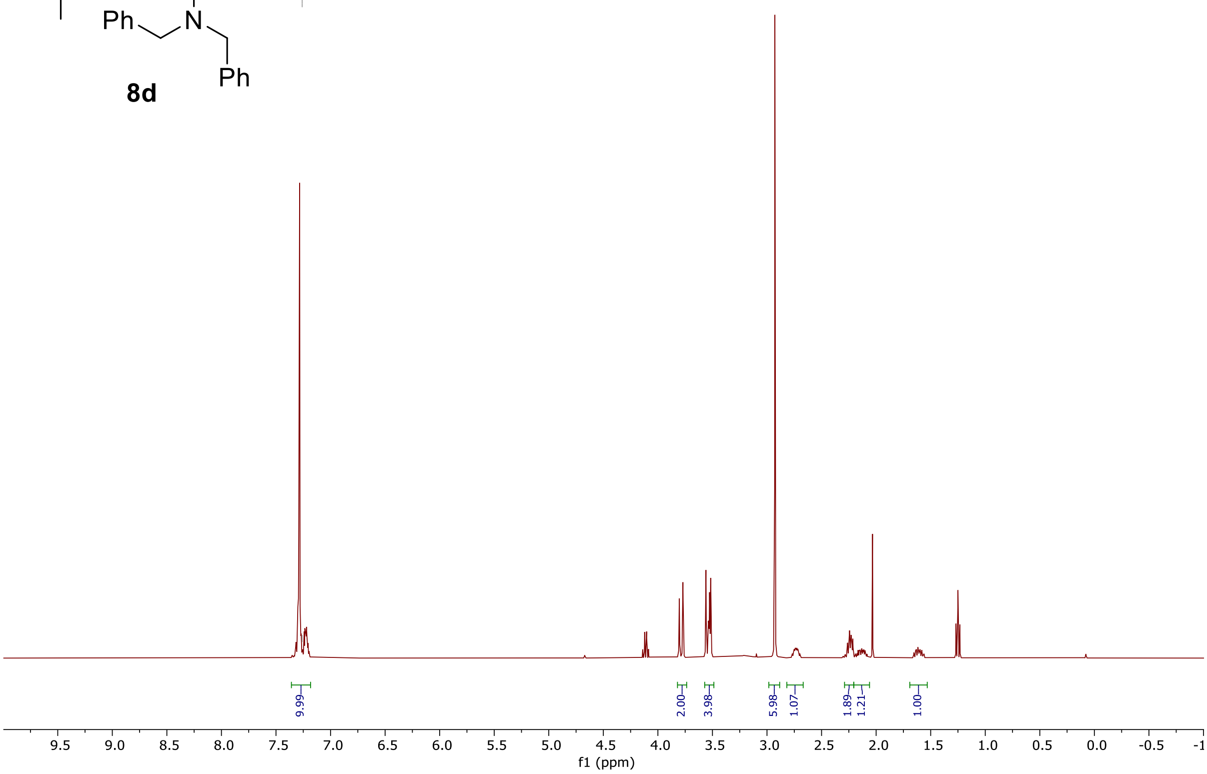
7.26 CDCl3
9.99
2.00
3.98
5.98
1.07
1.89
1.21
1.00
f1 (ppm)

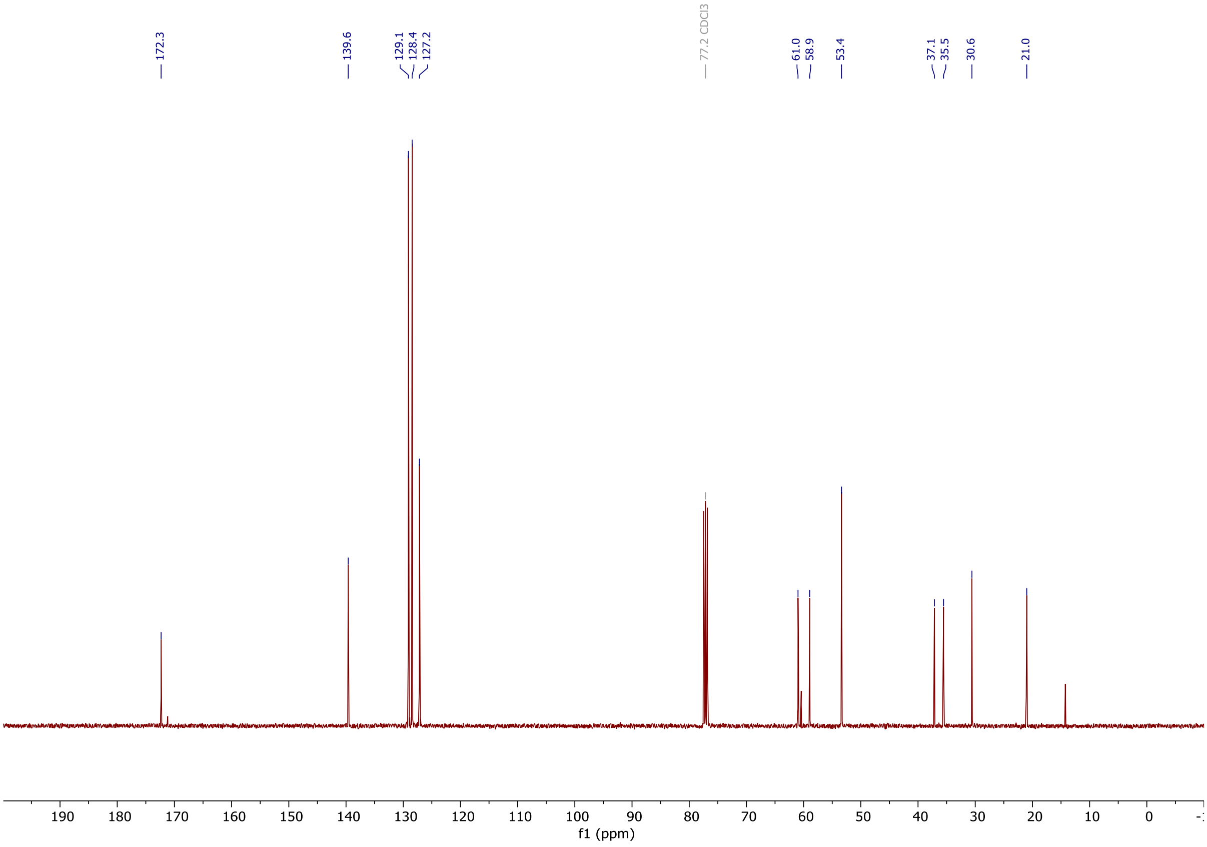
172.3
139.6
129.1
128.4
127.2
77.2 CDCl3
61.0
58.9
53.4
37.1
35.5
30.6
21.0
f1 (ppm)

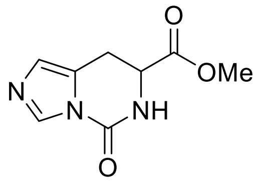
O
OMe
N
N
NH
O

S19

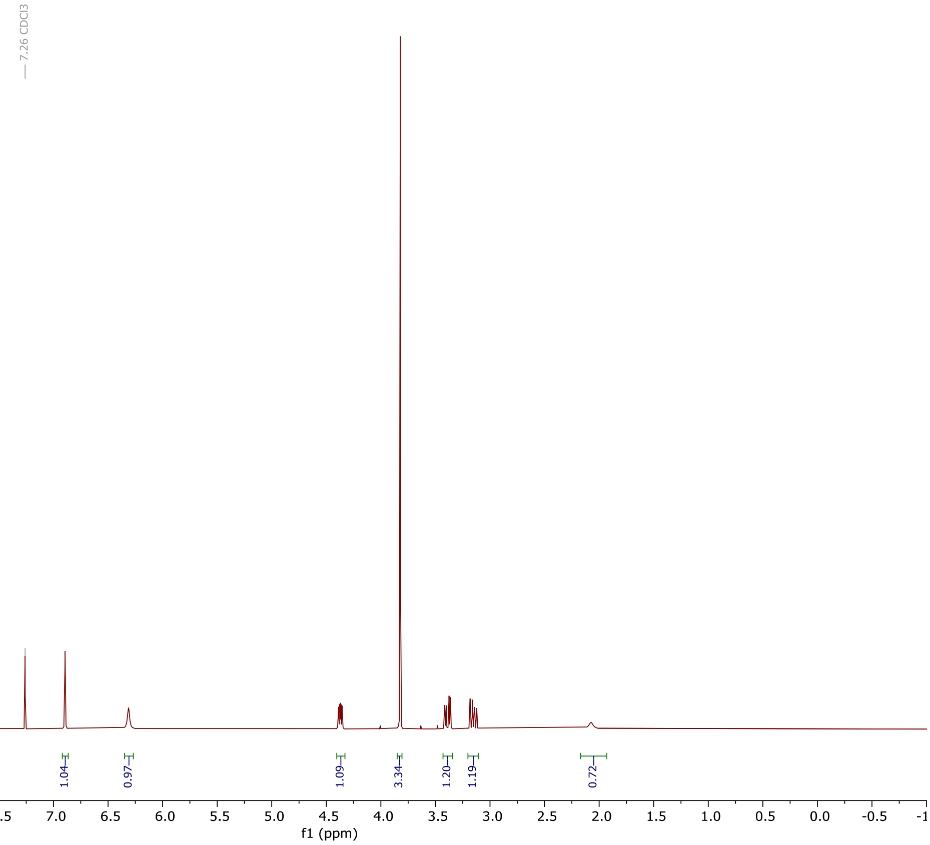
7.26 CDCl3
1.00
1.04
0.97
1.09
3.34
1.20
1.19
0.72
f1 (ppm)

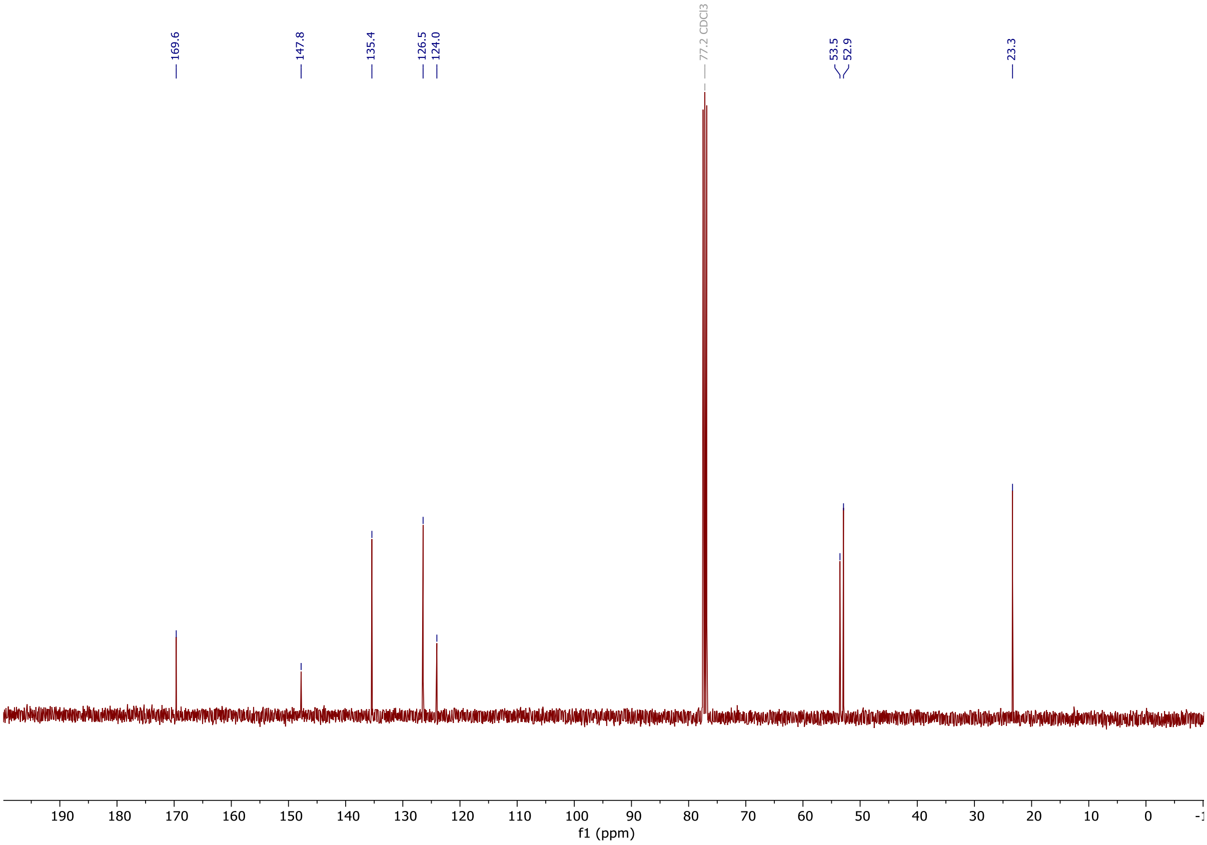
169.6
147.8
135.4
126.5
124.0
77.2 CDCl3
53.5
52.9
23.3
f1 (ppm)

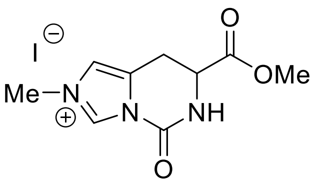
I
Me
N
N
NH
O
OMe
O

S20

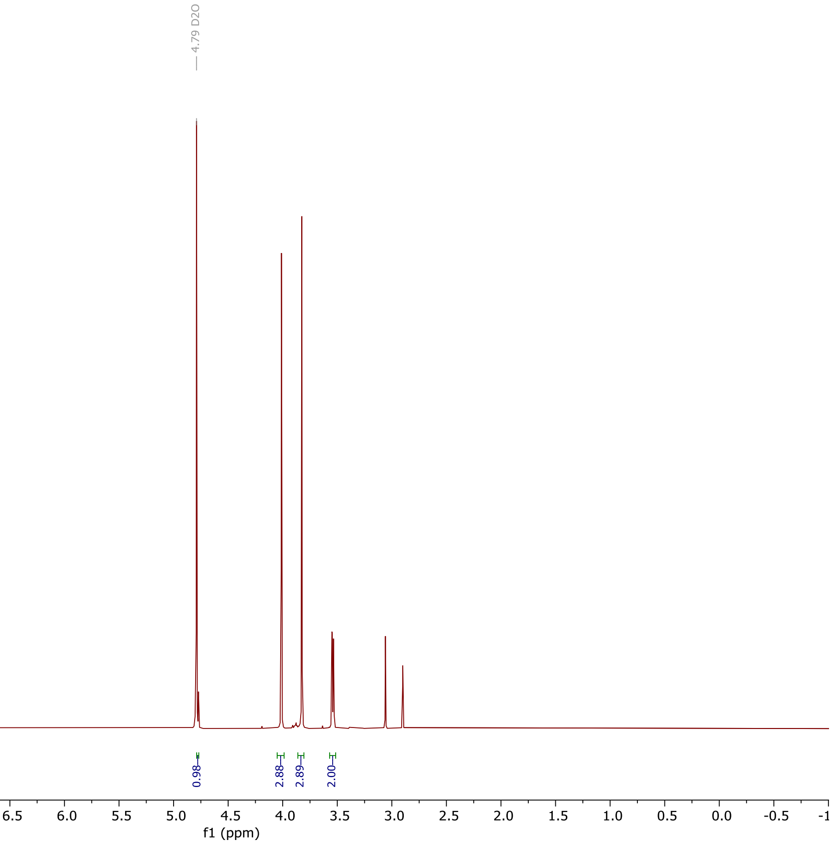
4.79 D2O
0.14
0.80
0.98
2.88
2.89
2.00
9.5 9.0 8.5 8.0 7.5 7.0 6.5 6.0 5.5 5.0 4.5 4.0 3.5 3.0 2.5 2.0 1.5 1.0 0.5 0.0 -0.5
f1 (ppm)

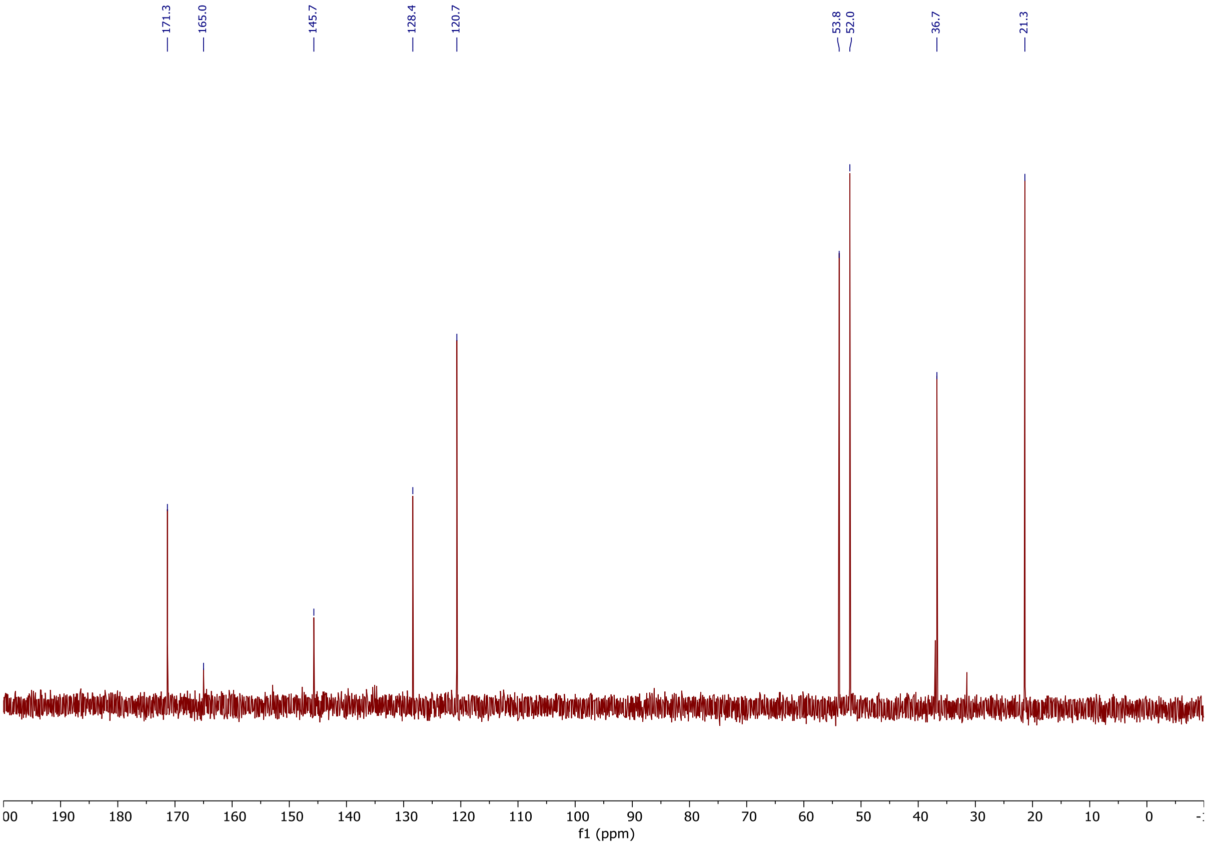
171.3
165.0
145.7
128.4
120.7
53.8
52.0
36.7
21.3
190 180 170 160 150 140 130 120 110 100 90 80 70 60 50 40 30 20 10 0
f1 (ppm)

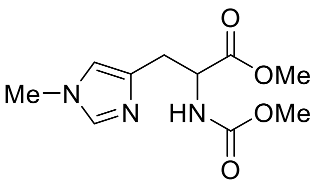

Me
N
N
HN
O
OMe
OMe
O

S21

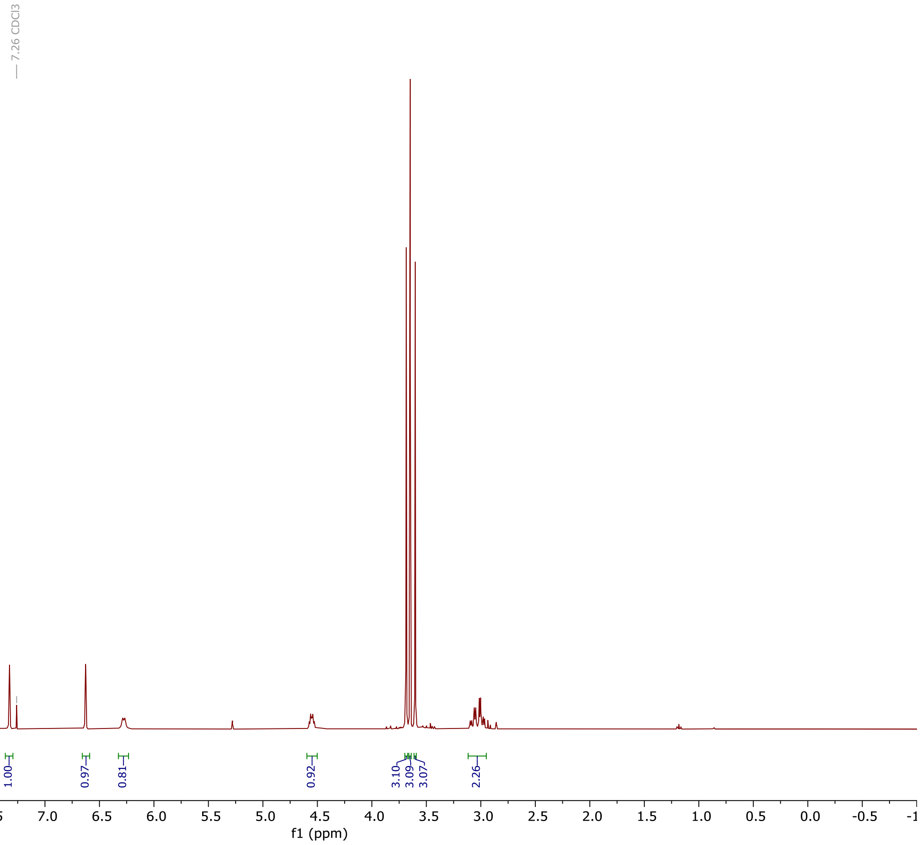

7.26 CDCl3
1.00
0.97
0.81
0.92
3.10
3.09
3.07
2.26
f1 (ppm)

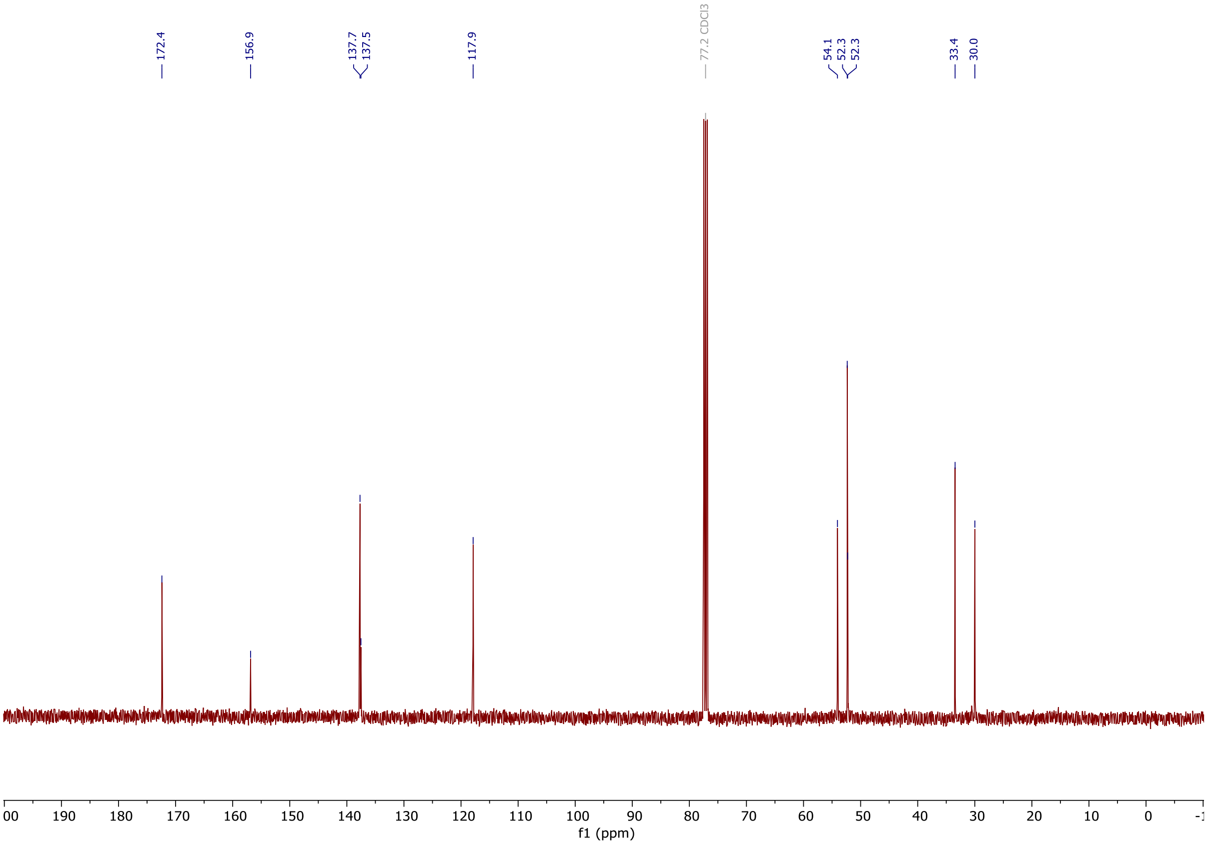

172.4
156.9
137.7
137.5
117.9
77.2 CDCl3
54.1
52.3
52.3
33.4
30.0
f1 (ppm)

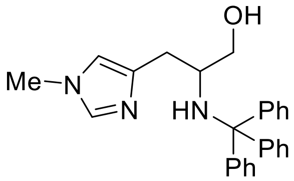
OH
Me
N
N
HN
Ph
Ph
Ph

8e

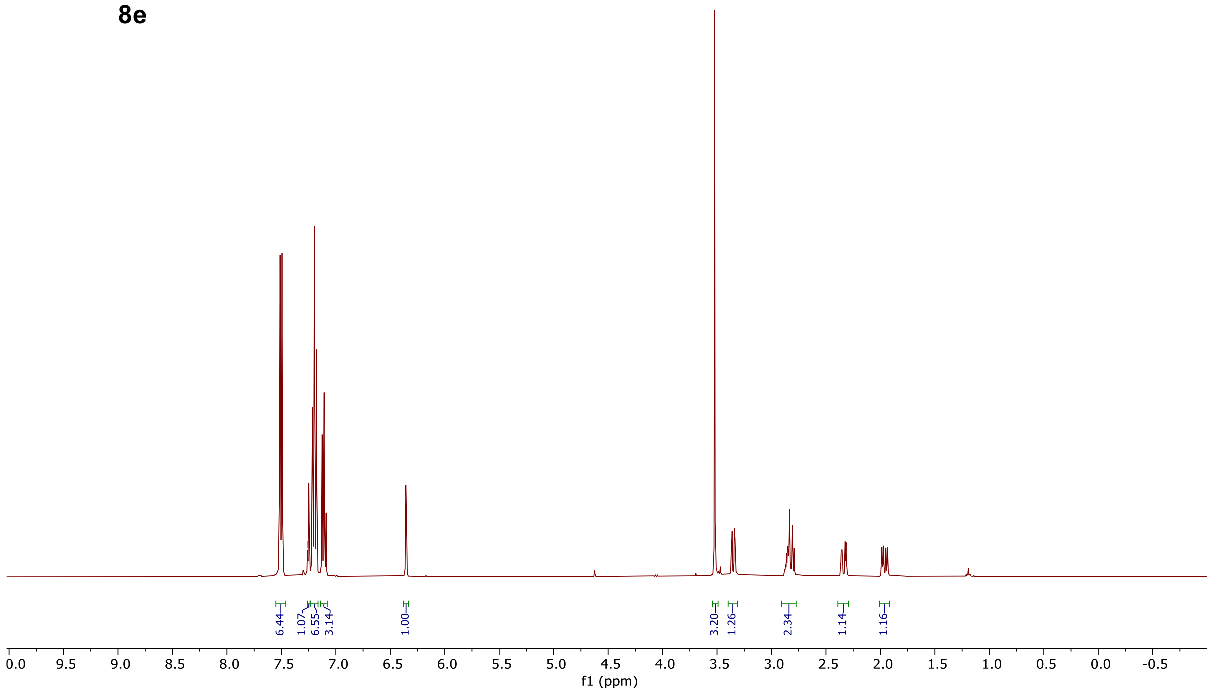
7.26 CDCl3
6.44
1.07
6.55
3.14
1.00
3.20
1.26
2.34
1.14
1.16
f1 (ppm)

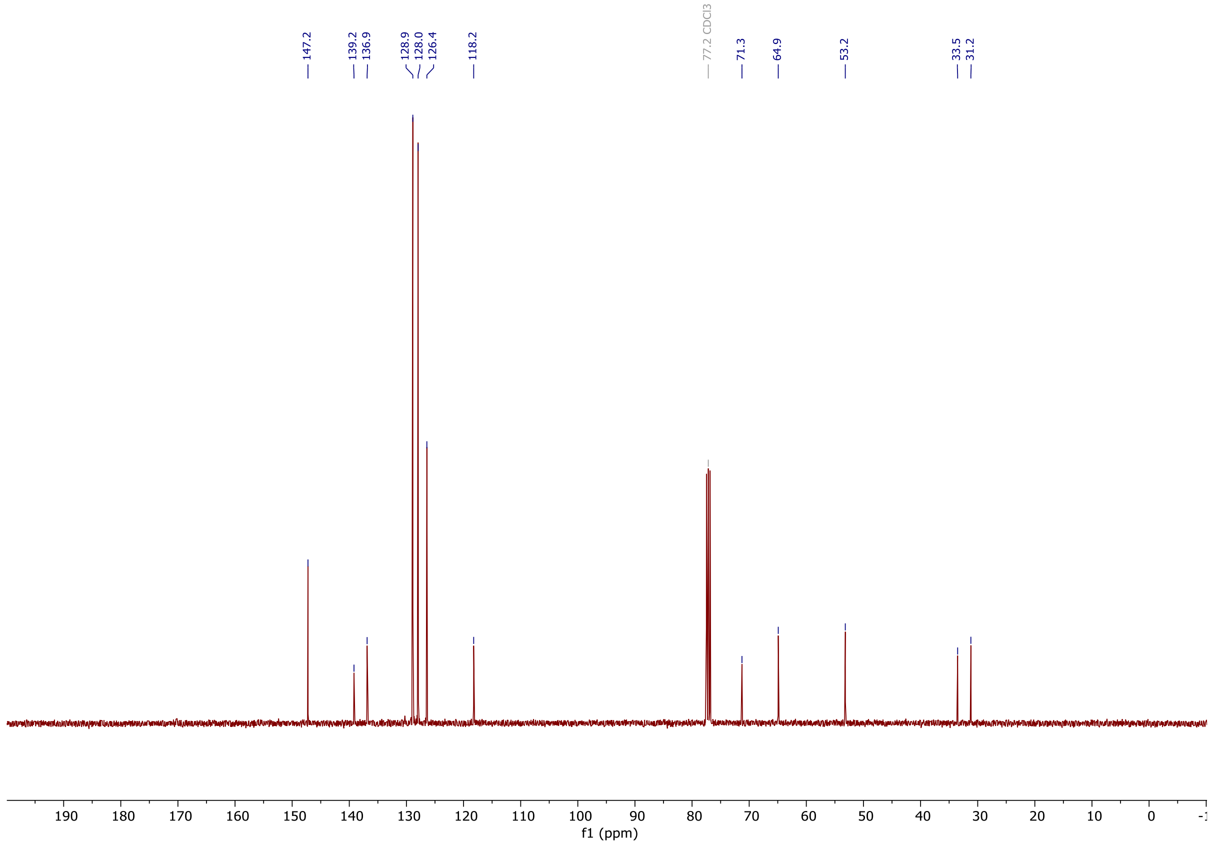
147.2
139.2
136.9
128.9
128.0
126.4
118.2
77.2 CDCl3
71.3
64.9
53.2
33.5
31.2
f1 (ppm)

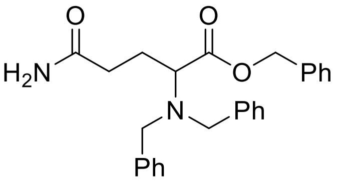
O
O
H2N
O
Ph
N
Ph
Ph

S22

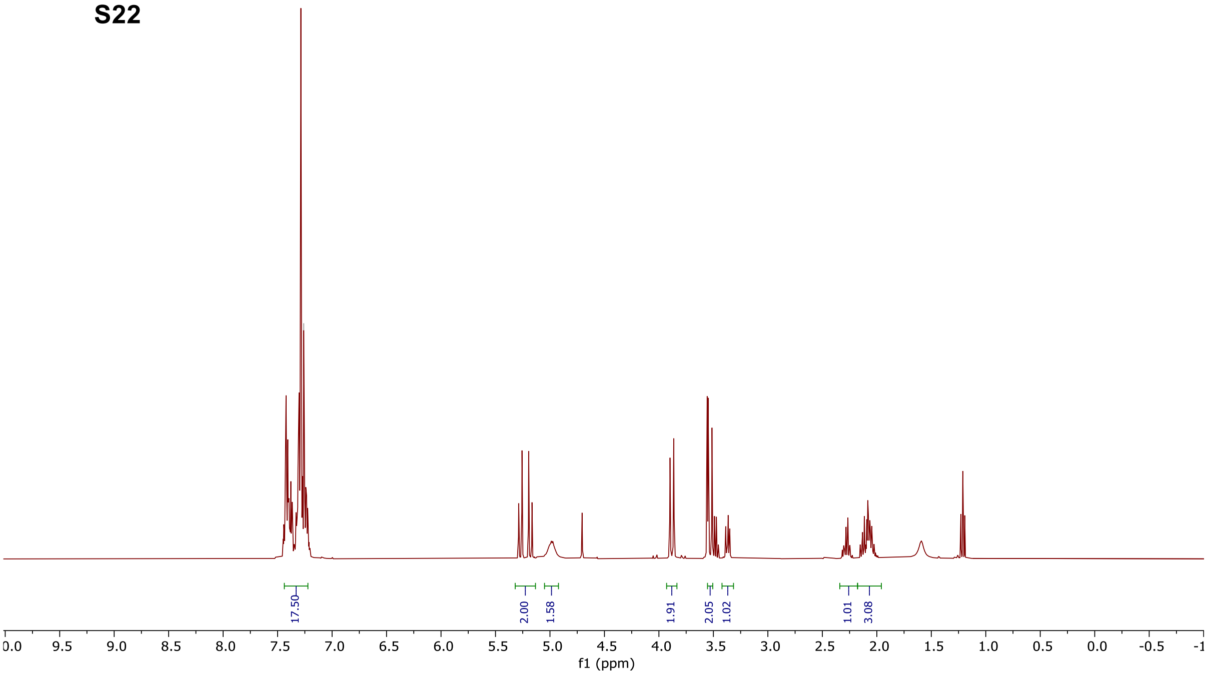
7.26 CDCl3
17.50
2.00
1.58
1.91
2.05
1.02
1.01
3.08
f1 (ppm)

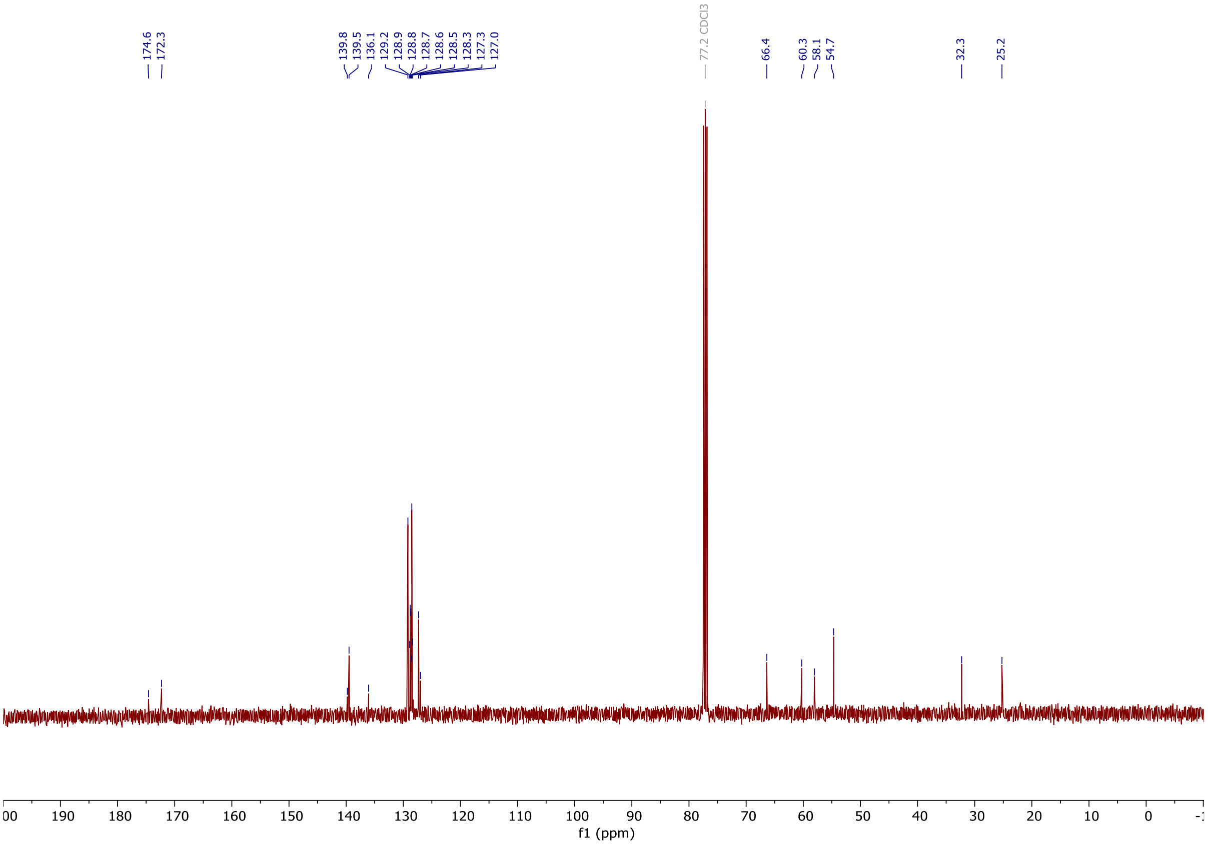
174.6
172.3
139.8
139.5
136.1
129.2
128.9
128.8
128.7
128.6
128.5
128.3
127.3
127.0
77.2 CDCl3
66.4
60.3
58.1
54.7
32.3
25.2
f1 (ppm)

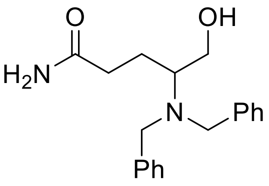

S23

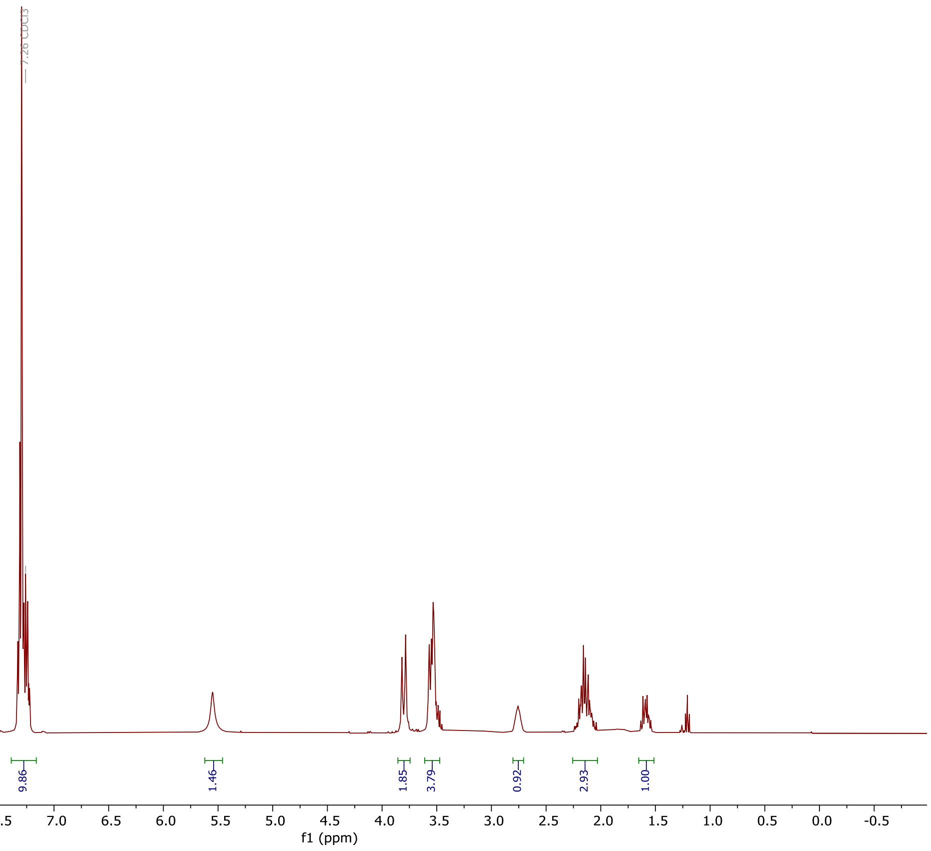
7.26 CDCl3
9.86
1.46
1.85
3.79
0.92
2.93
1.00
9.5
9.0
8.5
8.0
7.5
7.0
6.5
6.0
5.5
5.0
4.5
4.0
3.5
3.0
2.5
2.0
1.5
1.0
0.5
0.0
-0.5
f1 (ppm)

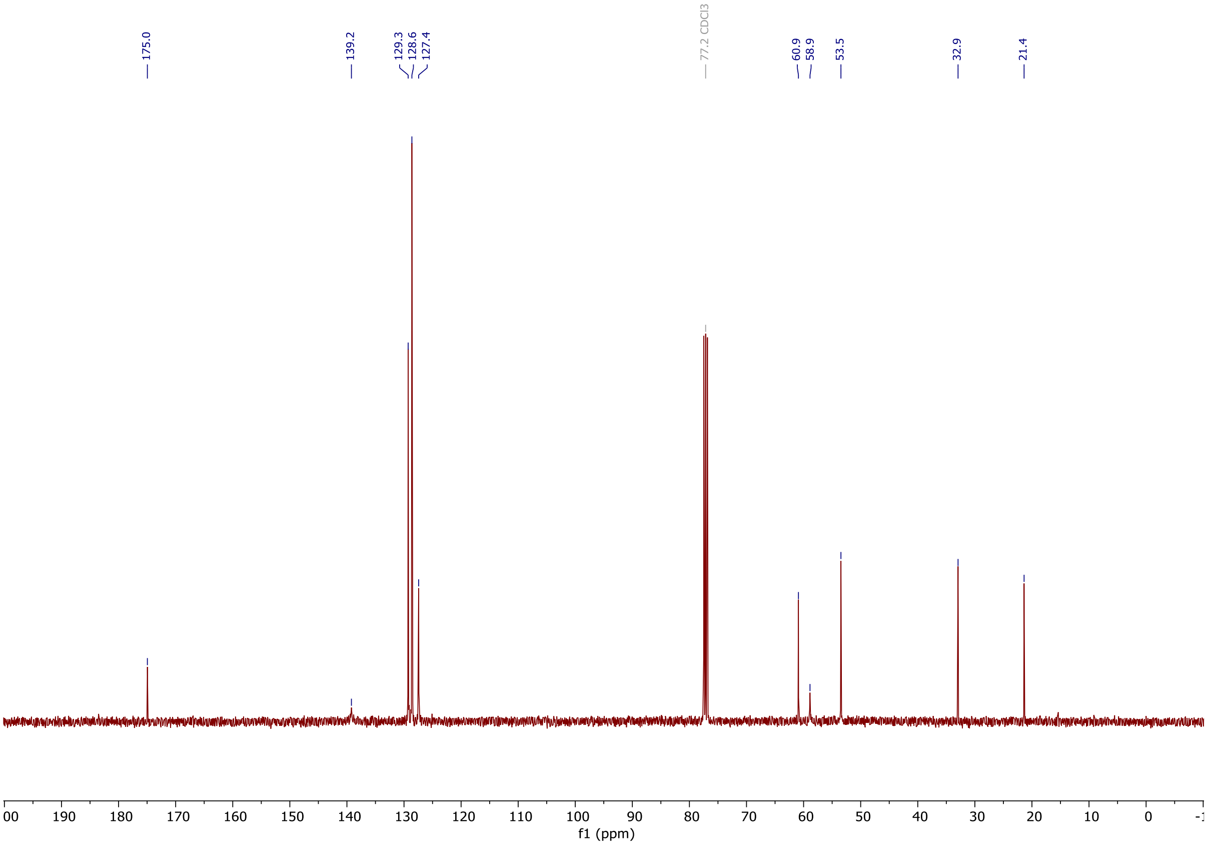
175.0
139.2
129.3
128.6
127.4
77.2 CDCl3
60.9
58.9
53.5
32.9
21.4
190
180
170
160
150
140
130
120
110
100
90
80
70
60
50
40
30
20
10
0
f1 (ppm)

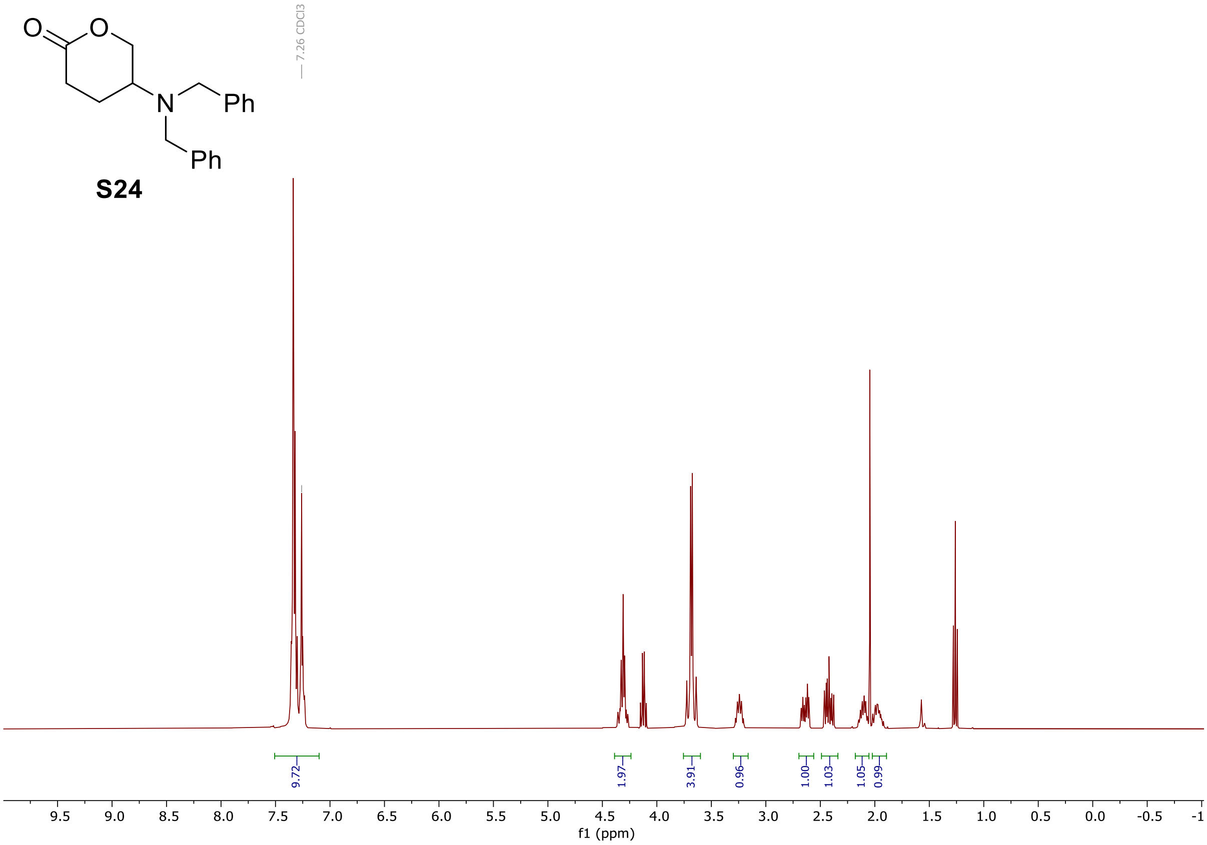
O
O
N
Ph
Ph
S24
7.26 CDCl3
9.72
1.97
3.91
0.96
1.00
1.03
1.05
0.99
f1 (ppm)

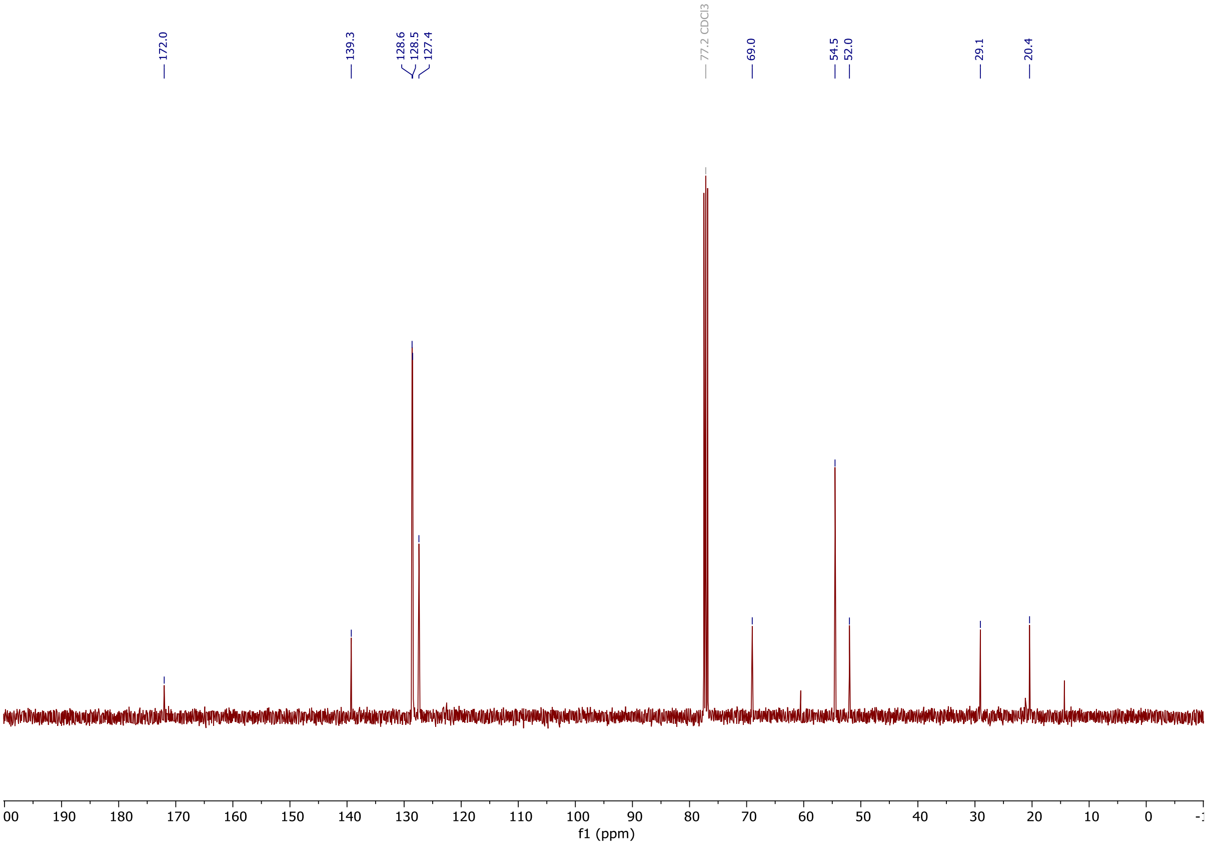
172.0
139.3
128.6
128.5
127.4
77.2 CDCl3
69.0
54.5
52.0
29.1
20.4
f1 (ppm)

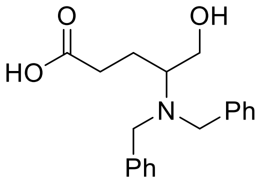
O
OH
HO
N
Ph
Ph

8f

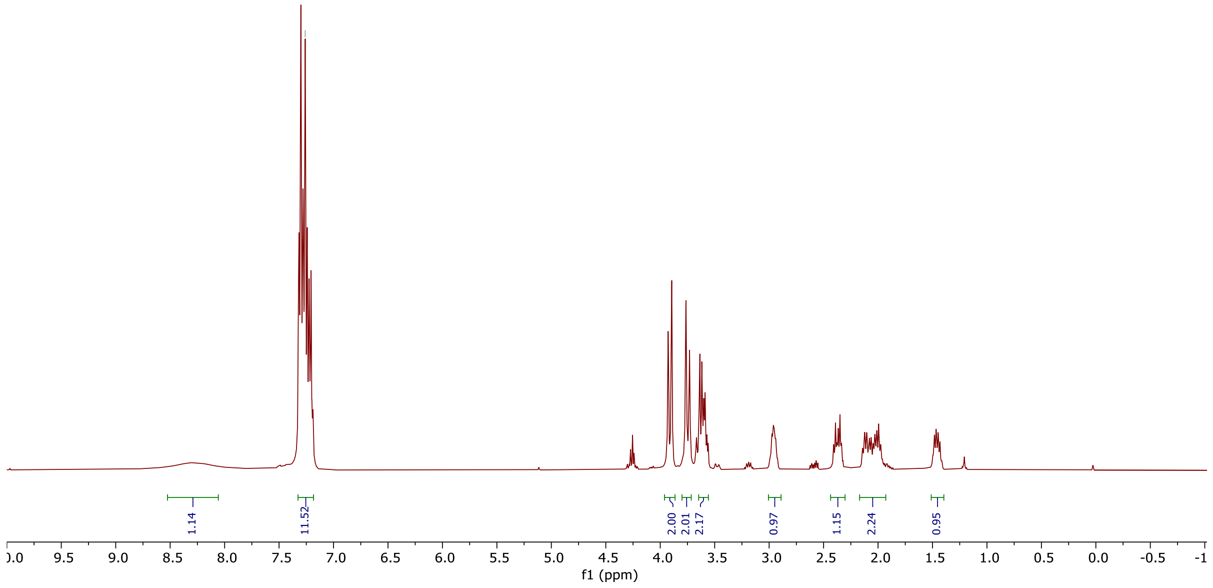
7.26 CDCl3
1.14
11.52
2.00
2.01
2.17
0.97
1.15
2.24
0.95
f1 (ppm)

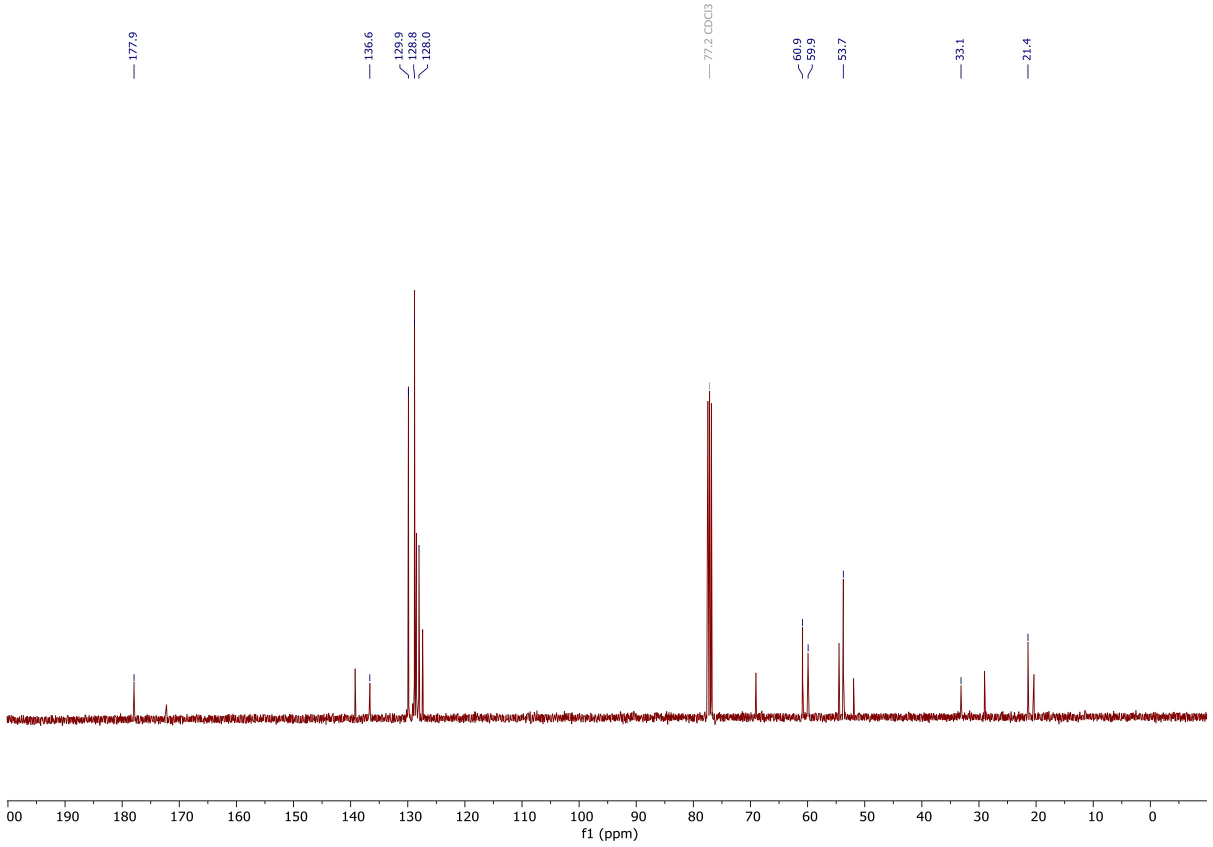
177.9
136.6
129.9
128.8
128.0
77.2 CDCl3
60.9
59.9
53.7
33.1
21.4
f1 (ppm)

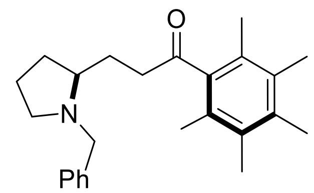

3a

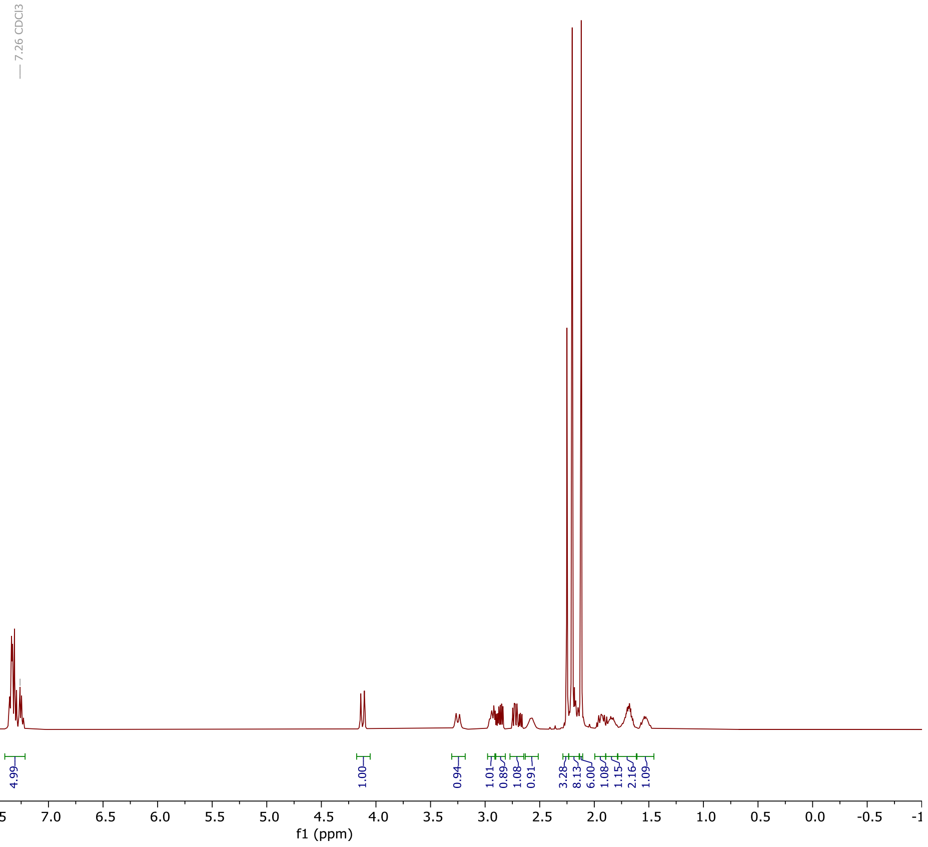

7.26 CDCl3
4.99
1.00
0.94
1.01
0.89
1.08
0.91
3.28
8.13
6.00
1.08
1.15
2.16
1.09
9.5 9.0 8.5 8.0 7.5 7.0 6.5 6.0 5.5 5.0 4.5 4.0 3.5 3.0 2.5 2.0 1.5 1.0 0.5 0.0 -0.5
f1 (ppm)

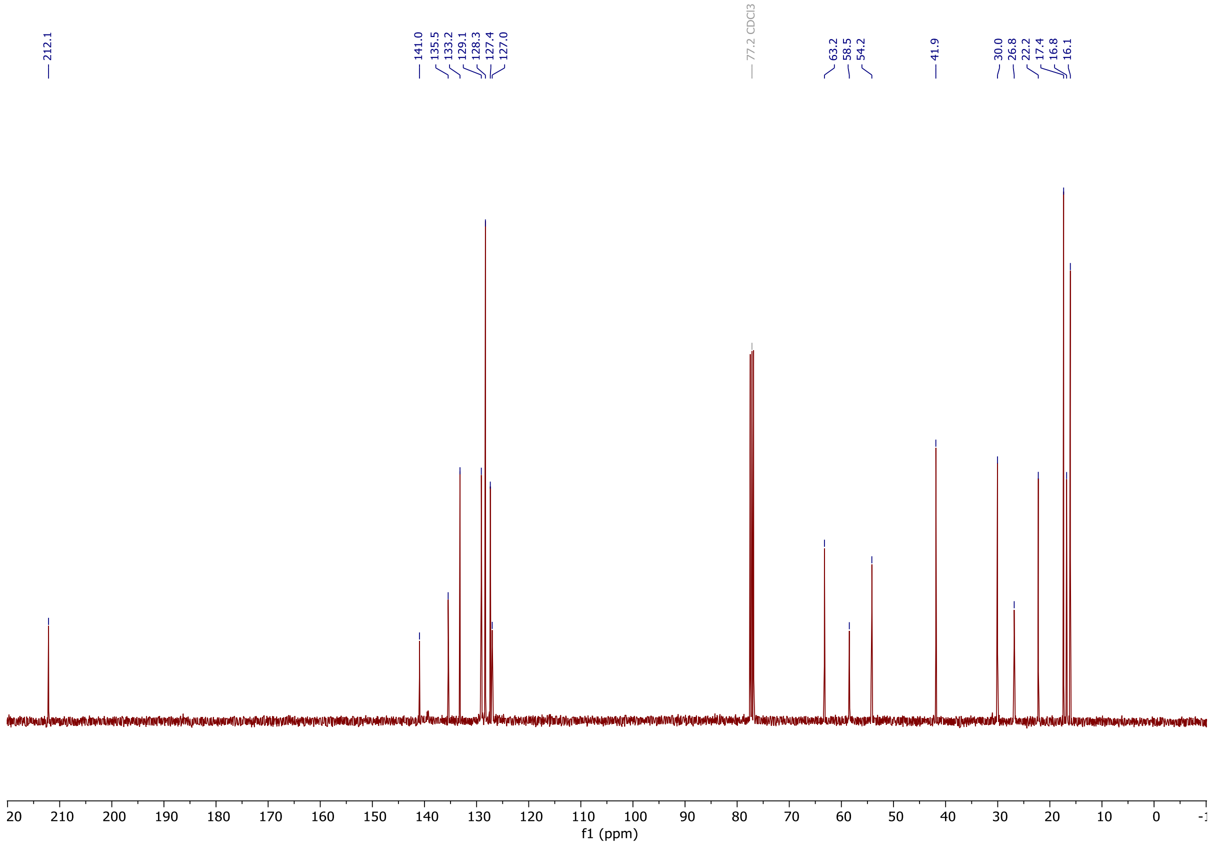

212.1
141.0
135.5
133.2
129.1
128.3
127.4
127.0
77.2 CDCl3
63.2
58.5
54.2
41.9
30.0
26.8
22.2
17.4
16.8
16.1
210 200 190 180 170 160 150 140 130 120 110 100 90 80 70 60 50 40 30 20 10 0
f1 (ppm)

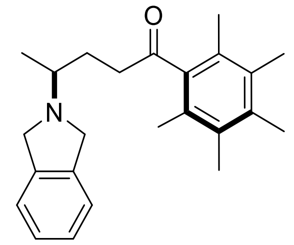

6a

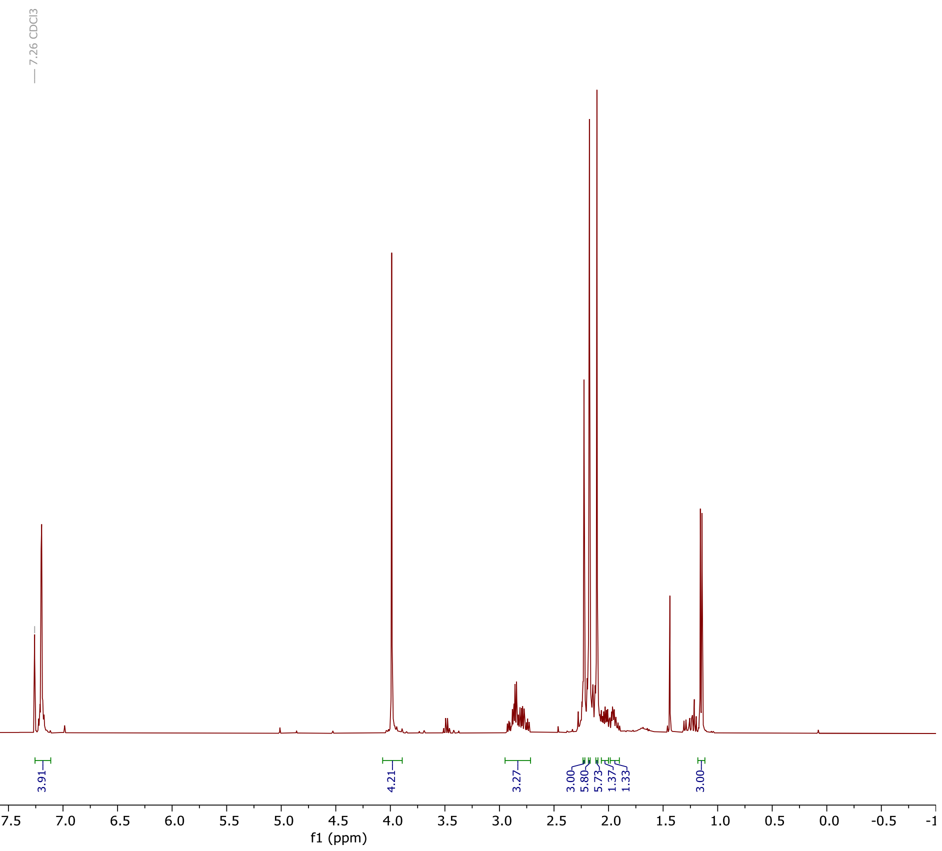

7.26 CDCl3
3.91
4.21
3.27
3.00
5.80
5.73
1.37
1.33
3.00
f1 (ppm)

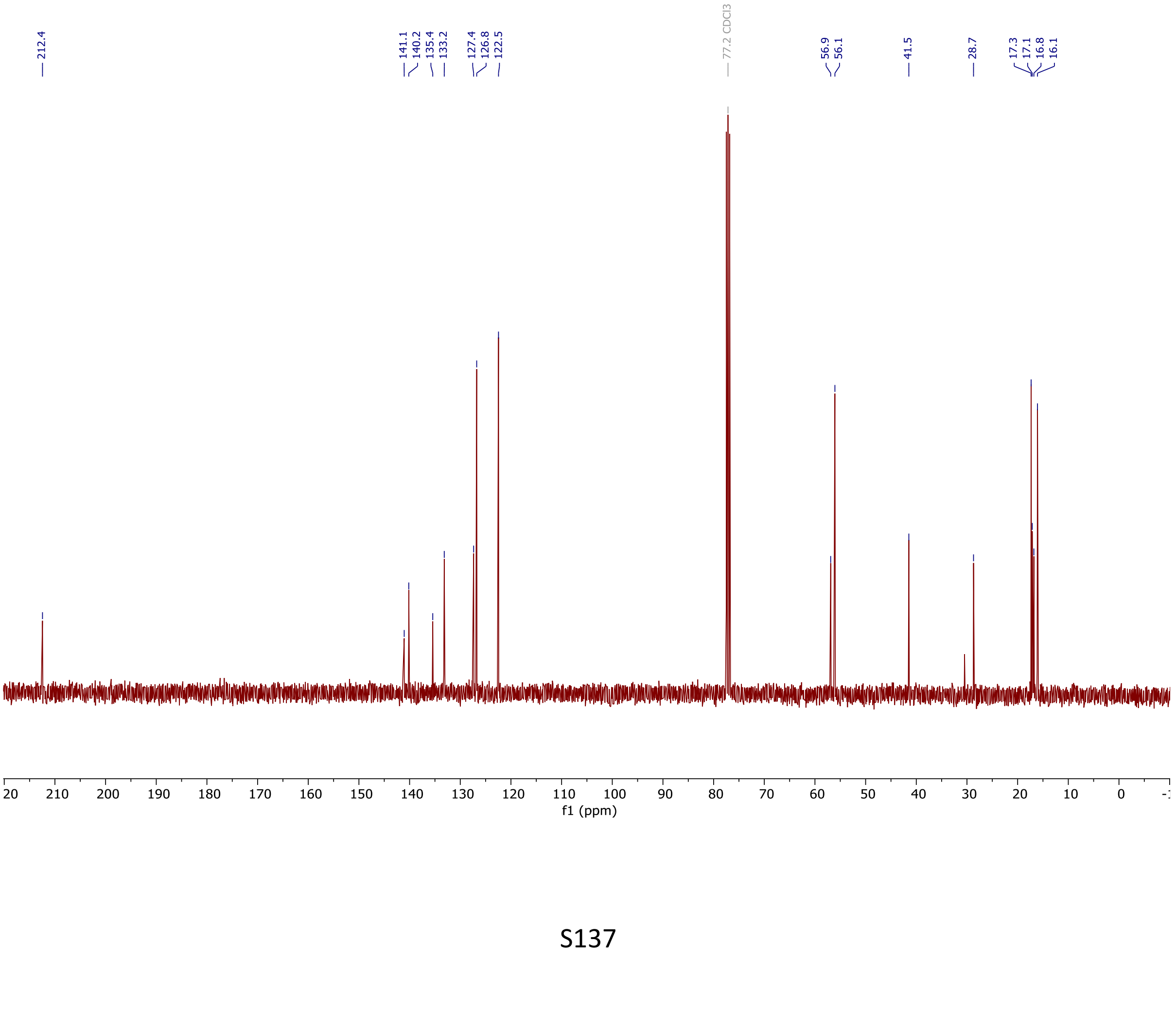

212.4
141.1
140.2
135.4
133.2
127.4
126.8
122.5
77.2 CDCl3
56.9
56.1
41.5
28.7
17.3
17.1
16.8
16.1
f1 (ppm)

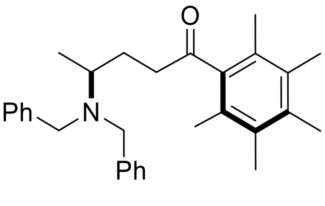
O
Ph
N
Ph

6b

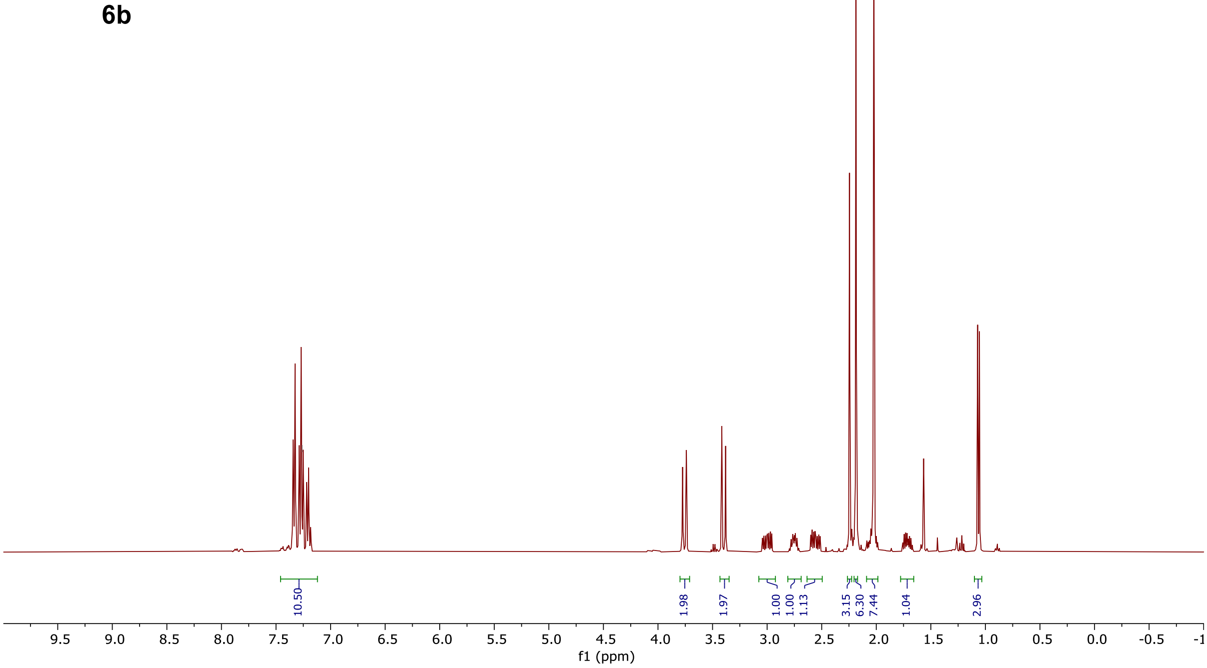
7.26 CDCl3
10.50
1.98
1.97
1.00
1.00
1.13
3.15
6.30
7.44
1.04
2.96
9.5 9.0 8.5 8.0 7.5 7.0 6.5 6.0 5.5 5.0 4.5 4.0 3.5 3.0 2.5 2.0 1.5 1.0 0.5 0.0 -0.5 -1
f1 (ppm)

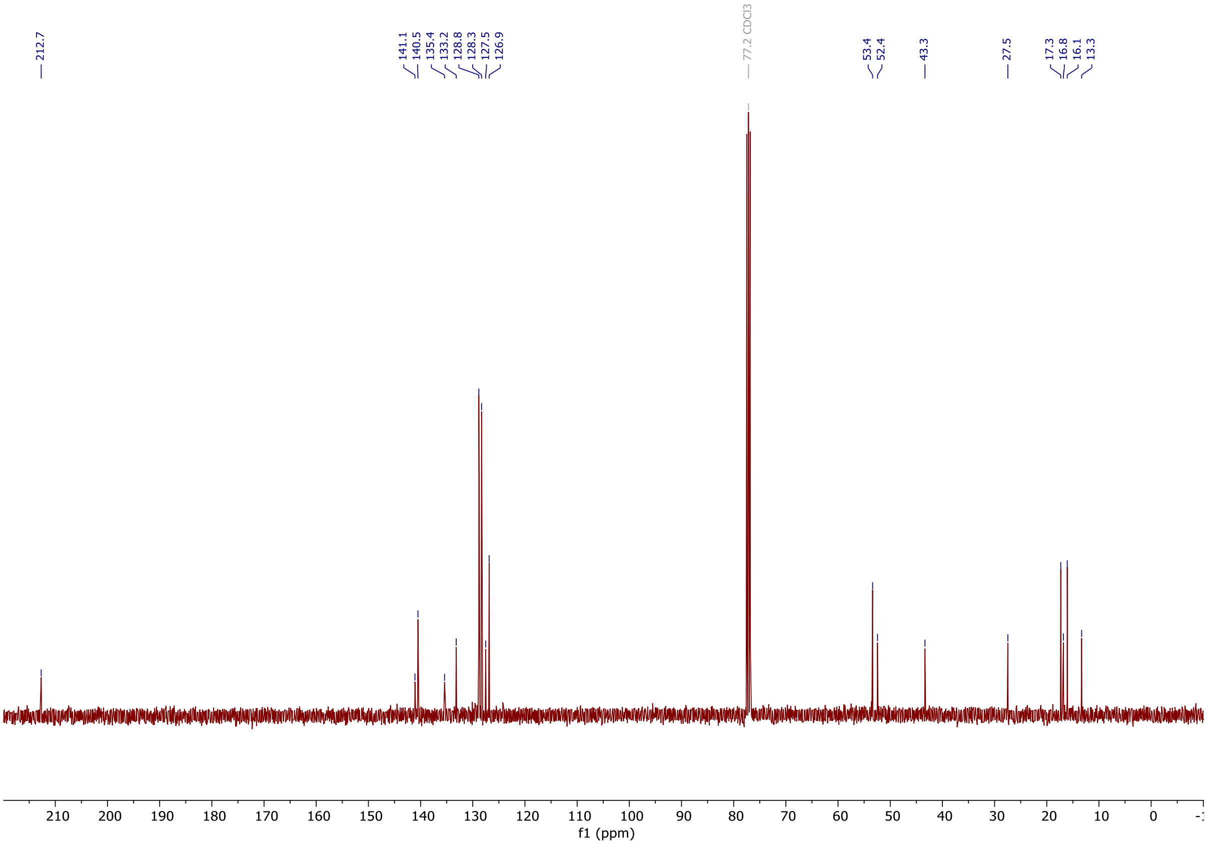
212.7
141.1
140.5
135.4
133.2
128.8
128.3
127.5
126.9
77.2 CDCl3
53.4
52.4
43.3
27.5
17.3
16.8
16.1
13.3
210 200 190 180 170 160 150 140 130 120 110 100 90 80 70 60 50 40 30 20 10 0
f1 (ppm)

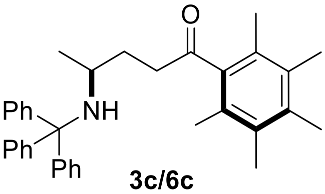
O
NH
Ph
Ph
Ph

3c/6c

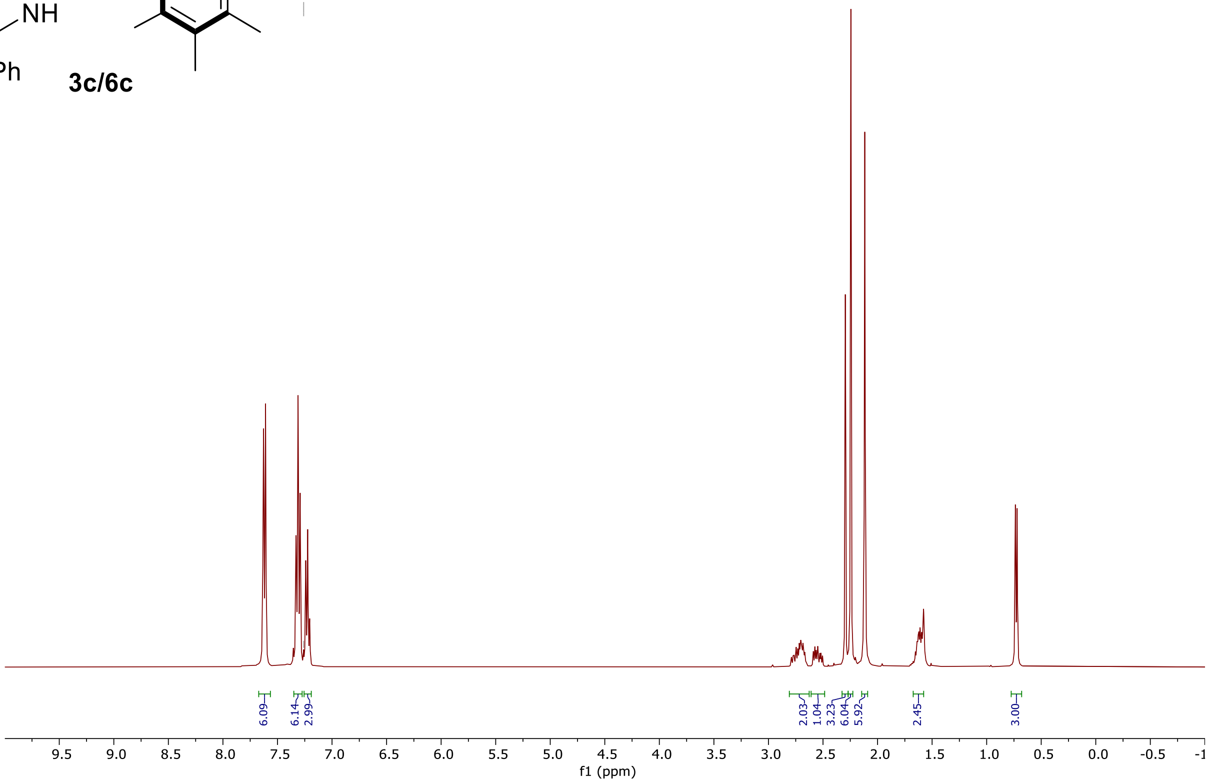
7.26 CDCl3
6.09
6.14
2.99
2.03
1.04
3.23
6.04
5.92
2.45
3.00
9.5 9.0 8.5 8.0 7.5 7.0 6.5 6.0 5.5 5.0 4.5 4.0 3.5 3.0 2.5 2.0 1.5 1.0 0.5 0.0 -0.5 -1
f1 (ppm)

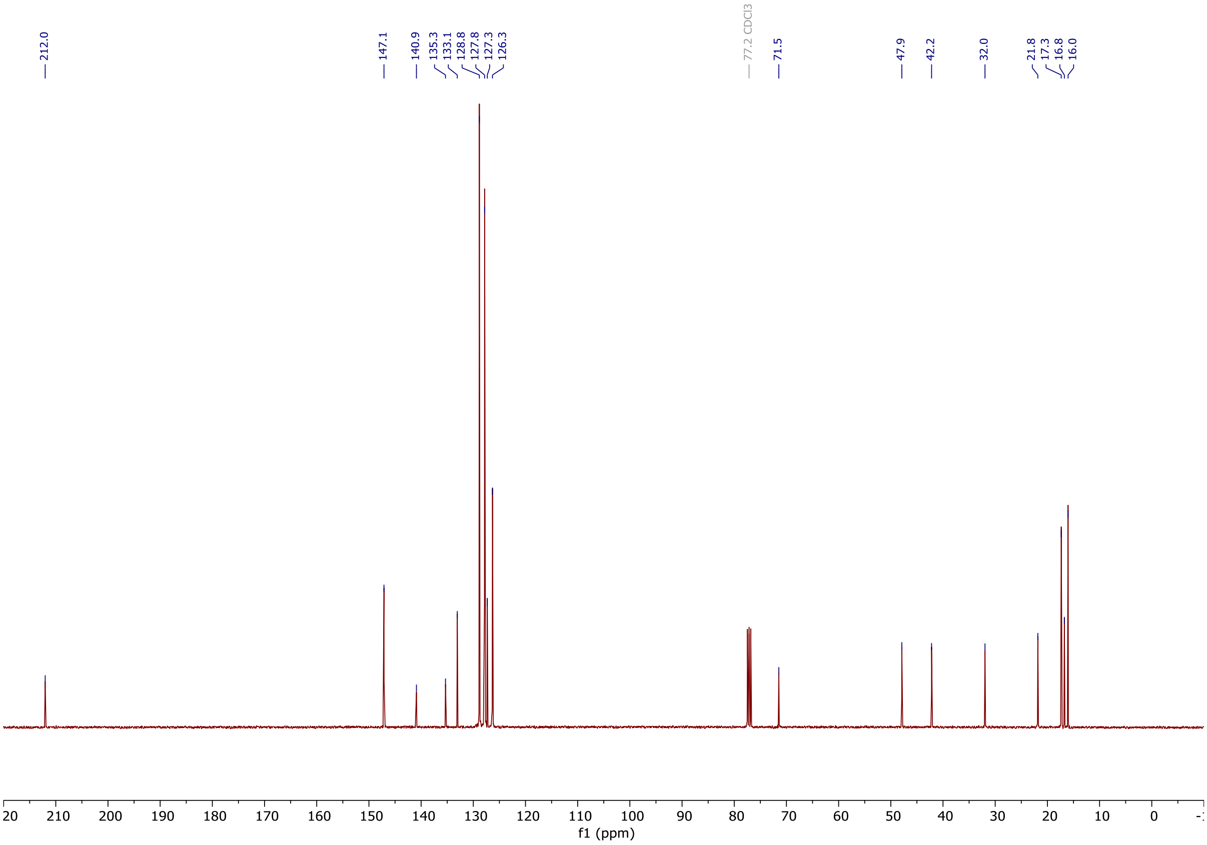
212.0
147.1
140.9
135.3
133.1
128.8
127.8
127.3
126.3
77.2 CDCl3
71.5
47.9
42.2
32.0
21.8
17.3
16.8
16.0
20 210 200 190 180 170 160 150 140 130 120 110 100 90 80 70 60 50 40 30 20 10 0
f1 (ppm)

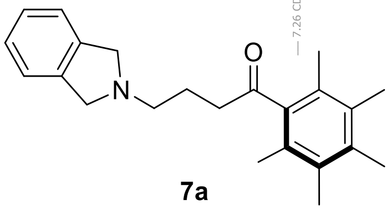

7a

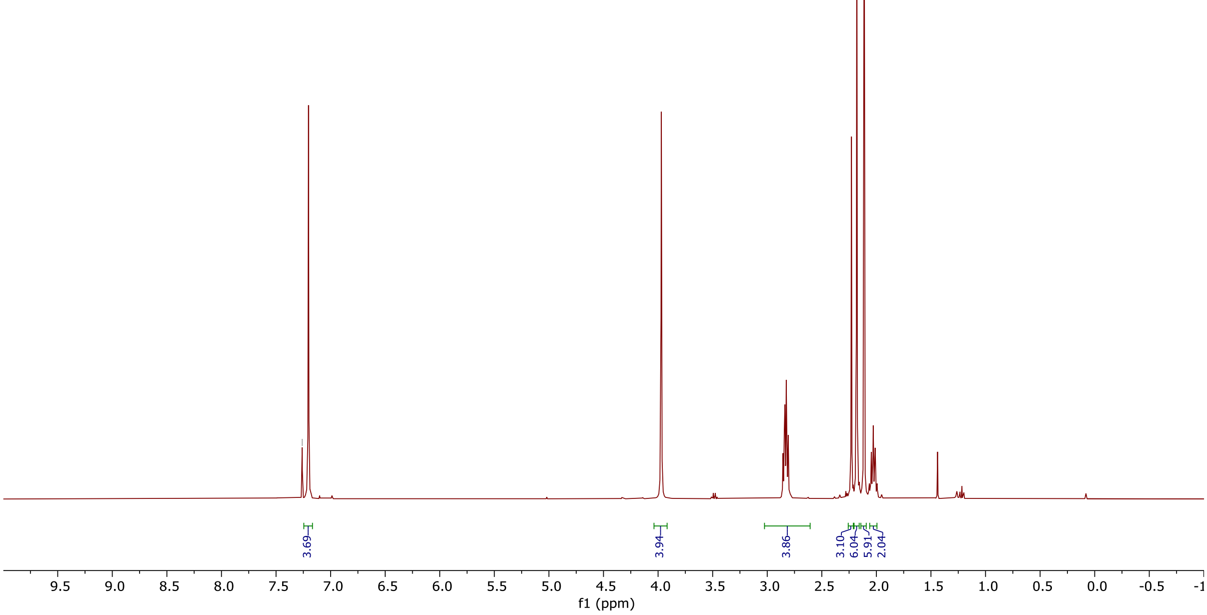

7.26 CDCl3
3.69
3.94
3.86
3.10
6.04
5.91
2.04
9.5 9.0 8.5 8.0 7.5 7.0 6.5 6.0 5.5 5.0 4.5 4.0 3.5 3.0 2.5 2.0 1.5 1.0 0.5 0.0 -0.5
f1 (ppm)

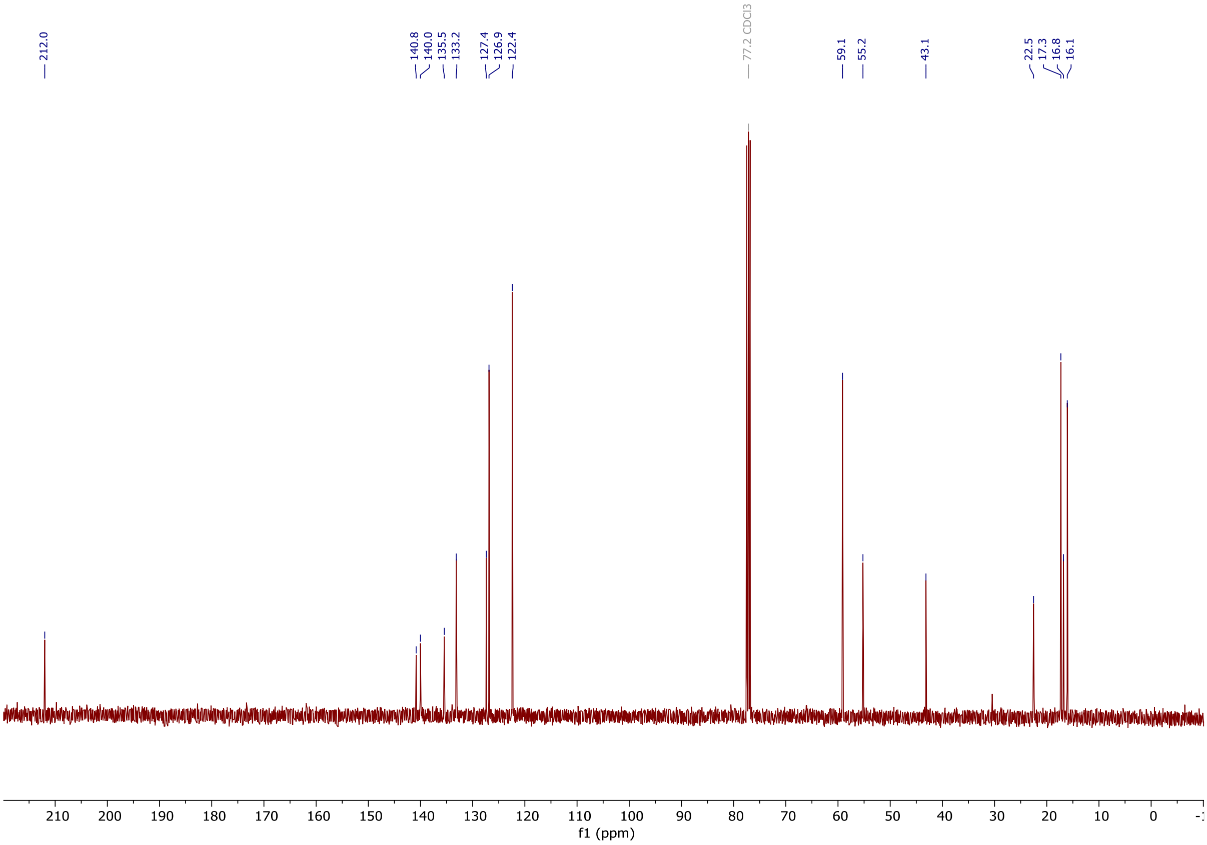

212.0
140.8
140.0
135.5
133.2
127.4
126.9
122.4
77.2 CDCl3
59.1
55.2
43.1
22.5
17.3
16.8
16.1
210 200 190 180 170 160 150 140 130 120 110 100 90 80 70 60 50 40 30 20 10 0
f1 (ppm)

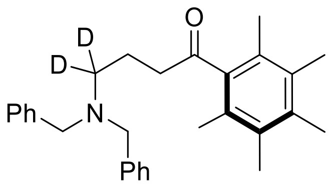

7b

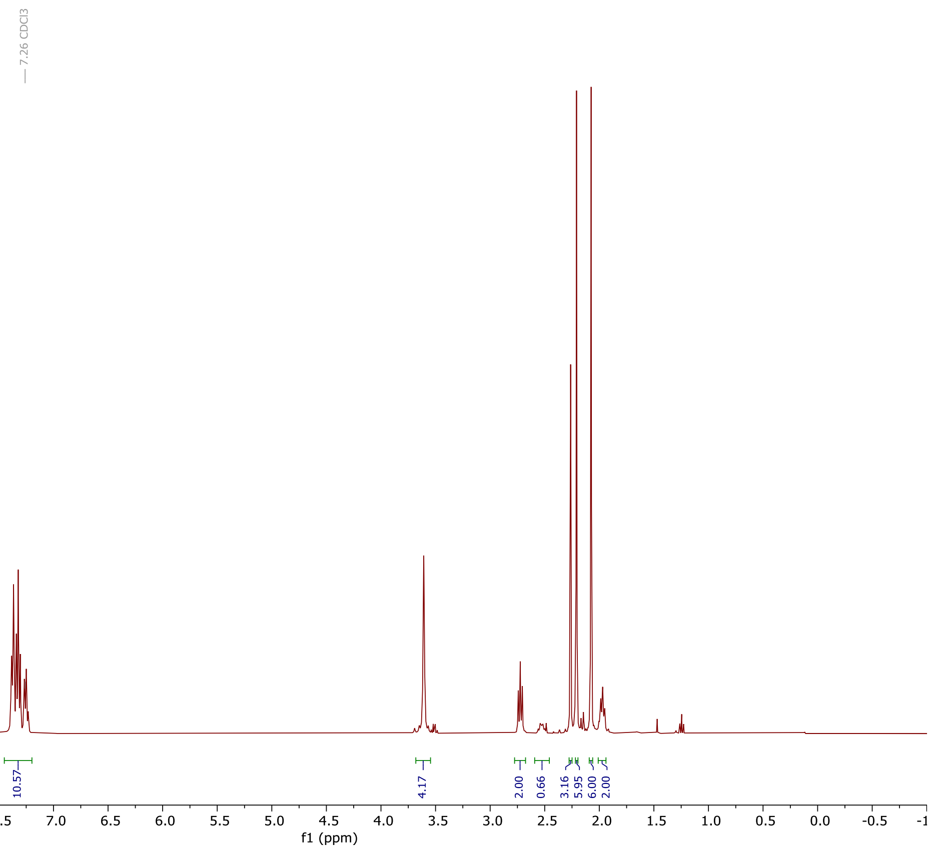
7.26 CDCl3
10.57
4.17
2.00
0.66
3.16
5.95
6.00
2.00
9.5 9.0 8.5 8.0 7.5 7.0 6.5 6.0 5.5 5.0 4.5 4.0 3.5 3.0 2.5 2.0 1.5 1.0 0.5 0.0 -0.5
f1 (ppm)

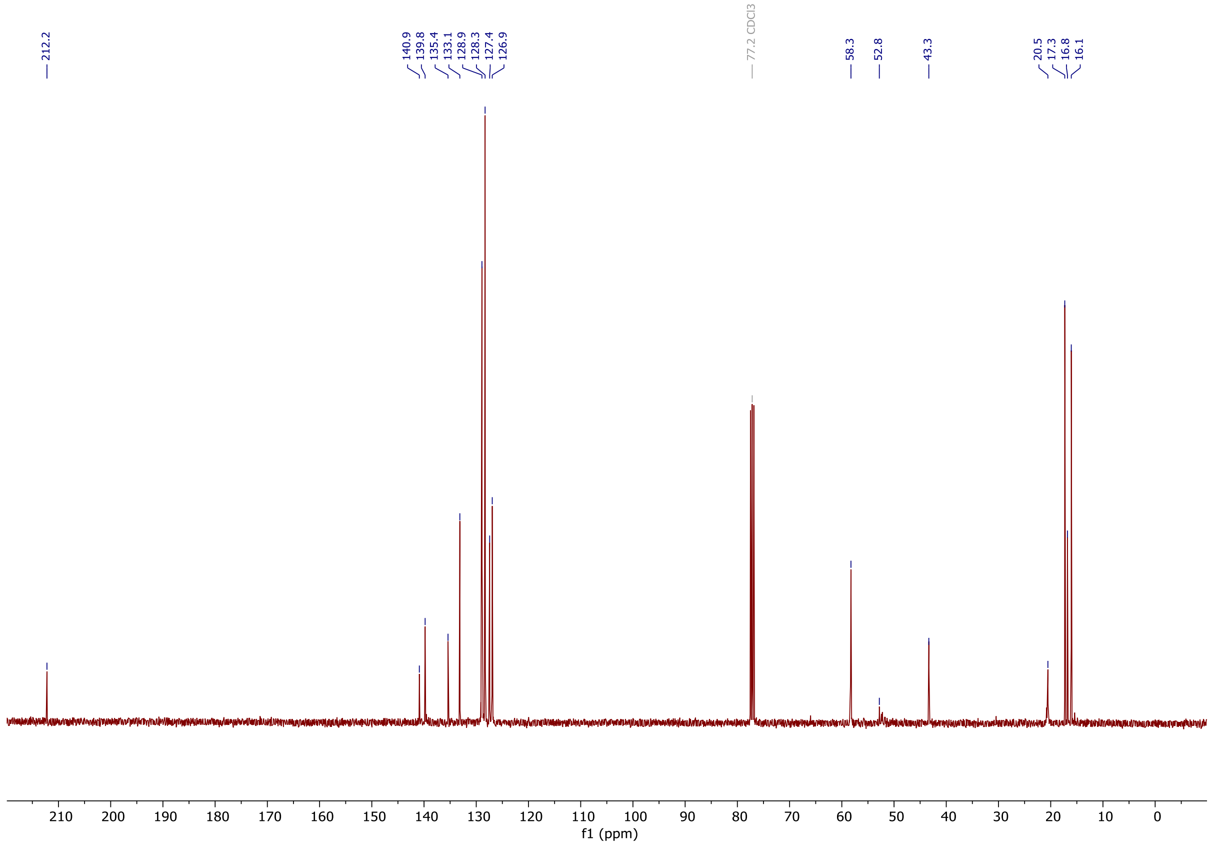
212.2
140.9
139.8
135.4
133.1
128.9
128.3
127.4
126.9
77.2 CDCl3
58.3
52.8
43.3
20.5
17.3
16.8
16.1
210 200 190 180 170 160 150 140 130 120 110 100 90 80 70 60 50 40 30 20 10 0
f1 (ppm)

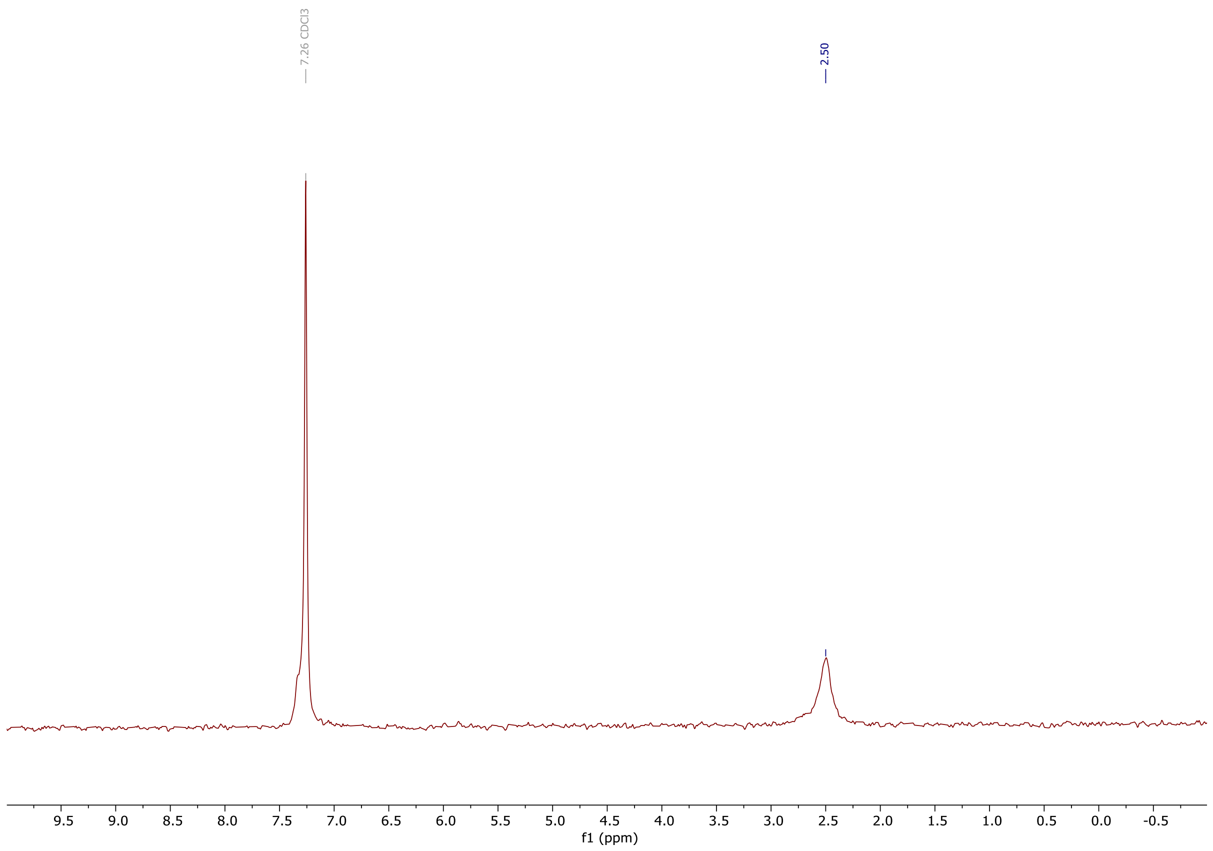
7.26 CDCl3
2.50
9.5 9.0 8.5 8.0 7.5 7.0 6.5 6.0 5.5 5.0 4.5 4.0 3.5 3.0 2.5 2.0 1.5 1.0 0.5 0.0 -0.5
f1 (ppm)

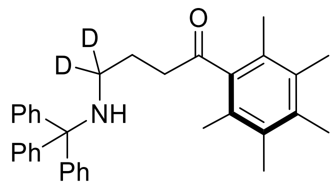
D
D
O
NH
Ph
Ph
Ph

7c

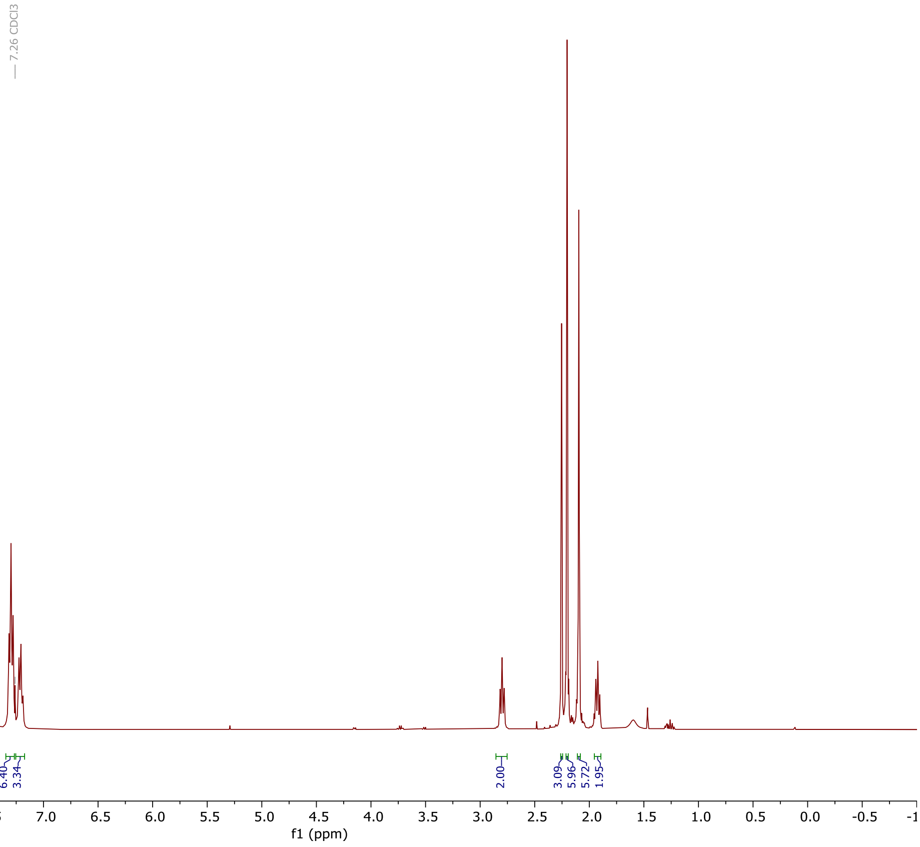
7.26 CDCl3
5.90
6.40
3.34
2.00
3.09
5.96
5.72
1.95
9.5 9.0 8.5 8.0 7.5 7.0 6.5 6.0 5.5 5.0 4.5 4.0 3.5 3.0 2.5 2.0 1.5 1.0 0.5 0.0 -0.5
f1 (ppm)

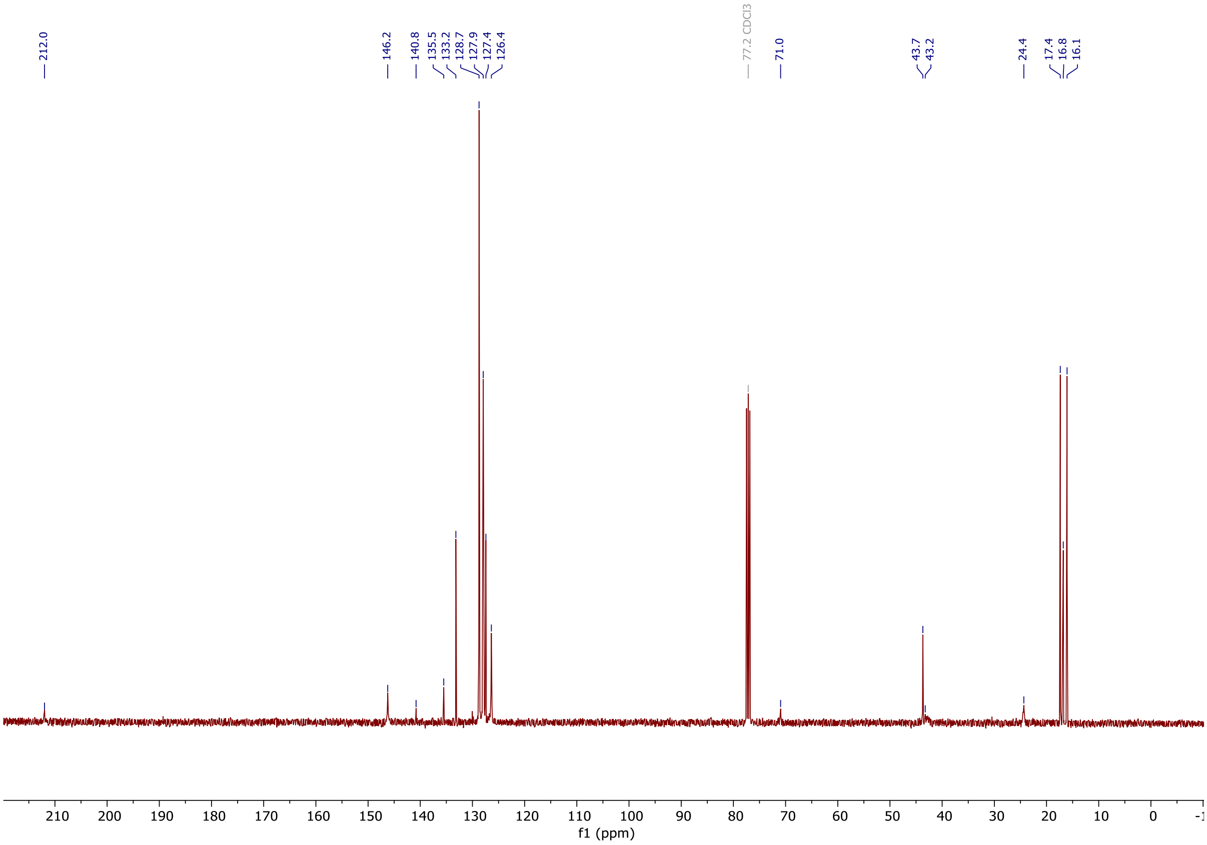
212.0
146.2
140.8
135.5
133.2
128.7
127.9
127.4
126.4
77.2 CDCl3
71.0
43.7
43.2
24.4
17.4
16.8
16.1
210 200 190 180 170 160 150 140 130 120 110 100 90 80 70 60 50 40 30 20 10 0
f1 (ppm)

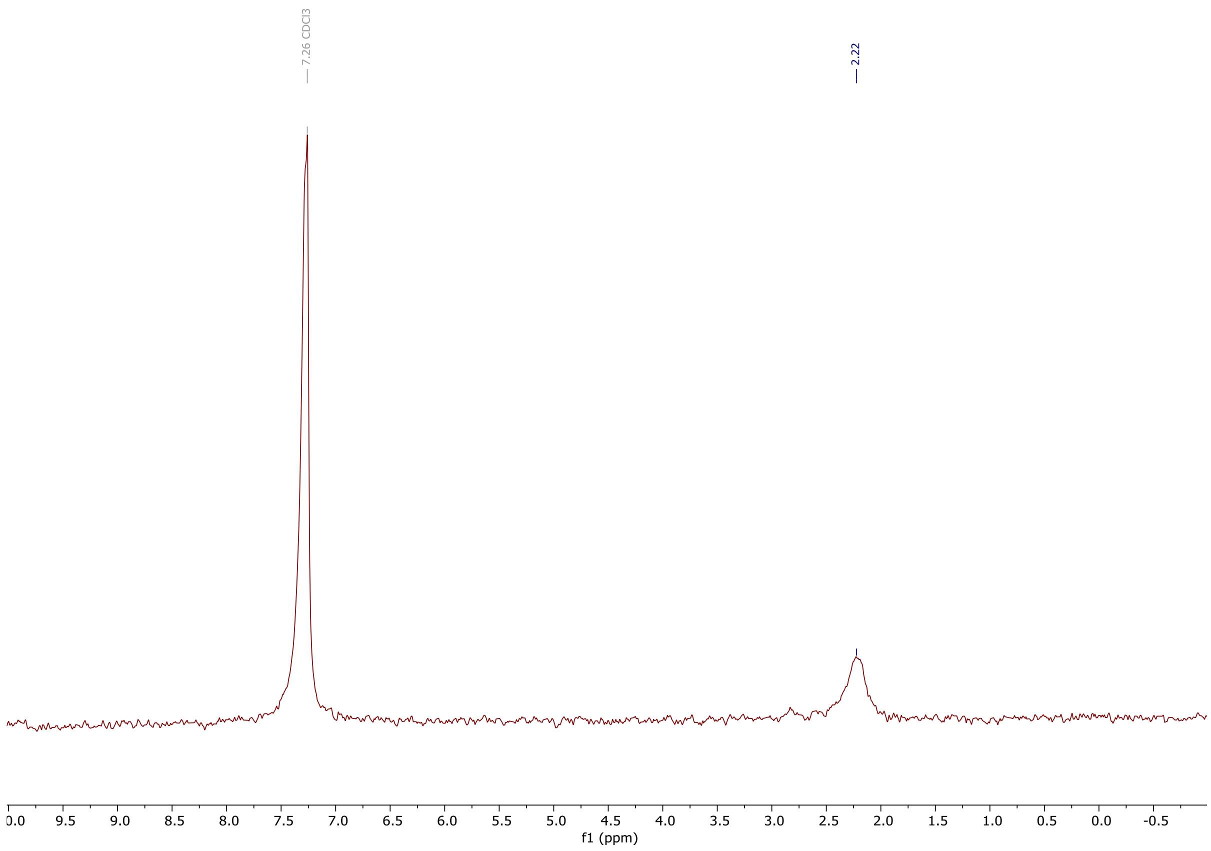

7.26 CDCl3
2.22
0.0
9.5
9.0
8.5
8.0
7.5
7.0
6.5
6.0
5.5
5.0
4.5
4.0
3.5
3.0
2.5
2.0
1.5
1.0
0.5
0.0
-0.5
f1 (ppm)

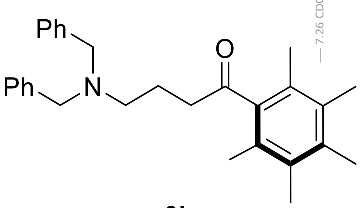
Ph
Ph
N
O

3b

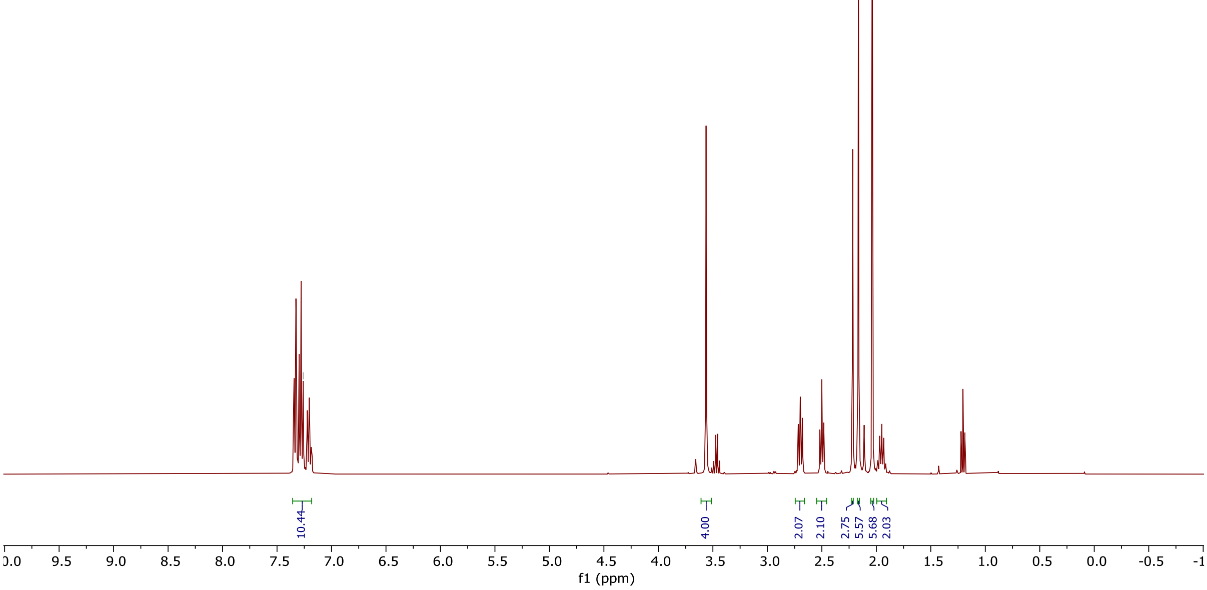
7.26 CDCl3
10.44
4.00
2.07
2.10
2.75
5.57
5.68
2.03
0.0 9.5 9.0 8.5 8.0 7.5 7.0 6.5 6.0 5.5 5.0 4.5 4.0 3.5 3.0 2.5 2.0 1.5 1.0 0.5 0.0 -0.5 -1
f1 (ppm)

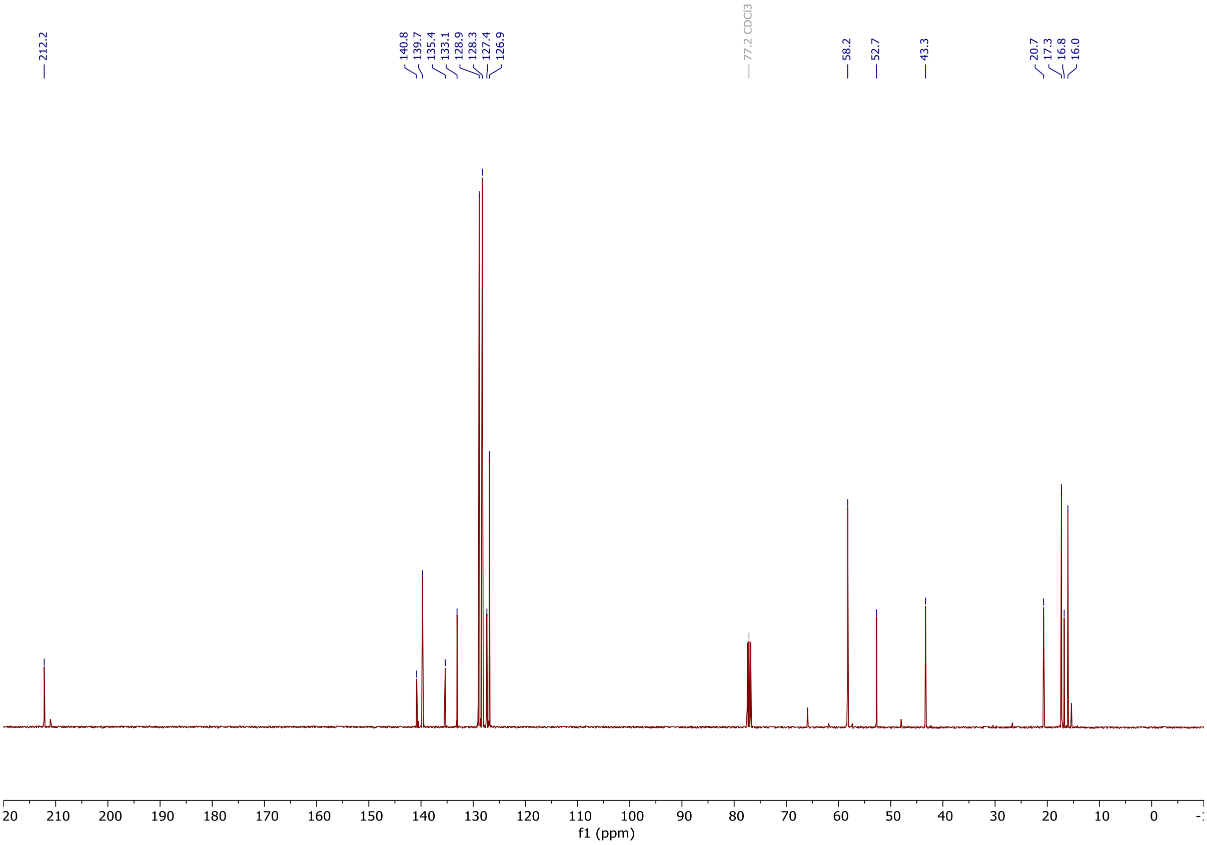
212.2
140.8
139.7
135.4
133.1
128.9
128.3
127.4
126.9
77.2 CDCl3
58.2
52.7
43.3
20.7
17.3
16.8
16.0
20 210 200 190 180 170 160 150 140 130 120 110 100 90 80 70 60 50 40 30 20 10 0
f1 (ppm)

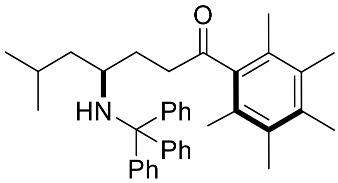
O
HN
Ph
Ph
Ph

3d

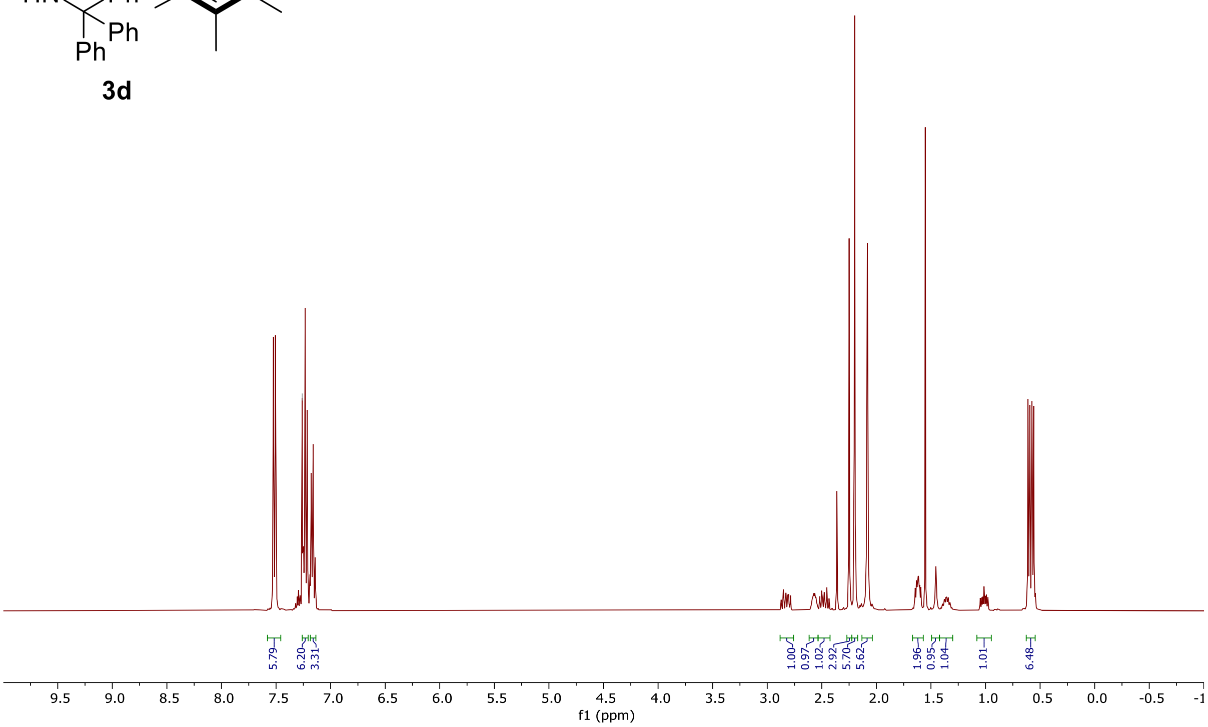
7.26 CDCl3
5.79
6.20
3.31
1.00
0.97
1.02
2.92
5.70
5.62
1.96
0.95
1.04
1.01
6.48
f1 (ppm)

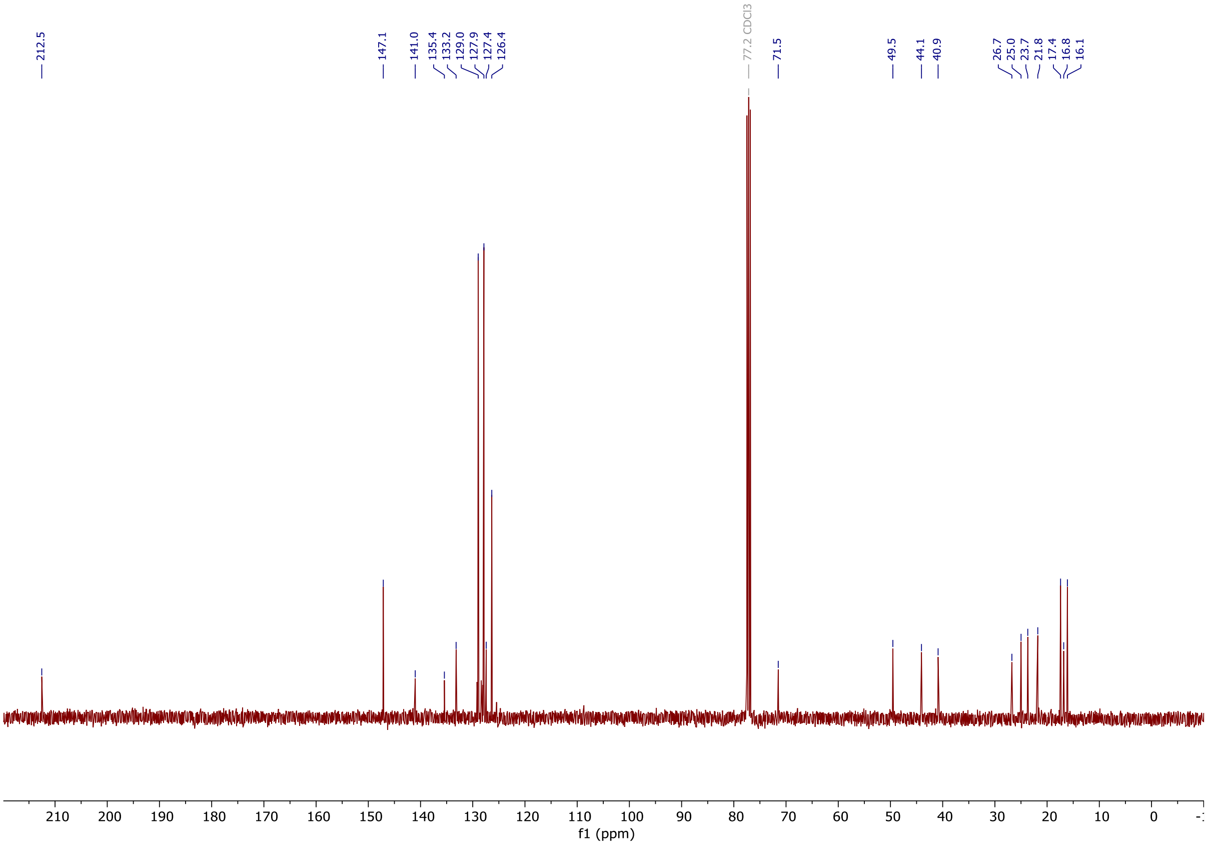
212.5
147.1
141.0
135.4
133.2
129.0
127.9
127.4
126.4
77.2 CDCl3
71.5
49.5
44.1
40.9
26.7
25.0
23.7
21.8
17.4
16.8
16.1
f1 (ppm)

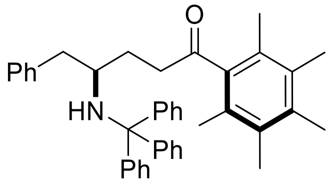
O
Ph
HN
Ph
Ph
Ph

3e

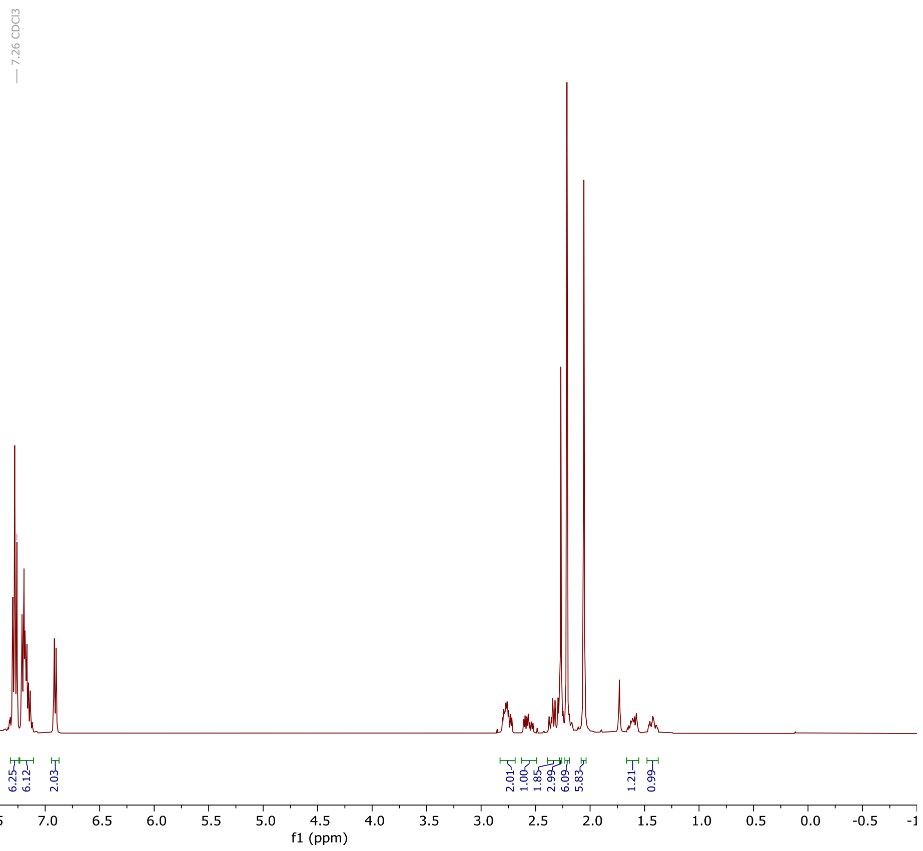
7.26 CDCl3
5.93
6.25
6.12
2.03
2.01
1.00
1.85
2.99
6.09
5.83
1.21
0.99
f1 (ppm)

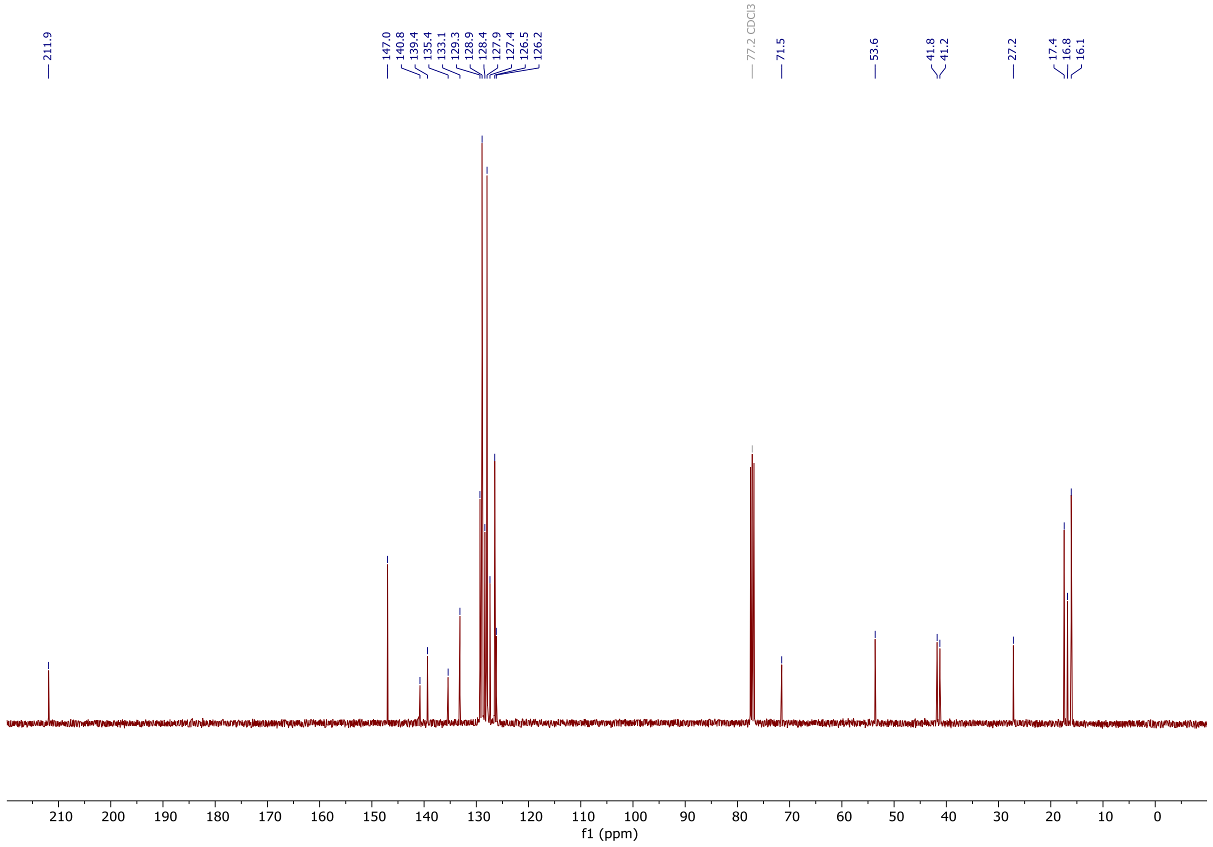
211.9
147.0
140.8
139.4
135.4
133.1
129.3
128.9
128.4
127.9
127.4
126.5
126.2
77.2 CDCl3
71.5
53.6
41.8
41.2
27.2
17.4
16.8
16.1
f1 (ppm)

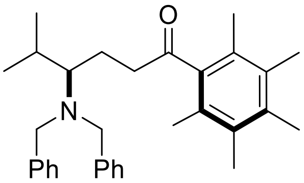

3f

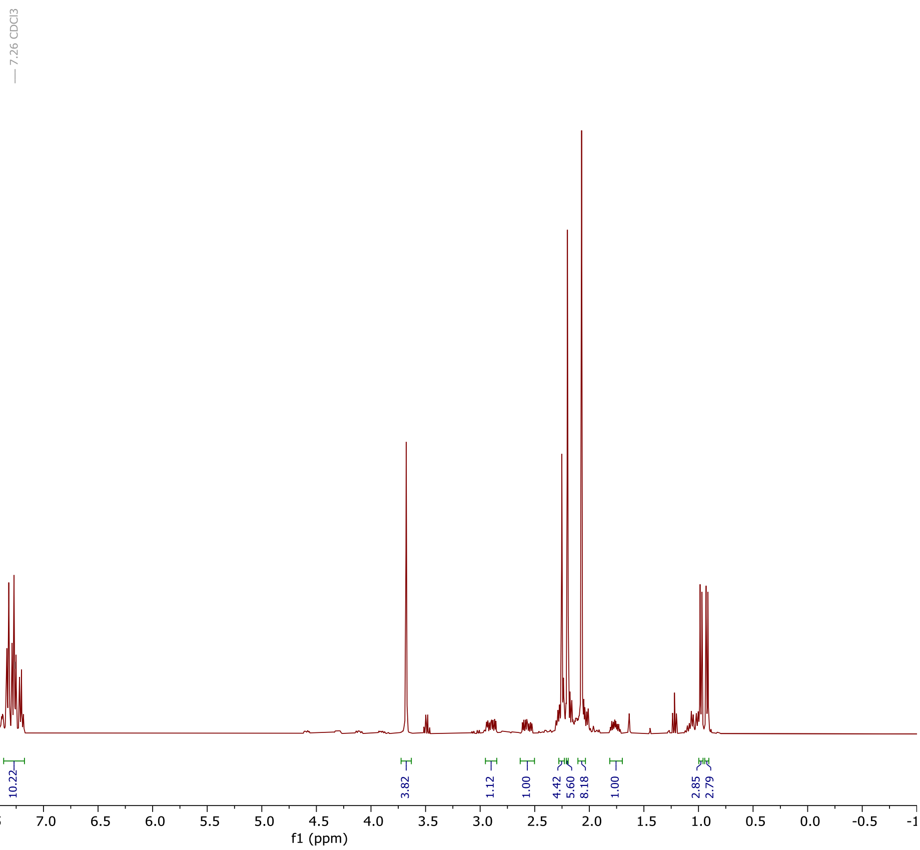

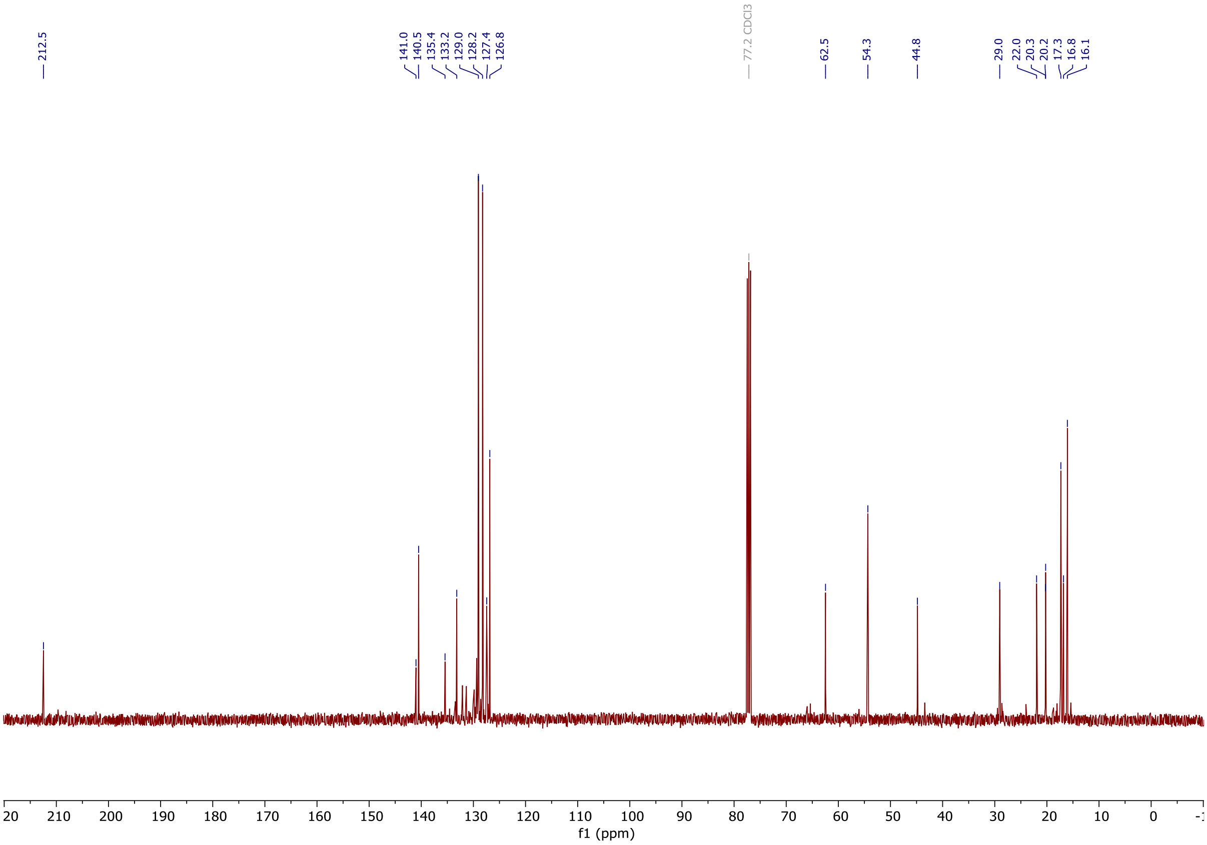

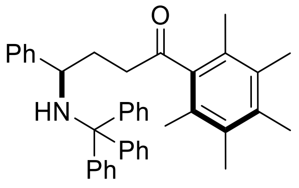
O
Ph
HN
Ph
Ph
Ph

3g

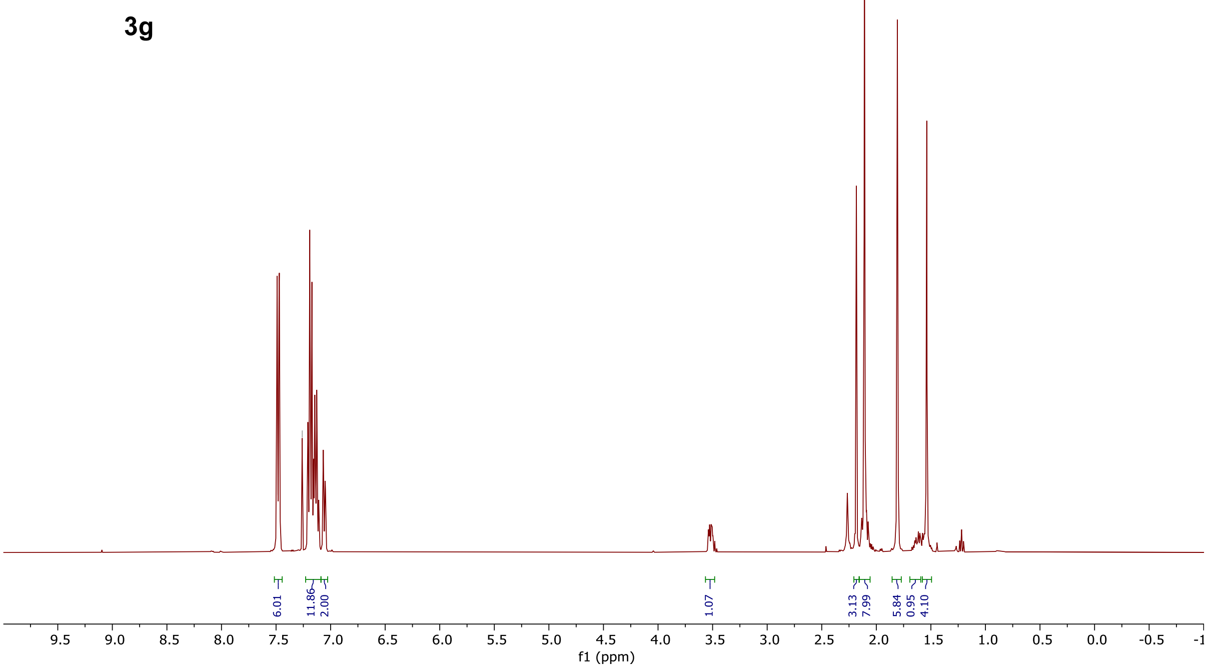
7.26 CDCl3
6.01
11.86
2.00
1.07
3.13
7.99
5.84
0.95
4.10
f1 (ppm)

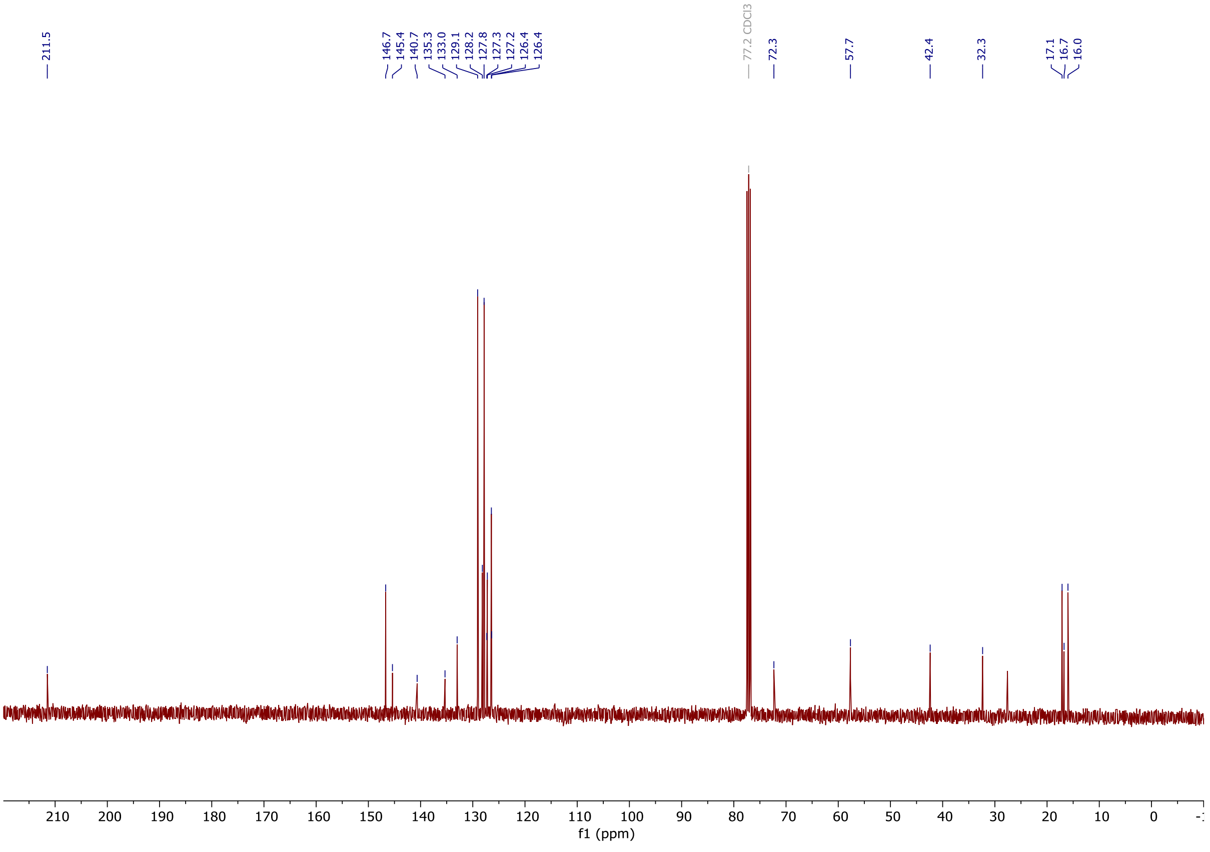
211.5
146.7
145.4
140.7
135.3
133.0
129.1
128.2
127.8
127.3
127.2
126.4
126.4
77.2 CDCl3
72.3
57.7
42.4
32.3
17.1
16.7
16.0
f1 (ppm)

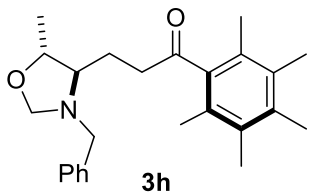
O
O
N
Ph

3h

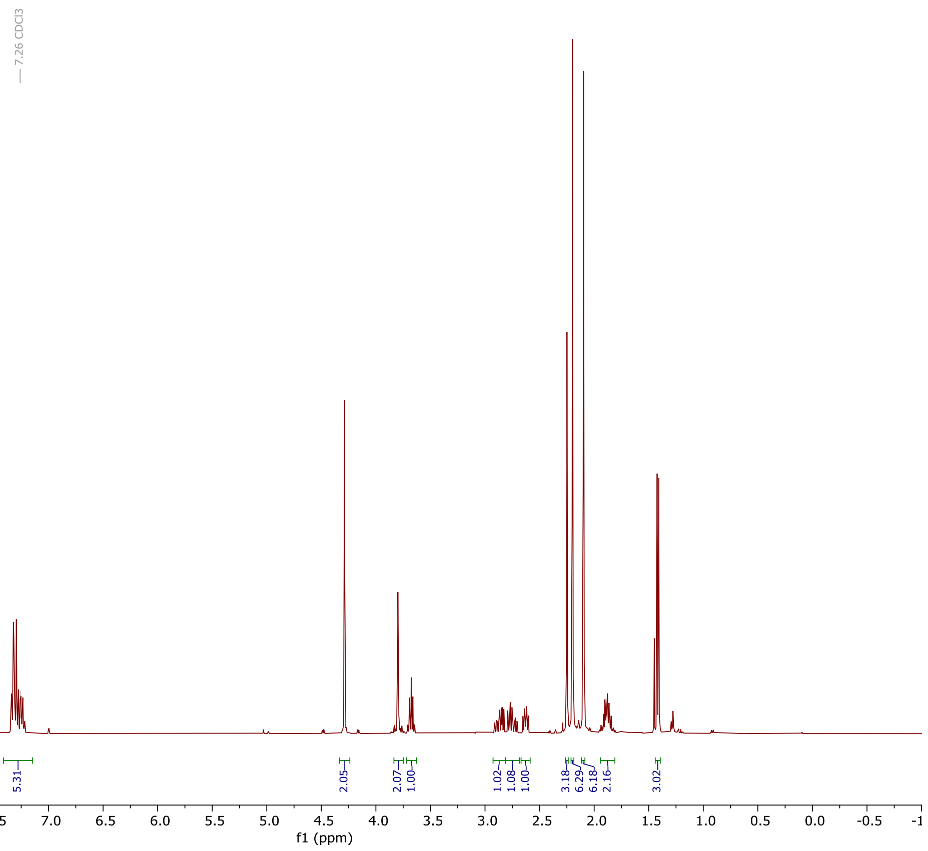
7.26 CDCl3
5.31
2.05
2.07
1.00
1.02
1.08
1.00
3.18
6.29
6.18
2.16
3.02
f1 (ppm)

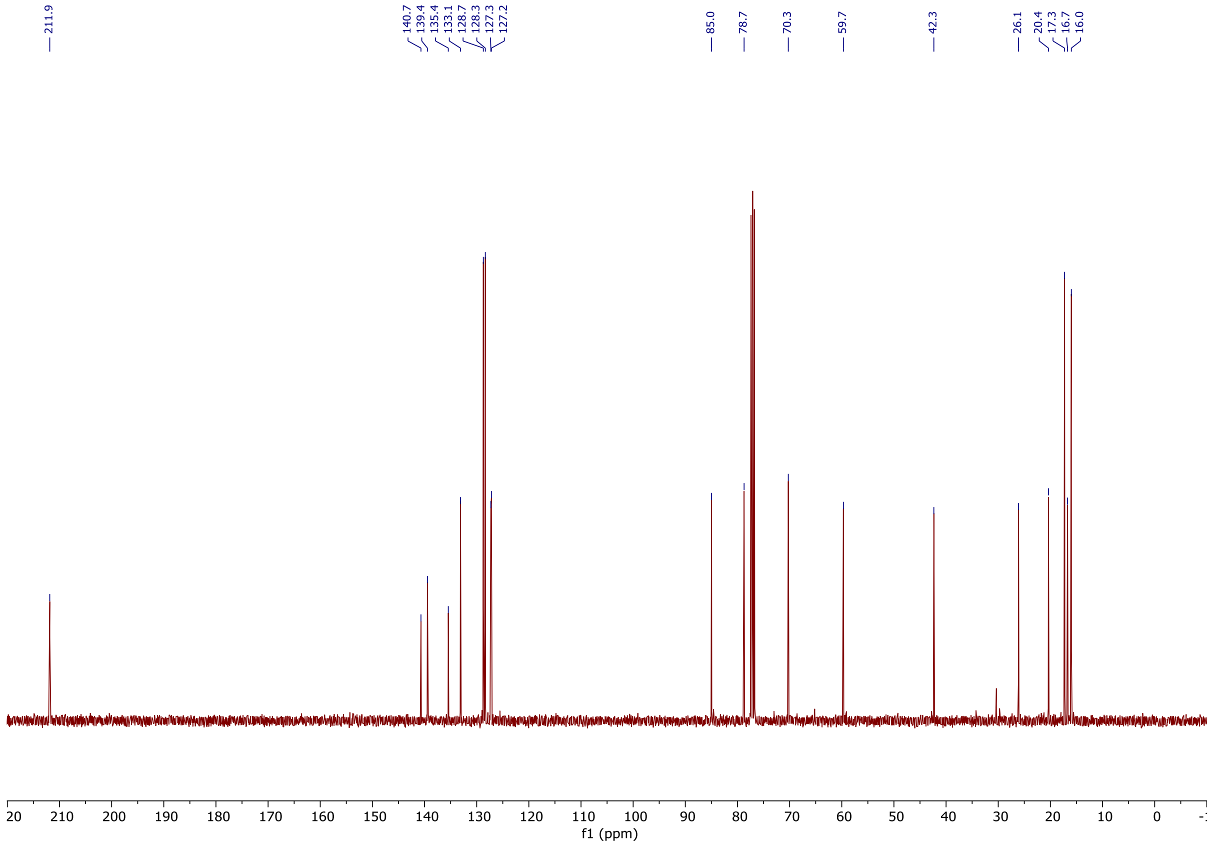
211.9
140.7
139.4
135.4
133.1
128.7
128.3
127.3
127.2
85.0
78.7
70.3
59.7
42.3
26.1
20.4
17.3
16.7
16.0
f1 (ppm)

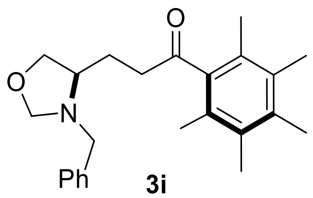
O
O
N
Ph

3i

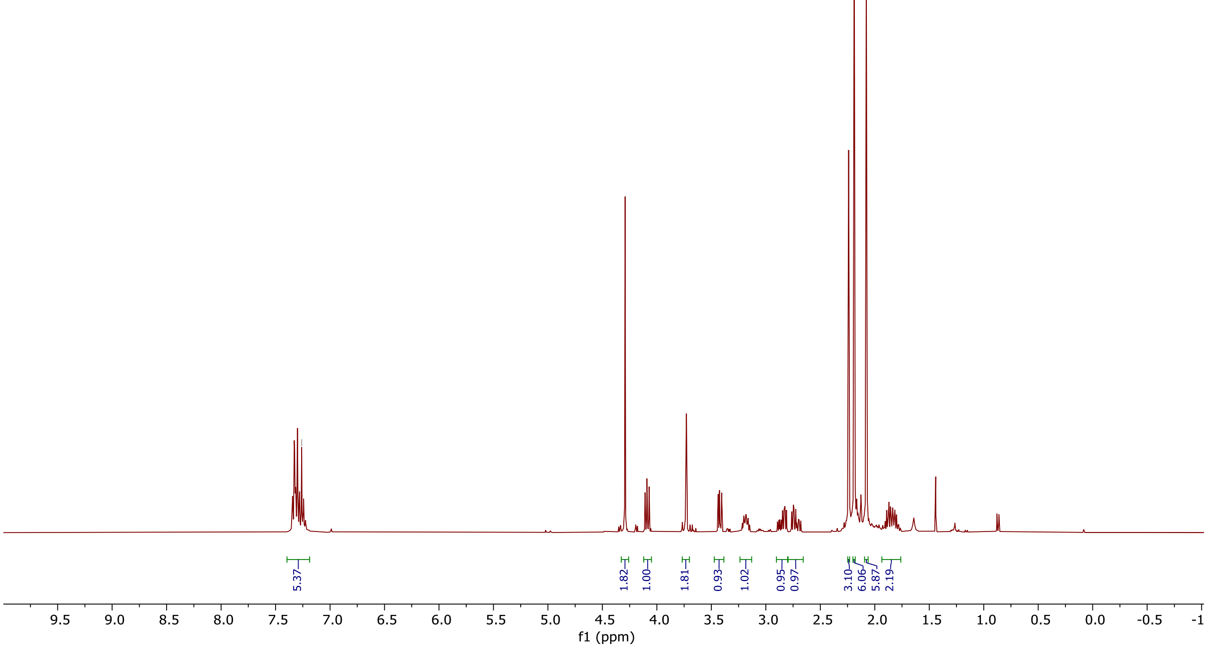
7.26 CDCl3
5.37
1.82
1.00
1.81
0.93
1.02
0.95
0.97
3.10
6.06
5.87
2.19
f1 (ppm)

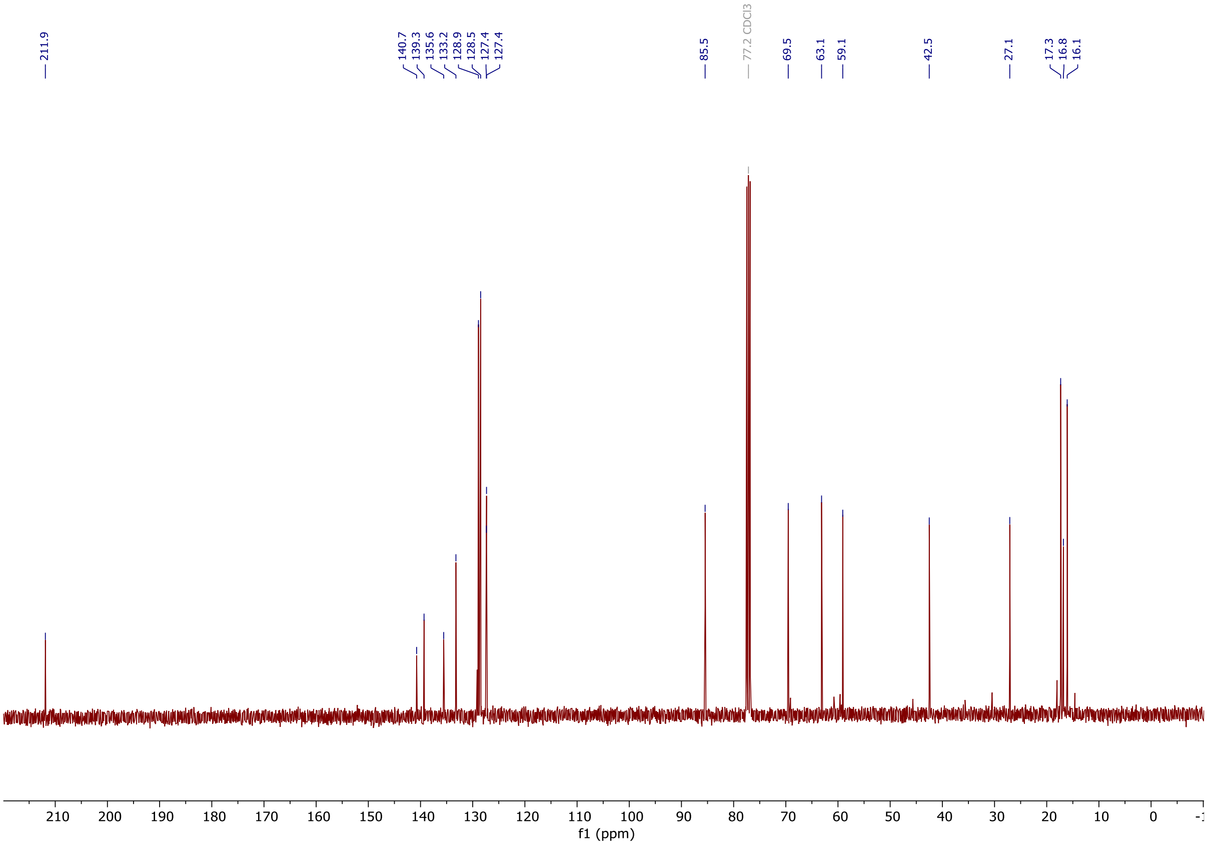
211.9
140.7
139.3
135.6
133.2
128.9
128.5
127.4
127.4
85.5
77.2 CDCl3
69.5
63.1
59.1
42.5
27.1
17.3
16.8
16.1
f1 (ppm)

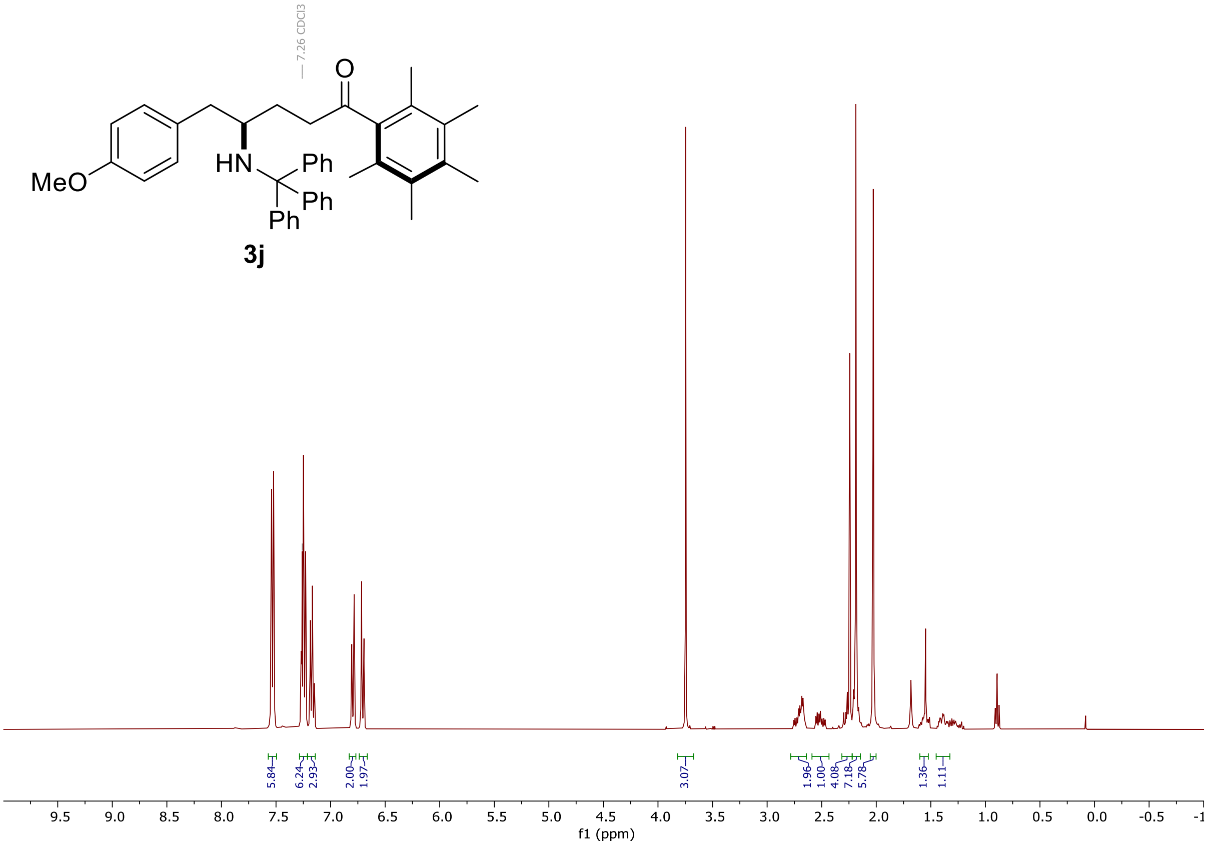
MeO
HN
Ph
Ph
Ph
O
3j
7.26 CDCl3
5.84
6.24
2.93
2.00
1.97
3.07
1.96
1.00
4.08
7.18
5.78
1.36
1.11
9.5 9.0 8.5 8.0 7.5 7.0 6.5 6.0 5.5 5.0 4.5 4.0 3.5 3.0 2.5 2.0 1.5 1.0 0.5 0.0 -0.5
f1 (ppm)

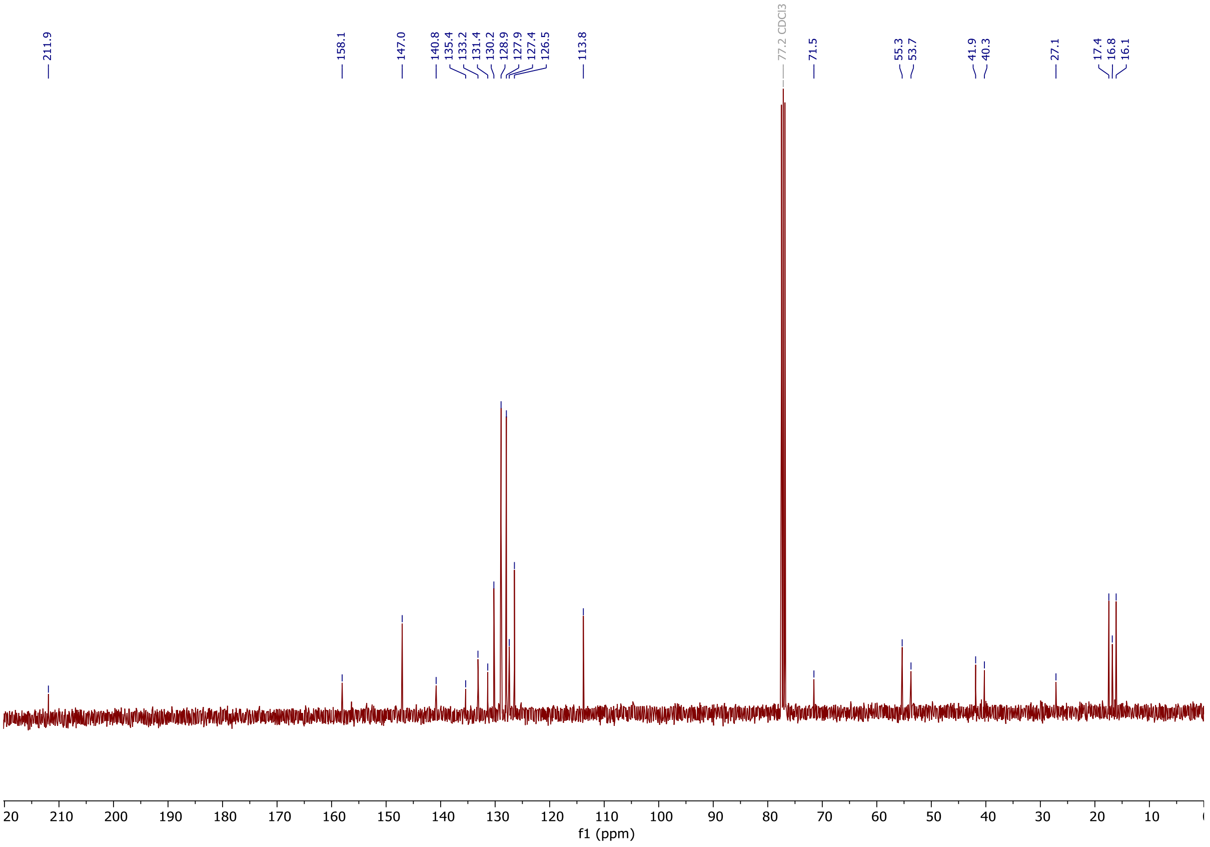
211.9
158.1
147.0
140.8
135.4
133.2
131.4
130.2
128.9
127.9
127.4
126.5
113.8
77.2 CDCl3
71.5
55.3
53.7
41.9
40.3
27.1
17.4
16.8
16.1
210 200 190 180 170 160 150 140 130 120 110 100 90 80 70 60 50 40 30 20 10
f1 (ppm)

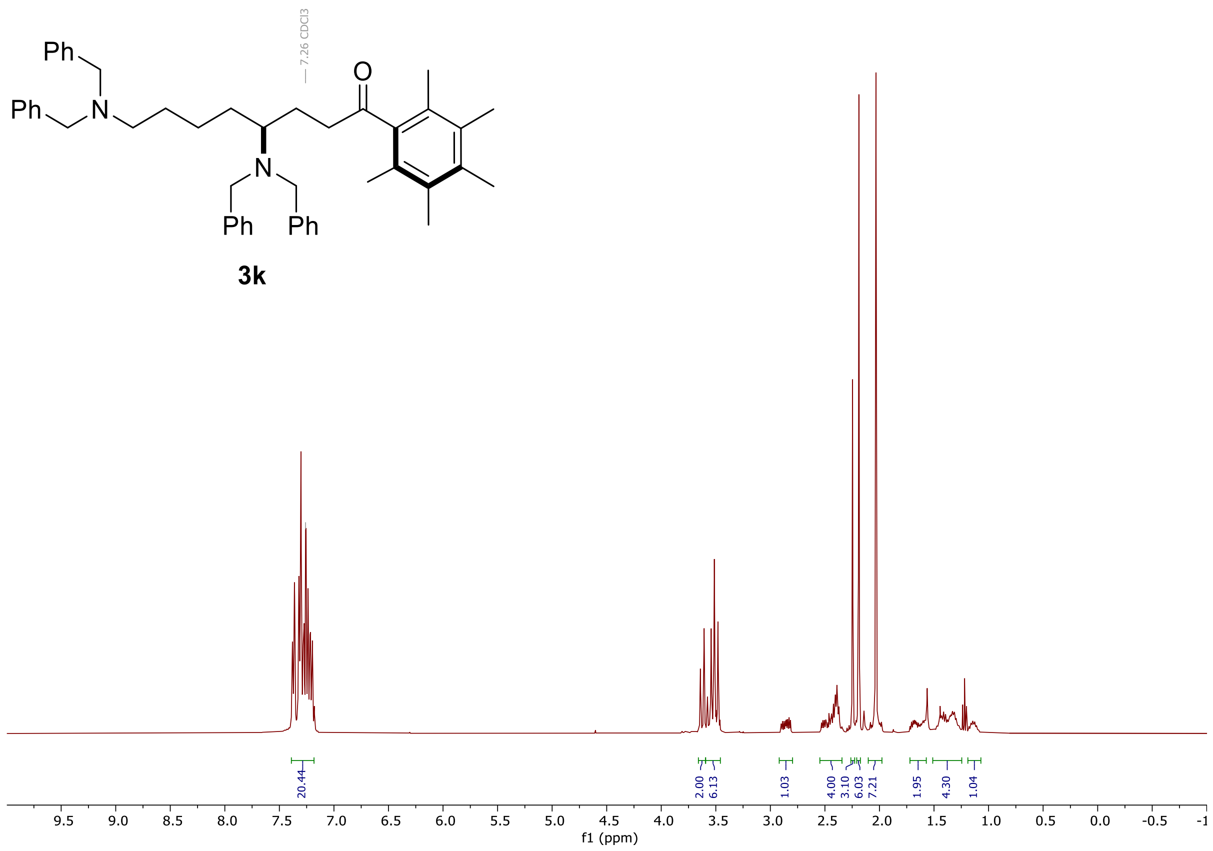

Ph
Ph
N
O
N
Ph
Ph
3k
7.26 CDCl3
20.44
2.00
6.13
1.03
4.00
3.10
6.03
7.21
1.95
4.30
1.04
9.5 9.0 8.5 8.0 7.5 7.0 6.5 6.0 5.5 5.0 4.5 4.0 3.5 3.0 2.5 2.0 1.5 1.0 0.5 0.0 -0.5
f1 (ppm)

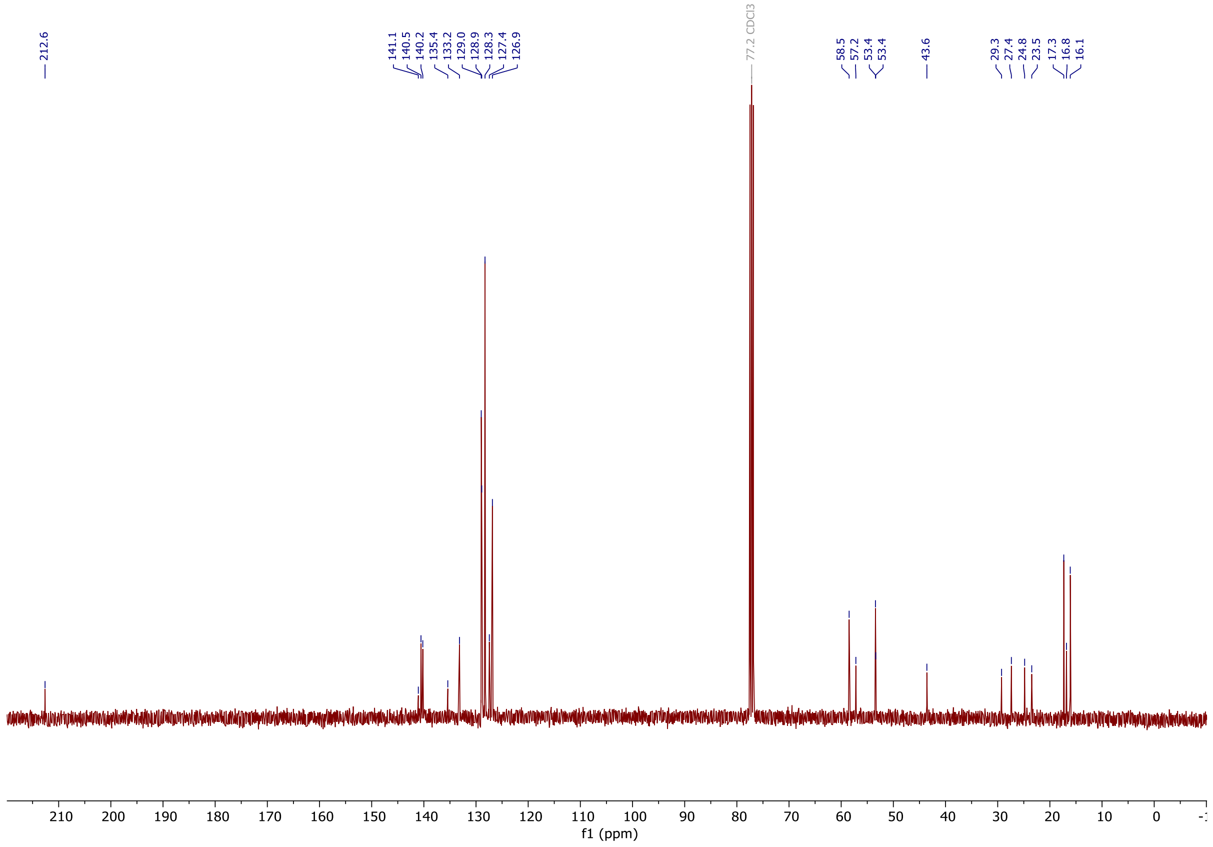

212.6
141.1
140.5
140.2
135.4
133.2
129.0
128.9
128.3
127.4
126.9
77.2 CDCl3
58.5
57.2
53.4
53.4
43.6
29.3
27.4
24.8
23.5
17.3
16.8
16.1
210 200 190 180 170 160 150 140 130 120 110 100 90 80 70 60 50 40 30 20 10 0
f1 (ppm)

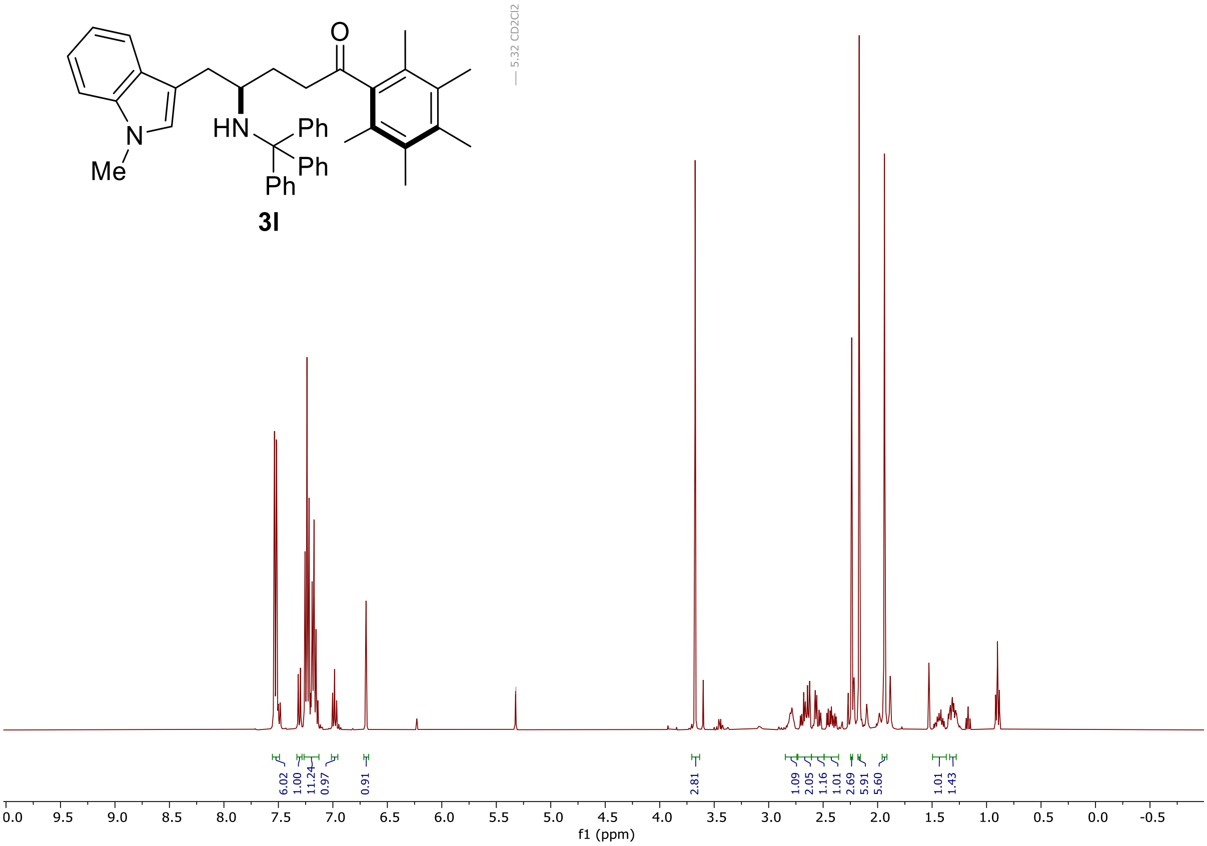
O
HN
Ph
Ph
Ph
N
Me
3l
5.32 CD2Cl2
6.02
1.00
11.24
0.97
0.91
2.81
1.09
2.05
1.16
1.01
2.69
5.91
5.60
1.01
1.43
0.0 9.5 9.0 8.5 8.0 7.5 7.0 6.5 6.0 5.5 5.0 4.5 4.0 3.5 3.0 2.5 2.0 1.5 1.0 0.5 0.0 -0.5
f1 (ppm)

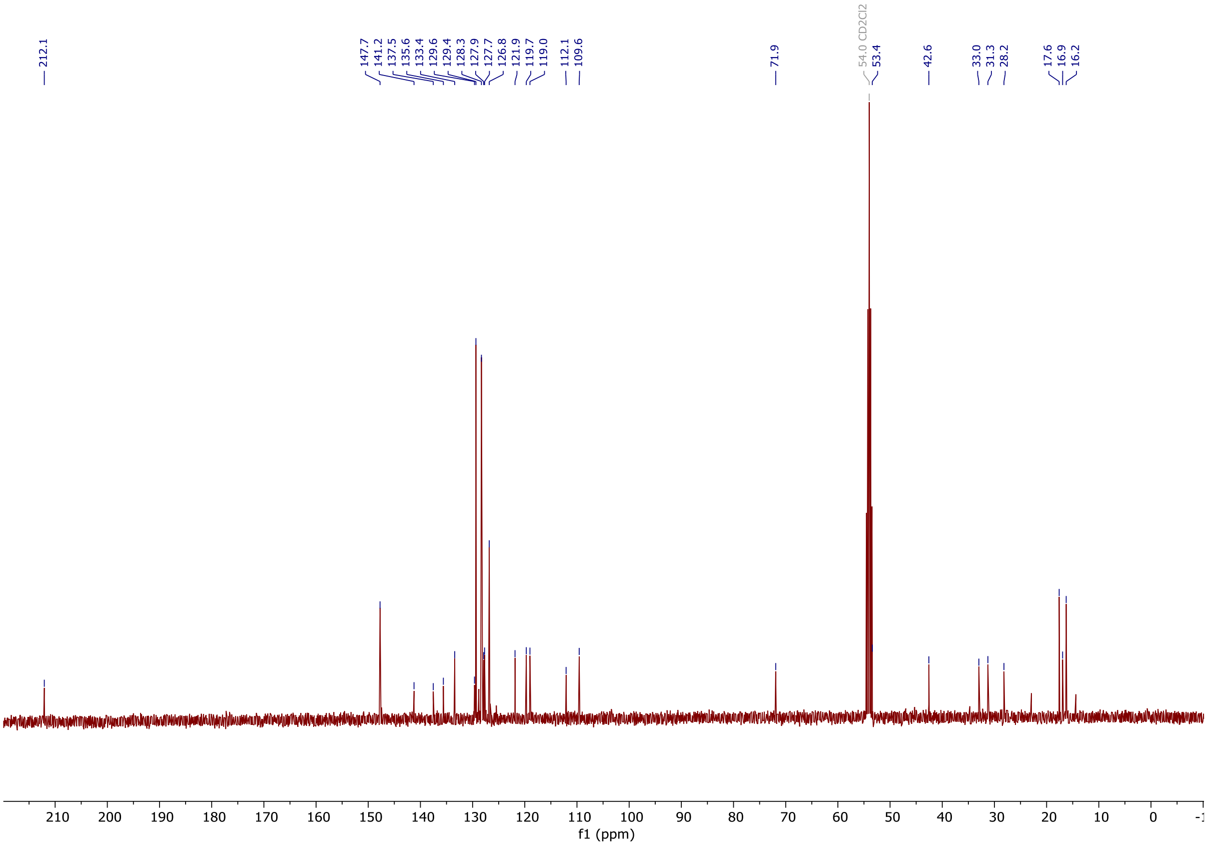
212.1
147.7
141.2
137.5
135.6
133.4
129.6
129.4
128.3
127.9
127.7
126.8
121.9
119.7
119.0
112.1
109.6
71.9
54.0 CD2Cl2
53.4
42.6
33.0
31.3
28.2
17.6
16.9
16.2
210 200 190 180 170 160 150 140 130 120 110 100 90 80 70 60 50 40 30 20 10 0
f1 (ppm)

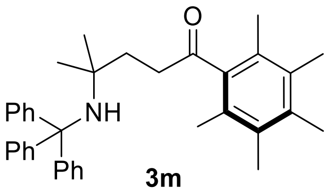
O
NH
Ph
Ph
Ph

3m

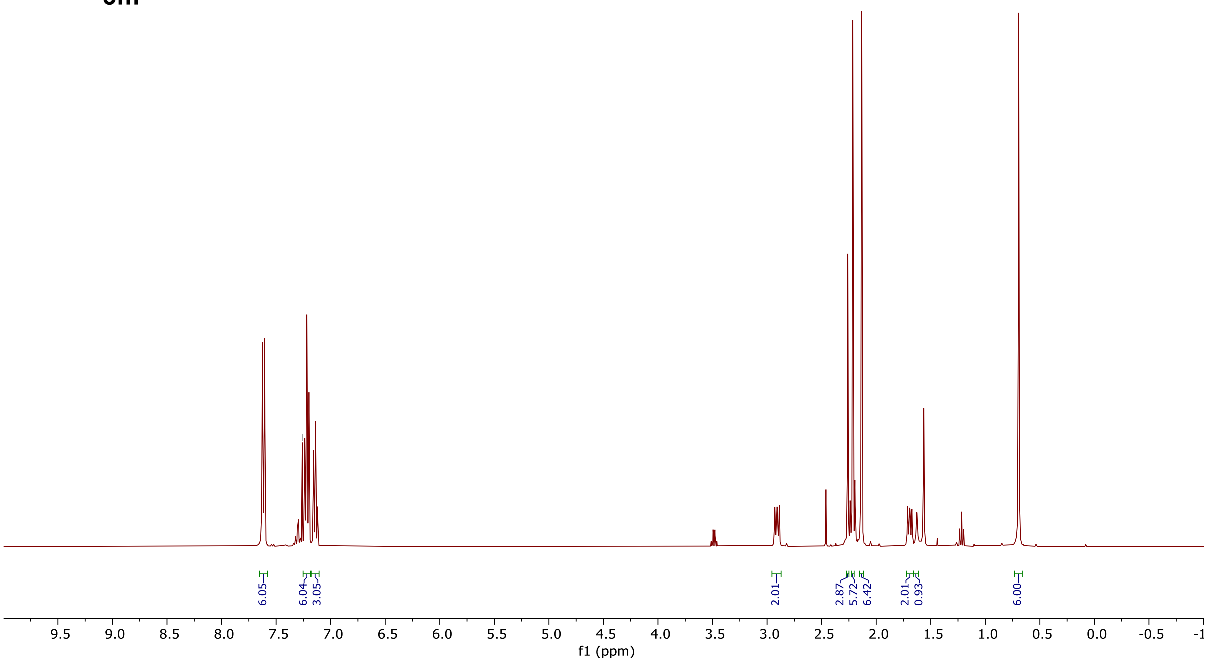
7.26 CDCl3
6.05
6.04
3.05
2.01
2.87
5.72
6.42
2.01
0.93
6.00
9.5 9.0 8.5 8.0 7.5 7.0 6.5 6.0 5.5 5.0 4.5 4.0 3.5 3.0 2.5 2.0 1.5 1.0 0.5 0.0 -0.5 -1
f1 (ppm)

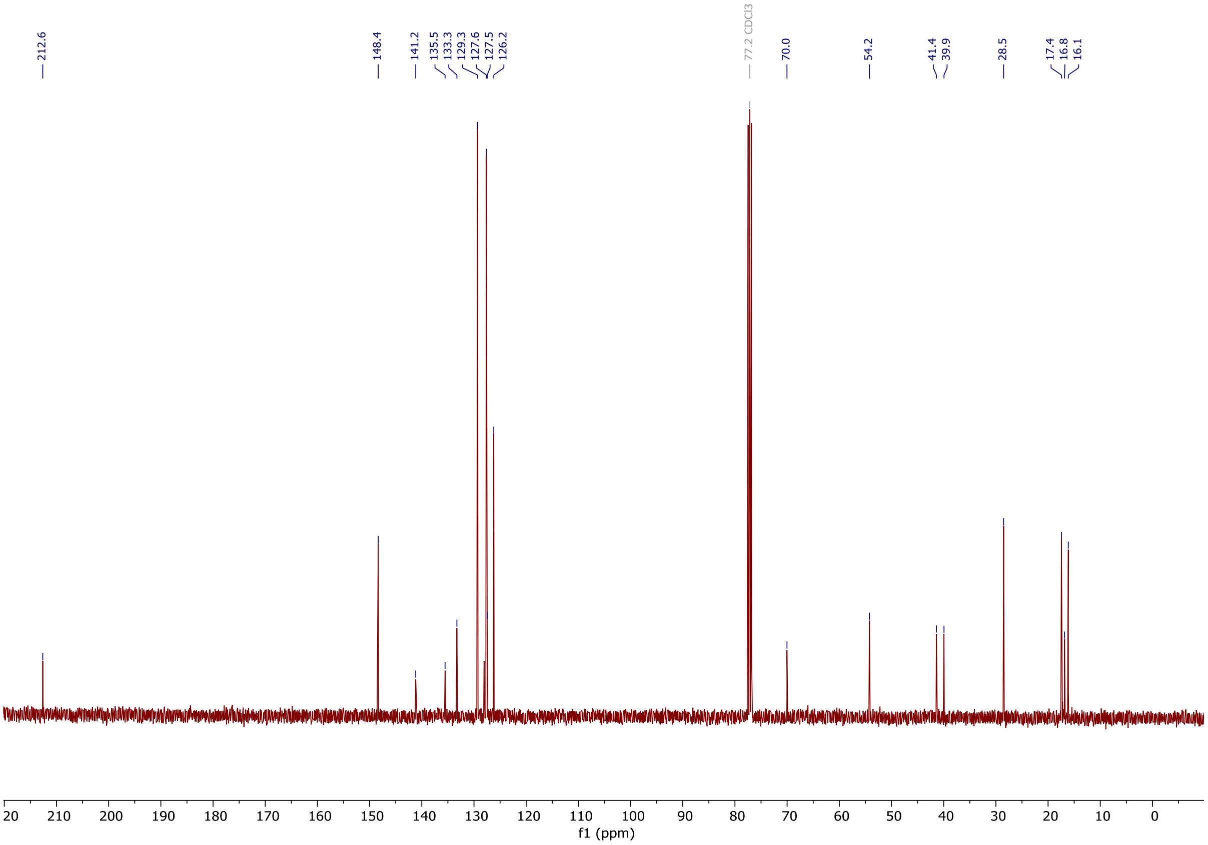
212.6
148.4
141.2
135.5
133.3
129.3
127.6
127.5
126.2
77.2 CDCl3
70.0
54.2
41.4
39.9
28.5
17.4
16.8
16.1
20 210 200 190 180 170 160 150 140 130 120 110 100 90 80 70 60 50 40 30 20 10 0
f1 (ppm)

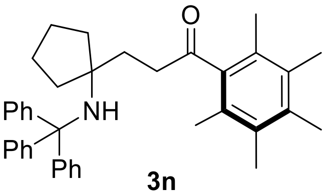
Ph
NH
Ph
Ph
O

3n

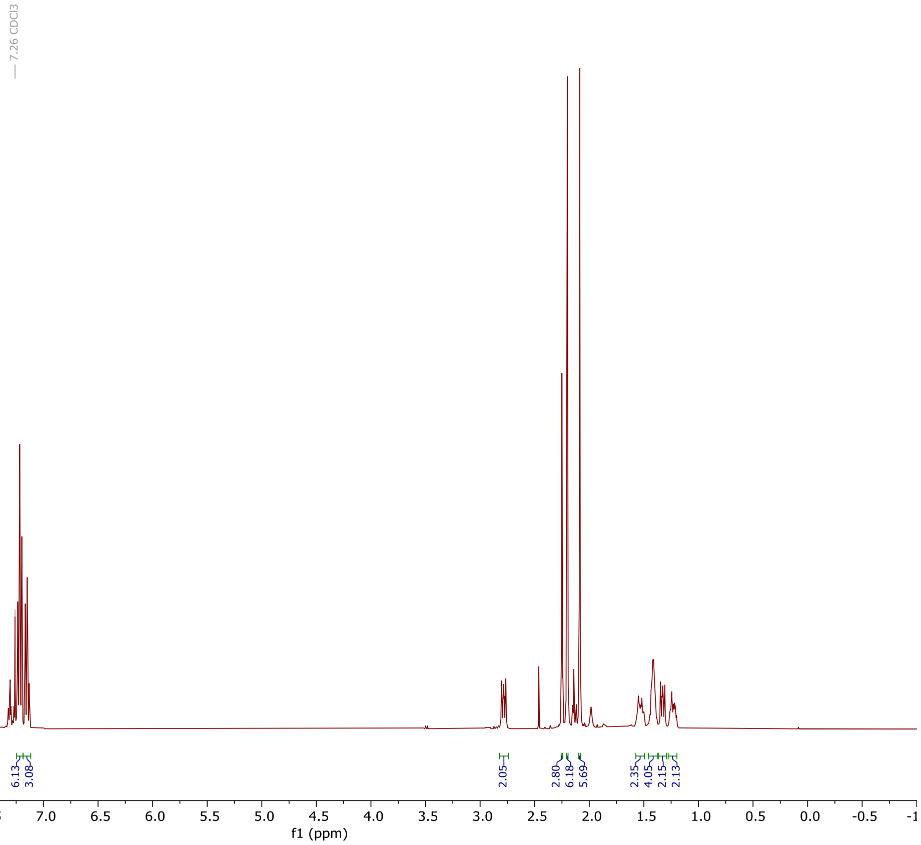
7.26 CDCl3
6.00
6.13
3.08
2.05
2.80
6.18
5.69
2.35
4.05
2.15
2.13
f1 (ppm)

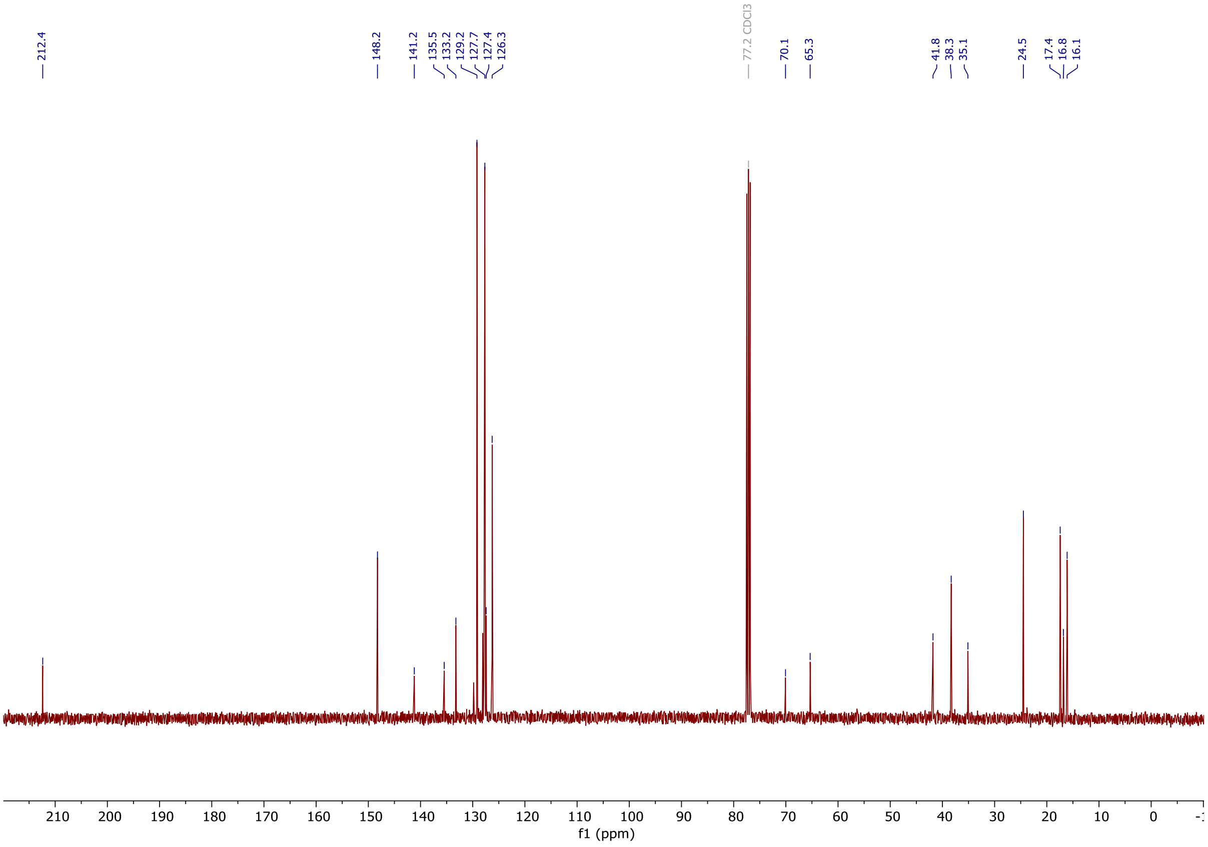
212.4
148.2
141.2
135.5
133.2
129.2
127.7
127.4
126.3
77.2 CDCl3
70.1
65.3
41.8
38.3
35.1
24.5
17.4
16.8
16.1
f1 (ppm)

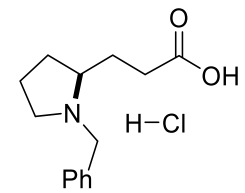
O
OH
N
H−Cl
Ph

9a

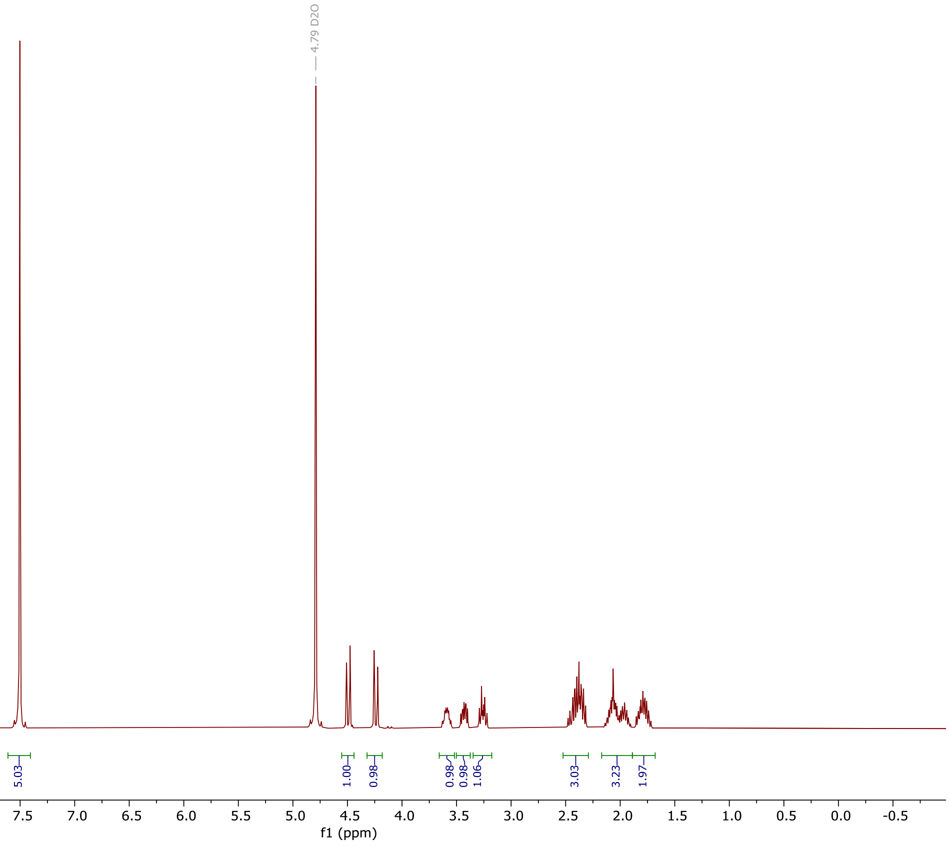
4.79 D2O
5.03
1.00
0.98
0.98
0.98
1.06
3.03
3.23
1.97
10.0 9.5 9.0 8.5 8.0 7.5 7.0 6.5 6.0 5.5 5.0 4.5 4.0 3.5 3.0 2.5 2.0 1.5 1.0 0.5 0.0 -0.5
f1 (ppm)

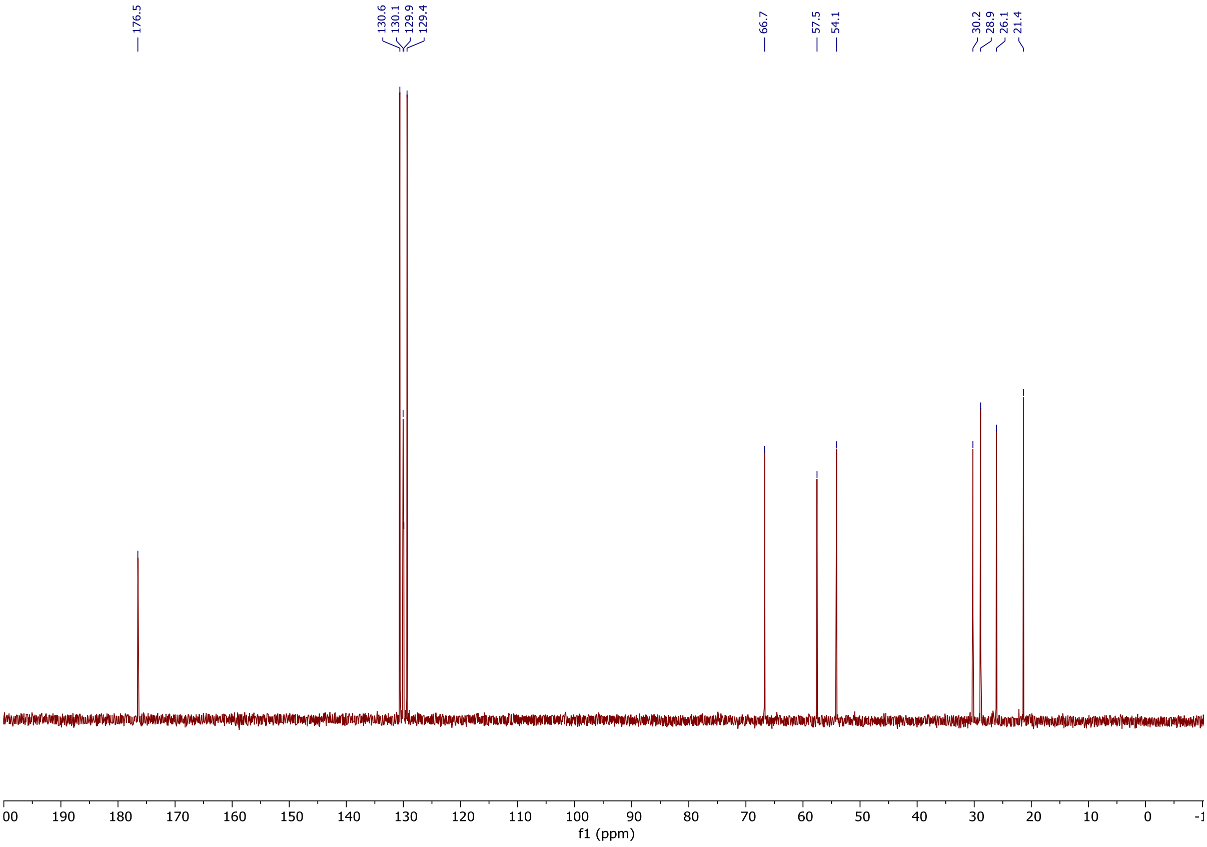
176.5
130.6
130.1
129.9
129.4
66.7
57.5
54.1
30.2
28.9
26.1
21.4
200 190 180 170 160 150 140 130 120 110 100 90 80 70 60 50 40 30 20 10 0 -10
f1 (ppm)

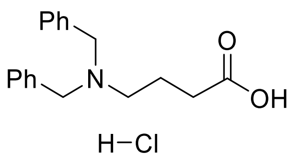

9b

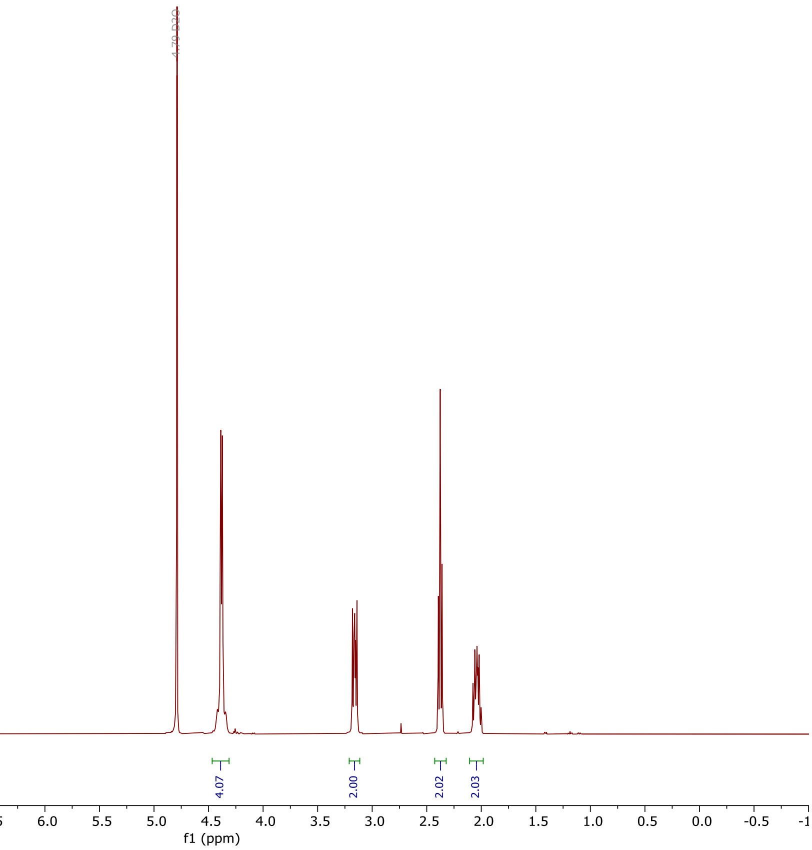

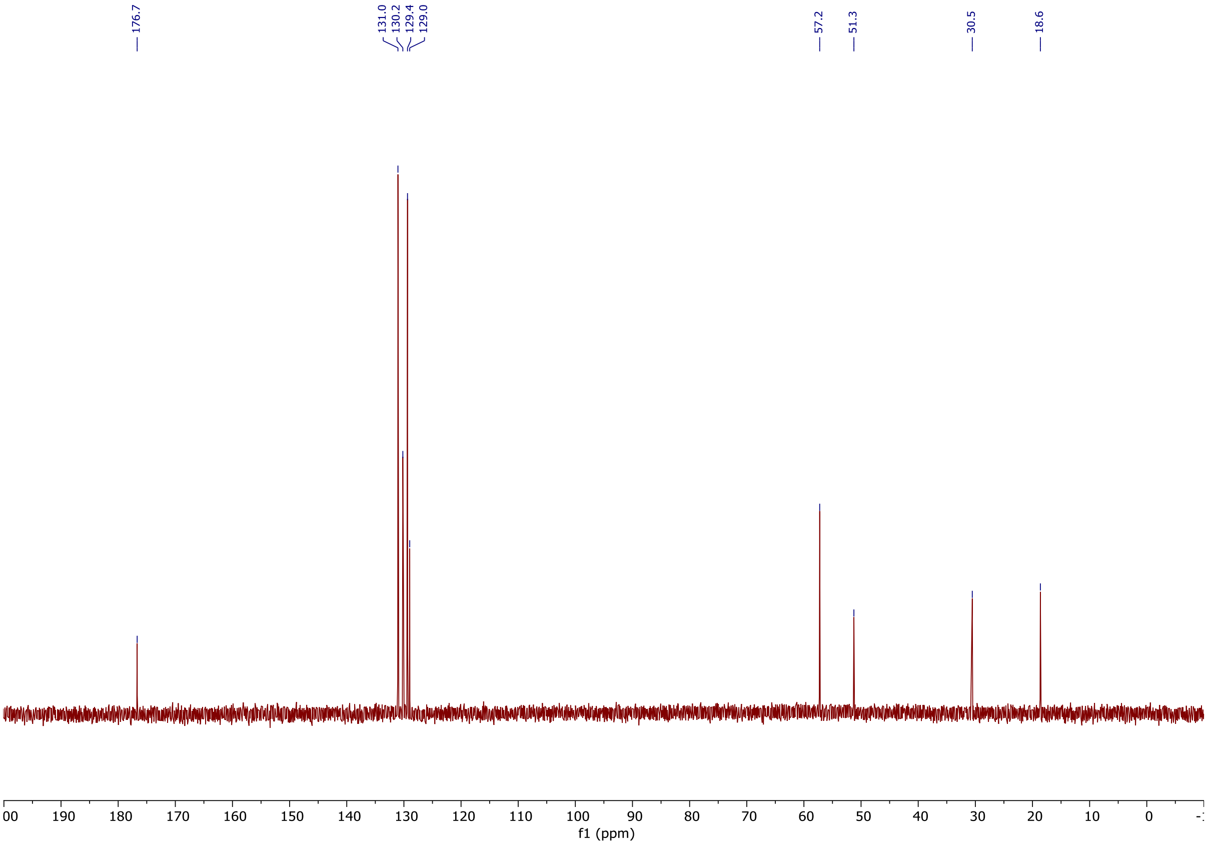

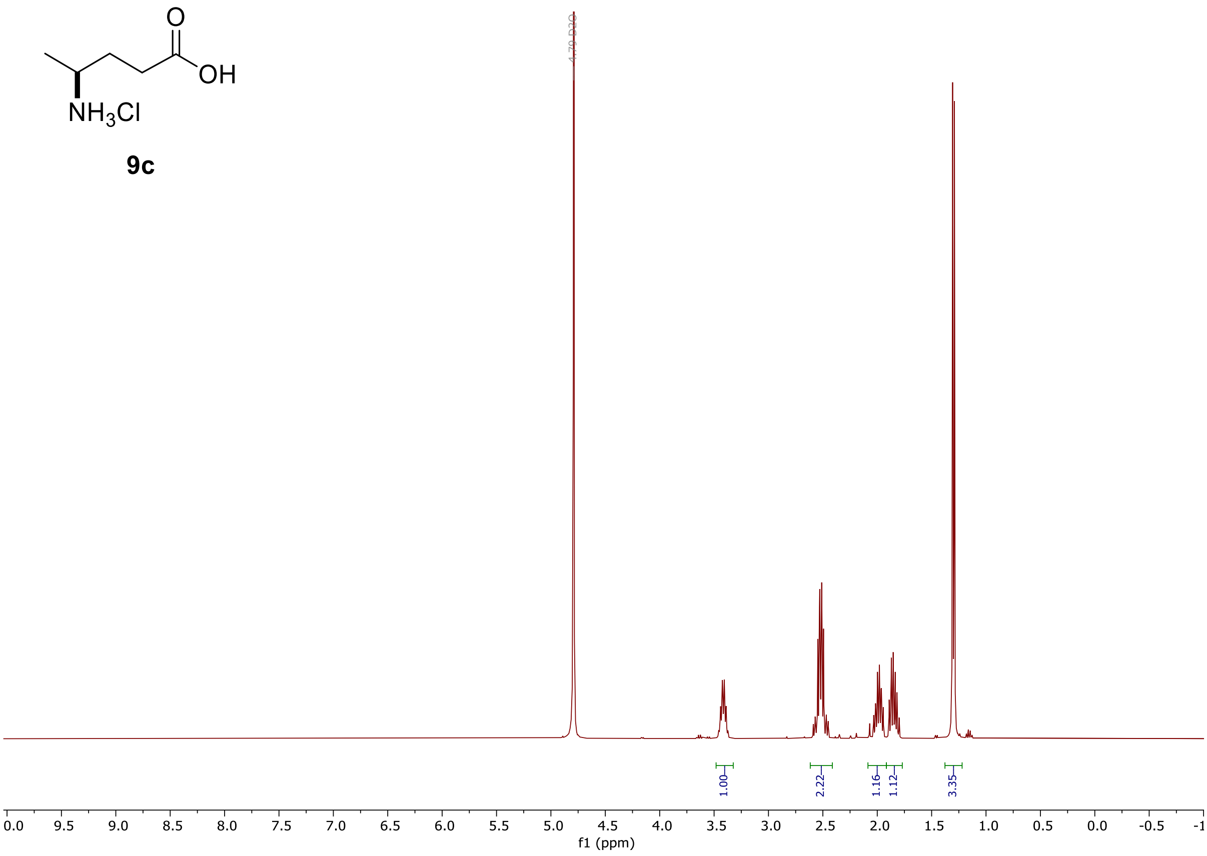
O
OH
NH3Cl
9c
4.79 D2O
1.00
2.22
1.16
1.12
3.35
f1 (ppm)

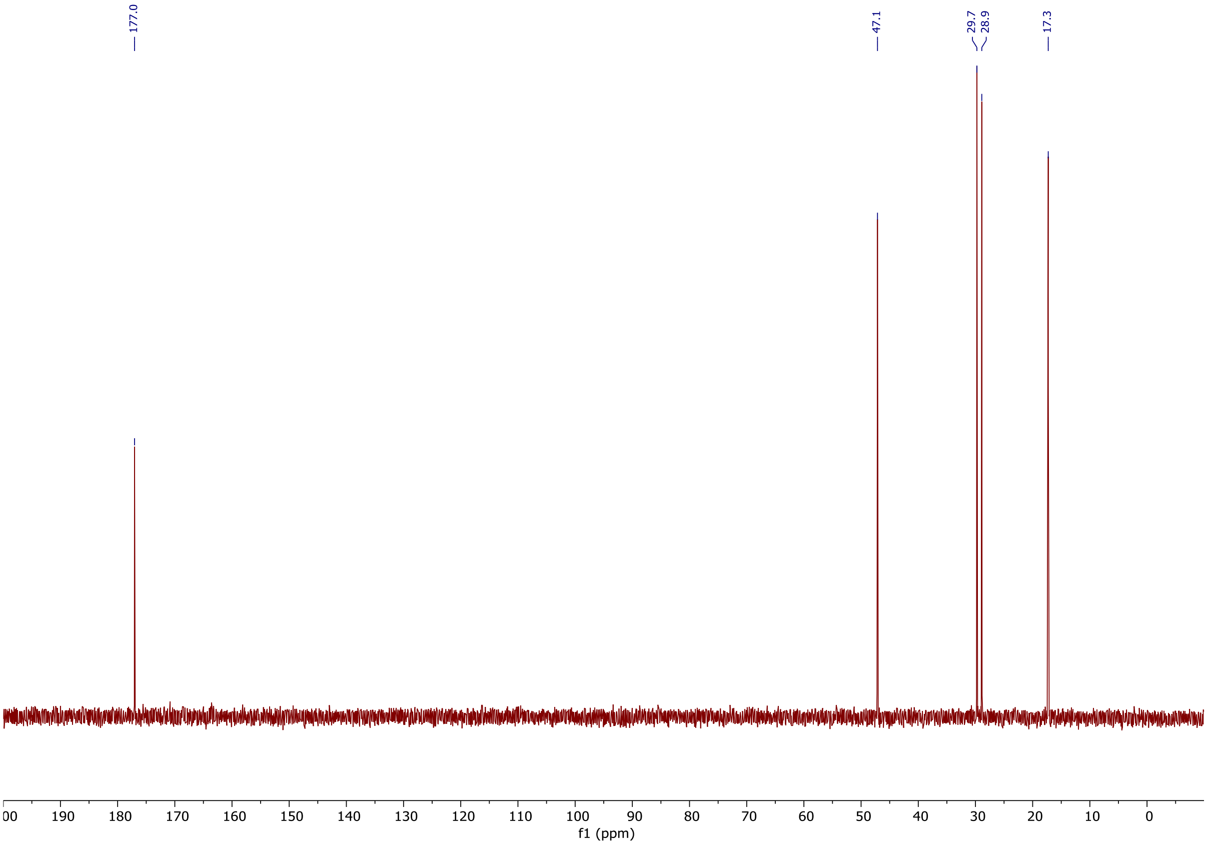
177.0
47.1
29.7
28.9
17.3
f1 (ppm)

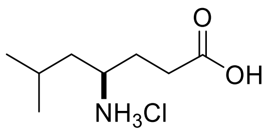
O
OH
NH3Cl

9d

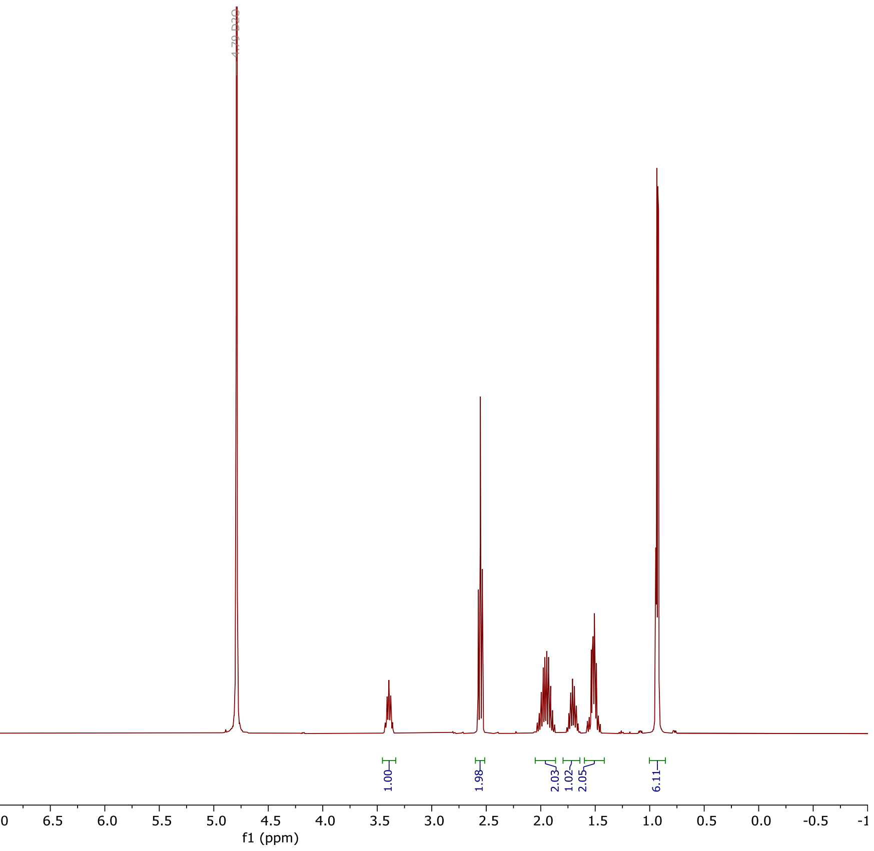
4.79 D2O
1.00
1.98
2.03
1.02
2.05
6.11
f1 (ppm)

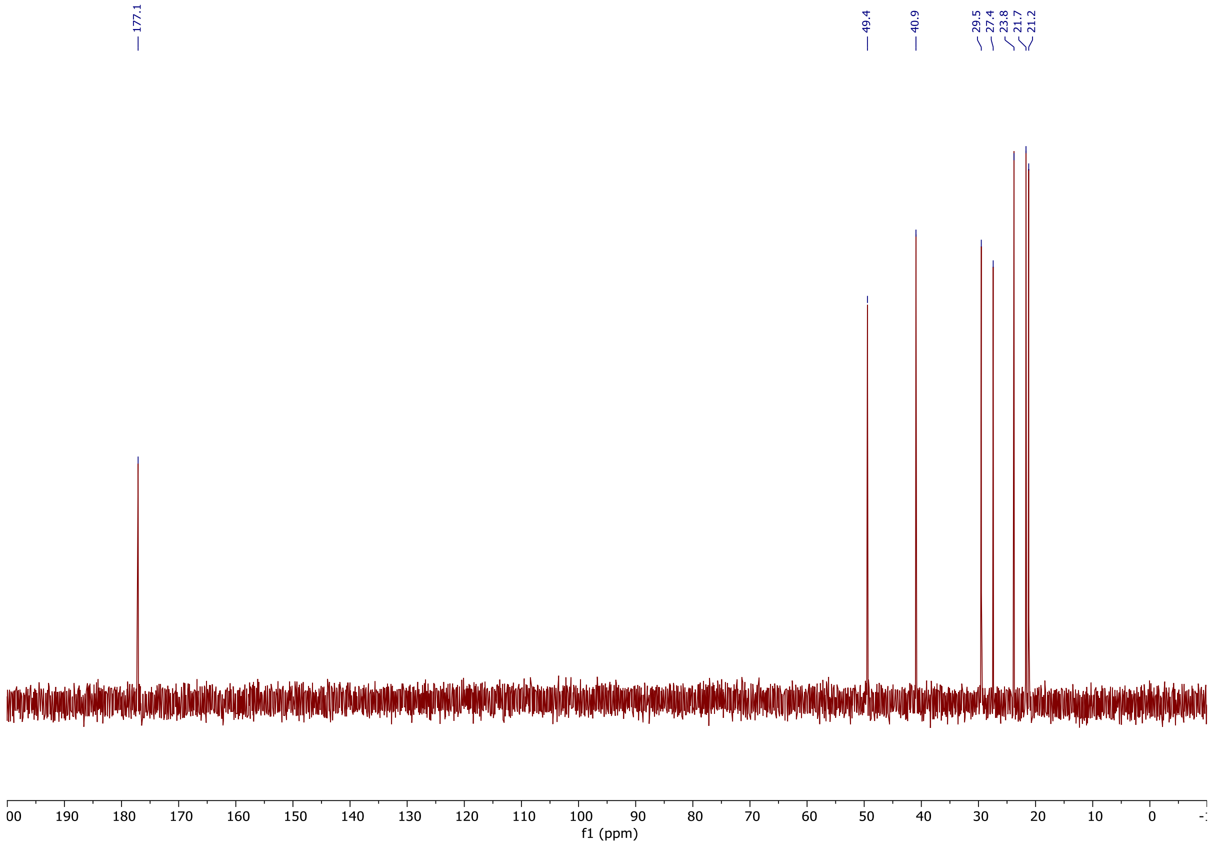
177.1
49.4
40.9
29.5
27.4
23.8
21.7
21.2
f1 (ppm)

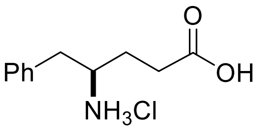
O
OH
Ph
NH3Cl

9e

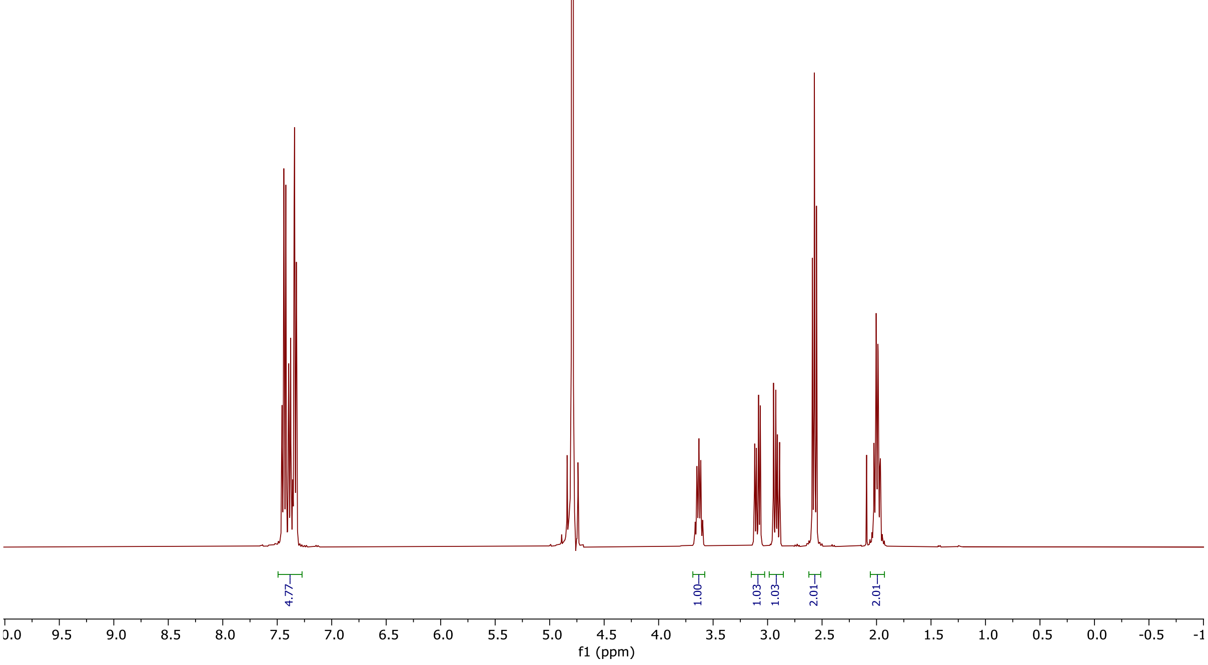
4.77
1.00
1.03
1.03
2.01
2.01
f1 (ppm)

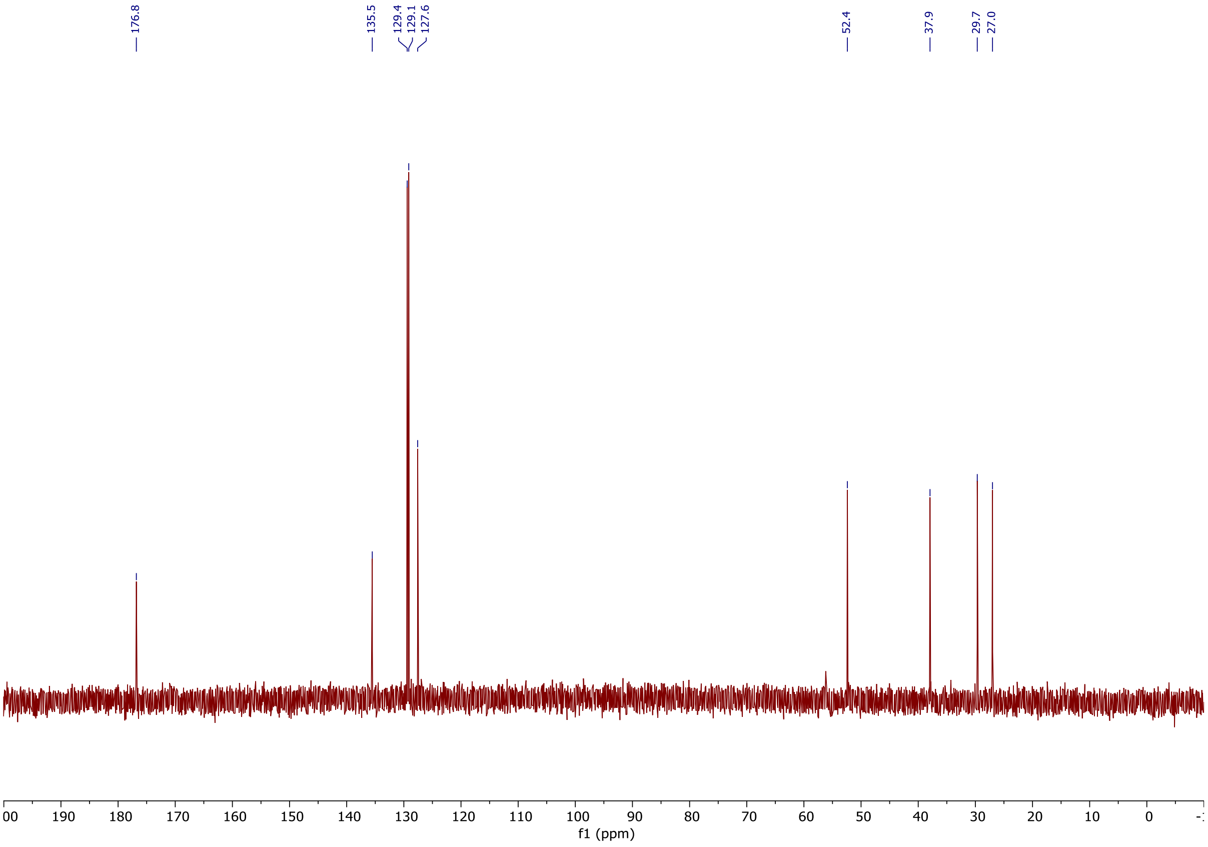
176.8
135.5
129.4
129.1
127.6
52.4
37.9
29.7
27.0
f1 (ppm)

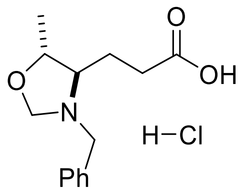

9f

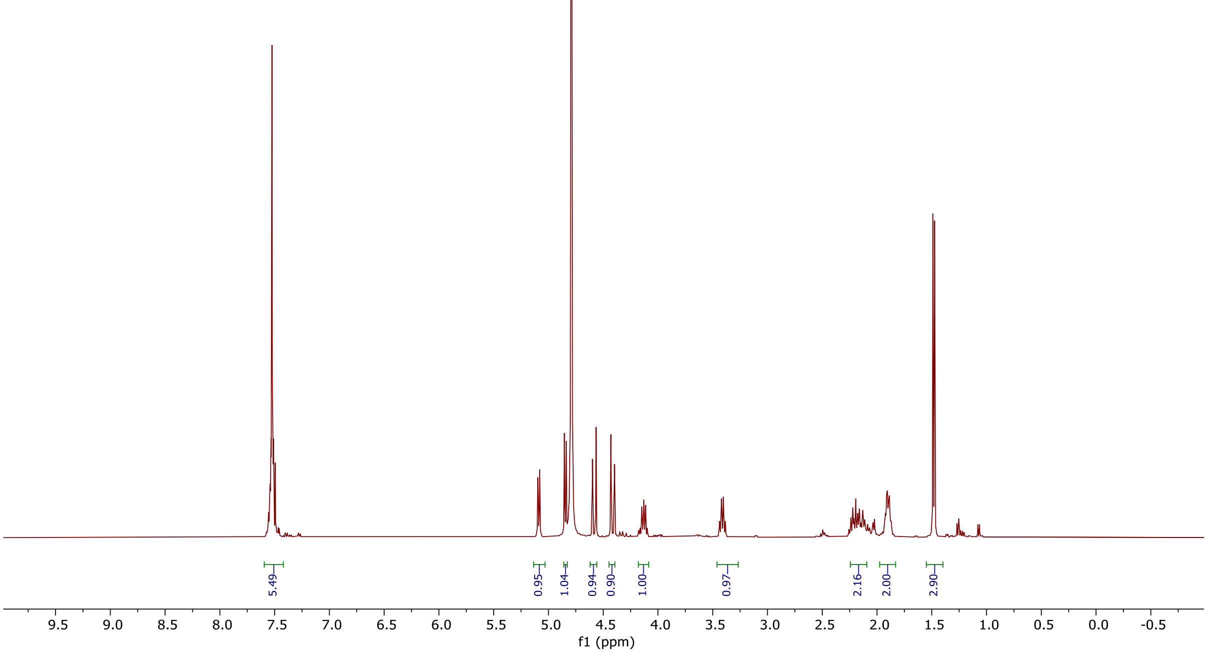

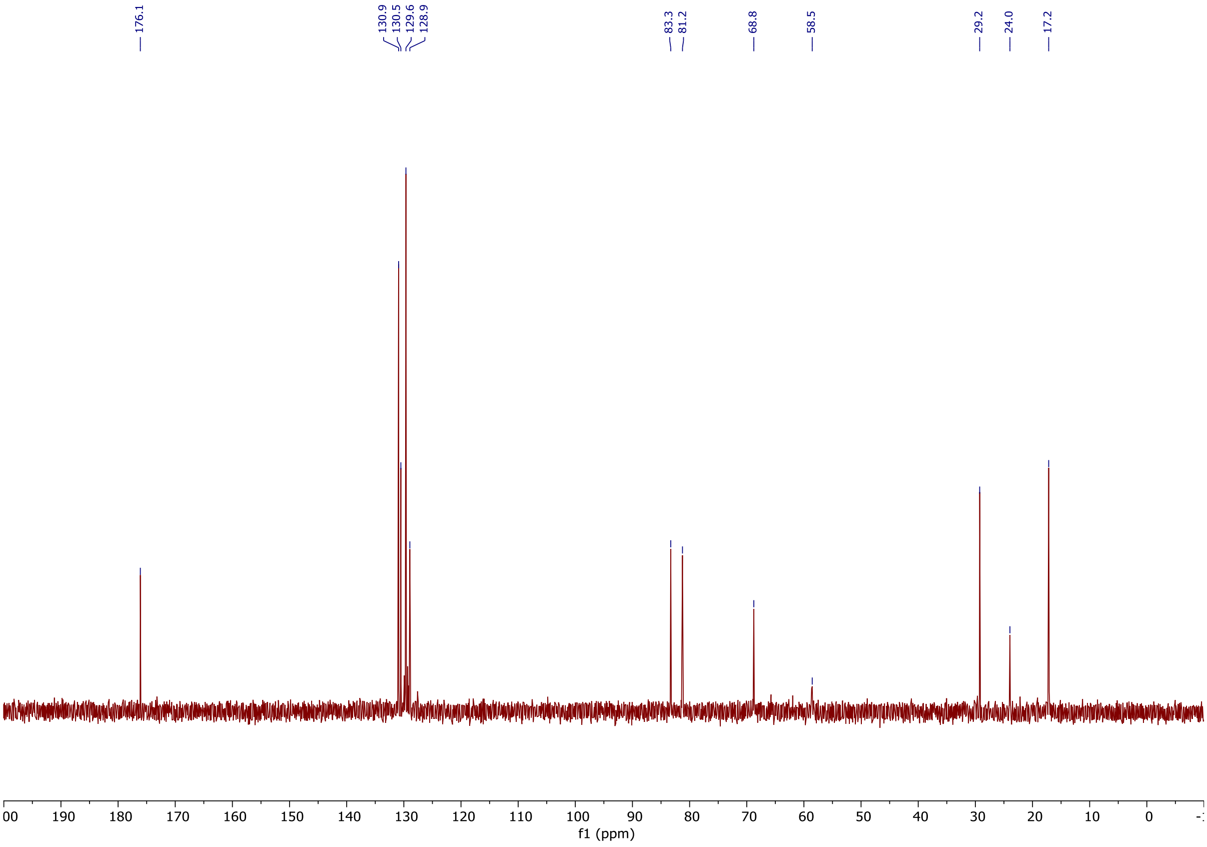

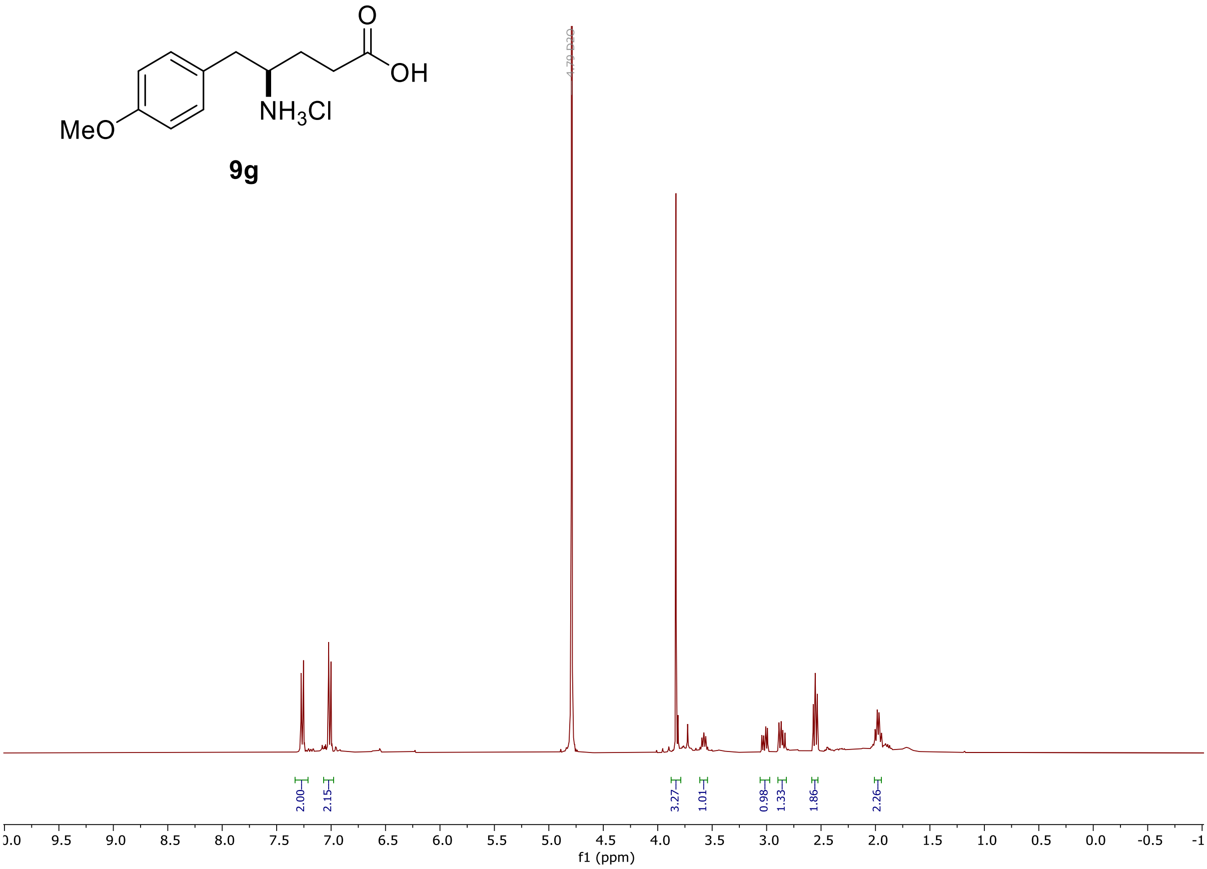
OH
O
MeO
NH3Cl
9g
2.00
2.15
3.27
1.01
0.98
1.33
1.86
2.26
f1 (ppm)

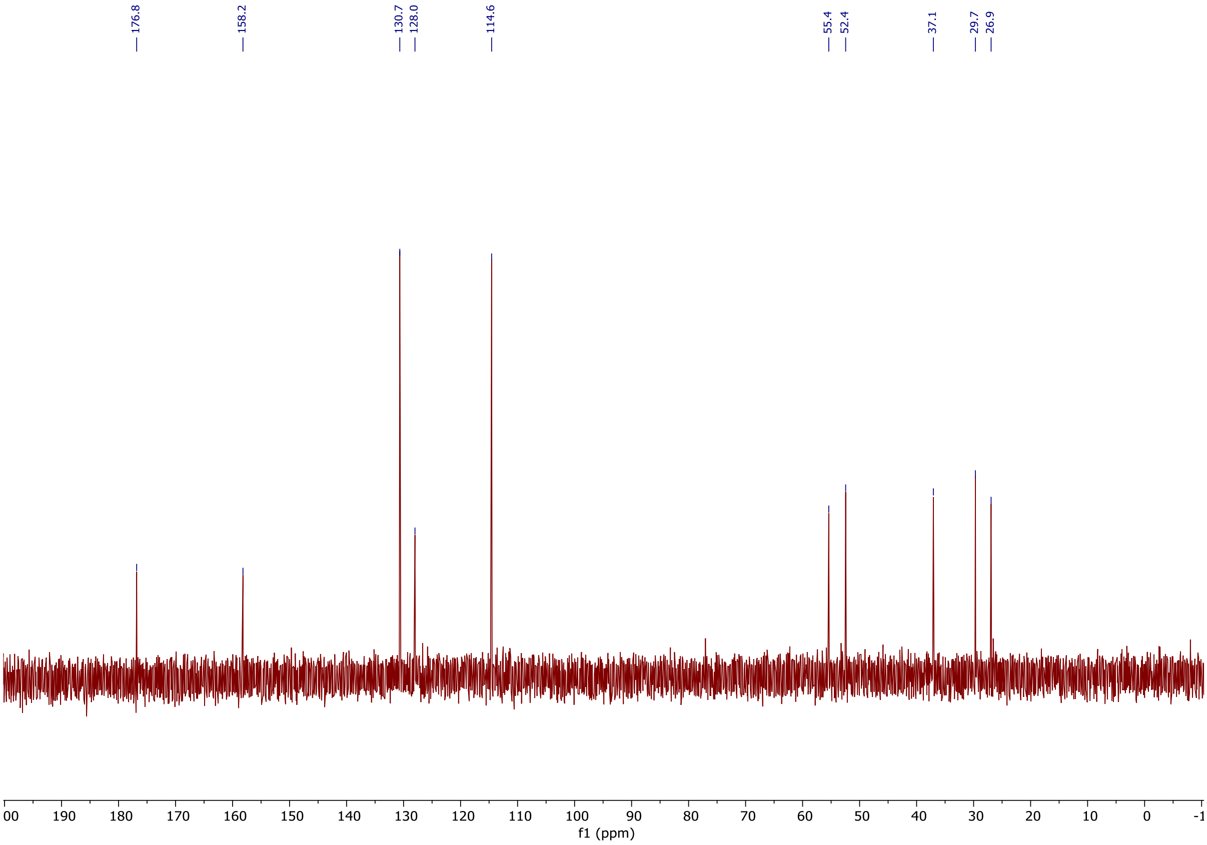
176.8
158.2
130.7
128.0
114.6
55.4
52.4
37.1
29.7
26.9
f1 (ppm)

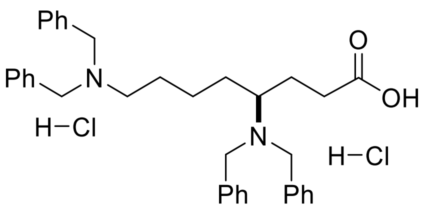
Ph
Ph
N
H−Cl
O
OH
N
H−Cl
Ph
Ph

9h

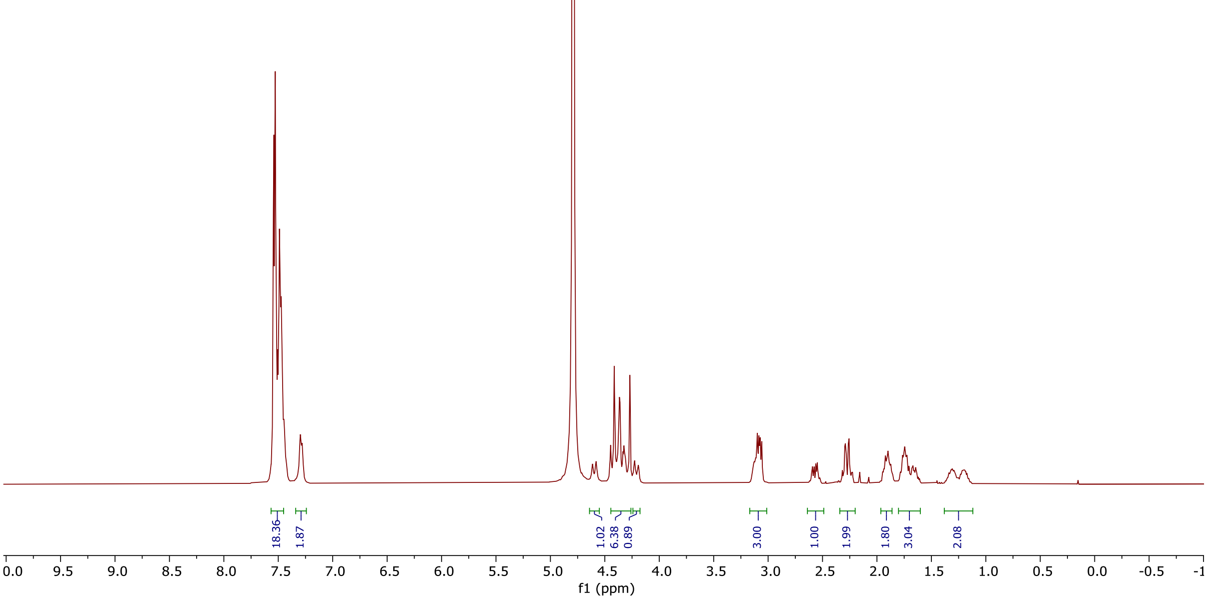
18.36
1.87
1.02
6.38
0.89
3.00
1.00
1.99
1.80
3.04
2.08
f1 (ppm)

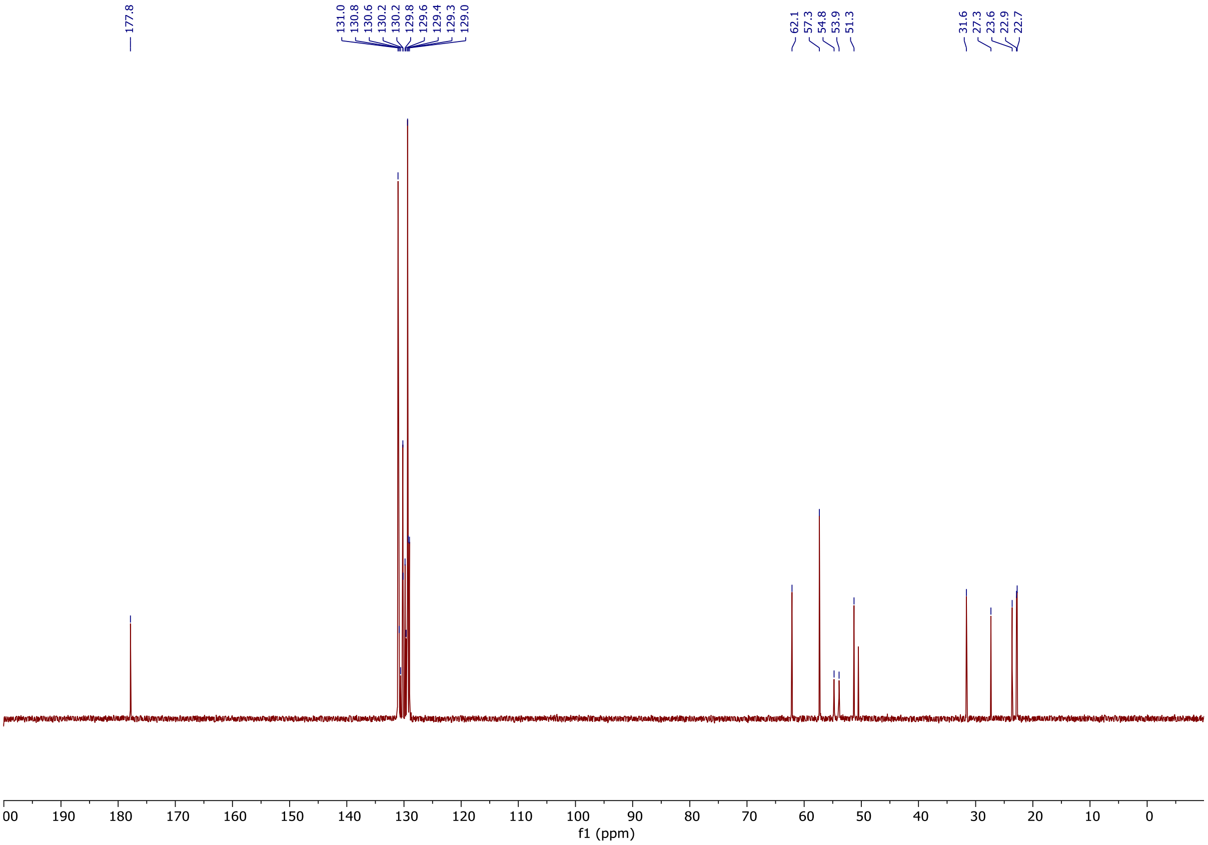
177.8
131.0
130.8
130.6
130.2
130.2
129.8
129.6
129.4
129.3
129.0
62.1
57.3
54.8
53.9
51.3
31.6
27.3
23.6
22.9
22.7
f1 (ppm)

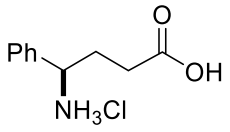
O
Ph
OH
NH3Cl

9i

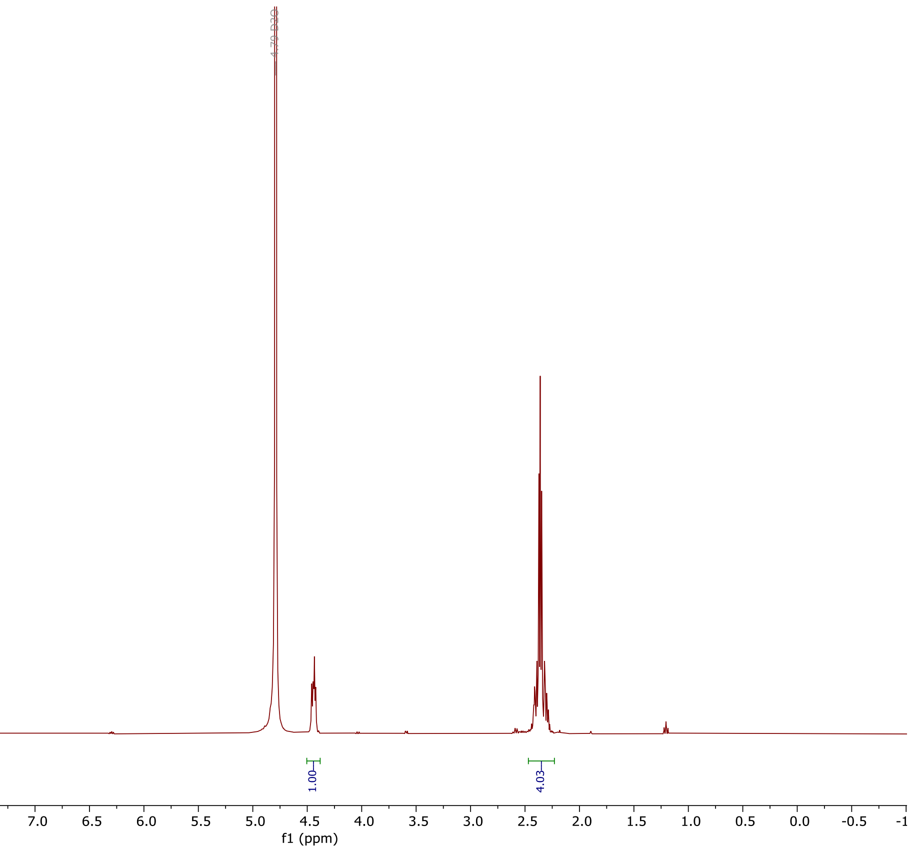
4.70
1.00
4.03
f1 (ppm)

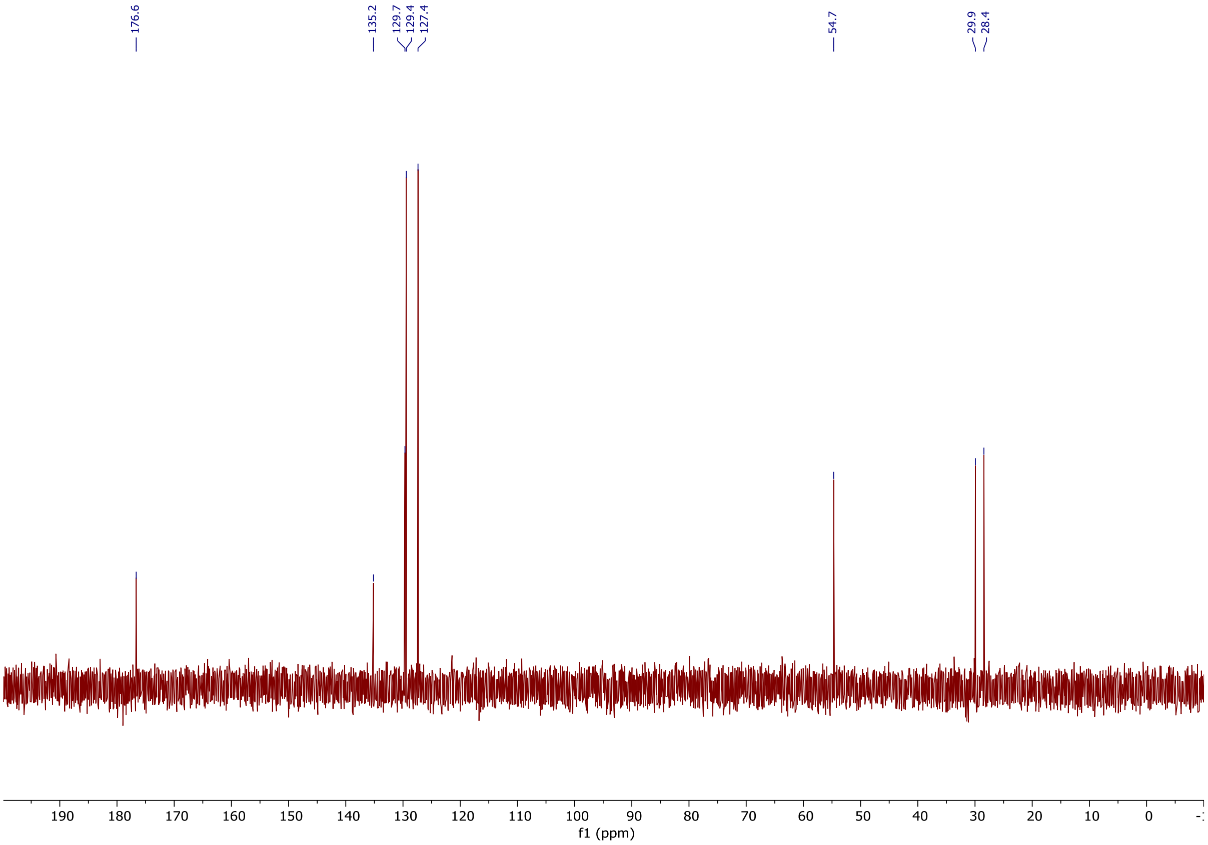
176.6
135.2
129.7
129.4
127.4
54.7
29.9
28.4
f1 (ppm)

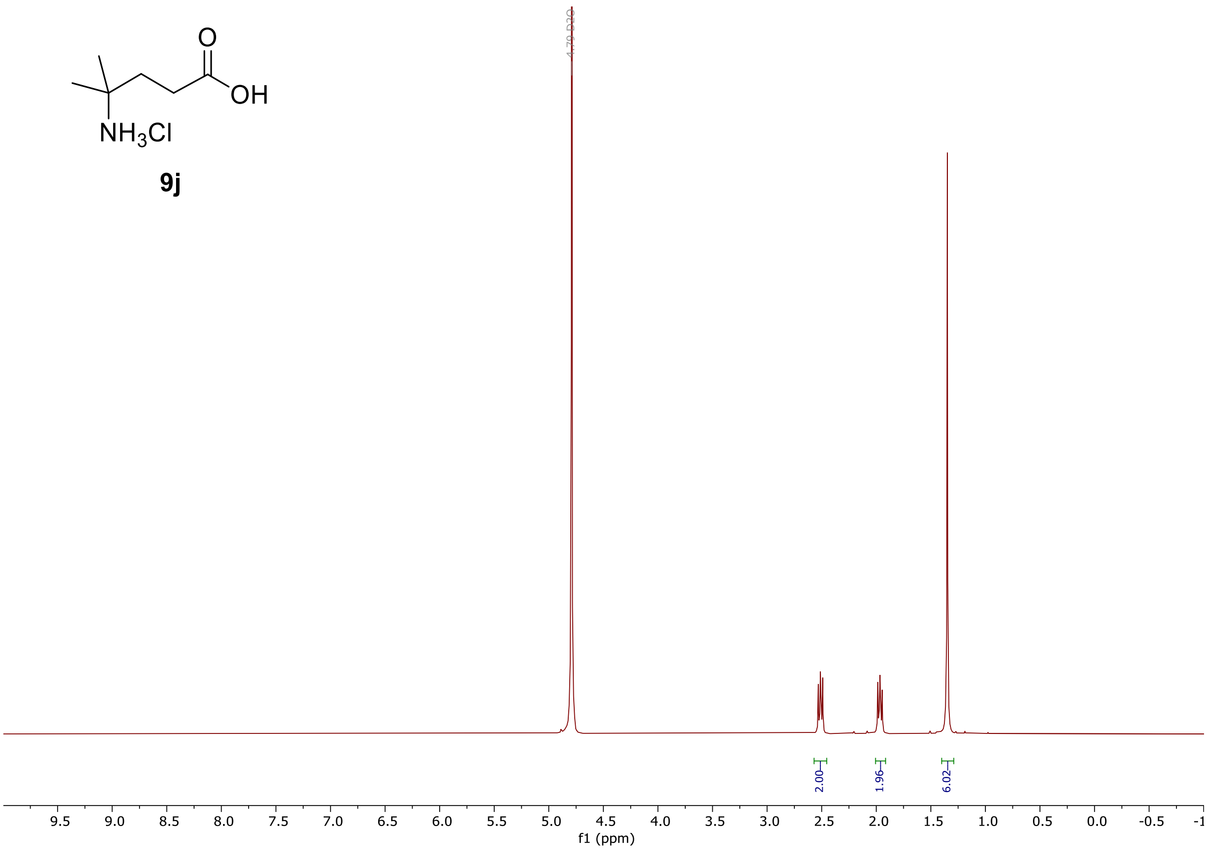
O
OH
NH3Cl
9j
2.00
1.96
6.02
f1 (ppm)

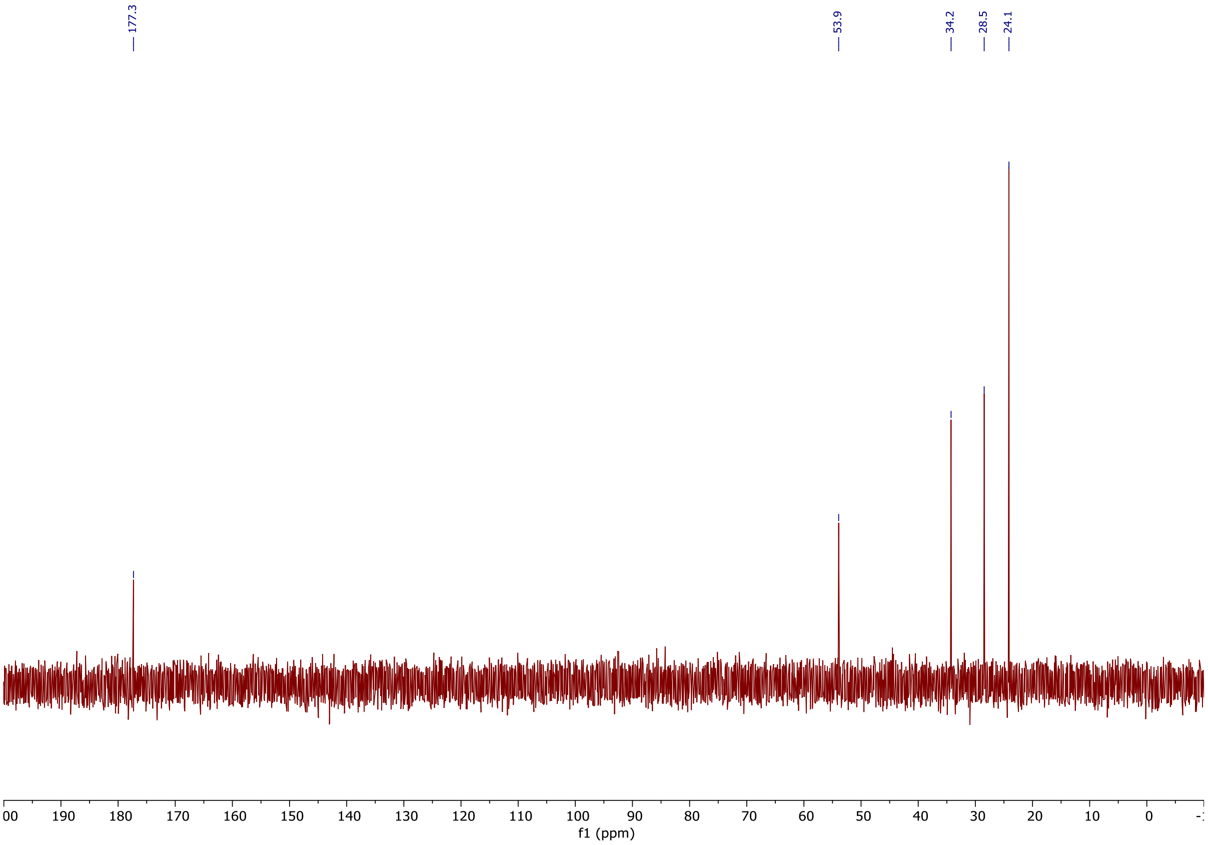
177.3
53.9
34.2
28.5
24.1
f1 (ppm)

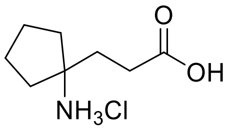

9k

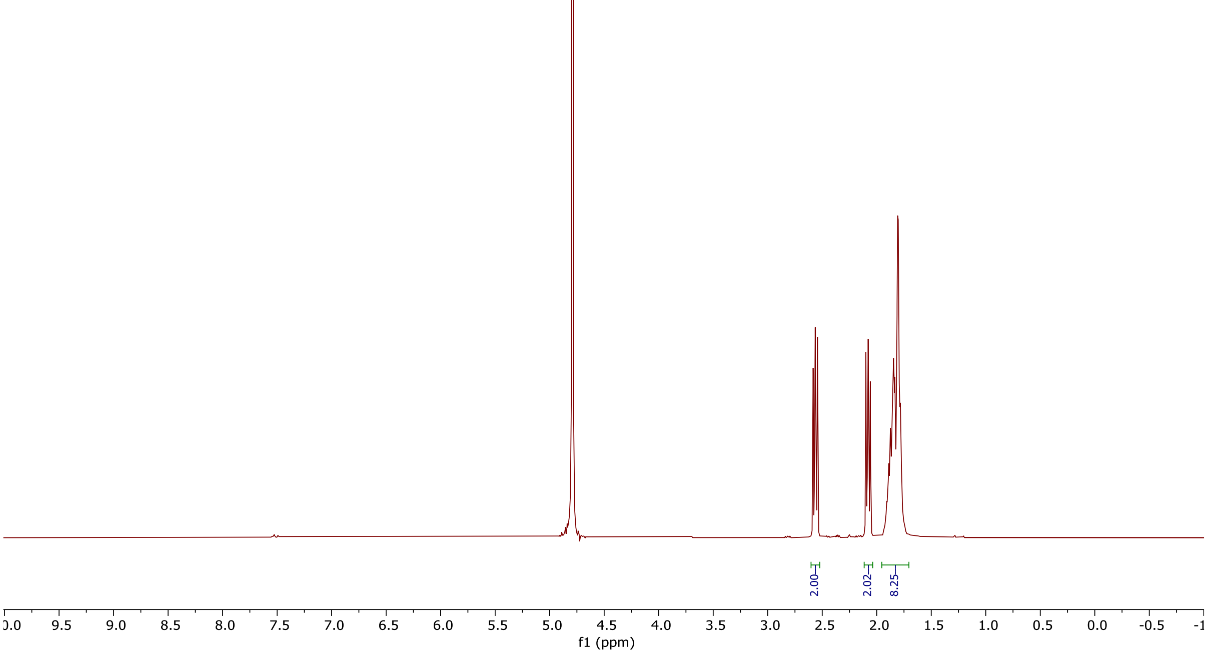

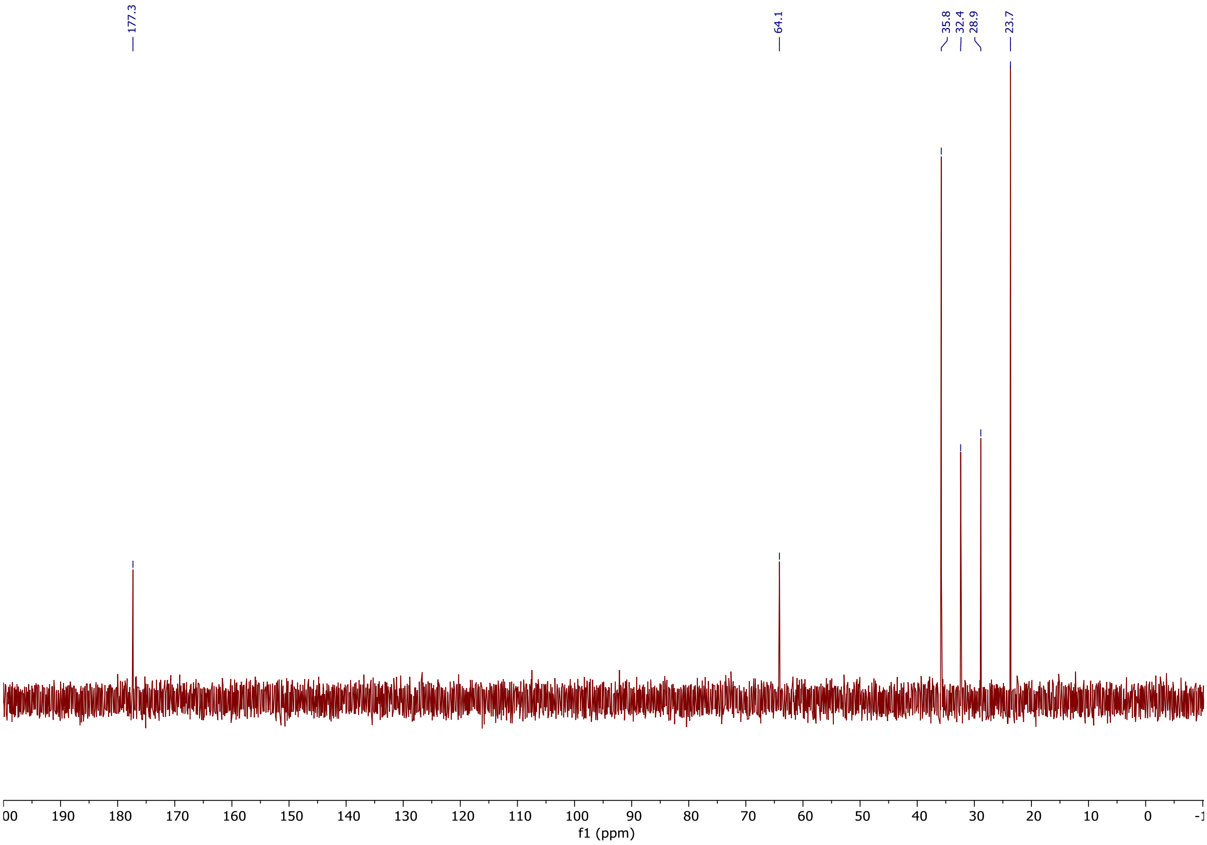

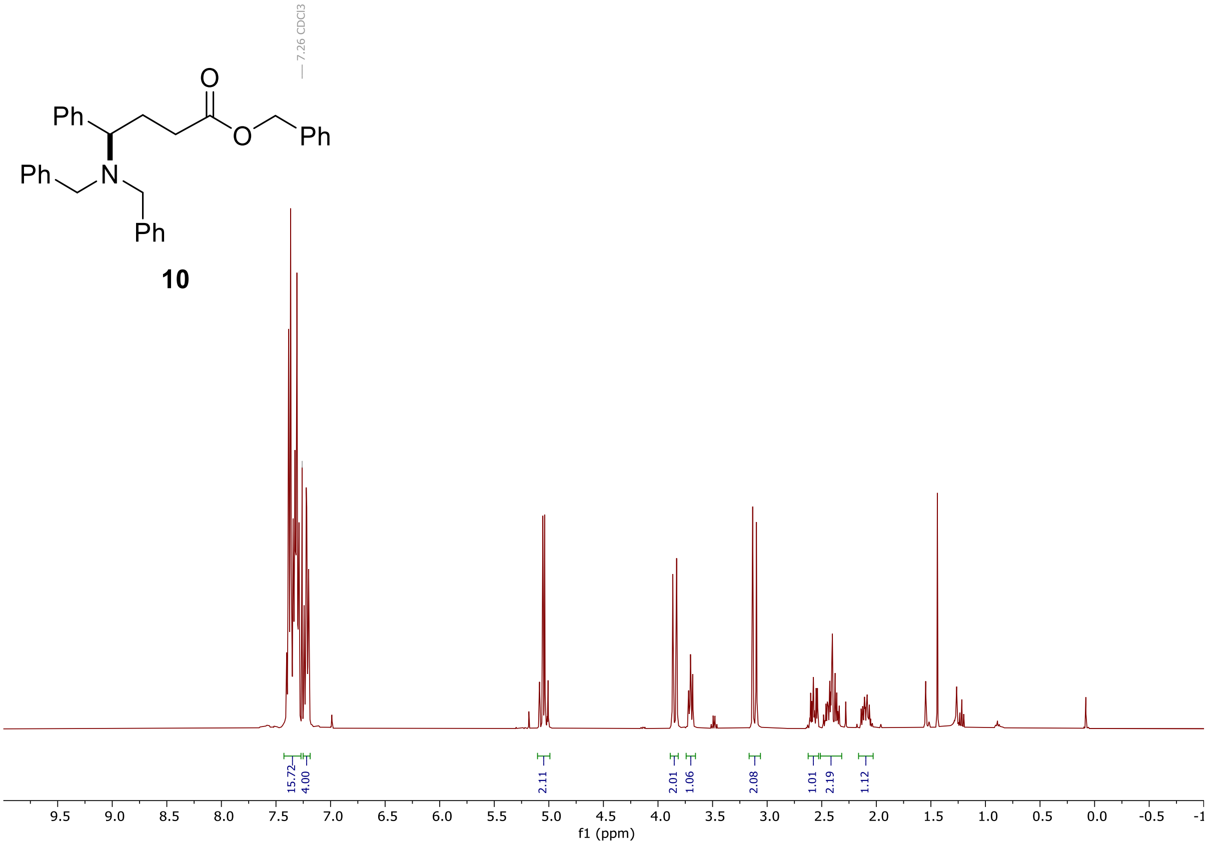
7.26 CDCl3
Ph
O
O
Ph
Ph
N
Ph
Ph
10
15.72
4.00
2.11
2.01
1.06
2.08
1.01
2.19
1.12
9.5
9.0
8.5
8.0
7.5
7.0
6.5
6.0
5.5
5.0
4.5
4.0
3.5
3.0
2.5
2.0
1.5
1.0
0.5
0.0
-0.5
f1 (ppm)

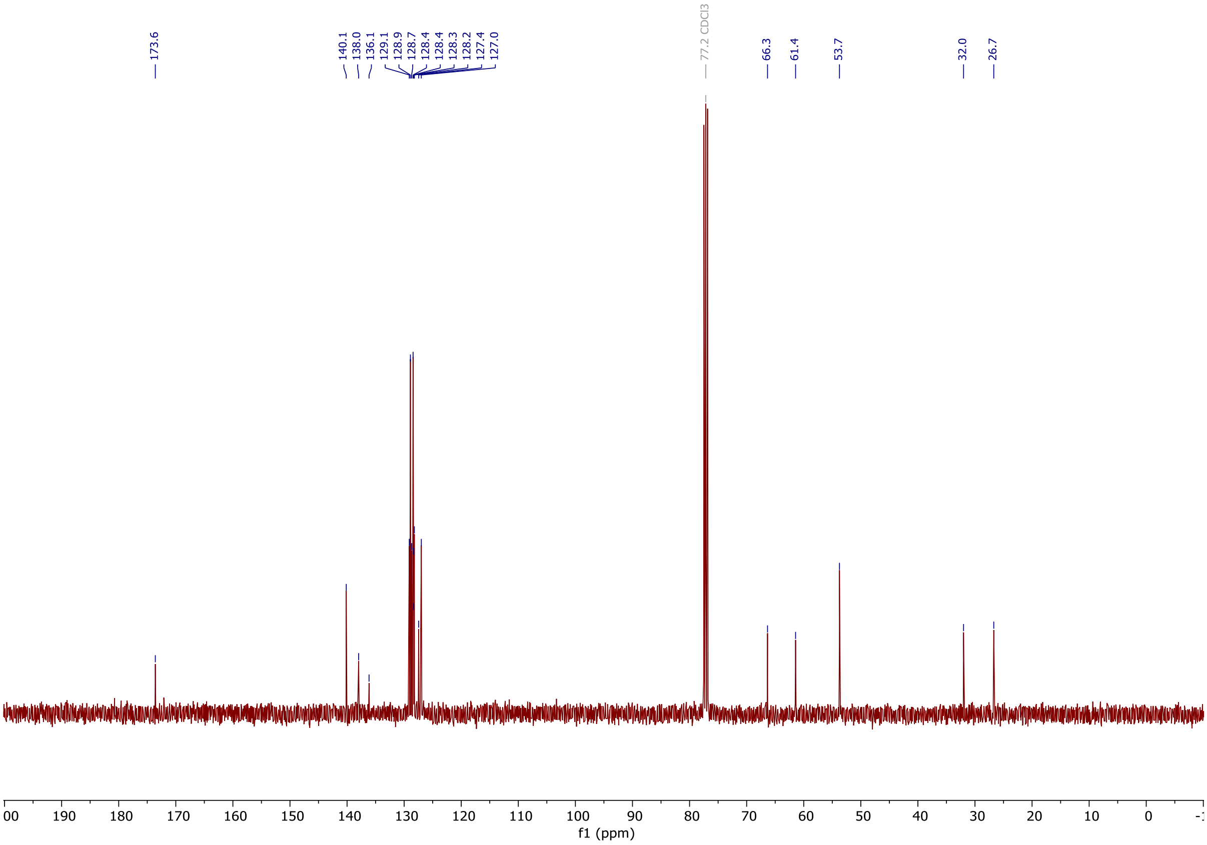
173.6
140.1
138.0
136.1
129.1
128.9
128.7
128.4
128.4
128.3
128.2
127.4
127.0
77.2 CDCl3
66.3
61.4
53.7
32.0
26.7
190
180
170
160
150
140
130
120
110
100
90
80
70
60
50
40
30
20
10
0
f1 (ppm)

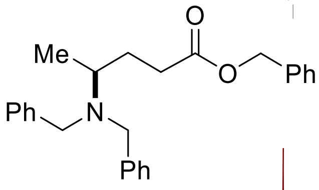
Me
O
O
Ph
Ph
N
Ph

S25

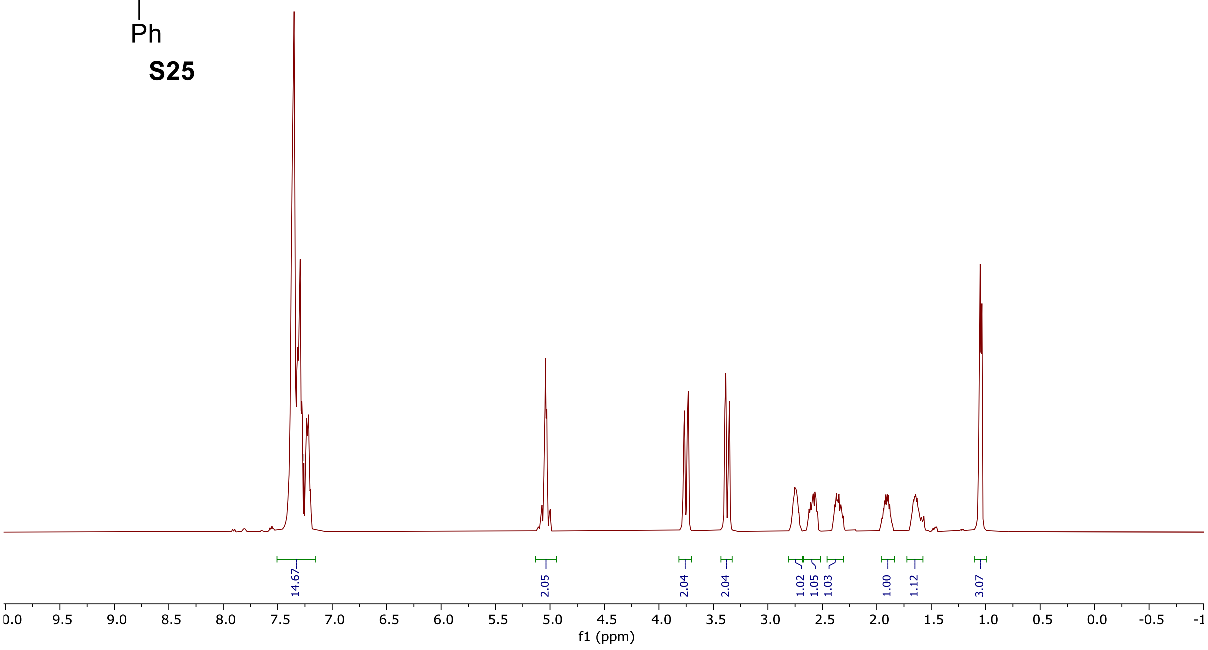
7.26 CDCl3
14.67
2.05
2.04
2.04
1.02
1.05
1.03
1.00
1.12
3.07
f1 (ppm)

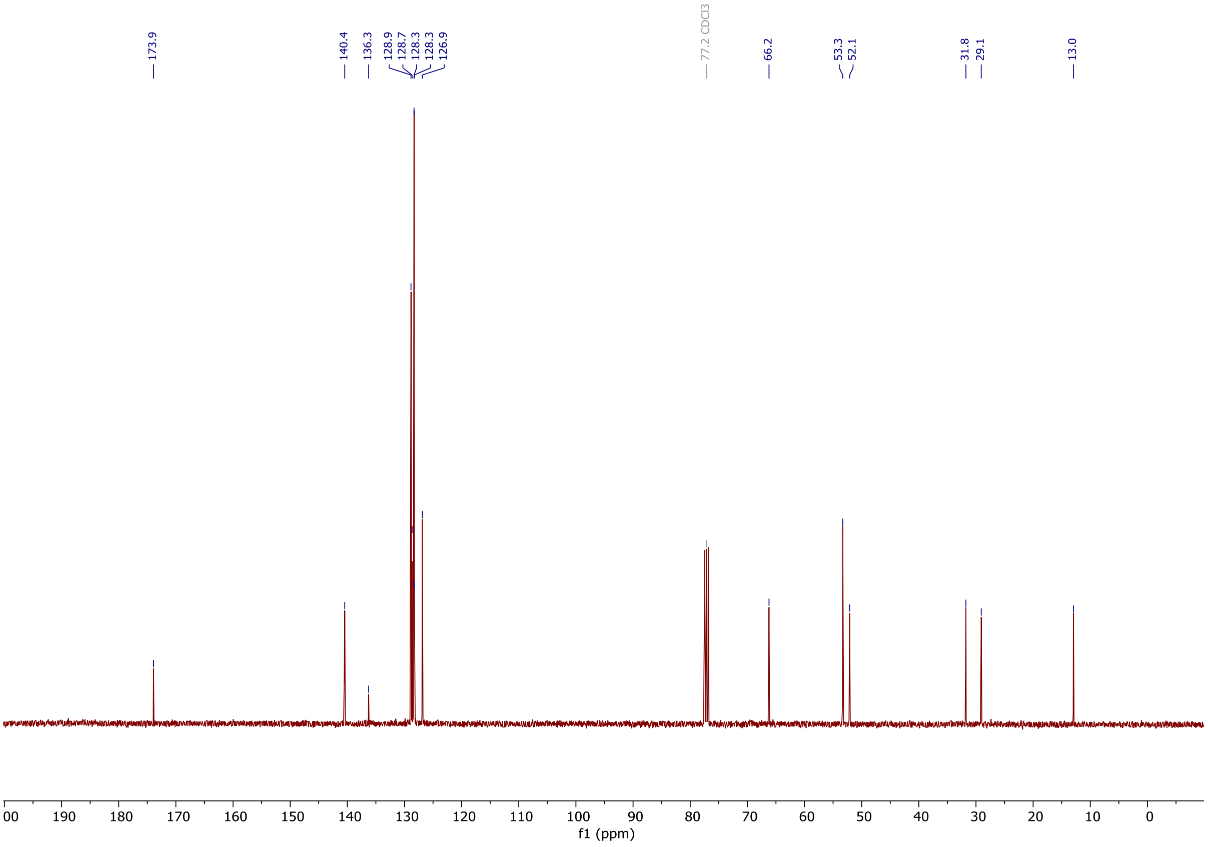
173.9
140.4
136.3
128.9
128.7
128.3
128.3
126.9
77.2 CDCl3
66.2
53.3
52.1
31.8
29.1
13.0
f1 (ppm)

7. *HRMS Data for Deuterated Compounds*

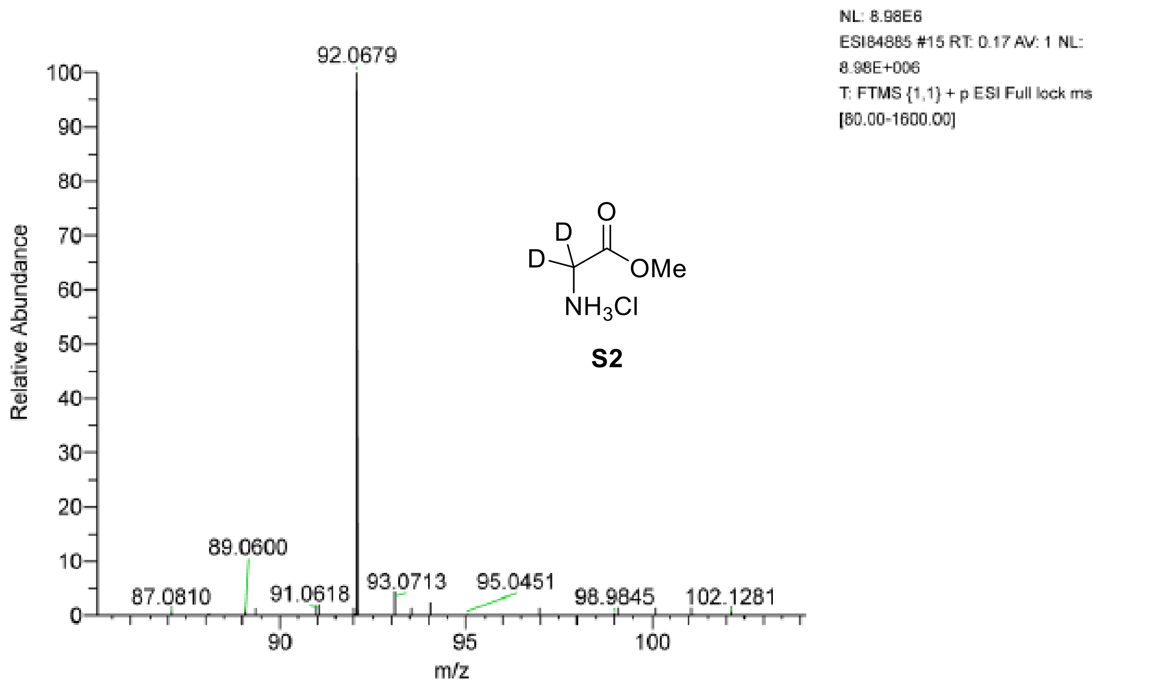

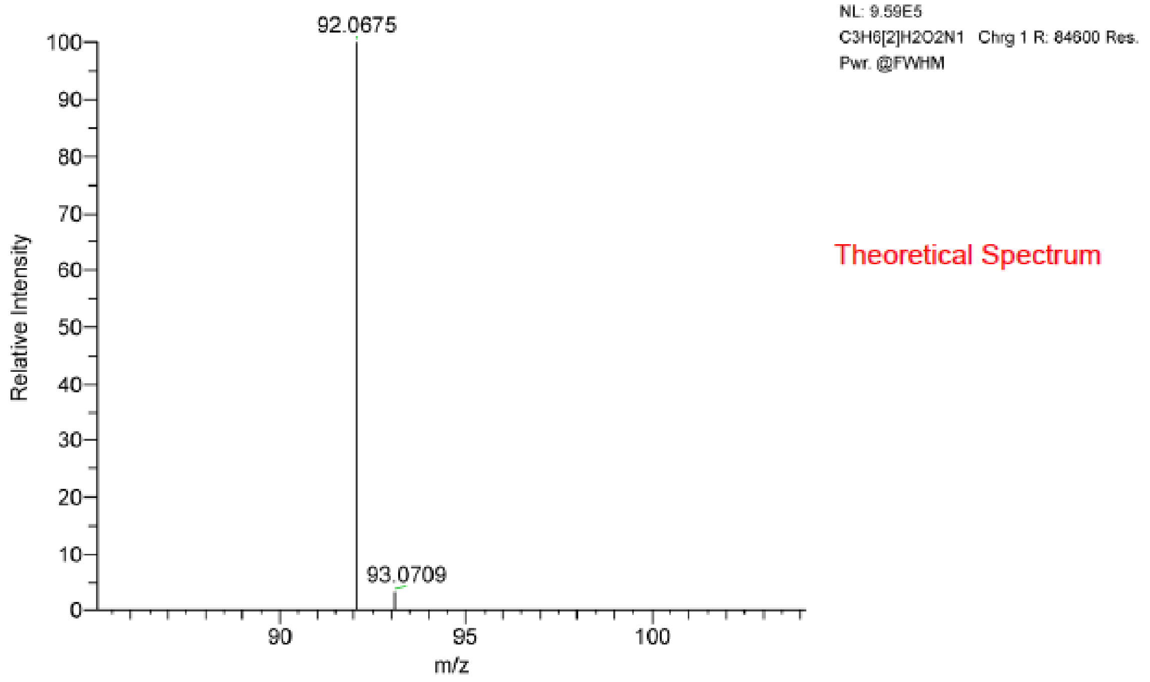

Peak Mass	Display...	Combin...	RDB	Delta [p...	Theo. m...	Rank	Combin...	# Match...	# Misse...	MS Cov...	Pattern...	MSMS...
92.0679	$C_3H_6{}^2H$...	0	0.50	4.19	92.06751	1	0	0	4	0	0	(Collec...

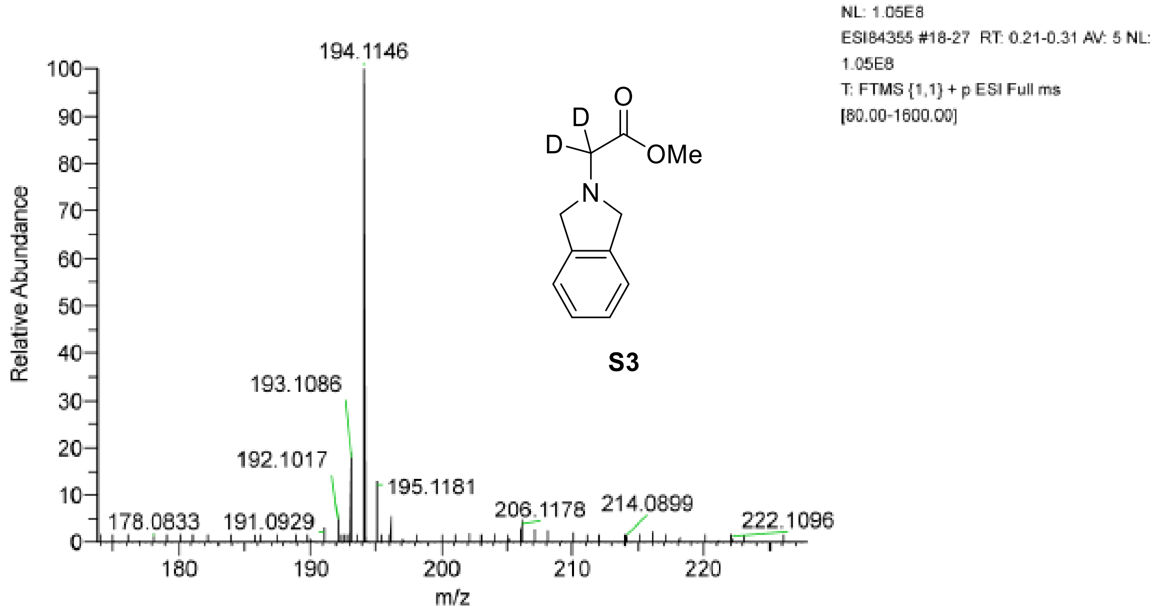

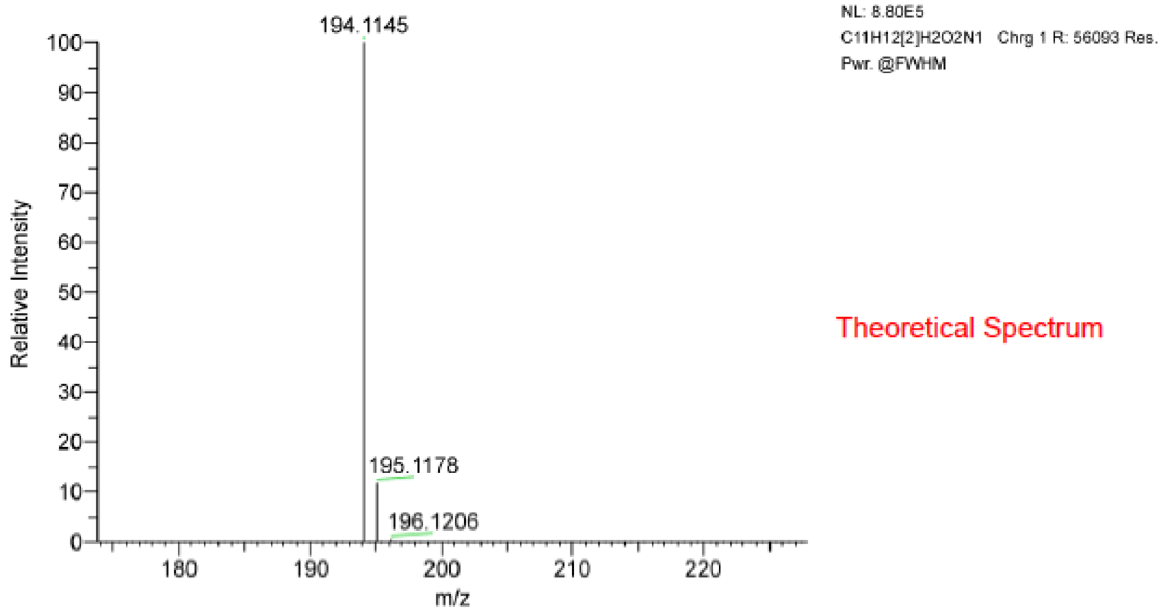

Peak Mass	Display...	Combin...	RDB	Delta [p...	Theo. m...	Rank	Combin...	# Match...	# Misse...	MS Cov...	Pattern...	MSMS...
194.1146	$C_{11}H_{12}{}^{2}$...	13.116...	5.50	0.78	194.11...	1	16.654...	1	3	16.850...	88.394...	(Collec...

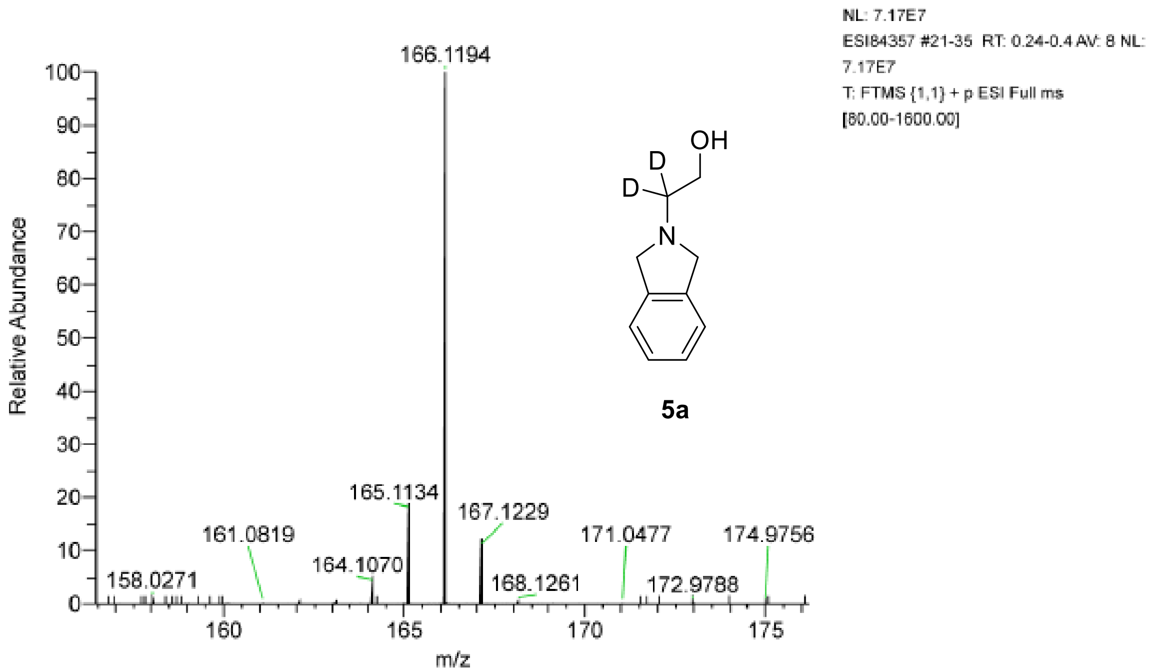

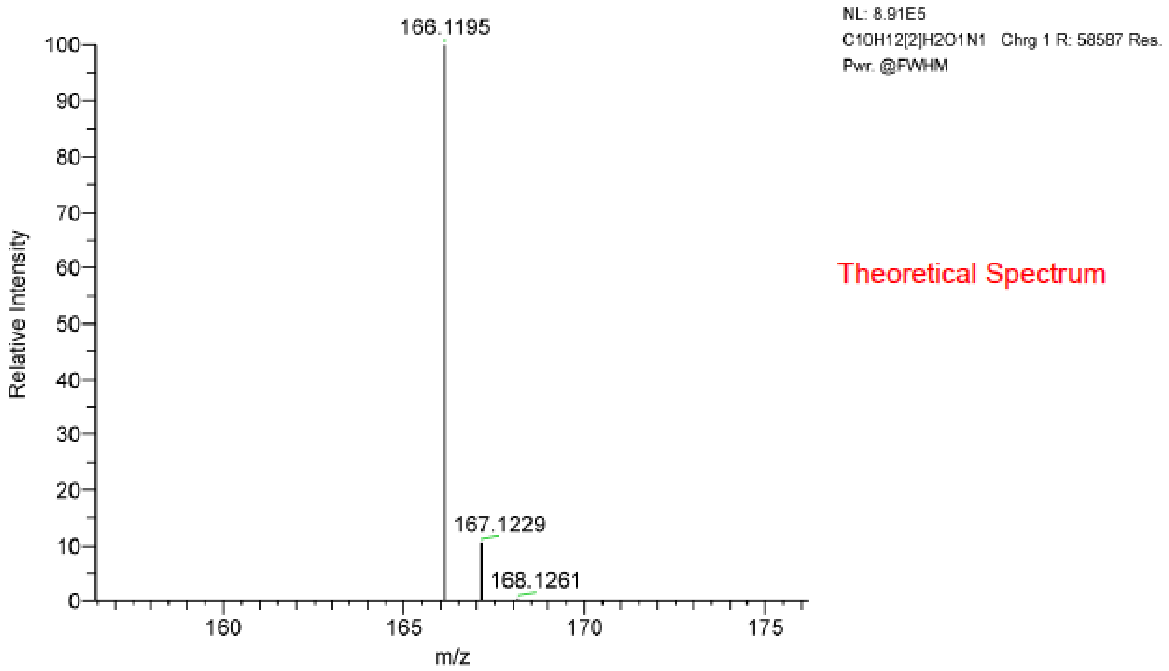

Peak Mass	Display...	Combin...	RDB	Delta [p...	Theo. m...	Rank	Combin...	# Match...	# Misse...	MS Cov....	Pattern...	MSMS...
166.1194	$C_{10}H_{12}{}^{2}$...	13.366...	4.50	-0.70	166.11...	1	13.419...	1	3	13.422...	89.463...	(Collec...

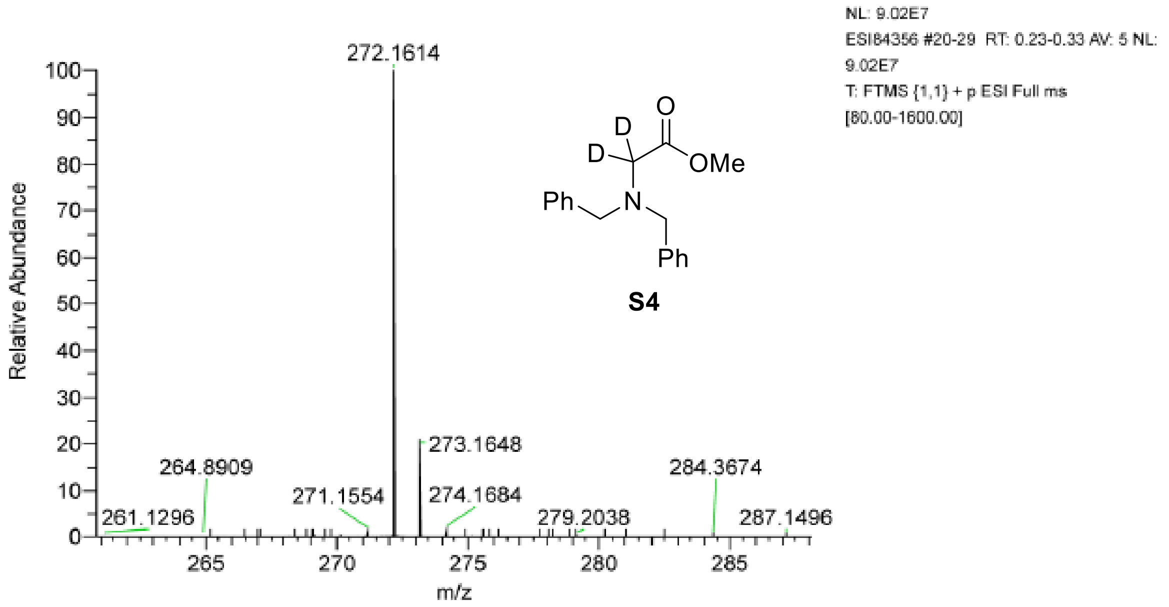

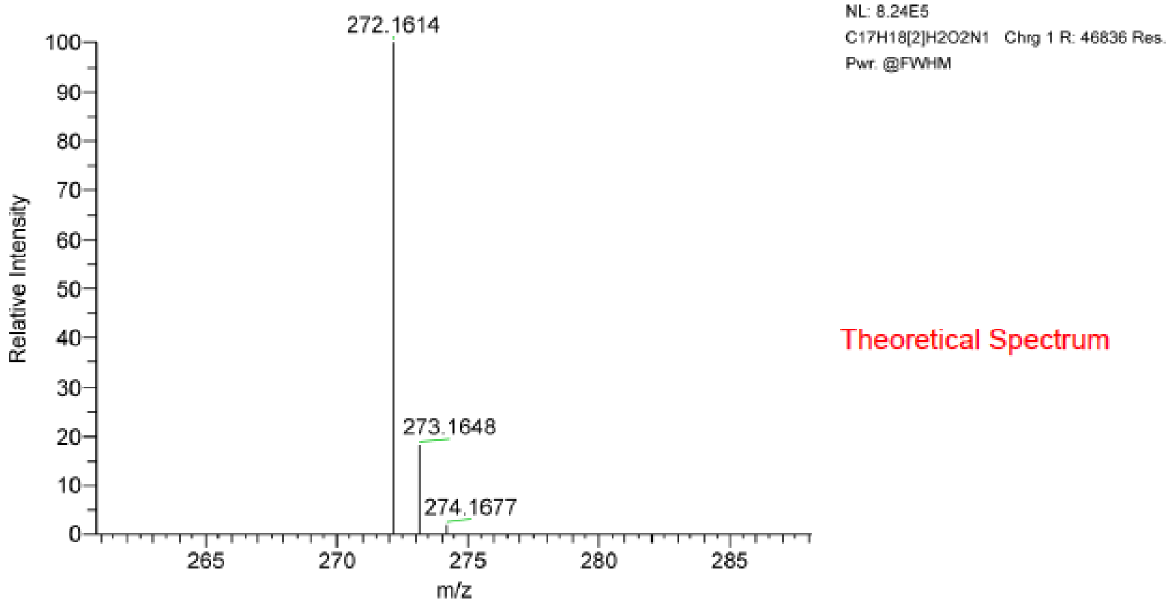

Theoretical Spectrum

Peak Mass	Display...	Combin...	RDB	Delta [p...	Theo. m...	Rank	Combin...	# Match...	# Misse...	MS Cov...	Pattern...	MSMS...
272.1614	$C_{17}H_{18}{}^{2}$...	0	8.50	-0.20	272.16...	1	0	0	4	0	0	(Collec...

NL: 1.69E8
ESI84354 #16-24 RT: 0.18-0.27 AV: 4 NL: 1.69E8
T: FTMS {1,1} + p ESI Full ms [80.00-1600.00]

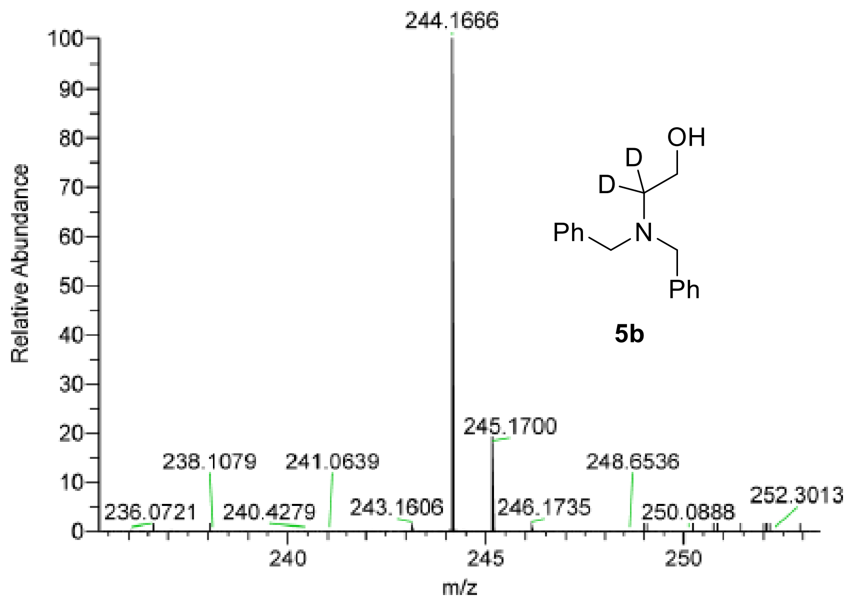

NL: 8.35E5
C16H18[2]H2O1N1 Chrg 1 R: 49943 Res. Pwr. @FWHM

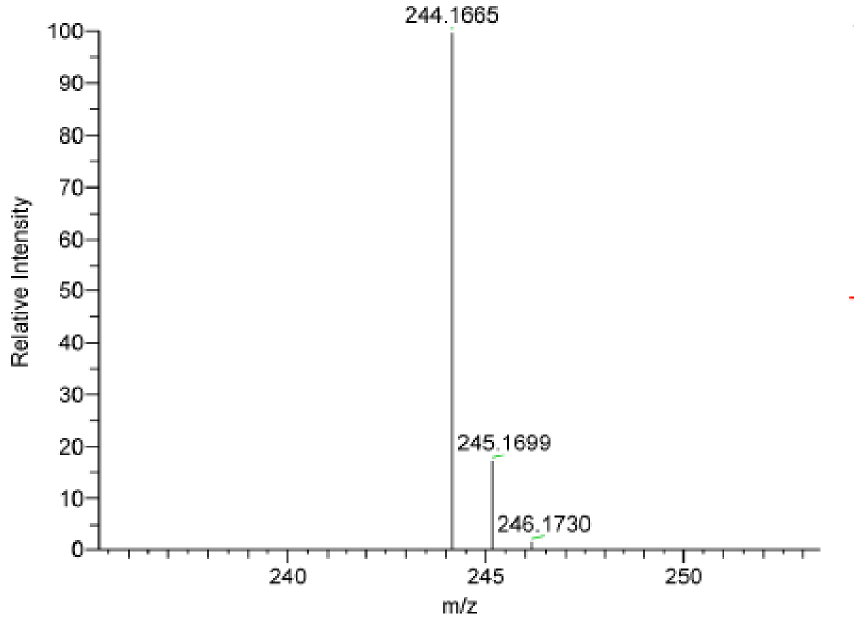

Theoretical Spectrum

Peak Mass	Display...	Combin...	RDB	Delta [p...	Theo. m...	Rank	Combin...	# Match...	# Misse...	MS Cov....	Pattern...	MSMS...
244.1666	$C_{16}H_{18}{}^{2}$...	0	7.50	0.23	244.16...	1	0	0	3	0	0	(Collec...

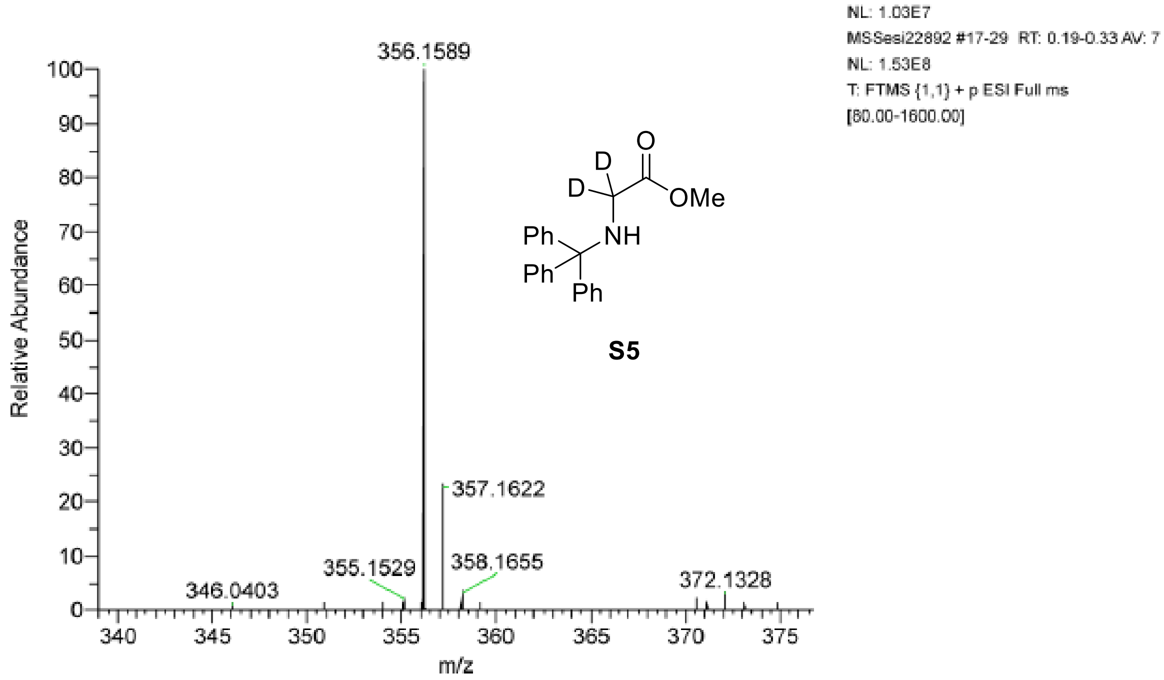

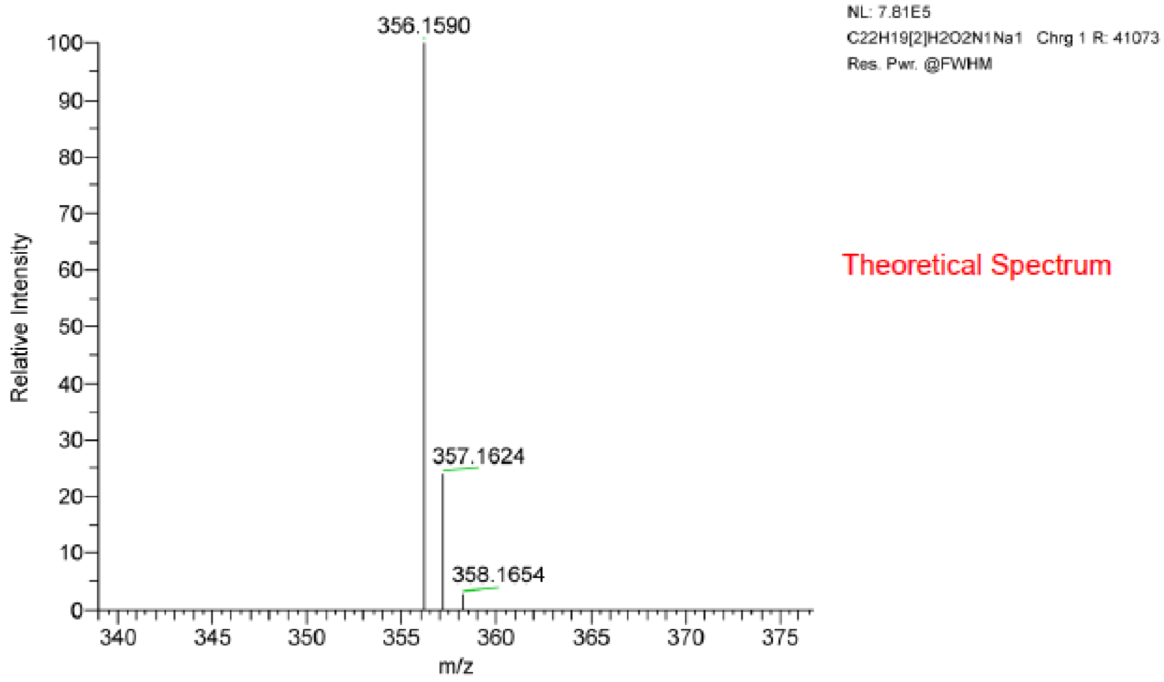

Peak Mass	Display...	Combin...	RDB	Delta [p...	Theo. m...	Rank	Combin...	# Match...	# Misse...	MS Cov....	Pattern...	MSMS...
356.1589	$C_{22}H_{19}{}^{2}$...	0	12.50	-0.40	356.15...	1	0	0	4	0	0	(Collec...

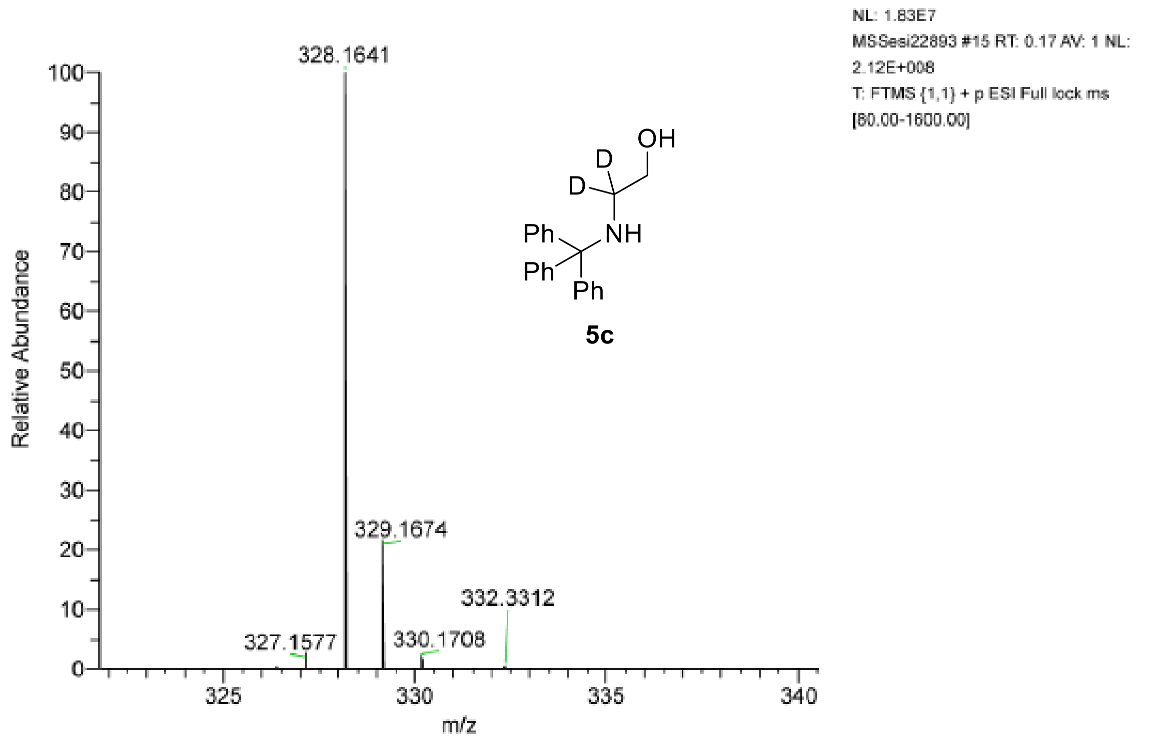

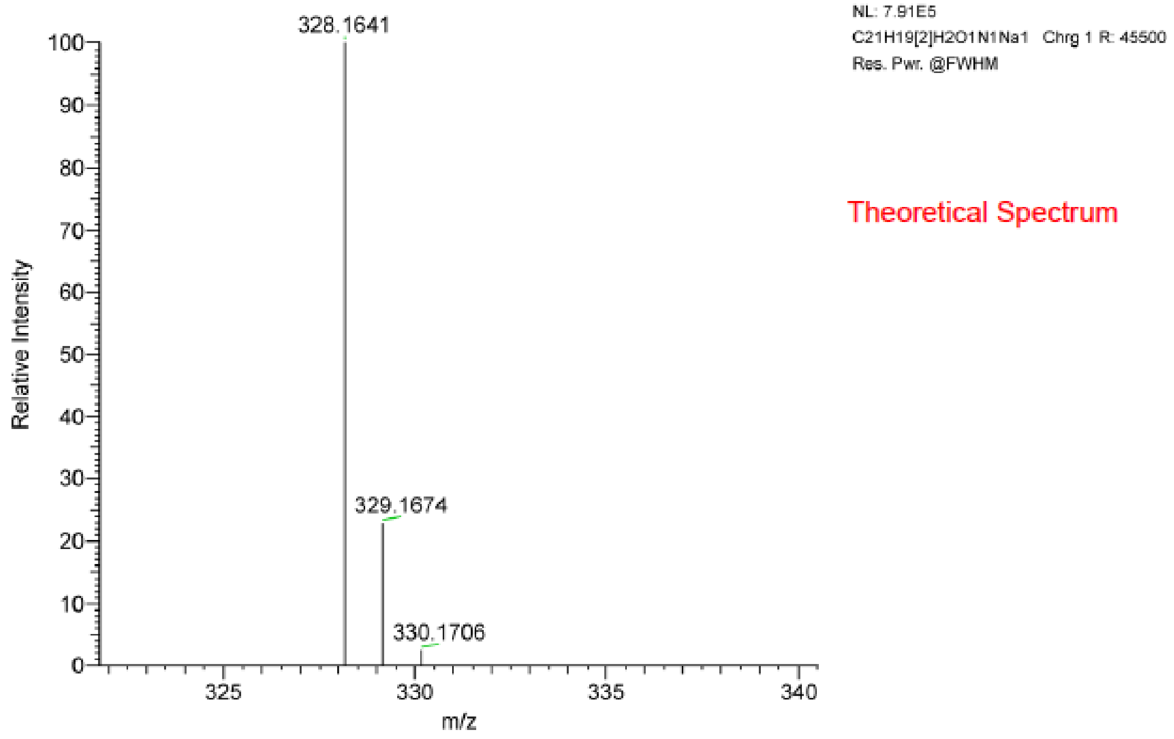

Peak Mass	Display...	Combin...	RDB	Delta [p...	Theo. m...	Rank	Combin...	# Match...	# Misse...	MS Cov...	Pattern...	MSMS...
328.1641	$C_{21}H_{19}{}^{2}$...	0	11.50	-0.08	328.16...	1	0	0	4	0	0	(Collec...

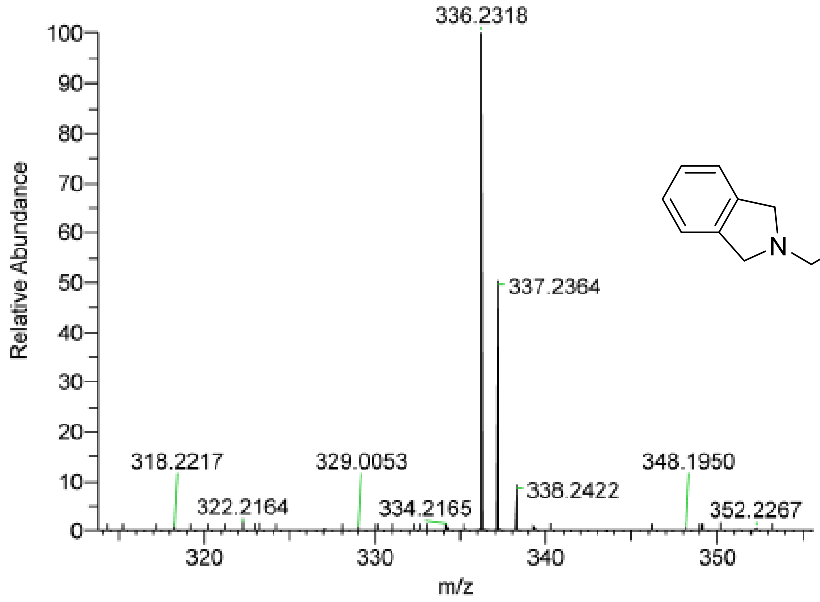

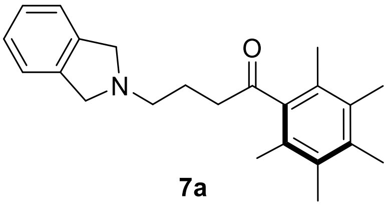

7a

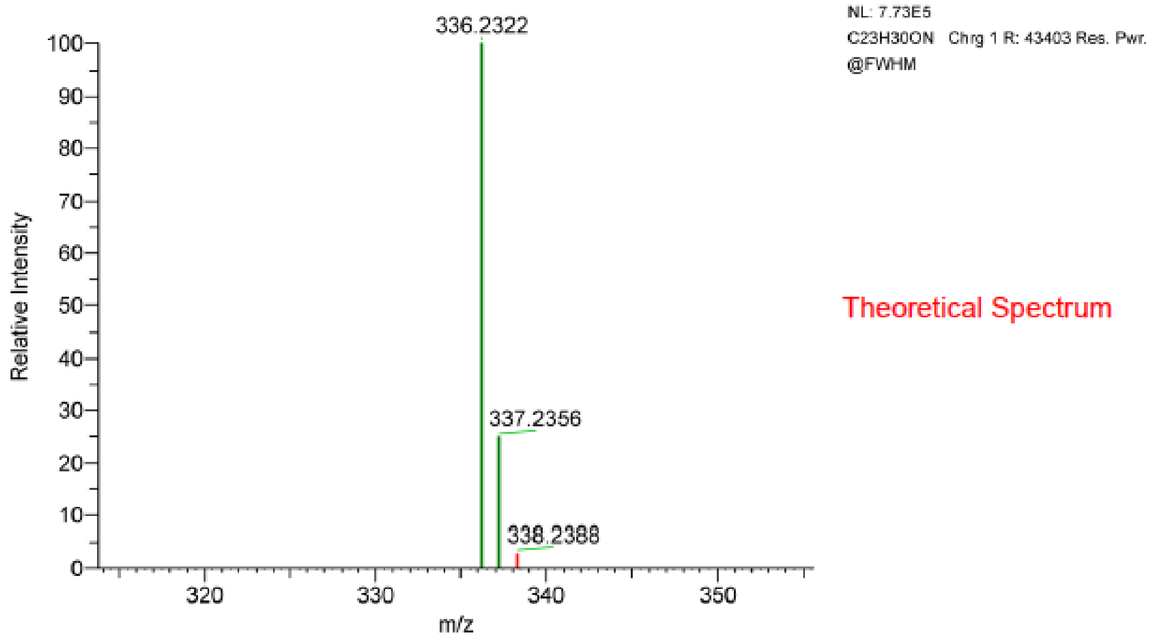

Theoretical Spectrum

Peak Mass	Display...	Combin...	RDB	Delta [p...	Theo. m...	Rank	Combin...	# Match...	# Misse...	MS Cov....	Pattern...	MSMS...
336.2318	$C_{23}H_{30}$...	13.022...	9.50	-1.14	336.23...	1	56.279...	2	2	58.682...	77.723...	(Collec...

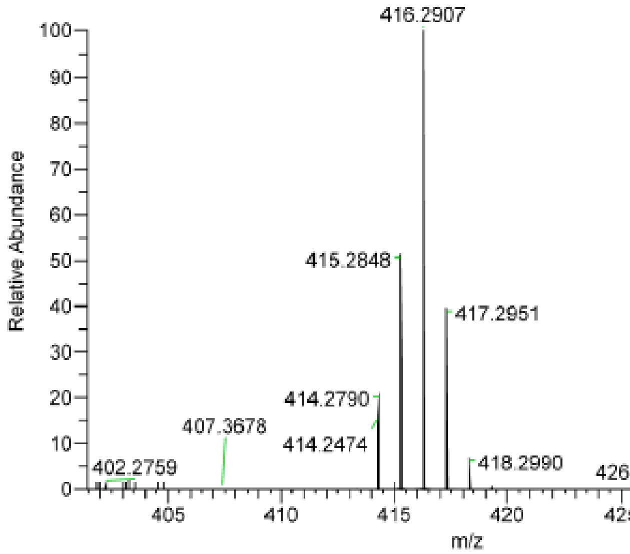

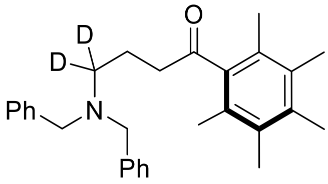

7b

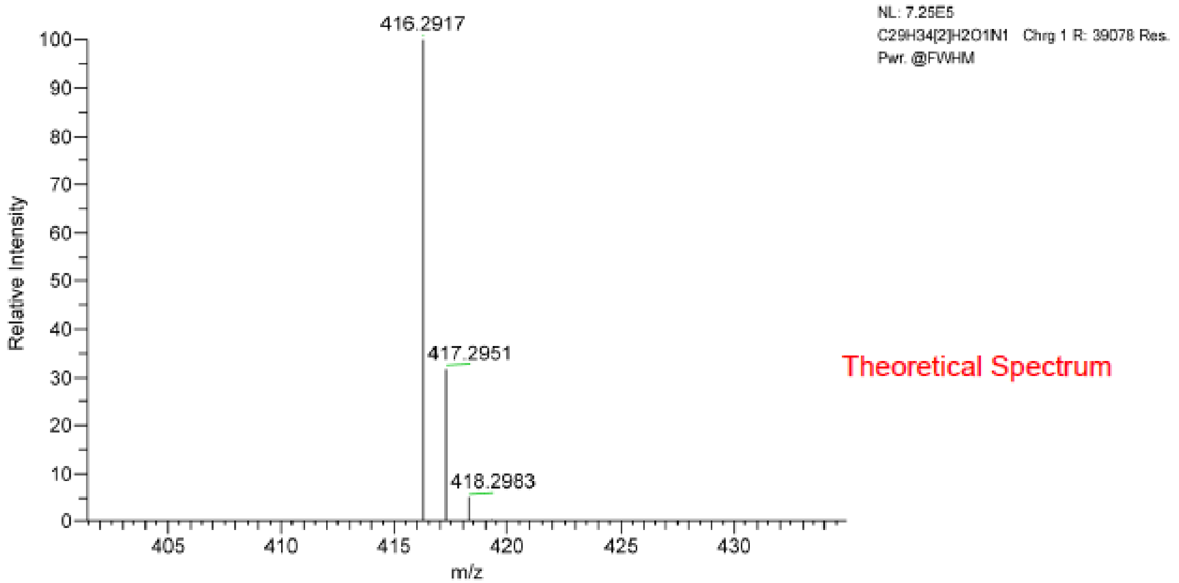

Peak Mass	Display...	Combin...	RDB	Delta [p...	Theo. m...	Rank	Combin...	# Match...	# Misse...	MS Cov....	Pattern...	MSMS...
416.2907	$C_{29}H_{34}{}^{2}$...	0	12.50	-2.32	416.29...	1	0	1	3	0	0	(Collec...

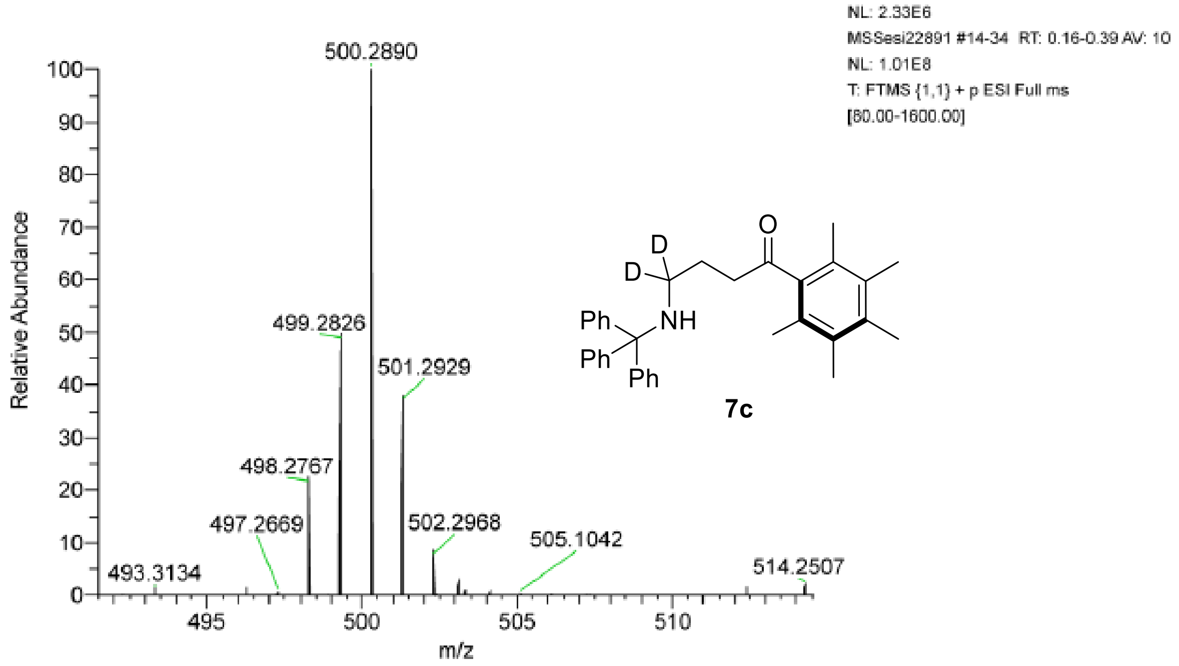

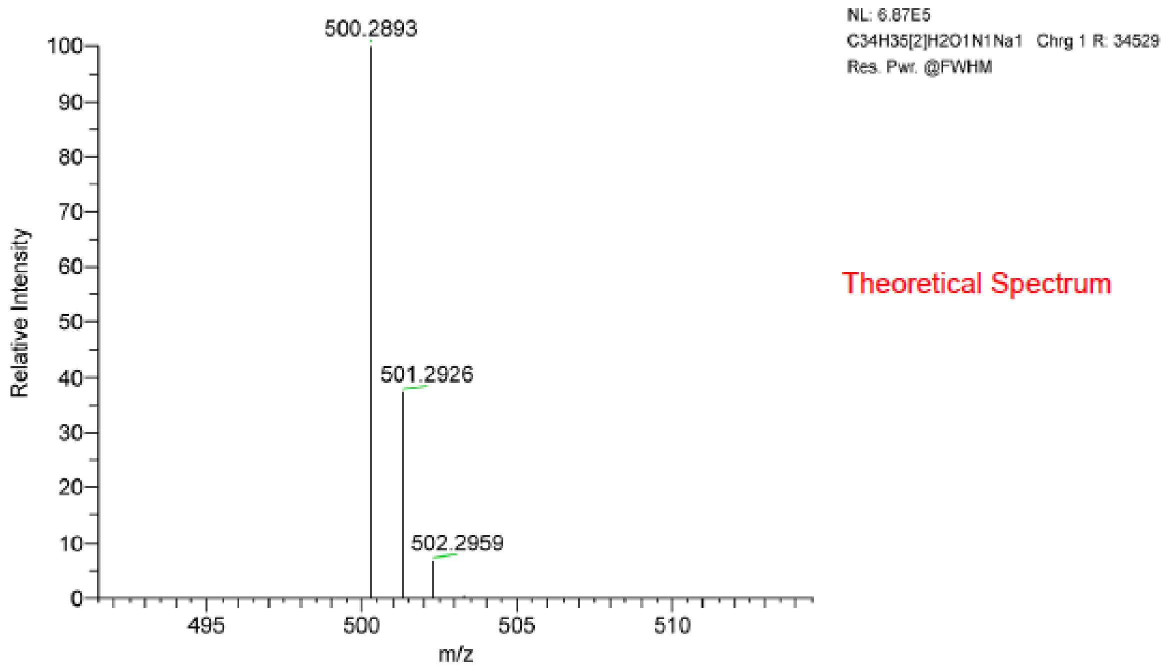

Peak Mass	Display...	Combin...	RDB	Delta [p...	Theo. m...	Rank	Combin...	# Match...	# Misse...	MS Cov....	Pattern...	MSMS...
500.2890	$C_{34}H_{35}$²...	0	16.50	-0.63	500.28...	1	0	1	3	0	0	(Collec...
